# Catalytic protodeboronation of pinacol boronic esters: formal anti-Markovnikov hydromethylation of alkenes†
†Electronic supplementary information (ESI) available. See DOI: 10.1039/c9sc02067e


**DOI:** 10.1039/c9sc02067e

**Published:** 2019-05-22

**Authors:** Florian Clausen, Marvin Kischkewitz, Klaus Bergander, Armido Studer

**Affiliations:** a Organisch-Chemisches Institut , Westfälische Wilhelms-Universität , Corrensstraβe 40 , 48149 Münster , Germany . Email: studer@uni-muenster.de

## Abstract

Pinacol boronic esters are highly valuable building blocks in organic synthesis. In contrast to the many protocols available on the functionalizing deboronation of alkyl boronic esters, protodeboronation is not well developed. Herein we report catalytic protodeboronation of 1°, 2° and 3° alkyl boronic esters utilizing a radical approach. Paired with a Matteson–CH_2_–homologation, our protocol allows for formal anti-Markovnikov alkene hydromethylation, a valuable but unknown transformation. The hydromethylation sequence was applied to methoxy protected (–)-Δ8-THC and cholesterol. The protodeboronation was further used in the formal total synthesis of δ-(*R*)-coniceine and indolizidine 209B.

## Abstract 2

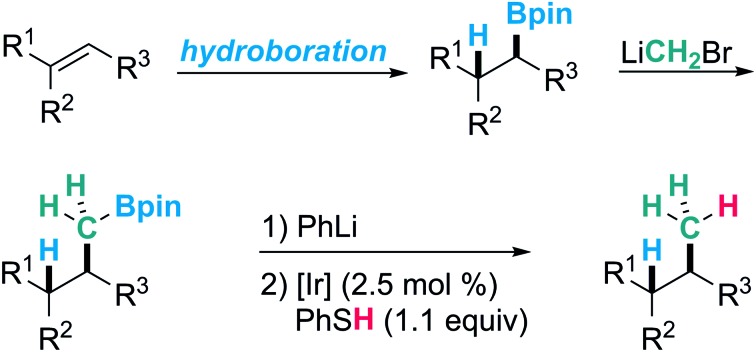
Methane is formally added to alkenes *via* sequential hydroboration, Matteson-CH_2_-homologation and radical protodeboronation.

## 

Organoboron compounds are highly valuable building blocks in organic synthesis.^1^,^2^ The most important application to be named is the Suzuki–Miyaura-coupling.^3^–^5^ Moreover, the boron moiety can be converted into a broad range of functional groups.^2^,^6^ These transformations include oxidations,^7^ aminations,^8^–^10^ halogenations,^11^,^12^ and C–C-bond-formations such as alkenylations,^13^,^14^ alkynylations^15^ and arylations.^16^ To access the B-building blocks, various borylation approaches have been developed over the years^17^–^19^ including the prominent asymmetric hydroboration reaction reported by H. C. Brown in 1961.^20^

However, considering synthetic applications, drawbacks associated with organoboranes are their limited air and moisture stability.^21^ Along these lines, the introduction of the more stable boronic ester moiety has significantly expanded the scope of boron chemistry. In particular the pinacol boronic esters, which are usually bench stable,^22^ easy to purify and often even commercially available have played a prominent role. These features are attractive for chemical transformations, where the valuable boron moiety remains in the product (homologations,^23^–^25^ conjunctive cross couplings^26^ or radical-polar crossover reactions^27^–^32^). However, the increased stability also rises new challenges, considering the removal of the boron moiety at the end of a sequence if required. While boranes undergo efficient protodeboronation with propionic acid by protonolysis^33^–^35^ or hydrogenation at elevated temperatures,^36^,^37^ boronic esters usually do not.^21^,^22^ We further noted that the protodeboronation of unactivated alkyl and especially primary alkyl boronic esters is underexplored. Reduction of 3° alkyl boronic esters was recently investigated by Aggarwal and coworkers; however, their method is only applicable to the protodeboronation of activated benzylic substrates.^38^ In 2005, Renaud and coworkers described an efficient sequence for the formal hydrogenation of unactivated alkenes to alkanes using a hydroboration-deboronation strategy (Scheme 1a).^39^ The protodeboronation of *in situ* generated catechol boronic esters proceeds *via* a radical chain reaction, but this reaction is limited to the more expensive catechol boronic esters and works well for 2° alkyl B-esters.^40^

**Scheme 1:**
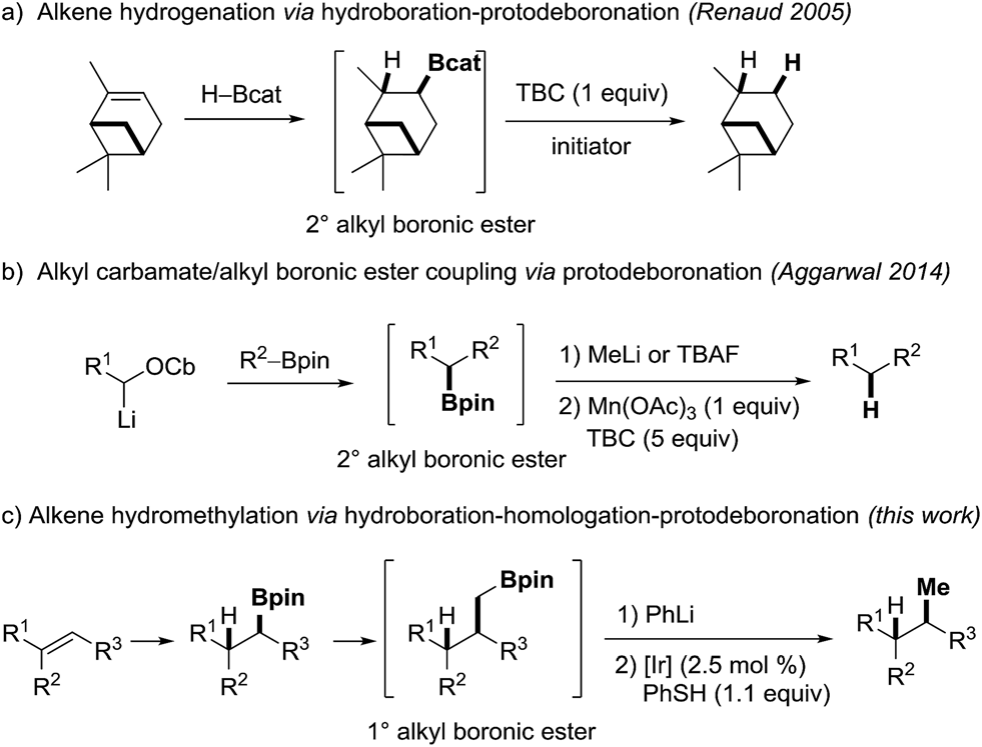
Protodeboronation and its application in synthesis.

An elegant C–C-coupling/protodeboronation strategy was introduced in 2014 by Aggarwal and coworkers (Scheme 1b).^41^ In this sequence, the protodeboronation of 2° alkyl pinacol boronic esters was achieved by oxidation of their corresponding methyl boron ate complexes with a stoichiometric amount of manganese(iii)acetate. The resulting secondary alkyl radicals are then reduced with 4-*tert*-butylcatechol (TBC). In contrast to the Renaud process, the latter protodeboronation works with pinacol boronic esters but does not proceed on primary B-esters (see below) and a large amount of reductant is required.

During a metabolite study, we targeted an anti-Markovnikov-type hydromethylation of a trisubstituted alkene and noted that different alkene hydromethylation approaches have been published over the years.^42^ However, all protocols developed so far provide the Markovnikov product. We therefore envisioned a hydroboration/CH_2_-homologation/protodeboronation strategy to achieve anti-Markovnikov alkene hydromethylation (Scheme 1c). In this sequence, the regioselectivity is controlled in the initial hydroboration step, where suitable methods are available.^43^,^44^ The methylene unit can readily be introduced *via* established Matteson-homologation using CH_2_Br_2_ as the methylene source. The real challenge turned out to be the final protodeboronation of the intermediate 1° alkyl boronic ester. The conditions reported by Aggarwal^41^ were ineffective for our systems and also other methods failed. We therefore initiated a project along those lines and aimed at a catalytic protodeboronation using boron ate complexes as substrates. Single electron oxidation by a redox catalyst should generate the corresponding alkyl radicals.^45^–^47^ Reduction of these alkyl radicals to give the targeted products should be achieved by thiols, since they are known to be efficient H-donors and the resulting thiyl radicals can be reduced by the photoredox catalyst thereby regenerating the initial oxidation state of the catalyst.^48^–^51^ Hence, an external stoichiometric oxidant is not required increasing the economy of the protodeboronation.

We commenced our investigations by testing different boron ate complexes derived from boronic ester **1a**. In each case, the ate complex was generated in Et_2_O. After removal of the ether, the residual ate complex was reacted with 2.5 mol% of the Ir-catalyst **PC1** and thiophenol (1.1 equiv.) in MeOH/acetone at room temperature under blue light irradiation. B-ate complexes derived from TBAF,^47^ DMAP,^47^ PPh_3_ (ref. 47) and 3-quinolidinol^47^ (1.1 equivalent each) did not afford the desired product **2a**. However, with PhLi, the target **2a** was obtained in 79% yield (Scheme 2). Phenyl boronic ester **3** was formed as the stoichiometric byproduct. Notably, in a proof of principle experiment we demonstrated that the cascade also works efficiently by using catalytic amounts of the thiol (10 mol%) in combination with diphenyl phosphate (1.1 equiv.) as the external proton source (95%, see ESI†). However, by using 50 mol% thiophenol in MeOH/acetone, the yield dropped significantly, showing the necessity of the phosphate in the thiol catalytic procedure. Since thiophenol is cheap, we decided to use the stoichiometric thiol protocol for the scope studies.

**Scheme 2:**
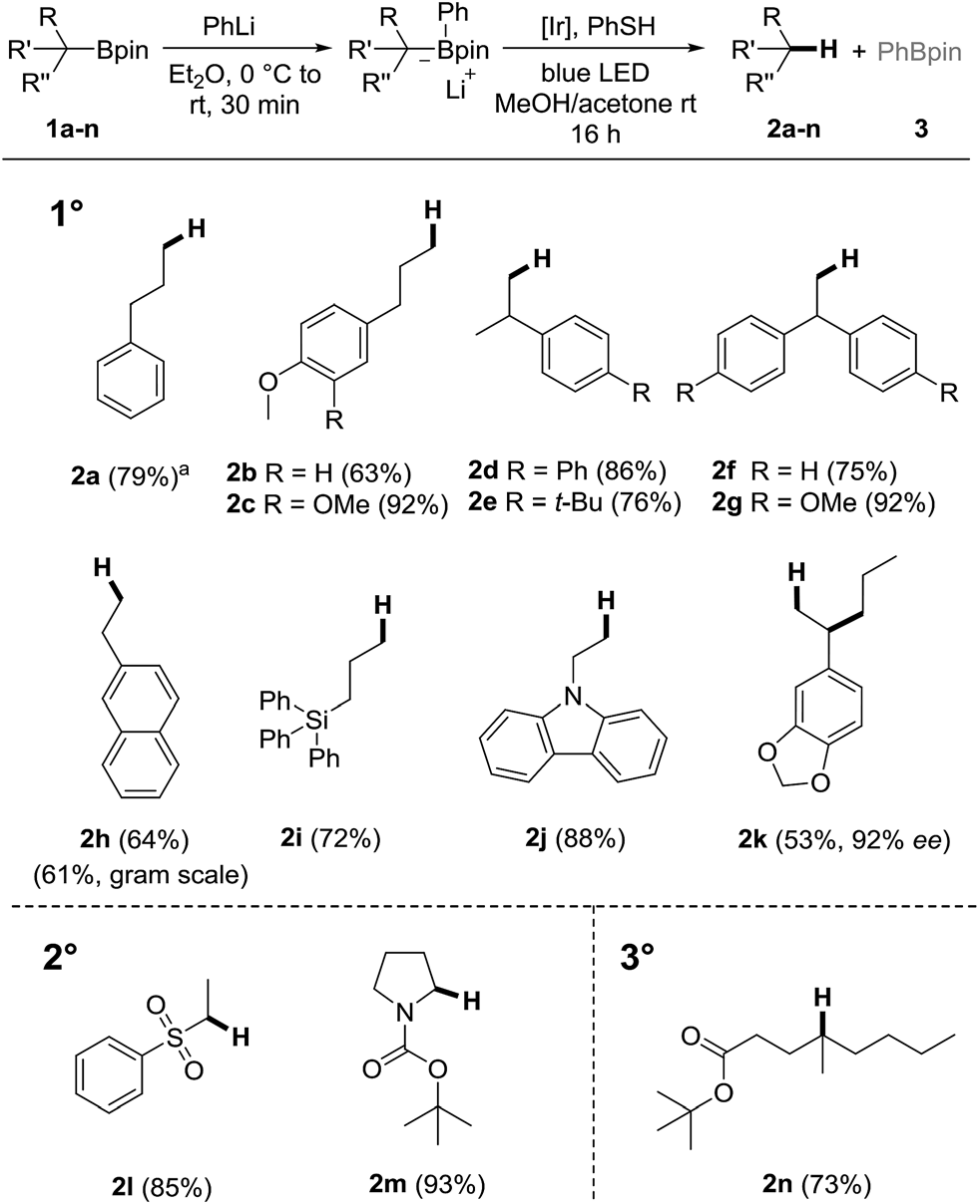
Substrate scope of the photoredox catalyzed protodeboronation. Conditions: **1a–n** (0.2 mmol), PhLi (0.22 mmol), Et_2_O (2 mL), thiophenol (0.22 mmol), Ir-catalyst **PC1** (2.5 mol%) in MeOH/acetone (1 : 1, 2 mL). Isolated yields, unless otherwise noted. ^a^Yield determined by GC. [Ir] = Ir(dFCF_3_ppy)_2_(dtbbpy)PF_6_ (**PC1**).

Under optimized conditions, various primary alkyl boronic esters **1b–n** were tested (Scheme 2). Reactions proceeded smoothly and the products **2b–n** were isolated in moderate to excellent yields (52–93%). Electron-rich arenes are well tolerated, as documented by the successful preparation of **2b–c**, **2g** and **2k**. Furthermore, substrates bearing a triphenyl silane (**1i**) and a carbazole moiety (**1j**) performed well and the desired products **2i–j** were isolated in 72% and 88% yield, respectively. As expected, the protodeboronation of enantioenriched boronic ester **1k**, readily prepared *via* stereoselective hydroboration,^52^ occurs with high stereospecificity (96% es). The reason for the slight drop in ee might be reversible H-abstraction at the benzylic position by the thiyl radical. In any case, this sequence represents a new strategy for the asymmetric synthesis of aryl dialkyl methanes of type **2k**.

The method was also applied to the protodeboronation of the 2° alkyl boronic esters **1l** and **1m** to yield the Boc-protected pyrrolidine **2l** and sulfone **2m** in 85 and 93% yield. Furthermore, the 3° alkyl boronic ester **1n** bearing a *tert*-butylester group was smoothly protodeboronated (73%). A gram scale experiment (preparation of **2h**) proved the robustness of the procedure (61%).

In order to verify that the protodeboronation occurs *via* a radical pathway, a probe experiment was conducted. The phenyl boron ate complex derived from the cyclopropylmethyl substituted boronic ester **1o** reacted under standard conditions to the ring opened alkene **2o** (*E*/*Z* = 4 : 1, 84%), clearly supporting the radical nature of the cascade (Scheme 3a).^53^ A possible mechanism is depicted in Scheme 3b. Photoexcited **PC1** first oxidizes the boron ate complex **A** to generate an alkyl radical R˙ along with Ph-Bpin. C-radical R˙ then abstracts an H-atom from thiophenol to form the product **2** and the phenyl thiyl radical, which is reduced by the Ir(ii)-complex to regenerate the starting Ir(iii)-complex **PC1**.

**Scheme 3:**
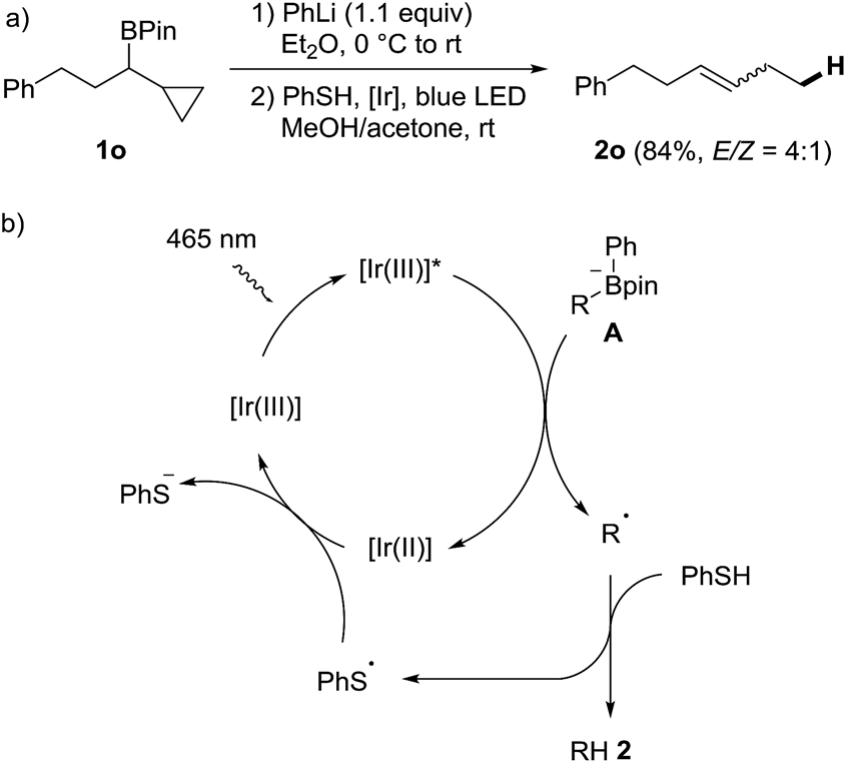
Radical probe experiment and proposed mechanism.

To further document the potential of the protodeboronation, we applied the novel protocol to the formal total synthesis of two indolizidine-core containing secondary metabolites, δ-(*R*)-coniceine (**9**) and indolizidine 209B (**13**) (Scheme 4). Starting with commercial *N*-Boc-pyrrolidine (**4**), enantiopure boronic ester **5** was prepared *via* a (+)-sparteine mediated asymmetric lithiation/borylation.^54^–^56^ The ester moiety was introduced *via* stereospecific radical-induced 1,2-migration^27^,^28^ of the corresponding *in situ* generated vinyl and isopropenyl boron ate complexes with α-iodo acetates as radical precursors to provide **6** and **10** in 52% and 65% yield. In the coniceine route, **6** was directly subjected to the protodeboronation to provide the pyrrolidine **7** in 61% yield. After ester cleavage and Boc-deprotection, the corresponding carboxylic acid was cyclised with EDC and DMAP to yield the bicyclic lactam **8** in 87% yield, completing the formal total synthesis^57^ of δ-(*R*)-coniceine (**9**). A crucial step in the indolizidine 209B synthesis was the build-up of the additional C-8 stereocenter *via* a stereoselective protodeboronation. To this end, the 3° boronic ester **10** was converted into the rigid indolizidine precursor **11***via* Boc-deprotection and lactamization (68%). Protodeboronation by using the less nucleophilic (3,5-bis(trifluoromethyl)phenyl)lithium for boron ate complex formation in place of PhLi, to prevent aryl addition to the lactam moiety, afforded the indolizidine **12** in 59% yield and good diastereoselectivity (dr = 5 : 1). Final transformation into indolizidine 209B (**13**) can be achieved by Grignard-addition to the lactam followed by stereoselective reductive deoxygenation.^58^

**Scheme 4:**
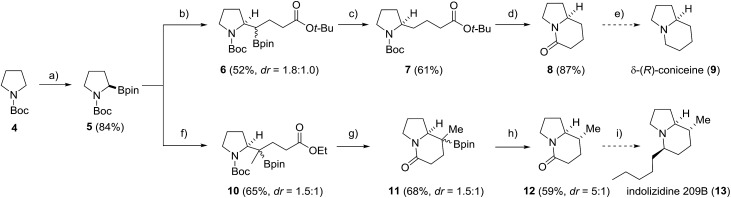
Formal total synthesis of δ-(*R*)-coniceine (**9**) and indolizidine 209B (**13**). Reagents and conditions: (a) *s*-BuLi (1.2 equiv.), (+)-sparteine (1.2 equiv.) Et_2_O, –78 °C, 3 h then isopropyl pinacol borate (1.3 equiv.), –78 °C, 2 h; (b) vinyllithium (1.3 equiv.), Et_2_O, –78 °C to rt, 30 min then *tert*-butyl 2-iodoacetate (2.0 equiv.), MeCN, *hν*, 50 °C, 24 h; (c) PhLi (1.1 equiv.), Et_2_O, 0 °C to rt, 30 min then PhSH (1.1 equiv.), **PC1** (2.5%), blue LED, MeOH/acetone, 16 h; (d) HCl in dioxane, rt, 8 h then EDC·HCl (2.0 equiv.), DMAP (20 mol%), Hünig's base (2.0 equiv.), CH_2_Cl_2_, rt, over night; (e) see ref. 57; (f) isopropenyllithium (1.3 equiv.), Et_2_O, –78 °C to rt, 30 min then ethyl 2-iodoacetate (5.0 equiv.), MeCN, *hν*, 50 °C, 24 h; (g) TFA (10 eq.), CH_2_Cl_2_, 0 °C to rt, 2 h then NEt_3_ (20 equiv.), CH_2_Cl_2_, rt, 12 h; (h) (3,5-bis(trifluoromethyl)phenyl)lithium (1.3 equiv.), Et_2_O, –78 °C, 1 h then PhSH (1.3 equiv.), **PC1** (2.5%), blue LED, MeOH/acetone, 16 h; (i) see ref. 58.

Finally, we focused on the formal alkene hydromethylation sequence (Scheme 5). Initial studies were conducted with the boronic esters **14a–d** which can be obtained *via* hydroboration of the corresponding styrene derivatives (see the ESI†). The Matteson-CH_2_-homologation was carried out with *in situ* generated CH_2_BrLi in THF at low temperature. After removal of the solvent, the crude homologated boronic esters **15a–d** were directly subjected to the protodeboronation to provide the targeted compounds **16a–d** in 65–66% overall yields. The heteroarenes **14e**, **14f** and a silylated boronic ester were eligible substrates and provided **16e–g** in 51–70% yield. When the OTs-protected phenol **14h** was subjected to the sequence, the deprotected phenol **16h** resulted (48%). The sequence was also applied to the hydromethylation of more complex alkenes such as the fluorine containing system **14d** to obtain the hydromethylated compound **16d** in 66% yield. The hetero arenes **14e** and **14f** were tolerated and provided the products **16e** and **16f** in 51–55% and yields. When the OTs-protected phenol **14h** was subjected to the sequence the deprotected compound **16h** was isolated in 48% yield. The method was also applied to cyclic olefins such as the cyclohexane derivative **14i** to give the product **16i** in 61% yield, the methyl protected (–)-Δ^8^-THC **14j** and the cholesterol derivative **14k**. Stereo- and regioselective hydroboration using catecholborane under neat conditions^16^ followed by transesterification with pinacol gave the 2° alkyl boronic esters **14j** and **14k** in 55% to 65% yield as single diastereoisomers (see ESI†). CH_2_-homologation and protodeboronation afforded the methylated products **16j** and **16k** in excellent overall yields (89% and 90%), convincingly documenting the potential of our novel sequence.

**Scheme 5:**
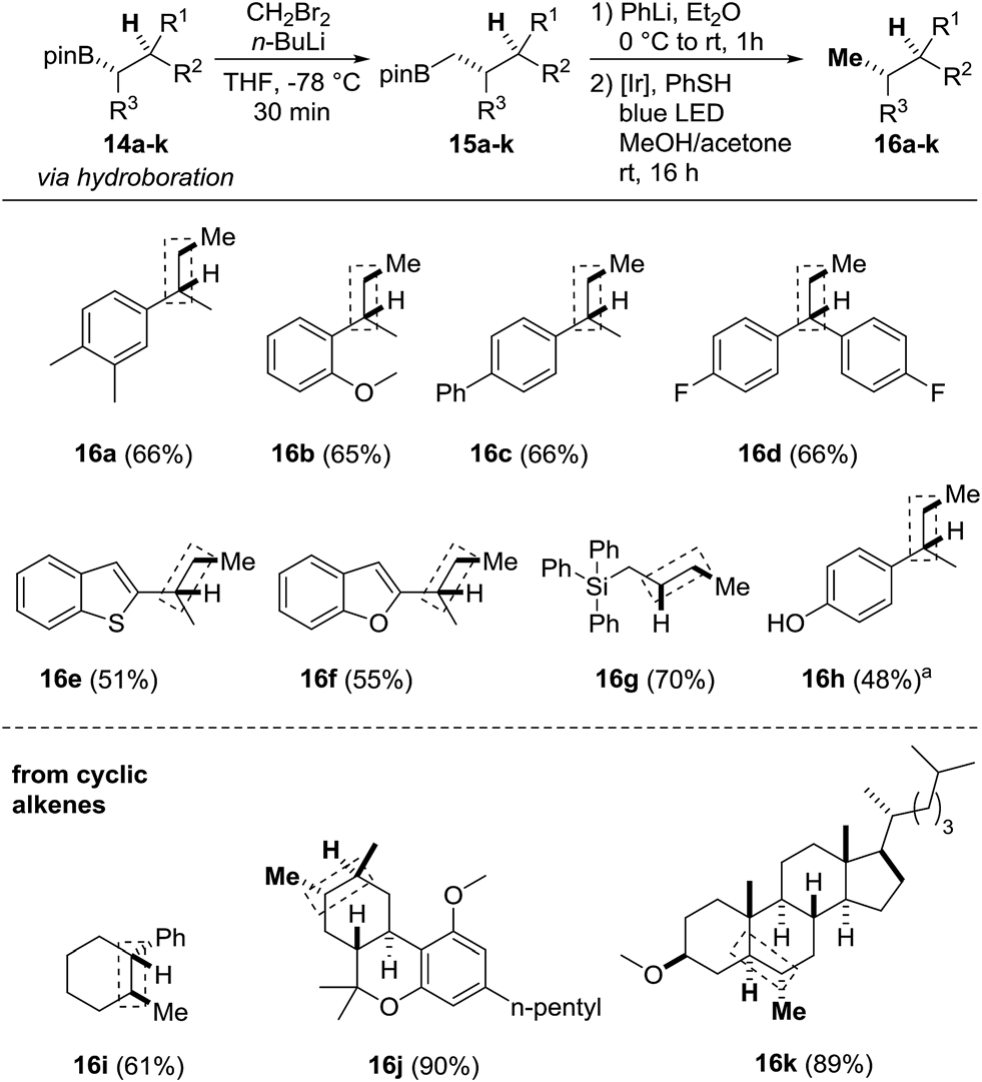
Formal alkene hydromethylation. Homologation: **14a–k** (0.05–0.2 mmol, 1.0 equiv.), CH_2_Br_2_ (2.0–3.0 equiv.), *n*-butyllithium (1.5–2.0 equiv.) in THF or Et_2_O: protodeboronation: **15a–k** (0.05–0.2 mmol, 1.0 equiv., used as crude from homologation step), PhLi (1.1 equiv.), Et_2_O (0.5–2.0 mL), thiophenol (1.1 equiv.), **PC1** (2.5 mol%) in MeOH/acetone (1 : 1, 0.5–2.0 mL). Yield corresponds to the two-step homologation-protodeboronation sequence. For the hydroboration step, we refer to the ESI.† ^a^Derived from O-Ts-protected phenol, O–S cleavage during reaction.

In summary, we have developed the first catalytic protodeboronation of unactivated 1°, 2° and 3° alkyl pinacol boronic esters utilizing photoredox catalysis. We were able to carry out a formal methane addition to various alkenes combining the protodeboronation protocol with a Matteson-CH_2_-homologation. The sequence was applied to the hydromethylation of secondary metabolite derivatives, a (–)-Δ^8^-THC derivative and *O*-methylated cholesterol. Furthermore, the protodeboronation was successfully also used in formal total syntheses of δ-(*R*)-coniceine (**9**) and indolizidine 209B (**13**).

## Conflicts of interest

The authors declare no conflict of interest.

## Supplementary Material

Supplementary information
