# Supplementary material for: Catalytic protodeboronation of pinacol boronic esters: formal anti-Markovnikov hydromethylation of alkenes
Source: Chem Sci. 2019 May 22;10(24):6210–4. doi: 10.1039/c9sc02067e (PMC6585874; doi:10.1039/c9sc02067e)

Electronic Supplementary Material (ESI) for Chemical Science.
This journal is © The Royal Society of Chemistry 2019

Supporting Information

Catalytic Protodeboronation of Pinacol Boronic Esters: Formal Anti-Markovnikov Hydromethylation of Alkenes

Florian Clausen, Marvin Kischkewitz, Klaus Bergander, and Armido Studer*

Table of Contents

1. General Information 5
2. Experimental Procedures 6
2.1. General Procedures 6
2.1.1 General procedure for hydroboration using BCl_3 and Et_3SiH (GP1) 6
2.1.2 General procedure for hydroboration using catecholborane (GP2) 6
2.1.3 General procedure for MATTESON-CH_2-homologation (GP3) 7
2.1.4 General procedure for protodeboronation of boronic acid pinacol esters (GP4) 7
2.2 Preparation of Starting Materials 8
2.2.1 4,4'-(Ethene-1,1-diyl)bis(methoxybenzene) 8
2.2.2 1-Methoxy-2-(prop-1-en-2-yl)benzene 8
2.2.3 5-(Pent-1-en-2-yl)benzo[d][1,3]dioxole 9
2.2.4 1,2-Dimethyl-4-(prop-1-en-2-yl)benzene 9
2.2.5 (*R*)-2-(6-Bromopyridin-2-yl)-4-isopropyl-4,5-dihydrooxazole 10
2.2.6 (*R*)-1-(6-(4-Isopropyl-4,5-dihydrooxazol-2-yl)pyridin-2-yl)ethan-1-one 10
2.2.7 (*R*,*E*)-*N*-(2,6-Diisopropylphenyl)-1-(6-(4-isopropyl-4,5-dihydrooxazol-2-yl)pyridin-2-yl)ethan-1-imine 11
2.2.8 Catalyst for asymmetric hydroboration (C1) 11
2.2.9 3-Phenylpropyl diisopropylcarbamate (CARB1) 11
2.3 Synthesis of Boronic Esters 12
2.3.1 2-(3-(3,4-Dimethoxyphenyl)propyl)-4,4,5,5-tetramethyl-1,3,2-dioxaborolane (1c) 12
2.3.2 2-(2-([1,1'-Biphenyl]-4-yl)propyl)-4,4,5,5-tetramethyl-1,3,2-dioxaborolane (1d, 14c) 12
2.3.3 2-(2-(4-(*tert*-Butyl)phenyl)propyl)-4,4,5,5-tetramethyl-1,3,2-dioxaborolan (1e) 13
2.3.4 2-(2,2-Diphenylethyl)-4,4,5,5-tetramethyl-1,3,2-dioxaborolane (1f) 13
2.3.5 2-(2,2-Bis(4-methoxyphenyl)ethyl)-4,4,5,5-tetramethyl-1,3,2-dioxaborolane (1g) 14
2.3.6 4,4,5,5-Tetramethyl-2-(2-(naphthalen-2-yl)ethyl)-1,3,2-dioxaborolane (1h) 14
2.3.7 Triphenyl(3-(4,4,5,5-tetramethyl-1,3,2-dioxaborolan-2-yl)propyl)silane (1i, 14g) 14
2.3.8 2-(2-(Benzo[d][1,3]dioxol-5-yl)pentyl)-4,4,5,5-tetramethyl-1,3,2-dioxaborolane (1k) 15
2.3.9 4,4,5,5-Tetramethyl-2-(1-(phenylsulfonyl)ethyl)-1,3,2-dioxaborolane (1l) 15
2.3.10 *tert*-Butyl (*R*)-2-(4,4,5,5-tetramethyl-1,3,2-dioxaborolan-2-yl)pyrrolidine-1-carboxylate (1m) 16
2.3.11 *tert*-Butyl 4-methyl-4-(4,4,5,5-tetramethyl-1,3,2-dioxaborolan-2-yl)octanoate (1n) 16
2.3.12 2-(1-Cyclopropyl-3-phenylpropyl)-4,4,5,5-tetramethyl-1,3,2-dioxaborolan (1o) 17
2.3.13 2-(2-(3,4-Dimethylphenyl)propyl)-4,4,5,5-tetramethyl-1,3,2-dioxaborolane (14a) 17
2.3.14 2-(2-(2-Methoxyphenyl)propyl)-4,4,5,5-tetramethyl-1,3,2-dioxaborolane (14b) 18
2.3.15 2-(2,2-Bis(4-fluorophenyl)ethyl)-4,4,5,5-tetramethyl-1,3,2-dioxaborolane (14d) 18
2.3.16 2-(2-(Benzothiophen-2-yl)propyl)-4,4,5,5-tetramethyl-1,3,2-dioxaborolane (14e) 19

2.3.17 2-(2-(Benzofuran-2-yl)propyl)-4,4,5,5-tetramethyl-1,3,2-dioxaborolane (14f) 19
2.3.18 2-(2-(2-Methoxyphenyl)propyl)-4,4,5,5-tetramethyl-1,3,2-dioxaborolane (14h) 19
2.3.19 *rac*-4,4,5,5-Tetramethyl-2-((1*R*,2*R*)-2-phenylcyclohexyl)-1,3,2-dioxaborolane (14i) 20
2.3.20 2-((6aR,8S,9S,10aR)-1-Methoxy-6,6,9-trimethyl-3-pentyl-6a,7,8,9,10,10a-hexahydro-6H-benzo[c]chromen-8-yl)-4,4,5,5-tetramethyl-1,3,2-dioxaborolane (14j) 20
2.3.21 2-((3*S*,5*R*,6*S*,8*S*,9*S*,10*R*,13*R*,14*S*,17*R*)-3-Methoxy-5,10,13-trimethyl-17-((*R*)-6-methylheptan-2-yl)hexadecahydro-1*H*-cyclopenta[a]phenanthren-6-yl)-4,4,5,5-tetramethyl-1,3,2-dioxaborolane (14k) 21
2.4 MATTESON-type CH_2-homologations 22
2.4.1 2-(3-(3,4-Dimethylphenyl)butyl)-4,4,5,5-tetramethyl-1,3,2-dioxaborolane (15a) 22
2.4.2 2-(3-(2-Methoxyphenyl)butyl)-4,4,5,5-tetramethyl-1,3,2-dioxaborolane (15b) 22
2.4.3 2-(3-([1,1'-Biphenyl]-4-yl)butyl)-4,4,5,5-tetramethyl-1,3,2-dioxaborolane (15c) 22
2.4.4 2-(3,3-bis(4-fluorophenyl)propyl)-4,4,5,5-tetramethyl-1,3,2-dioxaborolane (15d) 23
2.4.5 2-(3-(Benzo[b]thiophen-2-yl)butyl)-4,4,5,5-tetramethyl-1,3,2-dioxaborolane (15e) 23
2.4.6 2-(3-(Benzofuran-2-yl)butyl)-4,4,5,5-tetramethyl-1,3,2-dioxaborolane (15f) 23
2.4.7 Triphenyl(4-(4,4,5,5-tetramethyl-1,3,2-dioxaborolan-2-yl)butyl)silane (15g) 23
2.4.8 4-(4-(4,4,5,5-Tetramethyl-1,3,2-dioxaborolan-2-yl)butan-2-yl)phenyl 4-methylbenzenesulfonate (15h) 23
2.4.9 2-(3-(2-methoxyphenyl)butyl)-4,4,5,5-tetramethyl-1,3,2-dioxaborolane (15i) 24
2.4.10 2-(((6a*R*,8*S*,9*S*,10a*R*)-1-Methoxy-6,6,9-trimethyl-3-pentyl-6a,7,8,9,10,10a-hexahydro-6*H*-benzo[c]chromen-8-yl)methyl)-4,4,5,5-tetramethyl-1,3,2-dioxaborolane (15j) 24
2.4.11 2-(((3*S*,5*R*,6*S*,8*S*,9*S*,10*S*,13*R*,14*S*,17*R*)-3-Methoxy-10,13-dimethyl-17-((*R*)-6-methylheptan-2-yl)hexadecahydro-1*H*-cyclopenta[a]phenanthren-6-yl)methyl)-4,4,5,5-tetramethyl-1,3,2-dioxaborolane (15k) 24
2.5 Protodeborylation reactions 24
2.5.1 Propylbenzene (2a) 24
2.5.2 1-Methoxy-4-propylbenzene (2b) 26
2.5.3 1,2-Dimethoxy-4-propylbenzene (2c) 26
2.5.4 4-Isopropyl-1,1'-biphenyl (2d) 26
2.5.5 1-(*tert*-Butyl)-4-isopropylbenzene (2e) 26
2.5.6 1,1-Diphenylethan (2f) 27
2.5.7 1,1-Bis(4-methoxyphenyl)ethan (2g) 27
2.5.8 2-Ethylnaphthalene (2h) 27
2.5.9 Triphenylpropylsilane (2i) 28
2.5.10 *N*-Methylcarbazole (2j) 28
2.5.11 5-(pentan-2-yl)benzo[d][1,3]dioxole (2k) 28
2.5.12 Phenylethylsulfon (2l) 28
2.5.13 *tert*-Butyl pyrrolidine-1-carboxylate (2m) 29
2.5.14 *tert*-Butyl-4-methyloctanoate (2n) 29
2.5.15 4-(*sec*-Butyl)-1,2-dimethylbenzene (16a) 29

2.5.16 1-(*sec*-Butyl)-2-methoxybenzene (16b) 30
2.5.17 4-(*sec*-Butyl)-1,1'-biphenyl (16c) 30
2.5.18 4,4'-(Propane-1,1-diyl)bis(fluorobenzene) (16d) 30
2.5.19 2-(*sec*-Butyl)benzo[b]thiophene (16e) 31
2.5.20 2-(*sec*-Butyl)benzofuran (16f) 31
2.5.21 Butyltriphenylsilane (16g) 31
2.5.22 4-(*sec*-Butyl)phenol (16h) 32
2.5.23 *rac*-((1*S*,2*S*)-2-Methylcyclohexyl)benzene (16i) 32
2.5.24 (6a*R*,8*S*,9*S*,10a*R*)-1-Methoxy-6,6,8,9-tetramethyl-3-pentyl-6a,7,8,9,10,10a-hexahydro-6*H*-benzo[c]chromene (16j) 32
2.5.25 (3*S*,5*R*,6*S*,8*R*,9*S*,10*S*,13*R*,14*S*,17*R*)-3-Methoxy-6,10,13,14-tetramethyl-17-((*R*)-6-methylheptan-2-yl)hexadecahydro-1*H*-cyclopenta[a]phenanthrene (16k) 33
2.6 Synthesis of the indolizidine alkaloides 34
2.6.1 *tert*-Butyl (S)-2-((R)-4-(*tert*-butoxy)-4-oxo-1-(4,4,5,5-tetramethyl-1,3,2-dioxaborolan-2-yl)butyl)pyrrolidine-1-carboxylate / *tert*-Butyl (S)-2-((S)-4-(*tert*-butoxy)-4-oxo-1-(4,4,5,5-tetramethyl-1,3,2-dioxaborolan-2-yl)butyl)pyrrolidine-1-carboxylate (6) 34
2.6.2 *tert*-Butyl (*S*)-2-((*S*)-5-ethoxy-5-oxo-2-(4,4,5,5-tetramethyl-1,3,2-dioxaborolan-2-yl)pentan-2-yl)pyrrolidine-1-carboxylate / *tert*-Butyl (*S*)-2-((*R*)-5-ethoxy-5-oxo-2-(4,4,5,5-tetramethyl-1,3,2-dioxaborolan-2-yl)pentan-2-yl)pyrrolidine-1-carboxylate (10) 35
2.6.3 *tert*-Butyl (*R*)-2-(4-(*tert*-butoxy)-4-oxobutyl)pyrrolidine-1-carboxylate (7) 35
2.6.4 (*R*)-Hexahydroindolizidine-5(1*H*)-one (8) 36
2.6.5 (8*R*,8a*S*)-8-Methyl-8-(4,4,5,5-tetramethyl-1,3,2-dioxaborolan-2-yl) hexahydroindolizidine-5(1*H*)-one / (8*S*,8a*S*)-8-Methyl-8-(4,4,5,5-tetramethyl-1,3,2-dioxaborolan-2-yl)hexahydroindolizidin-5(1*H*)-one (11) 37
2.6.6 (8*R*,8a*S*)-8-Methylhexahydroindolizine-5(1*H*)-one / (8*S*,8a*S*)-8-Methylhexahydroindolizine-5(1*H*)-one (12) 37
2.7 Mechanistic Studies 38
References 38
3 Appendix 40
3.1 Chiral HPLC 40
3.1.1 2-(2-(benzo[d][1,3]dioxol-5-yl)pentyl)-4,4,5,5-tetramethyl-1,3,2-dioxaborolane 40
3.1.2 (*S*)-1-Methoxy-4-(pentan-2-yl)benzene (2k) 41
3.2 NMR Spectra 42
3.2.1 Starting materials 42
3.2.2 Boronic esters 49
3.2.3 Protodeboronation reactions 73
3.2.4 Mechanistic Studies 98
3.2.5 Synthesis of the indolizidine alkaloides 99

1. General Information

All reactions involving air or moisture sensitive reagents were carried out in flame-dried glass ware under argon atmosphere using standard SCHLENK techniques. **Solvents** used in reactions were either freshly distilled or obtained in extra-dry grade from commercial sources. Diethyl ether (Et_2O) was refluxed over K and freshly distilled from K-Na-alloy (4:1) afterwords. Tetrahydrofuran (THF) was refluxed over Na and distilled from K afterwords. Acetone (99.8%, Extra Dry, AcroSeal®) and methanol (MeOH, 99.8%, Extra Dry over Molecular Sieve, AcroSeal®) were purchased from ACROS ORGANICS. Solvents for flash chromatography (**FC**) were freshly distilled before use. *n*-butyl lithium (*n*-BuLi, 1.6 M in hexanes) was purchased from ACROS ORGANICS. The boronic esters not listed in the experimental section were purchased from ABCR. All other chemicals were purchased from ABCR, ACROS ORGANICS, ALFA AESAR, FLUKA, SIGMA ALDRICH and TCI and were used as received. Flash chromatography was performed on MERCK silica gel 60 (40 – 63 µm) with an excess argon pressure up to 1.0 bar. MERCK silica gel 60 F254 plates were used for thin layer chromatography (TLC) using UV light (254/366 nm), $KMnO_4$ (1.5 g in 200 mL H_2O, 5 g $NaHCO_3$) for detection. Visible light reactions were irradiated with Blue LED (465 nm, 5 W). Melting points (**MP**) were determined with a STUART SMP10 and are uncorrected. Infrared spectra (**IR**) were measured on a DIGILAB 3100 FT-IR EXCALIBUR SERIES spectrometer and the position of the absorption bands is given in wave numbers ν (cm^{-1}). **^{1}H NMR** (300 MHZ, 400 MHz and 600 MHz), **^{13}C NMR** (75 MHz, 100 MHz and 151 MHz) and **^{11}B NMR** (96 MHz, 128 MHz and 192 MHz) spectra were measured on a BRUKER DPX 300, BRUKER AV 300, BRUKER AV 400 or an AGILENT DD2 600 spectrometer. The multiplicity of all signals were described as s (singlet), d (doublet), t (triplet), q (quartet), p (pentet), h(hextet), hept (heptet) and m (multiplet). Chemical shifts (δ in ppm) were referenced on the residual peak of $CDCl_3$ (**^{1}H NMR**: δ = 7.26; **^{13}C NMR**: δ = 77.0), C_6D_6 (**^{1}H NMR**: δ = 7.16; **^{13}C NMR**: δ = 128.06), DMSO-*d*6 (**^{1}H NMR**: δ = 2.50; **^{13}C NMR**: δ = 39.52). **HRMS ESI** (m/z) measurements were performed on a BRUKER MICROTOF. Chiral HPLC was performed on a Hewlett Packard Series 1100 **HPLC** using UV detection (210 nm, 230 nm, 250 nm, 260 nm). Separation was performed using CHIRALPAK®AD-H (4.6 x 250 nm x 5 µm, DAICEL CHEMICAL INDUSTRIES, LTD.) or CHIRALCEL®OJ-RH (4.5 x 150 nm x 5 µm, Daicel Chemical Industries, Ltd.) columns. Optical rotations were measured on a JASCO P-2000 polarimeter.

2. Experimental Procedures

2.1. General Procedures

2.1.1 General procedure for hydroboration using BCl_3 and Et_3SiH (GP1)

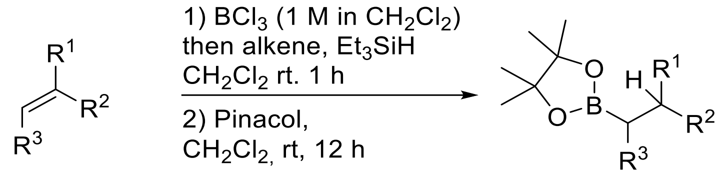

In analogy to a procedure of MATTESON and coworkers,[1] BCl_3 (1 M, CH_2Cl_2, 1.00-1.10 equiv.) was added to a SCHLENK tube containing a stir bar under inert atmosphere. The substrate and Et_3SiH (1.00 equiv.) were dissolved in CH_2Cl_2 (0.1 M – 1 M) and the thus generated solution was added dropwise to the BCl_3 at room temperature. After 1 h, pinacol (1.00-2.00 equiv.) was added as a solid portion wise (to avoid a vigorous evolution of HCl gas) at room temperature and the solution was stirred for another 12 h. Removal of the solvents *in vacuo* resulted in the crude boronic acid pinacol ester, which was further purified *via* column chromatography or bulb-to-bulb-distillation to obtain the pure boronic ester.

2.1.2 General procedure for hydroboration using catecholborane (GP2)

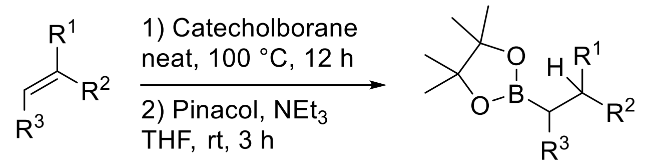

In analogy to a procedure by AGGARWAL and coworkers,[2] the substrate was dissolved in catecholborane (5.0 equiv.) in an inert atmosphere and added to a SCHLENK tube containing a stir bar. The tube was heated to 100 °C over a period of 12 h and allowed to cool to room temperature. The majority of the excess catecholborane was removed *in vacuo* and the crude catechol boronic ester was redissolved in THF (0.1 M). After the addition of pinacol (5.0 equiv.), NEt_3 (10 equiv.) was slowly added to the solution, which was then stirred for another 3 h. The solution was transferred to a separating funnel, washed with a saturated sodium bicarbonate solution and extracted with Et_2O (3x). The organic extracts were dried over magnesium sulfate and the solvent removed *in vacuo* to yield the crude boronic pinacol ester, which was further purified *via* column chromatography.

2.1.3 General procedure for MATTESON-CH_2-homologation (GP3)

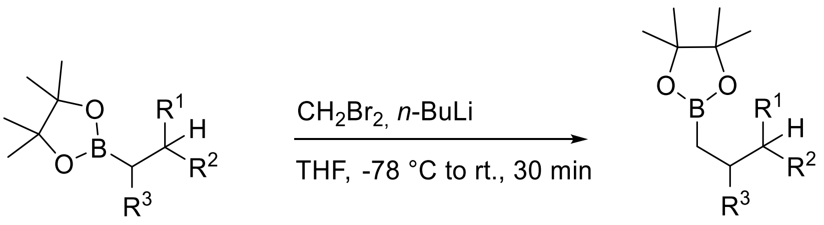

In analogy to a procedure by STUDER and coworkers,[3] the boronic ester and methylene bromide (2.0-3.0 equiv.) were dissolved in anhydrous THF or Et_2O (0.1 M) and cooled to -78 °C. With vigorous stirring, *n*-BuLi (1.6 M, *n*-hexane) was added dropwise and the reaction mixture was kept at that temperature for 30 min. The cooling bath was removed and the reaction was allowed to warm to room temperature. After removal of the solvent *in vacuo*, the crude boronic ester was used without further purification.

2.1.4 General procedure for protodeboronation of boronic acid pinacol esters (GP4)

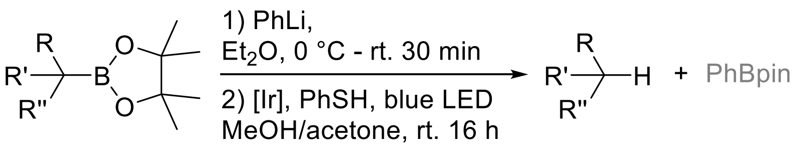

The boronic ester (0.20 mmol) was added to a SCHLENK-tube under an inert atmosphere and dissolved in Et_2O (2 mL, 0.1 M). After cooling to 0 °C phenyllithium (1.9 M, Bu_2O, 0.12 mL, 1.1 equiv.) was added dropwise and the reaction mixture was allowed to warm to room temperature and stirred for another 30 min. The solvent was removed *in vacuo* and the residue was taken up in methanol/acetone (1:1 mixture, 2 mL, 0.1 M) followed by the addition of $Ir(dFCF_3ppy)_2(dtbbpy)PF_6$ (5.0 mg, 2.5 mol%) and thiophenol (24 mg, 22 µL, 1.1 equiv.). After freeze pump thaw degassing of the mixture, the reaction was irradiated at ambient temperature for 16 h using a blue LED (see general informations). The mixture was diluted with Et_2O (30 mL) and washed with a saturated sodium bicarbonate solution. The organic layer was dried over magnesium sulfate and the solvent removed *in vacuo*. FC of the resulting residue afforded the protodeboronated product.

2.2 Preparation of Starting Materials

2.2.1 4,4'-(Ethene-1,1-diyl)bis(methoxybenzene)

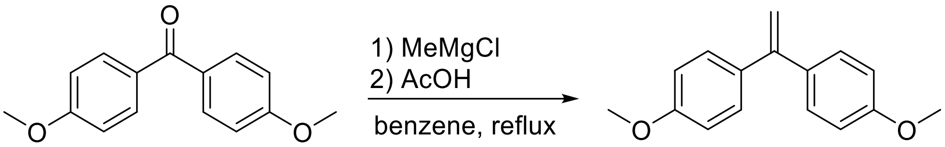

In a 250 mL two necked round bottom flask connected to a reflux condenser, bis(4-methoxyphenyl)methanone (5.00 g, 20.6 mmol, 1.00 equiv.) was dissolved in benzene (20 mL). At ambient temperature, MeMgCl (3.00 M, THF, 8.80 mmol, 1.30 equiv.) was added dropwise. Subsequently, the reaction mixture was heated to reflux for 3 h, followed by addition of glacial acetic acid (10 mL) and water (30 mL) at that temperature. After 1 h, the reaction was cooled to room temperature and dichloromethane (50 mL) was added. The organic layer was collected the solvent removed *in vacuo* to obtain a brown residue. Upon recrystallization from cyclohexane, the product was isolated as a colorless solid (4.00 g, 16.6 mmol, 81%). **^{1}H NMR** (300 MHz, $CDCl_3$) δ = 7.32 – 7.26 (m, 4H), 6.91 – 6.84 (m, 4H), 5.31 (s, 2H), 3.84 (s, 6H). **^{13}C NMR** (75 MHz, $CDCl_3$) δ = 159.5, 149.2, 134.5, 129.6, 113.7, 111.8, 55.4. The spectroscopic data are in accordance with those described in the literature.[4]

2.2.2 1-Methoxy-2-(prop-1-en-2-yl)benzene

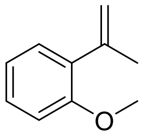

In a 250 mL round-bottom flask, methyltriphenylphosphonium bromide (4.3 g, 12.0 mmol, 1.5 equiv.) was suspended in THF (20 mL) and cooled to 0 °C. *n*-BuLi (1.6 M, hexanes, 6.5 mL, 10 mmol, 1.3 equiv.) was added dropwise and the reaction mixture was stirred for 1 h at room temperature followed by the addition of 1-(3,4-dimethylphenyl)ethan-1-one (1.2 g, 8.0 mmol, 1.0 equiv). The reaction mixture was refluxed for 4 h followed by addition of Et_2O (100 mL) and pentane (100 mL) resulting in the precipitatation of triphenylphosphine oxide. The thus formed suspension was filtered, the filter cake washed with additional Et_2O (100 mL) and the filtrate collected. After removal of the solvent *in vacuo*, the residue was purified *via* column chromatography (pentane) to afford the product as a colorless oil (0.54 g, 3.6 mmol, 45%). **^{1}H NMR** (300 MHz, $CDCl_3$) δ = 7.33 – 7.21 (m, 2H), 7.00 – 6.90 (m, 2H), 5.22 – 5.17 (m, 1H), 5.13 – 5.09 (m, 1H), 3.88 (s, 3H), 2.19 – 2.14 (m, 3H).**^{13}C NMR** (75 MHz, $CDCl_3$) δ = 156.8, 144.5, 133.0, 129.5, 128.4, 120.7, 115.2, 111.0, 55.6, 23.3. The spectroscopic data are in accordance with those described in the literature.[5]

2.2.3 5-(Pent-1-en-2-yl)benzo[d][1,3]dioxole

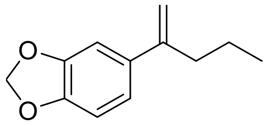

In a 500 mL round-bottom flask, methyltriphenylphosphonium bromide (10.7 g, 30.0 mmol, 1.20 equiv.) was suspended in THF (125 mL) and cooled to 0 °C. *n*-BuLi (1.6 M, hexanes, 30 mmol, 19 mL, 1.2 equiv.) was added dropwise and the reaction mixture was stirred for 1 h at room temperature followed by the addition of 1-(benzo[d][1,3]dioxol-5-yl)butan-1-one (4.8 g, 25 mmol, 1.0 equiv). The reaction mixture was refluxed for 4 h followed by addition of Et_2O (100 mL) and pentane (100 mL) resulting in the precipitatation of triphenylphosphine oxide. The thus formed suspension was filtered, the filter cake washed with additional Et_2O (100 mL) and the filtrate collected. After removal of the solvent *in vacuo*, the residue was purified *via* bulb-to-bulb distillation to afford the product as a colorless oil (2.4 g, 13 mmol, 50%). **^{1}H NMR** (300 MHz, $CDCl_3$) δ = 6.95 – 6.85 (m, 2H), 6.77 (dd, *J*=7.9 Hz, 0.6 Hz, 1H), 5.95 (s, 2H), 5.18 (d, *J*=1.5 Hz, 1H), 4.98 (d, *J*=1.5 Hz, 1H), 2.49 – 2.37 (m, 2H), 1.58 – 1.37 (m, 2H), 0.92 (t, *J*=7.3 Hz, 3H). **^{13}C NMR** (75 MHz, $CDCl_3$) δ = 148.1, 147.8, 147.0, 136.0, 119.7, 111.4, 108.1, 106.9, 101.1, 37.8, 21.5, 13.9. The spectroscopic data are in accordance with those described in the literature.[6]

2.2.4 1,2-Dimethyl-4-(prop-1-en-2-yl)benzene

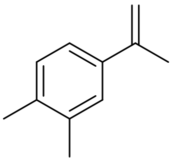

In a 250 mL round-bottom flask, methyltriphenylphosphonium bromide (4.3 g, 12 mmol, 1.5 equiv.) was suspended in THF (20 mL) and cooled to 0 °C. *n*-BuLi (1.6 M, hexanes, 6.5 mL, 10 mmol, 1.3 equiv.) was added dropwise and the reaction mixture was stirred for 1 h at room temperature followed by the addition of 1-(3,4-dimethylphenyl)ethan-1-one (1.2 g, 8.0 mmol, 1.0 equiv). The reaction mixture was refluxed for 4 h followed by addition of Et_2O (100 mL) and pentane (100 mL) resulting in the precipitatation of triphenylphosphine oxide. The thus formed suspension was filtered, the filter cake washed with additional Et_2O (100 mL) and the filtrate collected. After removal of the solvent *in vacuo*, the residue was purified *via* column chromatography (pentane) to afford the product as a colorless oil (0.86 g, 5.8 mmol, 73%). **^{1}H NMR** (300 MHz, $CDCl_3$) δ = 7.30 – 7.18 (m, 2H), 7.11 (d, *J*=7.8 Hz, 1H), 5.34 (dd, *J*=1.7 Hz, 0.9 Hz, 1H), 5.06 – 5.02 (m, 1H), 2.29 (s, 3H), 2.28 (s,3H), 2.18 – 2.13 (m, 3H). **^{13}C NMR** (75 MHz, $CDCl_3$) δ = 143.3, 139.0, 136.2, 135.8, 129.5, 126.8, 123.0, 111.4, 21.9, 19.9, 19.4. The spectroscopic data are in accordance with those described in the literature.[7]

2.2.5 (*R*)-2-(6-Bromopyridin-2-yl)-4-isopropyl-4,5-dihydrooxazole

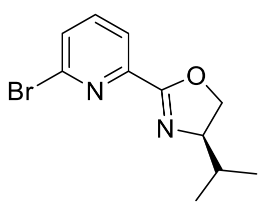

In analogy to a procedure by Lu and coworkers,[6] 2-bromo-5-cyanopyridine (3.0 g, 16 mmol, 1.0 equiv.) was dissolved in anhydrous toluene (70 mL) under an inert atmosphere followed by the addition of zink triflate (6.6 g, 18 mmol, 1.1 equiv.) and (*S*)-valinol (1.7 g, 16 mmol, 1.0 equiv.). The reaction mixture was stirred at 110 °C for 3 days. Ethyl acetate (20 mL) was added and the resulting solution washed with brine (2x30 mL) and saturated sodium bicarbonate solution (3x30 mL). The organic layers were collected, dried over $MgSO_4$ and the solvent was evaporated under vacuum yielding crude product which was further purified *via* column chromatography to yield the pyridine derivative as a colorless solid (2.0 g, 7.5 mmol, 45%). **^{1}H NMR** (300 MHz, $CDCl_3$) δ = 8.04 (dd, *J*=7.1 Hz, 1.6 Hz, 1H), 7.66 – 7.54 (m, 2H), 4.50 (dd, *J*=9.4 Hz, 8.0 Hz, 1H), 4.25 – 4.08 (m, 2H), 1.88 (hept, *J*=6.8 Hz, 1H), 1.03 (d, *J*=6.8 Hz, 3H), 0.92 (d, *J*=6.8 Hz, 3H). **^{13}C NMR** (75 MHz, $CDCl_3$) δ = 161.5, 147.9, 142.1, 138.8, 130.3, 123.0, 73.1, 71.1, 32.9, 19.1, 18.3. The spectroscopic data are in accordance with those described in the literature.[7]

2.2.6 (*R*)-1-(6-(4-Isopropyl-4,5-dihydrooxazol-2-yl)pyridin-2-yl)ethan-1-one

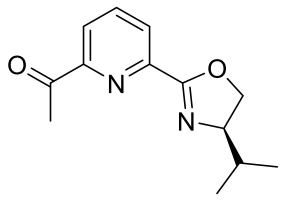

In analogy to a procedure by Lu and coworkers,[6] (*R*)-2-(6-bromopyridin-2-yl)-4-isopropyl-4,5-dihydrooxazole (0.60 g, 2.2 mmol, 1.0 equiv.) was dissolved in anhydrous ether (20 mL) under an inert atmosphere and cooled to -78 °C. Subsequently *n*-BuLi (1.6 M, hexanes, 2.2 mmol, 1.4 mL, 1.0 equiv.) was diluted with Et_2O (10 mL) and the thus formed solution added dropwise to the reaction mixture over the course of 20 min. After 1 h of stirring dimethylacetamide (0.45 mL, 4.4 mmol, 2.0 equiv.) was added at that temperature and stirring was continued for 2 h. The resulting solution was warmed to room temperature and water (20 mL) was added. Et_2O (20 mL) was added and the resulting solution was washed with saturated sodium chloride solution (2x30 mL) and saturated sodium bicarbonate solution (3x30 mL). The organic layers were collected, dried over $MgSO_4$ and the solvent was evaporated under vacuum. FC of the crude product yielded the pyridine derivative as a colorless solid (0.17 g, 0.73 mmol, 33%). **^{1}H NMR** (300 MHz, $CDCl_3$) δ = 8.25 (dd, *J*=7.8 Hz, 1.2 Hz, 1H), 8.11 (dd, *J*=7.8 Hz, 1.2 Hz, 1H), 7.90 (dd, *J*=*J*=7.8 Hz, 1H), 4.55 (dd, *J*=9.4 Hz, 8.0 Hz, 1H), 4.29 – 4.13 (m, 2H), 2.79 (s, 3H), 1.90 (hept, *J*=6.8 Hz, 1H), 1.07 (d, *J*=6.8 Hz, 3H), 0.96 (d, *J*=6.8 Hz, 3H). **^{13}C NMR** (75 MHz, $CDCl_3$) δ = 199.9, 162.4, 153.7, 146.7, 137.6, 127.6, 123.4, 73.2, 71.2, 33.0, 25.9, 19.2, 18.4. The spectroscopic data are in accordance with those described in the literature.[6]

2.2.7 (*R*,*E*)-*N*-(2,6-Diisopropylphenyl)-1-(6-(4-isopropyl-4,5-dihydrooxazol-2-yl)pyridin-2-yl)ethan-1-imine

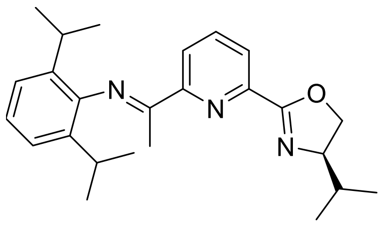

In analogy to a procedure by Lu and coworkers,[6] (*R*)-1-(6-(4-isopropyl-4,5-dihydrooxazol-2-yl)pyridin-2-yl)ethan-1-one (0.16 g, 0.68 mmol, 1.0 equiv.) was dissolved in anhydrous methanol followed by the addition of 2,6-diisopropylaniline (0.14 g, 0.95 mmol, 1.2 equiv.) and formic acid (7 µL, 9 µmol, 1 mol%). The reaction mixture was refluxed for 24 h and afterwards the solvent removed carefully *in vacuo*. Purification by column chromatography gave the product as a pale yellow solid (150 mg, 0.38 mmol, 56%). **^{1}H NMR** (300 MHz, CD_2Cl_2) δ = 8.46 (dd, *J*=7.9 Hz, 1.2 Hz, 1H), 8.16 (dd, *J*=7.9 Hz, 1.2 Hz, 1H), 7.90 (dd, *J*=*J*=7.9 Hz, 1H), 7.20 – 7.12 (m, 2H), 7.08 (dd, *J*=8.7 Hz, 6.4 Hzi, 1H), 4.58 – 4.46 (m, 1H), 4.26 – 4.10 (m, 2H), 2.73 (hept, 6.8 Hz, 2H), 2.22 (s, 3H), 1.88 (hept, *J*=6.8 Hz, 1H), 1.18 – 1.10 (m, 13H), 1.06 (d, *J*=6.8 Hz, 3H), 0.96 (d, *J*=6.8 Hz, 3H). **^{13}C NMR** (75 MHz, CD_2Cl_2) δ = 167.4, 163.0, 156.8, 147.0, 146.9, 137.5, 136.3, 125.9, 124.2, 123.6, 123.5, 73.7, 71.5, 33.6, 28.9, 23.6, 23.1, 19.4, 18.7, 17.5. The spectroscopic data are in accordance with those described in the literature.[6]

2.2.8 Catalyst for asymmetric hydroboration (C1)

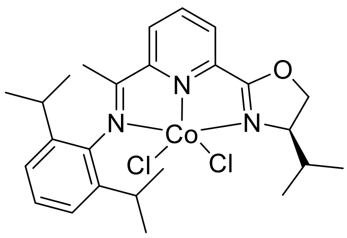

In analogy to a procedure by Lu and coworkers,[6] $CoCl_2$ (15.7 mg, 0.12 mmol, 0.95 equiv.) was dissolved in THF (1.3 mL) and (*R*,*E*)-N-(2,6-Diisopropylphenyl)-1-(6-(4-isopropyl-4,5-dihydrooxazol-2-yl)pyridin-2-yl)ethan-1-imine (50 mg, 0.13 mmol, 1.0 equiv.) was added to the reaction mixture. The solution is stirred for 4 h followed by the addition of Et_2O (1 mL) to precipitate the product as a green solid which is filtered and washed with Et_2O (1 mL) (50 mg, 0.09 mmol, 73%). **HRMS** (ESI) *m/z*: 485.1639 calcd. for $C_{25}H_{33}ClCoN_3O^+$ $[M-Cl]^+$, found: 485.1639.

2.2.9 3-Phenylpropyl diisopropylcarbamate (CARB1)

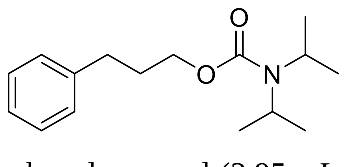

In analogy to a procedure of *Aggarwal* and coworkers,[8] triethylamine (5.00 mL, 36.1 mmol, 1.2 equiv) was added to a solution of *N*,*N*-diisopropylcarbamyl chloride (5.40 g, 33.1 mmol, 1.1 equiv.) and 3-phenylpropanol (3.95 mL, 30.1 mmol, 1.0 equiv.) in CH_2Cl_2 (30 mL) at room temperature. The reaction was sealed and refluxed for 16 h. After cooling to room temperature, aqueous NaOH solution (1 M) was added, the layers were separated and the aqueous phase was extracted with CH_2Cl_2 (3x15 mL). The combined organic layers were washed with aqueous HCl (1 N, 15 mL), saturated aqueous sodium

chloride solution (15 mL) and dried over $MgSO_4$. Removal of the solvents *in vacuo* and flash column chromatography (P:Et_2O = 10:1) afforded the compound (7.33 g, 27.8 mmol, 92%) as a colorless oil. **^{1}H NMR** (300 MHz, $CDCl_3$, 300 K): δ = 7.38 – 7.30 (m, 2H), 7.29 – 7.21 (m, 3H), 4.18 (t, *J*=6.5 Hz, 2H), 3.95 (br, 2H), 2.84 – 2.72 (m, 2H), 2.11 – 1.97 (m, 2H), 1.28 (d, *J*=6.8 Hz, 12H). **^{13}C NMR** (75 MHz, 300 K, $CDCl_3$): δ = 155.9, 141.7, 128.5, 128.5, 126.0, 64.2, 45.8, 32.7, 31.1, 21.2. **HRMS** (ESI) *m/z*: 286.1778 calcd. for $C_{16}H_{25}NO_2Na$ $[M+Na]^+$, found: 286.1785. Spectroscopic data are in accordance with those described in the literature.[9]

2.3 Synthesis of Boronic Esters

2.3.1 2-(3-(3,4-Dimethoxyphenyl)propyl)-4,4,5,5-tetramethyl-1,3,2-dioxaborolane (1c)

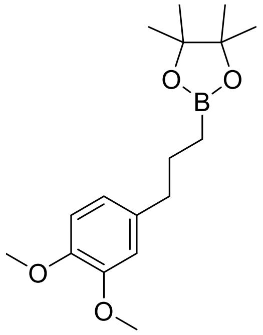

The reaction was performed according to general procedure **GP1** with 4-allyl-1,2-dimethoxybenzene (1.78 g, 10.0 mmol, 1.00 equiv.), boron trichloride (1.0 M, dichloromethane, 10.50 mL, 10.50 mmol, 1.05 equiv.), dichloromethane (10 mL), triethylsilane (1.49 mL, 10.0 mmol, 1.00 equiv.) and pinacol (1.18 g, 10.0 mmol, 1.00 equiv.). After bulb-to-bulb distillation the boronic ester was obtained as a colorless oil (830 mg, 2.21 mmol, 27%). **FTIR** (neat): υ (cm^{-1}) 2976, 2937, 2835, 2361, 2021, 1591, 1515, 1465, 1416, 1371, 1318, 1261, 1234, 1143, 1108, 1031, 968, 873, 847, 805, 764, 731, 692, 674, 632. **^{1}H NMR** (300 MHz, $CDCl_3$) δ = 6.88 – 6.59 (m, 3H), 3.85 (s, 3H), 3.83 (s, 3H), 2.54 (t, J=7.5 Hz, 2H), 1.77 – 1.64 (m, 2H), 1.23 (s, 12H), 0.81 (t, J=7.9 Hz, 2H). **^{13}C NMR** (75 MHz, $CDCl_3$) δ = 148.9, 147.2, 135.6, 120.5, 112.2, 111.4, 83.0, 77.4, 56.1, 55.9, 38.3, 26.3, 24.9. The signal of the α-B-carbon was not observed. **HRMS** (ESI): m/z = 329.1895 calcd. for $C_{17}H_{27}BO_4Na^+$ $[M+Na]^+$, found: 329.1908.

2.3.2 2-(2-([1,1'-Biphenyl]-4-yl)propyl)-4,4,5,5-tetramethyl-1,3,2-dioxaborolane (1d, 14c)

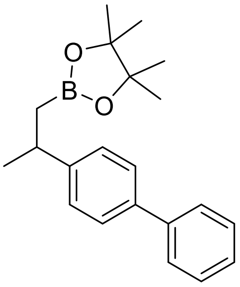

The reaction was performed according to general procedure **GP1** with 4-(prop-1-en-2-yl)-1,1'-biphenyl (480 mg, 2.47 mmol, 1.00 equiv.), boron trichloride (1.0 M, dichloromethane, 2.71 mL, 2.71 mmol, 1.10 equiv.), dichloromethane (5 mL), triethylsilane (400 µl, 2.47 mmol, 1.00 equiv.) and pinacol (584 mg, 4.94 mmol, 2.00 equiv.). After bulb-to-bulb distillation the boronic ester was obtained as a colorless oil (400 mg, 1.24 mmol, 50%). **^{1}H NMR** (300 MHz, $CDCl_3$) δ = 7.64 –

7.57 (m, 2H), 7.55 – 7.50 (m, 2H), 7.47 – 7.40 (m, 2H), 7.37 – 7.30 (m, 3H), 3.19 – 3.04 (m, 1H), 1.34 (d, *J*=6.9, 3H), 1.26 – 1.14 (m, 14H). **^{13}C NMR** (75 MHz, $CDCl_3$) δ 148.6, 141.4, 138.8, 128.8, 127.2, 127.1, 127.1, 127.0, 83.2, 35.6, 25.0, 24.9, 24.8, 21.4. The spectroscopic data are in accordance with those described in the literature [13]

2.3.3 2-(2-(4-(*tert*-Butyl)phenyl)propyl)-4,4,5,5-tetramethyl-1,3,2-dioxaborolan (1e)

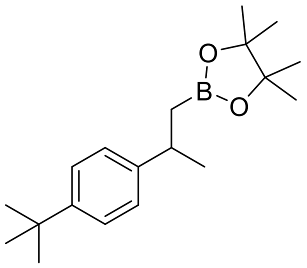

The reaction was performed according to **GP1** with 1-(*tert*-butyl)-4-(prop-1-en-2-yl)benzene (700 mg, 4.02 mmol, 1.0 equiv.) triethylsilane (639 µL, 4.02 mmol, 1.0 equiv.), boron trichloride (1.0 M, dichloromethane, 4.4 mL, 4.4 mmol, 1.1 equiv.) and pinacol (615 mg, 5.23 mmol, 1.3 equiv.) in CH_2Cl_2 (8.0 mL). bulb-to-bulb distillation (0.5 mbar, 150 °C) afforded the desired boronic ester **1d** (753 mg, 2.49 mmol, 62%) as a colorless oil. **^{1}H NMR** (300 MHz, $CDCl_3$): δ = 7.31 – 7.25 (m, 2H), 7.20 – 7.14 (m, 2H), 3.08 – 2.94 (m, 1H), 1.32 – 1.26 (m, 12H), 1.18 – 1.12 (m, 14H). **^{13}C NMR** (75 MHz, 300 K, $CDCl_3$):δ = 148.5, 146.3, 126.4, 125.1, 83.1, 35.4, 34.4, 31.6, 24.9, 24.9, 24.8. The signal of the α-B-carbon was not observed. **^{11}B NMR** (96 MHz, $CDCl_3$):δ = 33.2. **HRMS** (ESI) m/z: 325.2309 calcd. for $C_{19}H_{31}BO_2Na$ $[M+Na]^+$, found: 325.2312.

2.3.4 2-(2,2-Diphenylethyl)-4,4,5,5-tetramethyl-1,3,2-dioxaborolane (1f)

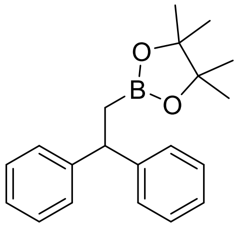

The reaction was performed according to general procedure **GP1** with 1,1-diphenylethene (2.03 g, 11.0 mmol, 1.0 equiv.), boron trichloride (1.0 M, hexanes, 11.0 mL, 2.10 mmol, 1.0 equiv.), *n*-hexane (11 mL), triethylsilane (1.73 mL, 11.0 mmol, 1.0 equiv.) and pinacol (1.30 g, 11.0 mmol, 1.0 equiv). After bulb-to-bulb distillation the boronic ester was obtained as a colorless oil (2.20 g, 7.20 mmol, 65%). **^{1}H NMR** (300 MHz, $CDCl_3$) δ = 7.28 – 7.21 (m, 8H), 7.18 – 7.09 (m, 2H), 4.24 (t, *J*=8.5 Hz, 1H), 1.55 (d, *J*=8.5 Hz, 2H), 1.04 (s, 12H). **^{13}C NMR** (75 MHz, $CDCl_3$) δ 146.8, 128.3, 127.8, 126.0, 83.2, 46.7, 24.7. The signal of the α-B-carbon was not observed. **HRMS** (ESI): m/z = 331.1840 calcd. for $C_{20}H_{25}BO_2Na^+$ $[M+Na]^+$, found: 331.1855. The spectroscopic data are in accordance with those described in the literature.[15]

2.3.5 2-(2,2-Bis(4-methoxyphenyl)ethyl)-4,4,5,5-tetramethyl-1,3,2-dioxaborolane (1g)

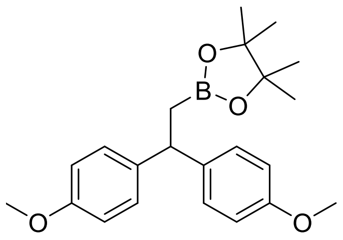

The reaction was performed according to general procedure **GP1** with 1,1-bis(4-methoxyphenyl)ethene (1.50 g, 6.25 mmol, 1.00 equiv.), boron trichloride (1.0 M, dichloromethane, 6.80 mL, 6.80 mmol, 1.10 equiv.), methylene chloride (10 mL), triethylsilane (1.00 mL, 6.25 mmol, 1.00 equiv.) and pinacol (738 mg, 6.25 mmol, 1.00 equiv). After bulb-to-bulb distillation the boronic ester was obtained as a colorless oil (1.50 g, 4.08 mmol, 65%). **FTIR** (neat): υ (cm^{-1}) 2977, 2936, 2834, 2364, 2046, 1982, 1608, 1590, 1512, 1465, 1417, 1371, 132, 1261, 1240, 1154, 1108, 1032, 968, 892, 847, 829, 805, 765, 675, 576, 555. **^{1}H NMR** (300 MHz, $CDCl_3$) δ = 7.21 – 7.11 (m, 4H), 6.86 – 6.75 (m, 4H), 4.19 (t, *J*=8.5 Hz, 1H), 3.75 (s, 6H), 1.63 – 1.49 (m, 2H), 1.07 (s, 12H). **^{13}C NMR** (75 MHz, $CDCl_3$) δ = 157.7, 139.3, 128.5, 113.6, 83.1, 55.2, 44.9, 24.6. The signal of the α-B-carbon was not observed. **HRMS** (ESI): m/z = 391.2051 calcd. for $C_{22}H_{29}BO_4Na^+$ $[M+Na]^+$, found: 391.2070.

2.3.6 4,4,5,5-Tetramethyl-2-(2-(naphthalen-2-yl)ethyl)-1,3,2-dioxaborolane (1h)

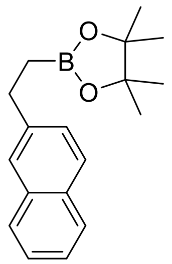

The reaction was performed according to general procedure **GP1** with 2-vinylnaphthalene (1.54 g, 10.0 mmol, 1.0 equiv.), boron trichloride (1.0 M, dichloromethane, 10.5 mL, 10.5 mmol, 1.05 equiv.), dichloromethane (10 mL), triethylsilane (1.49 mL, 10.0 mmol, 1.0 equiv.) and pinacol (1.18 g, 10.0 mmol, 1.00 equiv.). After bulb-to-bulb distillation, the boronic ester was obtained as a colorless solid (1.2 g, 4.21 mmol, 42%). **^{1}H NMR** (300 MHz, $CDCl_3$) δ = 7.85 – 7.74 (m, 3H), 7.68 (bs, 1H), 7.51 – 7.37 (m, 3H), 2.96 (t, *J*=8.1 Hz, 2H), 1.35 – 1.19 (m, 14H). **^{13}C NMR** (75 MHz, $CDCl_3$) δ 142.1, 133.8, 132.1, 127.8, 127.7, 127.6, 127.4, 125.8, 125.0, 83.6, 30.3, 25.0. The signal of the α-B-carbon was not observed. The spectroscopic data are in accordance with those described in the literature.[14]

2.3.7 Triphenyl(3-(4,4,5,5-tetramethyl-1,3,2-dioxaborolan-2-yl)propyl)silane (1i, 14g)

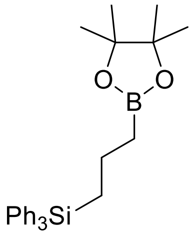

The reaction was performed according to general procedure **GP1** with triphenylallylsilane (1.50 g, 5.00 mmol, 1.0 equiv.), boron trichloride (1.0 M, dichloromethane, 5.50 mL, 5.50 mmol, 1.1 equiv.), dichloromethane (10 mL), triethylsilane (0,750 mL, 5.00 mmol, 1.00 equiv.) and pinacol (1.18 g, 10.0 mmol, 2.00 equiv.). After bulb-to-bulb distillation, the boronic ester was obtained as a colorless solid (1.01 g, 2.36 mmol, 47%). **^{1}H NMR** (300 MHz, $CDCl_3$) δ = 7.64 – 7.49 (m, 6H), 7.45 – 7.30 (m,

9H), 1.69 – 1.55 (m, 2H), 1.47 – 1.35 (m, 2H), 1.24 (s, 12H), 0.91 (t, *J*=7.2 Hz, 2H). **^{13}C NMR** (75 MHz, $CDCl_3$) δ 135.8, 135.6, 129.4, 127.9, 83.0, 25.0, 18.8, 16.5. The signal of the α-B-carbon was not observed. The spectroscopic data are in accordance with those described in the literature[10]

2.3.8 2-(2-(Benzo[d][1,3]dioxol-5-yl)pentyl)-4,4,5,5-tetramethyl-1,3,2-dioxaborolane (1k)

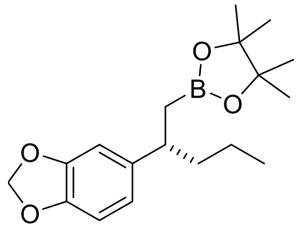

In analogy to a procedure of Lu and coworkers,[6] freshly distilled pinacolborane (187 µL, 1.25 mmol, 1.00 equiv.), 5-(pent-1-en-2-yl)benzo[d][1,3]dioxole (243 mg, 1.25 mmol, 1.00 equiv.) and chiral catalyst **C1** (34 mg, 0.070 mmol, 5 mol%) were added to a Schlenk tube containing a stir bar under inert atmosphere. After cooling the solution to -20 °C, sodium triethylborohydride (1 M, THF, 187 µL, 187 mmol, 0.15 equiv.) was added dropwise and the solution was again warmed to room temperature while stirring. After 1 h the crude reaction mixture purified *via* column chromatography to obtain the pure boronic ester (396 mg, 1.24 mmol, 98%). **^{1}H NMR** (300 MHz, $CDCl_3$) δ = 6.73 – 6.61 (m, 3H), 5.89 (s, 2H), 2.84 – 2.68 (m, 1H), 1.56 – 1.46 (m, 2H), 1.07-1.20 (m, 16H), 0.83 (t, *J*=7.3 Hz, 3H). **^{13}C NMR** (75 MHz, $CDCl_3$) δ 147.5, 145.5, 141.9, 120.5, 107.9, 107.8, 100.7, 83.1, 42.0, 41.2, 24.9, 24.9, 20.9, 14.2. The signal of the α-B-carbon was not observed. The spectroscopic data are in accordance with those described in the literature[6] The racemate was obtained according to **GP2** using 5-(pent-1-en-2-yl)benzo[d][1,3]dioxole. Enantiomeric ratio (er = 98:2) was determined by chiral HPLC analysis.

2.3.9 4,4,5,5-Tetramethyl-2-(1-(phenylsulfonyl)ethyl)-1,3,2-dioxaborolane (1l)

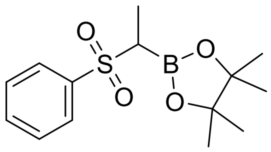

The reaction was performed according to general procedure **GP1** with phenyl vinyl sulfone (336 mg, 2.00 mmol, 1.00 equiv.), boron trichloride (1.0 M, dichloromethane, 2.10 mL, 2.10 mmol, 1.05 equiv.), dichloromethane (10 mL), triethylsilane (320 µl, 2.00 mmol, 1.00 equiv.) and pinacol (354 mg, 3.00 mmol, 1.50 equiv). After bulb-to-bulb distillation, the boronic ester was obtained as a colorless oil (463 mg, 1.56 mmol, 78%). **FTIR** (neat): υ (cm^{-1}) 2980, 2365, 2335, 2194, 2146, 1978, 1473, 1447, 1339, 1306, 1217, 1175, 1086, 982, 841, 782, 767, 732, 692, 674, 586, 561. **^{1}H NMR** (300 MHz, $CDCl_3$) δ = 7.88 – 7.80 (m, 2H), 7.58 – 7.51 (m, 1H), 7.49 – 7.41 (m, 2H), 2.95 (q, *J*=7.3 Hz, 1H), 1.25 (d, *J*=7.3 H, 3H), 1.16 (s, 12H). **^{13}C NMR** (75 MHz, $CDCl_3$) δ 139.5, 133.3, 128.8, 128.7, 84.9, 24.6, 10.7. The signal of the α-B-carbon was not observed. **HRMS** (ESI): m/z = 319.1146 calcd. for $C_{14}H_{21}BO_4SNa^+$ $[M+Na]^+$, found: 319.1150.

2.3.10 *tert*-Butyl (*R*)-2-(4,4,5,5-tetramethyl-1,3,2-dioxaborolan-2-yl)pyrrolidine-1-carboxylate (1m)

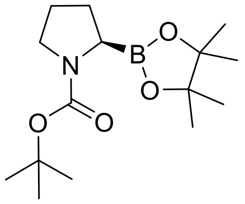

In analogy to a procedure of AGGARWAL and coworkers,[17] (+)-sparteine (1.7 mL, 7.2 mmol, 1.2 equiv.) and *N*-Boc-pyrrolidine (1.1 mL, 6.0 mmol, 1.0 equiv.) were dissolved in Et_2O (60 mL). The solution was cooled to -78 °C and *sec*-BuLi (1.4 M, cyclohexane, 5.1 mL, 7.2 mmol, 1.2 equiv.) was added dropwise. The mixture was stirred for 3 h at that temperature before 2-isopropoxy-4,4,5,5-tetramethyl-1,3,2-dioxaborolane (1.6 mL, 7.8 mmol, 1.3 equiv.) was added dropwise. After stirring at -78 °C for 2 h, the mixture was allowed to warm to room temperature. Subsequently, aqueous HCl-solution (1.0 M, 50 mL) was added and the layers were separated. The organic layer was washed with aqueous saturated NaCl-solution and the combined aqueous layers were extracted with Et_2O (3x50 mL). The combined organic layers were dried over $MgSO_4$ and the solvent was removed *in vacuo*. FC (P:Et_2O = 6:1) afforded the desired boronic ester **1m** (1.5 g, 5.1 mmol, 84%) as a colorless solid. $[\alpha]_D^{25}$ = -58.5 (*c* 1.0 CH_2Cl_2) **^{1}H NMR** (500 MHz, DMSO-d6, 363 K):δ = 3.32 – 3.25 (m, 1H), 3.23 – 3.16 (m, 1H), 2.97 (s, 1H), 1.99 – 1.59 (m, 4H), 1.40 (s, 9H), 1.23 – 1.18 (m, 12H). **^{13}C NMR** (126 MHz, DMSO-*d*6, 363 K):δ = = 153.4, 82.8, 77.6, 45.5, 42.7, 27.8; 27.1, 25.2, 24.2, 23.9. The signal of the α-B-carbon was not observed. **^{11}B NMR** (96 MHz, DMSO-*d*6, 363 K): δ = 31.8. **HRMS** (ESI) m/z: 320.2004 calcd. for $C_{15}H_{28}BNO_4Na$ $[M+Na]^+$, found: 320.2019. Spectroscopic data are in accordance with those described in the literature.[17]

2.3.11 *tert*-Butyl 4-methyl-4-(4,4,5,5-tetramethyl-1,3,2-dioxaborolan-2-yl)octanoate (1n)

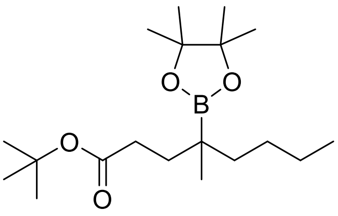

Isopropenylboronic acid pinacol ester (90%, 0.62 mL, 3.0 mmol, 1.0 equiv.) was dissolved Et_2O (10 mL) and *n*-BuLi (1.6 M, hexane, 2.1 mL, 3.3 mmol, 1.1 equiv.) was added dropwise over 5 min at 0 °C. The mixture was allowed to warm up to room temperature and stirred at that temperature for another 30 minutes. Subsequently, the solvent was carefully removed *in vacuo* and the resulting residue was taken up in MeCN (10 mL). After the addition of *tert*-butyl-2-iodoacetate (1.5 g, 6.0 mmol, 2.0 equiv) the tube was sealed and the mixture was stirred under irradiation by a 400 W lamp (temperature up to 50 °C) for 24 h. Removal of the solvent *in vacuo* and FC (P:Et_2O = 50:1→10:1) afforded the desired boronic ester **1n** (990 mg, 2.91 mmol, 97%) as a colorless oil. **IR (neat)**: ν (cm^{-1}) 2978, 2969, 2931, 1730, 1469, 1389, 1368, 1309, 1250, 1216, 1138, 967, 856, 692, 670.**^{1}H NMR (**300 MHz, $CDCl_3$, 299 K): δ = 2.24 – 2.09 (m, 2H), 1.74 – 1.62 (m, 1H), 1.53 – 1.40 (m, 10H), 1.35 – 1.14 (m, 18H), 0.91 – 0.83 (m, 6H).**^{13}C NMR (**75 MHz, $CDCl_3$, 299 K) δ = 174.0, 83.1, 79.8, 38.7, 33.8, 32.2, 28.3, 28.2, 24.9, 24.9, 23.7, 21.3, 14.2. The signal of the α-B-carbon was not observed. **^{11}B NMR**

(96 MHz, $CDCl_3$, 299 K) δ = 34.5. **HRMS** (ESI) *m/z* = 363.2677calcd. for $C_{19}H_{37}BO_4Na$ $[M+Na]^+$, found: 363.2700.

2.3.12 2-(1-Cyclopropyl-3-phenylpropyl)-4,4,5,5-tetramethyl-1,3,2-dioxaborolan (1o)

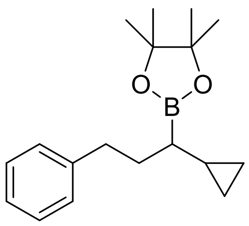

In analogy to a procedure of *Aggarwal* and coworkers,[8] *sec*-BuLi (1.4 M, cyclohexane, 2.4 mL, 3.3 mmol, 1.1 equiv.) was added dropwise to a solution of carbamate **CARB1** (948 mg, 3.60 mmol, 1.2 equiv.) and tetramethylethylenediamine (0.54 mL, 3.6 mmol, 1.2 equiv.) in Et_2O (25 mL) at -78 °C and the mixture was stirred at that temperature for 5 h. Subsequently, cyclopropyl boronic ester (0.55 mL, 3.0 mL. 1.0 equiv.) was added at -78 °C and the reaction was stirred at that temperature for 1 h. The mixture was allowed to warm to room temperature and was then refluxed for 12 h. After cooling down to room temperature, aqueous KH_2PO_4-solution (1.0 M, 15 mL) was added at 0 °C and the mixture was stirred for 10 min at that temperature. The aqueous layer was extracted with Et_2O (3x15 mL) and the combined organic layers were dried over $MgSO_4$. Removal of the solvent *in vacuo* and FC (P:Et_2O = 50:1) afforded the desired boronic ester **1o** (377 mg, 1.32 mmol, 44%) as a colorless oil. **^{1}H NMR (**300 MHz, $CDCl_3$, 300 K) δ = 7.31 – 7.13 (m, 5H), 2.66 (t, *J*=8.2 Hz, 2H), 1.94 – 1.73 (m, 2H), 1.28 (s, 12H), 0.78 – 0.65 (m, 1H), 0.52 – 0.35 (m, 3H), 0.18 – 0.01 (m, 2H). **^{13}C NMR (**75 MHz, 300 K, $CDCl_3$) δ = 143.3, 128.5, 128.3, 125.7, 83.1, 35.9, 33.8, 25.0, 24.9, 12.6, 5.4, 3.7. The signal of the α-B-carbon was not observed. **^{11}B NMR (**96 MHz, 300 K, $CDCl_3$) δ = 33.7. **HRMS** (ESI) m/z: 309.1996 calcd. for $C_{18}H_{27}BO_2Na$ $[M+Na]^+$, found: 309.1995. Spectroscopic data are in accordance with those described in the literature.[8]

2.3.13 2-(2-(3,4-Dimethylphenyl)propyl)-4,4,5,5-tetramethyl-1,3,2-dioxaborolane (14a)

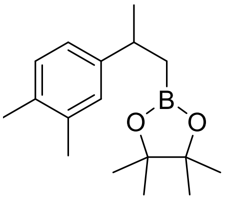

The reaction was performed according to general procedure **GP1** with 1,2-dimethyl-4-(prop-1-en-2-yl)benzene (0.86 g, 5.8 mmol, 1.0 equiv.), boron trichloride (1.0 M, dichloromethane, 6.4 mL, 6.4 mmol, 1.1 equiv.), dichloromethane (6 mL), triethylsilane (0.93 ml, 5.8 mmol, 1.00 equiv.) and pinacol (1.4 g, 12 mmol, 2.0 equiv.). After bulb-to-bulb distillation, the boronic ester was obtained as a colorless oil (1.2 g, 4.4 mmol, 76%). **FTIR** (neat): υ (cm^{-1}) 2976, 2924, 1505, 1453, 1363, 1318, 1271, 1214, 1198, 1144, 1110, 1021, 990, 969, 869, 847, 818, 717. **^{1}H NMR** (300 MHz, $CDCl_3$) δ = 7.07 – 6.95 (m, 3H), 3.07 – 2.91 (m, 1H), 2.24 (s, 3H), 2.22 (s, 3H), 1.27 (d, *J*=7.0 Hz, 3H), 1.22 – 1.03 (m, 14H). **^{13}C NMR** (75 MHz, $CDCl_3$) δ 147.0, 136.2, 133.7, 129.6, 128.2, 124.0, 83.1, 35.4, 25.0, 24.9, 19.9, 19.4. The signal of the α-

B-carbon was not observed. **HRMS** (ESI) m/z: 297.1996 calcd. for $C_{17}H_{27}BO_2Na$ $[M+Na]^+$, found: 297.2025.

2.3.14 2-(2-(2-Methoxyphenyl)propyl)-4,4,5,5-tetramethyl-1,3,2-dioxaborolane (14b)

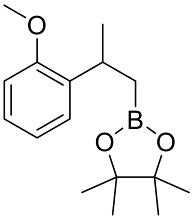

The reaction was performed according to general procedure **GP1** with 1-methoxy-2-(prop-1-en-2-yl)benzene (540 mg, 3.64 mmol, 1.0 equiv.), boron trichloride (1.0 M, dichloromethane, 4.0 mL, 4.0 mmol, 1.1 equiv.), dichloromethane (4 mL), triethylsilane (0.58 L, 3.7 mmol, 1.0 equiv.) and pinacol (0.86 g, 7.3 mmol, 2.0 equiv.). After bulb-to-bulb distillation the boronic ester was obtained as a colorless oil (800 mg, 2.90 mmol, 80%). **^{1}H NMR** (300 MHz, $CDCl_3$) δ = 7.22 (dd, *J*=7.4 Hz, 1.8, 1H), 7.13 (td, *J*=8.0 Hz, 1.8, 1H), 6.90 (td, *J*=7.4 Hz, 1.2 Hz, 1H), 6.82 (dd, *J*=8.0 Hz, 1.2 Hz, 1H), 3.82 (s, 3H), 3.54 – 3.38 (m, 1H), 1.24 (d, *J*=6.9 Hz, 3H), 1.20 – 1.14 (m, 14H). **^{13}C NMR** (75 MHz, $CDCl_3$) δ = 156.93, 137.63, 126.74, 126.54, 120.59, 110.54, 82.96, 55.47, 28.66, 24.92, 24.81, 23.53. The signal of the α-B-carbon was not observed. The spectroscopic data are in accordance with those described in the literature.[13]

2.3.15 2-(2,2-Bis(4-fluorophenyl)ethyl)-4,4,5,5-tetramethyl-1,3,2-dioxaborolane (14d)

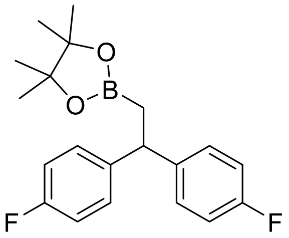

The reaction was performed according to general procedure **GP1** with 4,4'-(ethene-1,1-diyl)bis(fluorobenzene) (1.37 g, 6.30 mmol, 1.0 equiv.), boron trichloride (1.0 M, dichloromethane, 6.9 mL, 6.9 mmol, 1.1 equiv.), dichloromethane (10 mL), triethylsilane (1.0 mL, 6.3 mmol, 1.0 equiv.) and pinacol (1.48 mg, 12.6 mmol, 2.0 equiv.). After bulb-to-bulb distillation, the boronic ester was obtained as a colorless oil (1.70 g, 4.90 mmol, 78%). **FTIR** (neat): υ (cm^{-1}) 2978, 2931, 1603, 1506, 1365, 1157, 1143, 1090, 1015, 967, 889, 847, 831, 785, 744, 675. **^{1}H NMR** (300 MHz, $CDCl_3$) δ = 7.24 – 7.15 (m, 4H), 6.99 – 6.90 (m, 4H), 4.25 (t, *J*=8.4 Hz, 1H), 1.54 (d, *J*=8.4 Hz, 2H), 1.07 (s, 12H). **^{13}C NMR** (75 MHz, $CDCl_3$) δ **^{13}C NMR** (75 MHz, $CDCl_3$) δ 161.4 (d, *J*=244.1 Hz), 142.3 (d, *J*=3.3 Hz), 129.1 (d, *J*=7.8 Hz), 115.1 (d, *J*=21.1 Hz), 83.4, 45.2, 24.7. The signal of the α-B-carbon was not observed. **HRMS** (ESI) m/z: 367.1651 calcd. for $C_{20}H_{23}BF_2O_2Na^+$ $[M+Na]^+$, found: 367.1658.

2.3.16 2-(2-(Benzothiophen-2-yl)propyl)-4,4,5,5-tetramethyl-1,3,2-dioxaborolane (14e)

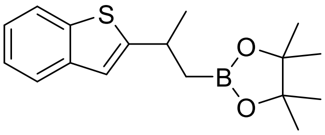

The reaction was performed according to general procedure **GP1** with 2-(prop-1-en-2-yl)benzothiophen (795 mg, 4.55 mmol, 1.0 equiv.), boron trichloride (1.0 M, dichloromethane, 5.0 mL, 5.0 mmol, 1.10 equiv.), dichloromethane (5 mL), triethylsilane (725 µl, 4.55 mmol, 1.00 equiv.) and pinacol (1.1 g, 9.0 mmol, 2.0 equiv.). After bulb-to-bulb distillation, the boronic ester was obtained as a colorless oil (1.2 g, 4.0 mmol, 88%). **^{1}H NMR** (300 MHz, $CDCl_3$) δ = 7.80 – 7.73 (m, 1H), 7.69 – 7.62 (m, 1H), 7.34 – 7.19 (m, 2H), 7.06 – 7.00 (m, 1H), 3.47 – 3.34 (m, 1H), 1.46 – 1.32 (m, 4H), 1.28 – 1.17 (m, 13H). **^{13}C NMR** (75 MHz, $CDCl_3$) δ = 154.9, 140.2, 139.1, 124.0, 123.4, 122.9, 122.3, 118.5, 83.4, 32.2, 25.2, 25.0, 24.9. The signal of the α-B-carbon was not observed. The spectroscopic data are in accordance with those described in the literature.[12]

2.3.17 2-(2-(Benzofuran-2-yl)propyl)-4,4,5,5-tetramethyl-1,3,2-dioxaborolane (14f)

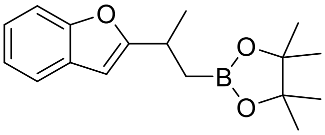

The reaction was performed according to general procedure **GP1** with 2-(prop-1-en-2-yl)benzofuran (645 mg, 4.10 mmol, 1.0 equiv.), boron trichloride (1.0 M, dichloromethane, 4.5 mL, 4.5 mmol, 1.10 equiv.), dichloromethane (5 mL), triethylsilane (653 µl, 4.10 mmol, 1.00 equiv.) and pinacol (0.97 g, 8.2 mmol, 2.00 equiv.). After bulb-to-bulb distillation, the boronic ester was obtained as a colorless oil (892 mg, 3.11 mmol, 76%). **^{1}H NMR** (300 MHz, $CDCl_3$) δ = 7.50 – 7.44 (m, 1H), 7.42 – 7.36 (m, 1H), 7.22 – 7.12 (m, 2H), 6.36 (t, *J*=0.8 Hz, 1H), 3.32 – 3.13 (m, 1H), 1.38 (d, *J*=6.9 Hz, 3H), 1.24 (m, 14H), 1.18 – 1.08 (m, 1H). **^{13}C NMR** (75 MHz, $CDCl_3$) δ 165.4, 154.7, 129.1, 123.1, 122.4, 120.4, 110.9, 100.0, 83.3, 29.8, 25.0, 24.9, 21.5. The signal of the α-B-carbon was not observed. The spectroscopic data are in accordance with those described in the literature.[11]

2.3.18 2-(2-(2-Methoxyphenyl)propyl)-4,4,5,5-tetramethyl-1,3,2-dioxaborolane (14h)

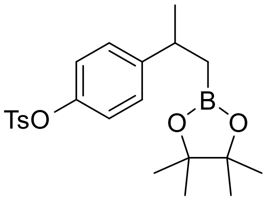

The reaction was performed according to general procedure **GP1** with 4-(prop-1-en-2-yl)phenyl 4-methylbenzenesulfonate (1.1 g, 3.7 mmol, 1.0 equiv.), boron trichloride (1.0 M, dichloromethane, 4.1 mL, 4.1 mmol, 1.1 equiv.), dichloromethane (4 mL), triethylsilane (0.59 mL, 3.7 mmol, 1.0 equiv.) and pinacol (0.88 g, 7.4 mmol, 2.0 equiv.). After bulb-to-bulb distillation the boronic ester was obtained as a colorless oil (0.66 g, 1.6 mmol, 43%). **FTIR** (neat): υ (cm^{-1}): 2976, 2930, 1598, 1502, 1365, 1322, 1270,

1197, 1176, 1142, 1093, 1017, 968, 863, 841, 813, 766, 733. **^{1}H NMR** (300 MHz, $CDCl_3$) δ = 7.73 – 7.67 (m, 2H), 7.29 (d, *J*=8.1 Hz, 2H), 7.16 – 7.10 (m, 2H), 6.92 – 6.83 (m, 2H), 3.06 – 2.93 (m, 1H), 2.44 (s, 3H), 1.22 (d, *J*=6.9 Hz, 3H), 1.14 (s, 6H), 1.13 (s, 6H), 1.09 (d, *J*=7.8 Hz, 2H). **^{13}C NMR** (75 MHz, $CDCl_3$) δ **^{13}C NMR** (75 MHz, $CDCl_3$) δ 148.3, 147.7, 145.2, 132.9, 129.8, 128.7, 127.9, 122.1, 83.2, 35.4, 25.0, 24.9, 24.9, 21.8. The signal of the α-B-carbon was not observed. **HRMS** (ESI) m/z: 439.1721 calcd. for $C_{22}H_{29}BSO_5Na^+$ $[M+Na]^+$, found: 439.1723

2.3.19 *rac*-4,4,5,5-Tetramethyl-2-((1*R*,2*R*)-2-phenylcyclohexyl)-1,3,2-dioxaborolane (14i)

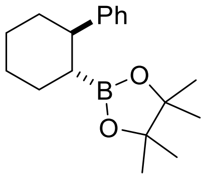

The reaction was performed according to general procedure **GP1** with 1-phenyl-1-cyclohexene (540 mg, 3.64 mmol, 1.0 equiv.), boron trichloride (1.0 M, dichloromethane, 6.6 mL, 6.6 mmol, 1.1 equiv.), dichloromethane (10 mL), triethylsilane (0.95 mL, 6.0 mmol, 1.0 equiv.) and pinacol (1.3 g, 12 mmol, 2.0 equiv.). After bulb-to-bulb distillation the boronic ester was obtained as a colorless oil (1.06 g, 3.70 mmol, 62%). **^{1}H NMR** (300 MHz, benzene-*d*$_6$) δ = 7.41 – 7.34 (m, 2H), 7.33 – 7.24 (m, 2H), 7.21 – 7.13 (m, 1H), 2.82 (td, *J*=11.4 Hz, 3.5 Hz, 1H), 2.10 – 2.00 (m, 1H), 2.01 – 1.89 (m, 1H), 1.88 – 1.77 (m, 1H), 1.77 – 1.22 (m, 5H), 0.97 (s, 6H), 0.95 (s, 6H). **^{13}C NMR** (75 MHz, benzene-*d*$_6$) δ = 148.2, 128.3, 128.1, 126.1, 82.6, 46.7, 36.7, 28.8, 27.5, 27.2, 24.7, 24.6. The signal of the α-B-carbon was not observed. The spectroscopic data are in accordance with those described in the literature.[16]

2.3.20 2-((6aR,8S,9S,10aR)-1-Methoxy-6,6,9-trimethyl-3-pentyl-6a,7,8,9,10,10a-hexahydro-6H-benzo[c]chromen-8-yl)-4,4,5,5-tetramethyl-1,3,2-dioxaborolane (14j)

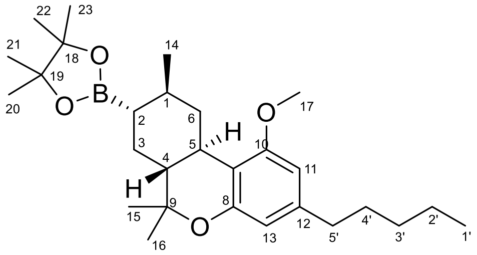

The reaction was performed according to general procedure **GP2** with catecholborane (1.20 mL, 11.9 mmol, 10.0 equiv.) and *O*-methyl-(−)-Δ^8-THC (0.390 mg, 1.19 mmol, 1.0 eq.). Purification by column chromatography on silica gel, eluting with pentane:EtOAc (98:2) gave the boronic ester as a colorless oil (292 mg, 0.630 mmol, 54% yield). **FTIR** (neat): υ (cm^{-1}) 2977, 2928, 2864, 1616, 1574, 1449, 1420, 1370, 1335, 1317, 1257, 1216, 1184, 1159, 1143, 1123, 1106, 1052, 969, 950, 899, 888, 850, 818, 766, 728, 579. **^{1}H NMR** (599 MHz, benzene-*d*$_6$) δ = 6.70 (d, *J*=1.6 Hz, 1H), 6.24 (d, *J*=1.6 Hz, 1H), 3.43 (s, 3H), 3.38 (ddd, *J*=12.7 Hz, 3.7 Hz, 2.6 Hz, 1H), 2.72 (td, *J*=11.0 Hz, 2.7 Hz, 1H), 2.50 (t, *J*=7.7 Hz, 2H), 2.07 – 1.96 (m, 2H), 1.64 – 1.57 (m, 3H), 1.38 – 1.30 (m, 4H), 1.27 – 1.23 (m, 4H), 1.22 (d, *J*=6.5 Hz, 3H), 1.08 (s, 12H), 1.02 (d, *J*=0.9 Hz, 3H), 0.96 (td, *J*=11.9 H, 3.7 Hz, 1H), 0.90 (dt, *J*=12.6 Hz, 11.4 Hz, 1H), 0.86 – 0.82 (m, 3H). **^{13}C NMR** (151 MHz, benzene-*d*$_6$) δ 159.63,

155.54, 142.44, 112.32, 111.21, 103.34, 82.79, 76.64, 54.84, 50.45, 41.10, 36.52, 36.30, 34.25, 31.93, 31.46, 30.18, 27.95, 25.05, 24.81, 23.13, 22.98, 19.11, 14.25. The relative stereochemistry of the hydroboration product could be assigned via various NMR-experiments. The 6ax-proton shows a pseudo quartett motiv which indicates an axial-axial-coupling. This means 1-H has to be an axial proton.

Total assignment of **14j**:

Atom	Chemical Shift	Atom	Chemical Shift
1 C	34.25	9 C	76.64
H	1.96	10 C	159.63
1c C	14.25	11 C	102.94
H3	0.79	H	6.19
2 C	31.33	12 C	142.44
H	0.92	13 C	110.80
2c C	22.98	H	6.66
H2	1.21	14 C	23.13
3 C	30.18	H3	1.17
Hax	1.28	15 C	27.95
Heq	1.96	H3	0.97
3c C	31.93	16 C	19.11
H2	1.21	H3	1.28
4 C	50.45	17 C	54.84
H	1.56	H3	3.38
4c C	31.50	18 C	82.79
H2	1.56	19 C	82.79
5 C	36.30	20 C	25.05
H	2.67	H3	1.03
5c C	36.52	21 C	25.05
H2	2.46	H3	1.03
6 C	41.10	22 C	24.81
Hax	0.85	H3	1.03
Heq	3.34	23 C	24.81
7 C	111.92	H3	1.03
8 C	155.54		

2.3.21 2-((3*S*,5*R*,6*S*,8*S*,9*S*,10*R*,13*R*,14*S*,17*R*)-3-Methoxy-5,10,13-trimethyl-17-((*R*)-6-methylheptan-2-yl)hexadecahydro-1*H*-cyclopenta[a]phenanthren-6-yl)-4,4,5,5-tetramethyl-1,3,2-dioxaborolane (14k)

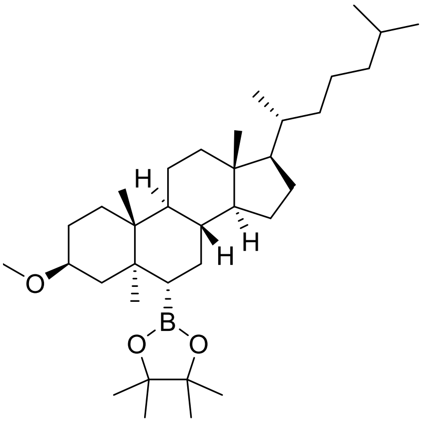

The reaction was performed according to general procedure **GP2** with catecholborane (0.48 g, 4.0 mmol, 8.0 equiv.) and O-methylcholesterol (0.4 g, 0.5 mmol, 1.0 eq.). Purification by column chromatography on silica gel, eluting with pentane:EtOAc (95:5→75:25) gave the boronic ester as a white solid (165 mg, 63% yield). **FTIR** (neat): υ (cm^{-1}) 2930, 2912, 2868, 2850, 1468, 1379, 1319, 1273, 1215, 1165, 1146, 1105, 972, 931, 851. **M.p.**: 138 – 140 °C. $[\boldsymbol{\alpha}]_{D}^{24}$ +23° (*c* 1.2, $CHCl_3$). **^{1}H NMR** (300 MHz, $CDCl_3$) δ 3.31

(s, 3H), 3.24 – 3.05 (m, 1H), 2.01 – 1.67 (m, 5H), 1.65 – 1.44 (m, 4H), 1.39 – 1.27 (m, 6H), 1.24 (s, 6H), 1.23i (s, 6H), 1.22 – 0.92 (m, 14H), 0.91 – 0.84 (m, 9H), 0.78 (s, 3H), 0.73 – 0.66 (m, 1H), 0.63 (s, 3H). **^{13}C NMR** (75 MHz, $CDCl_3$) δ 82.9, 79.8, 56.3, 55.4, 54.1, 45.3, 42.6, 40.1, 39.5, 36.7, 36.2, 35.8, 35.6, 35.6, 33.7, 33.5, 28.3, 28.0, 27.9, 24.9, 24.6, 24.2, 23.9, 22.8, 22.6, 21.2, 18.7, 12.4, 12.1, . The signal of the α-B-carbon was not observed. **HRMS** (ESI) *m/z* = 551.4606 calcd. for $C_{34}H_{61}BO_3$ Na^+ $[M+Na]^+$, found: 551.4625

2.4 MATTESON-type CH_2-homologations

2.4.1 2-(3-(3,4-Dimethylphenyl)butyl)-4,4,5,5-tetramethyl-1,3,2-dioxaborolane (15a)

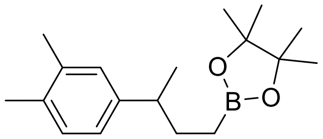

The reaction was performed according to general procedure (GP3) with boronic ester **14a** (54.8 mg, 0.20 mmol). The obtained 1° boronic ester was used without further purification.

2.4.2 2-(3-(2-Methoxyphenyl)butyl)-4,4,5,5-tetramethyl-1,3,2-dioxaborolane (15b)

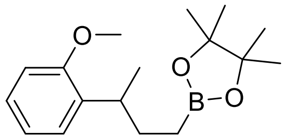

The reaction was performed according to general procedure (GP3) with boronic ester **14b** (55.2 mg, 0.20 mmol). The obtained 1° boronic ester was used without further purification.

2.4.3 2-(3-([1,1'-Biphenyl]-4-yl)butyl)-4,4,5,5-tetramethyl-1,3,2-dioxaborolane (15c)

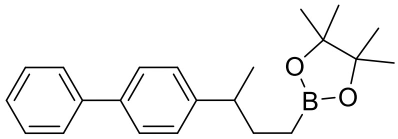

The reaction was performed according to general procedure (GP3) with boronic ester **14c** (64.5 mg, 0.20 mmol). The obtained 1° boronic ester was used without further purification.

2.4.4 2-(3,3-bis(4-fluorophenyl)propyl)-4,4,5,5-tetramethyl-1,3,2-dioxaborolane (15d)

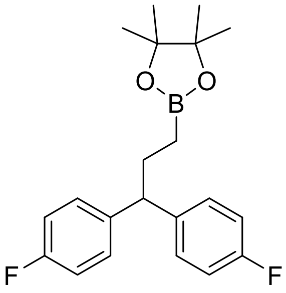

The reaction was performed according to general procedure (GP3) with boronic ester **14d** (68.8 mg, 0.20 mmol). The obtained 1° boronic ester was used without further purification.

2.4.5 2-(3-(Benzo[b]thiophen-2-yl)butyl)-4,4,5,5-tetramethyl-1,3,2-dioxaborolane (15e)

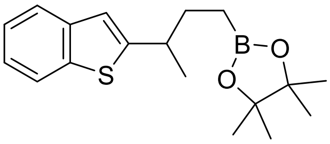

The reaction was performed according to general procedure (**GP3**) with boronic ester **14e** (60.4 mg, 0.20 mmol). The obtained 1° boronic ester was used without further purification.

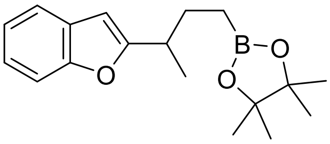

2.4.6 2-(3-(Benzofuran-2-yl)butyl)-4,4,5,5-tetramethyl-1,3,2-dioxaborolane (15f)

The reaction was performed according to general procedure (GP3) with boronic ester **14f** (57.2 mg, 0.20 mmol). The obtained 1° boronic ester was used without further purification.

2.4.7 Triphenyl(4-(4,4,5,5-tetramethyl-1,3,2-dioxaborolan-2-yl)butyl)silane (15g)

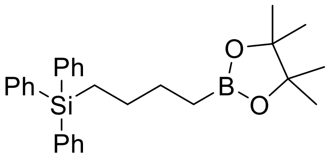

The reaction was performed according to general procedure (GP3) with boronic ester **14g** (85.7 mg, 0.20 mmol). The obtained 1° boronic ester was used without further purification.

2.4.8 4-(4-(4,4,5,5-Tetramethyl-1,3,2-dioxaborolan-2-yl)butan-2-yl)phenyl 4-methylbenzenesulfonate (15h)

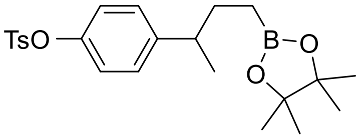

The reaction was performed according to general procedure (**GP3**) with boronic ester **14h** (83.3 mg, 0.20 mmol). The obtained 1° boronic ester was used without further purification.

2.4.9 2-(3-(2-methoxyphenyl)butyl)-4,4,5,5-tetramethyl-1,3,2-dioxaborolane (15i)

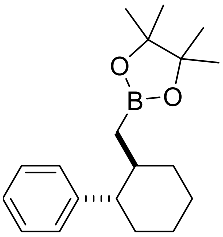

The reaction was performed according to general procedure (GP3) with boronic ester **14i** (55.2 mg, 0.20 mmol). The obtained 1° boronic ester was used without further purification.

2.4.10 2-(((6a*R*,8*S*,9*S*,10a*R*)-1-Methoxy-6,6,9-trimethyl-3-pentyl-6a,7,8,9,10,10a-hexahydro-6*H*-benzo[c]chromen-8-yl)methyl)-4,4,5,5-tetramethyl-1,3,2-dioxaborolane (15j)

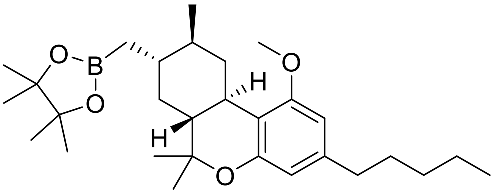

The reaction was performed according to general procedure (**GP3**) with boronic ester **14j** (25.1 mg, 0.0550 mmol). The obtained 1° boronic ester was used without further purification. The crude product was characterized by GC-MS (EI): m/z = 470.4 calcd for $C_{19}H_{29}BO_4^+$ $[M]^+$, found 470.4

2.4.11 2-(((3*S*,5*R*,6*S*,8*S*,9*S*,10*S*,13*R*,14*S*,17*R*)-3-Methoxy-10,13-dimethyl-17-((*R*)-6-methylheptan-2-yl)hexadecahydro-1*H*-cyclopenta[a]phenanthren-6-yl)methyl)-4,4,5,5-tetramethyl-1,3,2-dioxaborolane (15k)

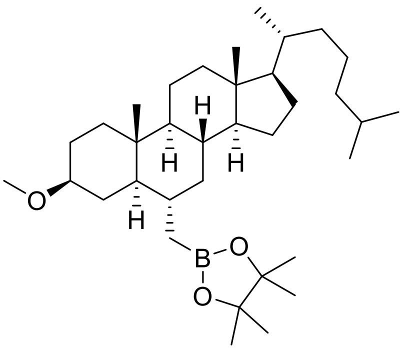

The reaction was performed according to general procedure (**GP3**) with boronic ester **14k** (106 mg, 0.200 mmol). The obtained 1° boronic ester was used without further purification. The crude product was characterized by GC-MS (EI): m/z = 542.5 calcd for $C_{19}H_{29}BO_4^+$ $[M]^+$, found 542.5

2.5 Protodeborylation reactions

2.5.1 Propylbenzene (2a)

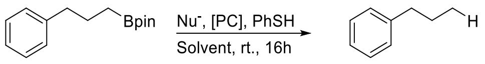

Table 1. Optimization studies.

Nucleophile (X eq)	[PC] (2%)[b]	PhSH (eq.)	Solvent	Yield [c]
TBAF (2.0)	$Ru(bpy)_3(PF_6)_2$	0.5[a]	PhH	0%
TBAF (2.0)	Mes-Acr-Me	0.5[a]	PhH	0%
TBAF (2.0)	[Ir]	0.5[a]	PhH	0%
TBAF (2.0)	[Ir]	0.5	MeOH/ACN	0%
DMAP (1.0)	[Ir]	0.5	MeOH/ACN	0%
DMAP (1.0)	[Ir]	0.5	MeOH/Acetone	0%
PPh_3 (1.0)	[Ir]	0.5	MeOH/Acetone	0%
3-Quinolidinol (1.0)	[Ir]	0.5	MeOH/Acetone	0%
PhLi (1.1)	[Ir]	0.5	MeOH/Acetone	15%
PhLi (1.1)	[Ir]	1.1	MeOH/Acetone	79%
PhLi (1.1)	[Ir]	0.1[d]	MeOH/Acetone	94%
PhLi (1.1)	-/-	1.1	MeOH/Acetone	0%

[a] 1 eq. $(PhO)_2PO(OH)$ added. [b] [Ir] = $Ir(dF(CF_3)ppy)_2(dtbbpy)PF_6$. [c] yield determined via GC. [d] 1.1 eq. $(PhO)_2PO(OH)$ added.

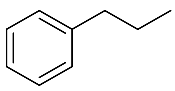

The reaction was performed according to general procedure (**GP4**) with boronic ester **1a** (55.2 mg, 0.20 mmol). The yield was determined *via* GC-FID with commercial propylbenzene as reference material (79%). *n*-Dodecane was used as the internal standard.

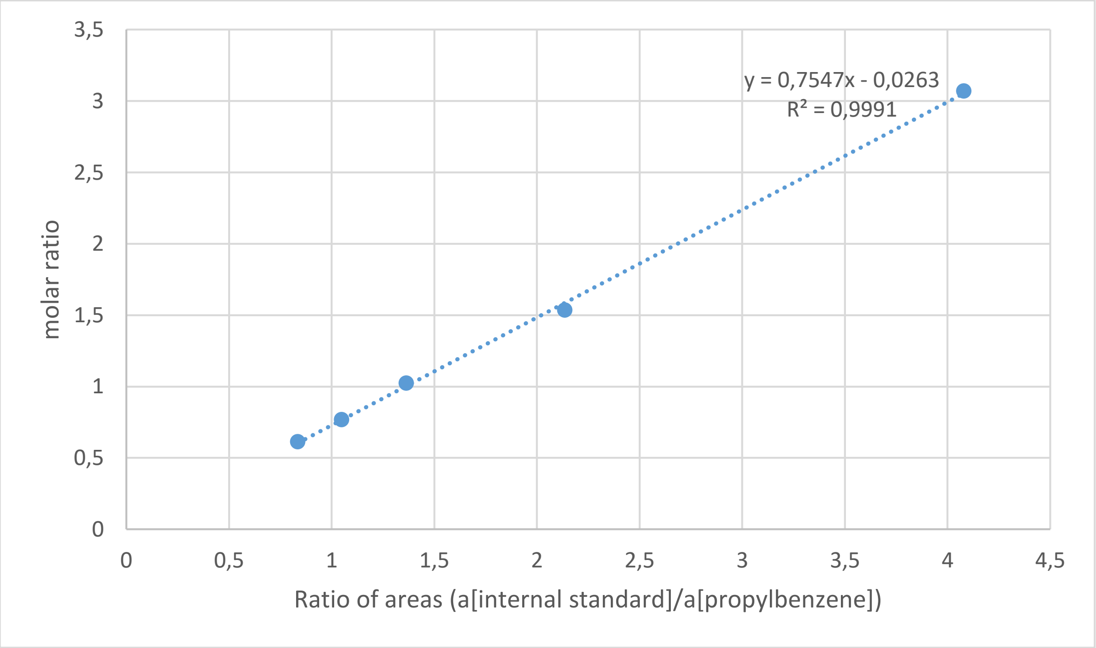

2.5.2 1-Methoxy-4-propylbenzene (2b)

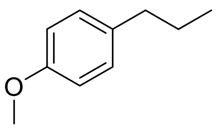

The reaction was performed according to general procedure (**GP4**) with boronic ester **1b** (55.2 mg, 0.200 mmol). Purification *via* column chromatography on silica gel gave the product **2b** as a colorless oil (18.9 mg, 0.126 mmol, 63%). **^{1}H NMR** (300 MHz, $CDCl_3$) δ = 7.15 – 7.06 (m, 2H), 6.89 – 6.80 (m, 2H), 3.79 (s, 3H), 2.54 (t, *J*=7.9 Hz, 2H), 1.69 – 1.55 (m, 2H), 0.94 (t, *J*=7.3 Hz, 3H). **^{13}C NMR** (75 MHz, $CDCl_3$) δ = 157.8, 135.0, 129.5, 113.8, 55.4, 37.3, 24.9, 13.9. The spectroscopic data are in accordance with those described in the literature.[18]

2.5.3 1,2-Dimethoxy-4-propylbenzene (2c)

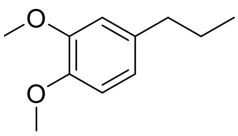

The reaction was performed according to general procedure (**GP4**) with boronic ester **1c** (61.2 mg, 0.200 mmol). Purification *via* column chromatography on silica gel gave the product **2c** as a colorless oil (33.1 mg, 0.184 mmol, 92%). **^{1}H NMR** (300 MHz, $CDCl_3$) δ = 6.79 (m, 1H), 6.71 (m, 2H), 3.87 (s, 3H), 3.86 (s, 3H), 2.53 (t, *J*=7.8 Hz, 2H), 1.70 – 1.56 (m, 2H), 0.94 (t, *J*=7.3 Hz, 3H). **^{13}C NMR** (75 MHz, $CDCl_3$) δ = 148.9, 147.2, 135.5, 120.4, 112.0, 111.4, 56.1, 55.9, 37.8, 24.9, 13.9. The spectroscopic data are in accordance with those described in the literature.[19]

2.5.4 4-Isopropyl-1,1'-biphenyl (2d)

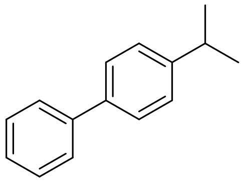

The reaction was performed according to general procedure (**GP4**) with boronic ester **1d** (55.2 mg, 0.200 mmol). Purification *via* column chromatography on silica gel gave the product **2d** as a colorless oil (34.4 mg, 0.174 mmol, 87%). **^{1}H NMR** (300 MHz, $CDCl_3$) δ = 7.54 – 7.41 (m, 4H), 7.37 – 7.30 (m, 2H), 7.27 – 7.19 (m, 3H), 2.87 (hept, *J*=6.9 Hz, 1H), 1.21 (d, *J*=6.9 Hz, 6H). **^{13}C NMR** (75 MHz, $CDCl_3$) δ = 148.2, 141.4, 138.9, 128.8, 127.2, 127.2, 127.1, 127.0, 34.0, 24.2. The spectroscopic data are in accordance with those described in the literature. [20]

2.5.5 1-(*tert*-Butyl)-4-isopropylbenzene (2e)

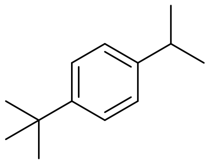

The reaction was performed according to general procedure (**GP4**) with boronic ester **1e** (60 mg, 0.20 mmol). FC (P) afforded compound **2e** (26 mg, 0.15 mmol, 74%) as a colorless oil. **^{1}H NMR** (300 MHz, $CDCl_3$, 299 K) δ = 7.37 – 7.31 (m, 2H), 7.23 – 7.16 (m, 2H), 2.91 (hept, *J*=6.9 Hz, 1H), 1.34 (s, 9H), 1.29 (s, 3H), 1.26 (s, 3H). **^{13}C NMR**

(75 MHz, $CDCl_3$, 299 K) δ = 148.6, 145.9, 126.2, 125.3, 34.5, 33.7, 31.6, 24.2. Spectroscopic data are in accordance with those described in the literature.[21]

2.5.6 1,1-Diphenylethan (2f)

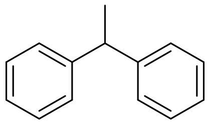

The reaction was performed according to general procedure (**GP4**) with boronic ester **1f** (61.6 mg, 0.200 mmol). Purification *via* column chromatography on silica gel gave the product **2f** as a colorless oil (27.3 mg, 0.150 mmol, 75%). **^{1}H NMR** (300 MHz, $CDCl_3$) δ = 7.35 – 7.17 (m, 10H), 4.18 (q, *J*=7.2 Hz, 1H), 1.67 (d, *J*=7.2 Hz, 3H), **^{13}C NMR** (75 MHz, $CDCl_3$) δ = 146.6, 128.5, 127.8, 126.2, 44.9, 22.0. The spectroscopic data are in accordance with those described in the literature.[22]

2.5.7 1,1-Bis(4-methoxyphenyl)ethan (2g)

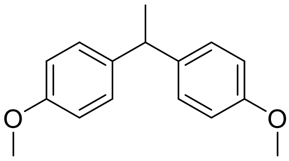

The reaction was performed according to general procedure (**GP4**) with boronic ester **1g** (73.6 mg, 0.200 mmol). Purification *via* column chromatography on silica gel gave the product **2g** as a colorless solid (44.5 mg, 0.184 mmol, 92%). **^{1}H NMR** (300 MHz, $CDCl_3$) δ = 7.18 – 7.11 (m, 4H), 6.89 – 6.81 (m, 4H), 4.08 (q, *J*=7.2 Hz, 1H), 3.79 (s, 6H), 1.61 (d, *J*=7.2 Hz, 3H). **^{13}C NMR** (75 MHz, $CDCl_3$) δ = 158.0, 139.1, 128.6, 113.9, 55.4, 43.3, 22.4. The spectroscopic data are in accordance with those described in the literature.[23,24]

2.5.8 2-Ethylnaphthalene (2h)

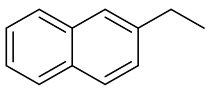

The reaction was performed according to general procedure (**GP4**) with boronic ester **1h** (56.4 mg, 0.200 mmol). Purification *via* column chromatography on silica gel, eluting with pentane, gave the product **2h** as a colorless oil (20.0 mg, 0.128 mmol, 64%). **^{1}H NMR** (300 MHz, $CDCl_3$) δ = 7.87 – 7.74 (m, 3H), 7.65 (bs, 1H), 7.52 – 7.33 (m, 3H), 2.84 (q, J=7.6 Hz, 2H), 1.35 (t, J=7.6 Hz, 3H). **^{13}C NMR** (75 MHz, $CDCl_3$) δ 141.9, 133.9, 132.1, 128.0, 127.7, 127.6, 127.2, 126.0, 125.7, 125.2, 29.2, 15.6. The spectroscopic data are in accordance with those described in the literature.[25]

A gram-scale reaction was performed using boronic ester **1h** (1.0 g, 3.5 mmol, 1.0 equiv.), phenyllithium (1.9 M, Bu_2O, 2.1 mL, 1.1 equiv.), Et_2O (35 ml), thiophenol (0.42 g, 3.9 mmol, 1.1 equiv.) and **PC1** (80 mg, 2 mol%). The radiation was carried out using a 30W LED with a wave length of 465 nm. The product **2h** was isolated as a colorless oil (0.33 g, 2.1 mmol, 61%).

2.5.9 Triphenylpropylsilane (2i)

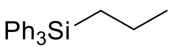

The reaction was performed according to general procedure (**GP4**) with boronic ester **1i** (85.7 mg, 0.200 mmol). Purification *via* column chromatography on silica gel gave the product **2i** as a colorless solid (34.4 mg, 0.144 mmol, 72%). **^{1}H NMR** (300 MHz, $CDCl_3$) δ = 7.61 – 7.50 (m, 6H), 7.47 – 7.33 (m, 9H), 1.63 – 1.47 (m, 2H), 1.45 – 1.36 (m, 2H), 1.03 (t, *J*=7.0 Hz, 3H). **^{13}C NMR** (75 MHz, $CDCl_3$) δ = 135.8, 135.6, 129.5, 128.0, 18.7, 17.8, 16.1. The spectroscopic data are in accordance with those described in the literature.[26]

2.5.10 *N*-Methylcarbazole (2j)

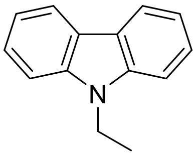

The reaction was performed according to general procedure (**GP4**) with boronic ester **1j** (64.2 mg, 0.200 mmol). Purification *via* column chromatography on silica gel gave the product **2j** as a colorless solid (34.5 mg, 0.176 mmol, 88%). **^{1}H NMR** (300 MHz, $CDCl_3$) δ = 8.15 (dt, *J*=7.8 Hz, 1.0, 2H), 7.58 – 7.39 (m, 4H), 7.27 (ddd, *J*=7.9 Hz, 6.9 Hz, 1.2 Hz, 2H), 4.40 (q, *J*=7.2 Hz, 2H), 1.46 (t, *J*=7.2 Hz, 3H). **^{13}C NMR** (75 MHz, $CDCl_3$) δ 140.1, 125.7, 123.1, 120.5, 118.9, 108.6, 37.6, 13.9. The spectroscopic data are in accordance with those described in the literature.[27]

2.5.11 5-(pentan-2-yl)benzo[d][1,3]dioxole (2k)

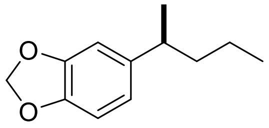

The reaction was performed according to general procedure (**GP4**) with boronic ester **1k** (63.6 mg, 0.200 mmol). Purification *via* column chromatography on silica gel gave the product **2k** as a colorless solid (20.5 mg, 0.106 mmol, 53%). **^{1}H NMR** (300 MHz, $CDCl_3$) δ = 6.73 (d, *J*=7.9 Hz, 1H), 6.69 (d, *J*=1.7 Hz, 1H), 6.63 (dd, *J*=7.9 Hz, 1.7 Hz, 1H), 5.92 (s, 2H), 2.71 – 2.55 (m, 1H), 1.59 – 1.43 (m, 2H), 1.28 – 1.15 (m, 5H), 0.87 (t, *J*=7.3 Hz, 3H). **^{13}C NMR** (75 MHz, $CDCl_3$) δ 147.7, 145.6, 142.2, 120.0, 108.1, 107.4, 100.8, 41.0, 39.6, 22.7, 20.9, 14.2. The spectroscopic data are in accordance with those described in the literature.[27] Enantiomeric ratio er = 96:4 was determined *via* chiral HPLC (see appendix).

2.5.12 Phenylethylsulfon (2l)

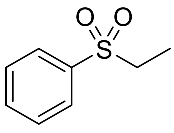

The reaction was performed according to general procedure (**GP4**) with boronic ester **1l** (59.4 mg, 0.200 mmol). Purification *via* column chromatography on silica gel gave the product **2l** as a colorless solid (29.4 mg, 0.170 mmol, 85%). **^{1}H NMR** (300 MHz, $CDCl_3$) δ = 7.94 – 7.88 (m, 2H), 7.69 – 7.62 (m, 1H), 7.56 (m, 2H), 3.11 (q, J=7.4 Hz, 2H), 1.27 (t, J=7.4 Hz,

3H). **^{13}C NMR** (75 MHz, $CDCl_3$) δ 138.8, 133.8, 129.4, 128.3, 50.7, 7.5. The spectroscopic data are in accordance with those described in the literature. [28]

2.5.13 *tert*-Butyl pyrrolidine-1-carboxylate (2m)

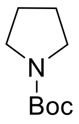

The reaction was performed according to general procedure (**GP4**) with boronic ester **1m** (59 mg, 0.20 mmol, 1.0 equiv.). FC (P:Et_2O = 5:1) afforded compound **2m** (32 mg, 0.19 mmol, 93%) as a colorless oil. **^{1}H NMR** (300 MHz, $CDCl_3$, 299 K) δ = 3.38 – 3.23 (m, 4H), 1.89 – 1.77 (m, 4H), 1.46 (s, 9H).**^{13}C NMR** (75 MHz, $CDCl_3$, 299 K) δ = 154.9, 79.1, 46.0, 28.7, 25.5. Spectroscopic data are in accordance with those described in the literature.[29]

2.5.14 *tert*-Butyl-4-methyloctanoate (2n)

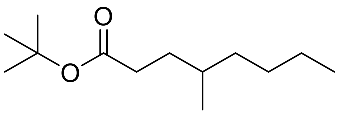

The reaction was performed according to general procedure (**GP4**) with boronic ester **1n** (68.1 mg, 0.200 mmol). Purification *via* column chromatography on silica gel gave the product **2n** as a colorless oil (31.3 mg, 0.146 mmol, 73%). **^{1}H NMR** (500 MHz, C_6D_6, 299 K) δ = 2.12 – 2.24 (m, 2H), 1.66 – 1.73 (m, 1H), 1.40 – 1.48 (m, 10H), 1.26 – 1.38 (m, 3H), 1.15 – 1.24 (m, 4H), 0.87 (t, *J*=7.0 Hz, 3H), 0.78 (d, *J*=6.6 Hz, 3H). **^{13}C NMR** (126 MHz, C_6D_6) δ 172.82, 79.34, 36.78, 33.57, 32.69, 32.48, 29.57, 28.19, 23.33, 19.49, 14.33. **HRMS** (ESI) *m/z* = 237.1825 calcd. for $C_{13}H_{26}O_2Na$ $[M+Na]^+$, found: 237.1840.

2.5.15 4-(*sec*-Butyl)-1,2-dimethylbenzene (16a)

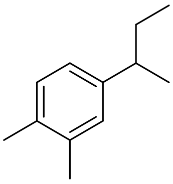

The reaction was performed according to **GP4** with boronic ester **15a** (crude from homologation reaction, 0.20 mmol, 1.0 equiv.), phenyllithium (1.9 M, Bu_2O, 0.12 mL, 0.22 mmol, 1.1 equiv.), thiophenol (23 µL, 0.22 mmol, 1.1 equiv) and Ir(dFCF$_3$ppy)$_2$(dtbbpy)PF$_6$ (5.0 mg, 5.0 µmol, 2.5 mol%) in acetone/MeOH (1:1, 2.0 mL). FC (P) afforded compound **16a** (21.0 mg, 0.13 mmol, 66%) as a colorless oil. **^{1}H NMR** (300 MHz, $CDCl_3$, 299 K) δ = 7.08 (d, *J*=7.6 Hz, 1H), 7.00 – 6.91 (m, 2H), 2.64 – 2.46 (m, 1H), 2.30 – 2.27 (s, 3H), 2.26 (s, 3H), 1.67 – 1.54 (m, 2H), 1.24 (d, *J*=7.0 Hz, 3H), 0.85 (t, *J*=7.4 Hz, 3H). **^{13}C NMR** (75 MHz, $CDCl_3$, 299 K) δ = 145.4, 136.4, 133.9, 129.7, 128.6, 124.5, 41.4, 31.3, 22.1, 20.0, 19.4, 12.5.

2.5.16 1-(*sec*-Butyl)-2-methoxybenzene (16b)

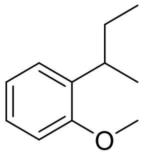

The reaction was performed according to **GP4** with boronic ester **15b** (crude from homologation reaction, 0.20 mmol, 1.0 equiv.), phenyllithium (1.9 M, Bu_2O, 0.12 mL, 0.22 mmol, 1.1 equiv.), thiophenol (23 µL, 0.22 mmol, 1.1 equiv) and Ir(dFCF$_3$ppy)$_2$(dtbbpy)PF$_6$ (5.0 mg, 5.0 µmol, 2.5 mol%) in acetone/MeOH (1:1, 2.0 mL). FC (P) afforded compound **16b** (21.3 mg, 0.13 mmol, 65%) as a colorless oil. **^{1}H NMR** (300 MHz, $CDCl_3$, 299 K) δ = 7.22 – 7.11 (m, 1H), 6.94 (td, *J*=7.4 Hz, 1.2, 1H), 6.91 – 6.82 (m, 1H), 3.83 (s, 3H), 3.20 – 3.04 (m, 1H), 1.75 – 1.47 (m, 2H), 1.21 (d, *J*=7.0 Hz, 3H), 0.86 (t, *J*=7.4 Hz, 3H). **^{13}C NMR** (75 MHz, $CDCl_3$, 299 K) δ = 157.1, 136.0, 126.8, 126.4, 120.6, 110.5, 55.4, 33.5, 29.8, 20.5, 12.2.

2.5.17 4-(*sec*-Butyl)-1,1'-biphenyl (16c)

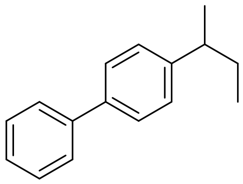

The reaction was performed according to **GP4** with boronic ester **15c** (crude from homologation reaction, 0.20 mmol, 1.0 equiv.), phenyllithium (1.9 M, Bu_2O, 0.12 mL, 0.22 mmol, 1.1 equiv.), thiophenol (23 µL, 0.22 mmol, 1.1 equiv) and Ir(dFCF$_3$ppy)$_2$(dtbbpy)PF$_6$ (5.0 mg, 5.0 µmol, 2.5 mol%) in acetone/MeOH (1:1, 2.0 mL). FC (P) afforded compound **16c** (28.0 mg, 0.13 mmol, 66%) as a colorless oil. **^{1}H NMR** (300 MHz, $CDCl_3$, 299 K) δ = 7.66 – 7.61 (m, 2H), 7.60 – 7.52 (m, 2H), 7.50 – 7.42 (m, 2H), 7.40 – 7.26 (m, 3H), 2.62-2.77 (m, 1H), 1.62 – 1.75 (m, 2H), 1.32 (d, *J*=7.0 Hz, 3H), 0.91 (t, *J*=7.4 Hz, 3H). **^{13}C NMR** (75 MHz, $CDCl_3$, 299 K) δ = 146.9, 141.2, 138.7, 128.7, 127.5, 127.0, 127.0, 127.0, 41.4, 31.2, 21.8, 12.3. Spectroscopic data are in accordance with those described in the literature.[33]

2.5.18 4,4'-(Propane-1,1-diyl)bis(fluorobenzene) (16d)

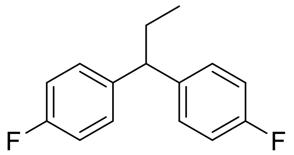

The reaction was performed according to **GP4** with boronic ester **15d** (crude from homologation reaction, 0.20 mmol, 1.0 equiv.), phenyllithium (1.9 M, Bu_2O, 0.12 mL, 0.22 mmol, 1.1 equiv.), thiophenol (23 µL, 0.22 mmol, 1.1 equiv) and Ir(dFCF$_3$ppy)$_2$(dtbbpy)PF$_6$ (5.0 mg, 5.0 µmol, 2.5 mol%) in acetone/MeOH (1:1, 2.0 mL). FC (P) afforded compound **16d** (30.7 mg, 0.13 mmol, 66%) as a colorless oil. **^{1}H NMR** (300 MHz, $CDCl_3$, 299 K) δ = 7.20 – 7.12 (m, 4H), 7.02 – 6.92 (m, 4H), 3.77 (t, *J*=7.8 Hz, 1H), 2.10 – 1.95 (m, 2H), 0.89 (t, *J*=7.3 Hz, 3H). **^{13}C NMR** (75 MHz, $CDCl_3$, 299 K) δ =°161.5 (d, *J* = 244.2 Hz), 140.8 (d, *J* = 3.2 Hz), 129.3 (d, *J* = 7.8 Hz), 115.3 (d, *J* = 21.1 Hz), 51.8, 29.0, 12.8. Spectroscopic data are in accordance with those described in the literature.[34]

2.5.19 2-(*sec*-Butyl)benzo[b]thiophene (16e)

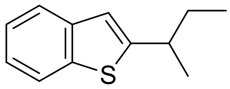

The reaction was performed according to **GP4** with boronic ester **15e** (crude from homologation reaction, 0.20 mmol, 1.0 equiv.), phenyllithium (1.9 M, Bu_2O, 0.12 mL, 0.22 mmol, 1.1 equiv.), thiophenol (23 µL, 0.22 mmol, 1.1 equiv) and $Ir(dFCF_3ppy)_2(dtbbpy)PF_6$ (5.0 mg, 5.0 µmol, 2.5 mol%) in acetone/MeOH (1:1, 2.0 mL). FC (P) afforded compound **16e** (19.5 mg, 0.10 mmol, 51%) as a colorless oil. **^{1}H NMR** (300 MHz, $CDCl_3$, 299 K) δ = 7.81 – 7.74 (m, 1H), 7.70 – 7.63 (m, 1H), 7.35 – 7.21 (m, 2H), 7.02 (bs, 1H), 3.09 – 2.92 (m, 1H), 1.83 – 1.64 (m, 2H), 1.39 (d, *J*=6.9 Hz, 3H), 0.94 (t, *J*=7.4 Hz, 3H). **^{13}C NMR** (75 MHz, $CDCl_3$, 299 K) δ = 152.8, 140.1, 138.9, 124.0, 123.3, 122.7, 122.2, 119.1, 37.8, 31.7, 22.2, 11.9. **HRMS** (EI): m/z = 190.0811 for $C_{12}H_{14}S^+$ $[M]^+$, found: 190.0812.

2.5.20 2-(*sec*-Butyl)benzofuran (16f)

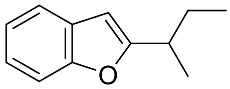

The reaction was performed according to **GP4** with boronic ester **15f** (crude from homologation reaction, 0.20 mmol, 1.0 equiv.), phenyllithium (1.9 M, Bu_2O, 0.12 mL, 0.22 mmol, 1.1 equiv.), thiophenol (23 µL, 0.22 mmol, 1.1 equiv) and $Ir(dFCF_3ppy)_2(dtbbpy)PF_6$ (5.0 mg, 5.0 µmol, 2.5 mol%) in acetone/MeOH (1:1, 2.0 mL). FC (P) afforded compound **16f** (19.0 mg, 0.11 mmol, 55%) as a colorless oil. **^{1}H NMR** (300 MHz, $CDCl_3$, 299 K) δ = 7.53 – 7.46 (m, 1H), 7.45 – 7.39 (m, 1H), 7.24 – 7.13 (m, 2H), 6.38 (bs, 1H), 2.94 – 2.80 (m, 1H), 1.92 – 1.75 (m, 1H), 1.73 – 1.58 (m, 1H), 1.34 (d, *J*=7.0 Hz, 3H), 0.94 (t, *J*=7.4 Hz, 3H). **^{13}C NMR** (75 MHz, $CDCl_3$, 299 K) δ = 164.0, 154.7, 129.1, 123.1, 122.5, 120.4, 110.9, 100.9, 35.3, 28.4, 18.6, 11.7. Spectroscopic data are in accordance with those described in the literature.[31]

2.5.21 Butyltriphenylsilane (16g)

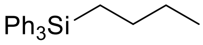

The reaction was performed according to **GP4** with boronic ester **15g** (crude from homologation reaction, 0.20 mmol, 1.0 equiv.), phenyllithium (1.9 M, Bu_2O, 0.12 mL, 0.22 mmol, 1.1 equiv.), thiophenol (23 µL, 0.22 mmol, 1.1 equiv) and $Ir(dFCF_3ppy)_2(dtbbpy)PF_6$ (5.0 mg, 5.0 µmol, 2.5 mol%) in acetone/MeOH (1:1, 2.0 mL). FC (P) afforded compound **16g** (44.4 mg, 0.14 mmol, 70%) as a colorless solid. **^{1}H NMR** (300 MHz, $CDCl_3$, 299 K) δ = 7.58 – 7.50 (m, 6H), 7.46 – 7.32 (m, 9H), 1.57 – 1.32 (m, 6H), 0.89 (t, *J*=6.9 Hz, 3H). **^{13}C NMR** (75 MHz, $CDCl_3$, 299 K) δ = 135.8, 135.6, 129.5, 128.0, 26.9, 26.3, 13.8, 13.2. Spectroscopic data (^{1}H) are in accordance with those described in the literature.[30]

2.5.22 4-(*sec*-Butyl)phenol (16h)

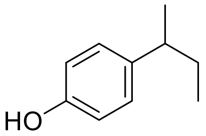

The reaction was performed according to **GP4** with boronic ester **15h** (crude from homologation reaction, 0.20 mmol, 1.0 equiv.), phenyllithium (1.9 M, Bu_2O, 0.12 mL, 0.22 mmol, 1.1 equiv.), thiophenol (23 µL, 0.22 mmol, 1.1 equiv) and $Ir(dFCF_3ppy)_2(dtbbpy)PF_6$ (5.0 mg, 5.0 µmol, 2.5 mol%) in acetone/MeOH (1:1, 2.0 mL). FC (P/ether, 10:1) afforded compound **16h** (14.5 mg, 0.09 mmol, 48%) as a colorless oil. **^{1}H NMR** (300 MHz, $CDCl_3$, 299 K) δ = 7.08 – 7.01 (m, 2H), 6.81 – 6.73 (m, 2H), 4.66 (bs, 1H), 2.58 – 2.36 (m, 1H), 1.54 – 1.35 (m, 2H), 1.20 (d, *J*=7.0 Hz, 3H), 0.81 (t, *J*=7.4 Hz, 3H). **^{13}C NMR** (75 MHz, $CDCl_3$, 299 K) δ = 153.6, 140.2, 128.2, 115.2, 41.0, 31.5, 22.1, 12.3. Spectroscopic data are in accordance with those described in the literature.[32]

2.5.23 *rac*-((1*S*,2*S*)-2-Methylcyclohexyl)benzene (16i)

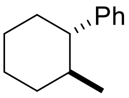

The reaction was performed according to **GP4** with boronic ester **15i** (crude from homologation reaction, 0.20 mmol, 1.0 equiv.), phenyllithium (1.9 M, Bu_2O, 0.12 mL, 0.22 mmol, 1.1 equiv.), thiophenol (23 µL, 0.22 mmol, 1.1 equiv) and $Ir(dFCF_3ppy)_2(dtbbpy)PF_6$ (5.0 mg, 5.0 µmol, 2.5 mol%) in acetone/MeOH (1:1, 2.0 mL). FC (P) afforded compound **16i** (21.3 mg, 0.13 mmol, 61%) as a colorless oil. **^{1}H NMR** (300 MHz, $CDCl_3$, 299 K) δ = 7.39 – 7.31 (m, 2H), 7.27 – 7.21 (m, 3H), 2.14 (td, *J*=11.1 Hz, 3.1 Hz, 1H), 1.95 – 1.80 (m, 4H), 1.65 (m, 1H), 1.56 – 1.35 (m, 3H), 1.24 – 1.10 (m, 1H), 0.73 (d, *J*=6.5 Hz, 3H). **^{13}C NMR** (75 MHz, $CDCl_3$, 299 K) δ = 146.9, 128.2, 127.6, 125.7, 52.6, 37.7, 35.8, 35.7, 27.0, 26.7, 20.8. **HRMS** (EI) *m/z* = 174.1403 calcd. for $C_{13}H_{18}$ $[M]^+$, found: 174.1403.

2.5.24 (6a*R*,8*S*,9*S*,10a*R*)-1-Methoxy-6,6,8,9-tetramethyl-3-pentyl-6a,7,8,9,10,10a-hexahydro-6*H*-benzo[c]chromene (16j)

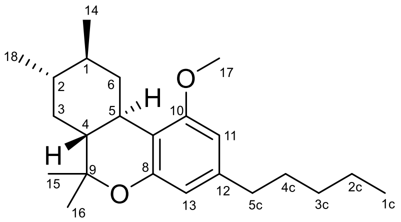

The reaction was performed according to **GP4** with boronic ester **15j** (crude from homologation reaction, 0.055 mmol, 1.0 equiv.), phenyllithium (1.9 M, Bu_2O, 32 µL, 0.060 mmol, 1.1 equiv.), thiophenol (6 µL, 0.06 mmol, 1.1 equiv) and $Ir(dFCF_3ppy)_2(dtbbpy)PF_6$ (1.4 mg, 1.4 µmol, 2.5 mol%) in acetone/MeOH (1:1, 0.5 mL). FC (P:ether 50:1) afforded compound **16j** (17.4 mg, 0.0510 mmol, 92%) as a colorless oil. **^{1}H NMR** (599 MHz, C_6D_6) δ = 6.68 (d, *J*=1.6 Hz, 1H), 6.21 (d, *J*=1.7 Hz, 1H), 3.40 (s, 3H), 3.24 (ddd, *J*=12.9 Hz, 3.8 Hz, 2.8 Hz, 1H), 2.54 (td, *J*=11.0 Hz, 2.8 Hz, 1H), 2.49 – 2.45 (m, 2H), 1.59 – 1.50 (m, 4H), 1.31 (s, 3H), 1.26 – 1.12 (m, 5H), 1.00 (s, 3H), 0.97 – 0.87 (m, 4H), 0.87 – 0.82 (m, 4H), 0.81 – 0.78 (m, 3H), 0.63 (dd, *J*=12.7 Hz, 11.4 Hz, 1H). **^{13}C NMR** (151 MHz, Benzene-d_6)

δ = 159.19, 155.10, 142.15, 111.58, 110.81, 102.89, 76.06, 54.42, 49.43, 39.63, 39.50, 39.27, 37.04, 36.12, 35.96, 31.52, 31.06, 27.58, 22.58, 20.08, 20.06, 18.75, 13.84. **HRMS** (ESI) m/z calcd. for $C_{23}H_{36}O_2Na^+$ $[M+Na]^+$: 367.2608. found: 367.2608. The total assignment was carried out via 2D-NMR and NOE-NMR-experiments.

Atom	Chemical Shift
1 C	39.27
H	1.21
1c C	13.84
H3	0.80
2 C	22.58
H	0.94
2c C	22.58
H2	1.21
3 C	37.04
Hax	0.63
Heq	1.54
3c C	31.52
H2	1.21
4 C	49.43
H	1.54
4c C	31.06
H2	1.54
5 C	35.96
H	2.54
5c C	36.12
H2	2.47
6 C	39.63
Hax	0.83
Heq	3.24

Atom	Chemical Shift
7 C	142.15
8 C	155.10
9 C	76.06
10 C	159.19
11 C	102.89
H	6.21
12 C	111.58
13 C	110.81
H	6.68
14 C	20.08
H3	0.83
15 C	27.58
H3	1.31
16 C	18.75
H3	1.00
17 C	54.42
H3	3.40
18 C	20.06
H3	0.94

2.5.25 (3*S*,5*R*,6*S*,8*R*,9*S*,10*S*,13*R*,14*S*,17*R*)-3-Methoxy-6,10,13,14-tetramethyl-17-((*R*)-6-methylheptan-2-yl)hexadecahydro-1*H*-cyclopenta[a]phenanthrene (16k)

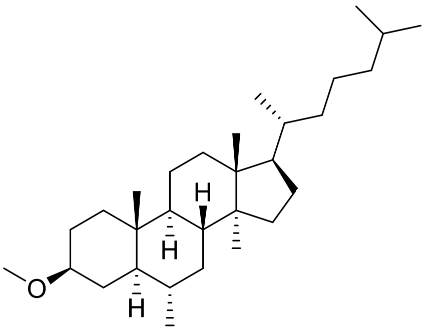

The reaction was performed according to **GP4** with boronic ester **15k** (crude from homologation reaction, 0.055 mmol, 1.0 equiv.), phenyllithium (1.9 M, Bu_2O, 32 µL, 0.060 mmol, 1.1 equiv.), thiophenol (6 µL, 0.060 mmol, 1.1 equiv) and $Ir(dFCF_3ppy)_2(dtbbpy)PF_6$ (1.4 mg, 1.4 µmol, 2.5 mol%) in acetone/MeOH (1:1, 0.5 mL). FC (P:ether 50:1) afforded compound **16k** (17.4 mg, 0.051 mmol, 92%) as a colorless oil. **^{1}H NMR** (599 MHz, $CDCl_3$) δ = 3.35 (s, 3H), 3.07 (tt, *J*=11.1 Hz, 4.7 Hz, 1H), 2.03 – 1.94 (m, 2H), 1.90 – 1.85 (m, 1H), 1.81 (dtd, *J*=13.4 Hz, 9.5 Hz, 6.1 Hz, 1H), 1.73 (dt, *J*=13.3 Hz, 3.7 Hz, 1H), 1.64 – 1.46 (m, 5H),

1.42 – 1.19 (m, 9H), 1.17 – 0.93 (m, 9H), 0.93 – 0.84 (m, 10H), 0.83 – 0.79 (m, 6H), 0.71 – 0.52 (m, 6H). **^{13}C NMR** (151 MHz, $CDCl_3$) δ = 80.51, 56.62, 56.47, 55.71, 54.61, 51.32, 42.70, 42.09, 40.26, 39.67, 37.20, 36.34, 36.11, 35.96, 35.20, 31.23, 30.35, 28.45, 28.16, 27.57, 24.34, 24.01, 22.97, 22.71, 21.45, 20.57, 18.83, 13.44, 12.24. **HRMS** (ESI) *m/z*: 453.4067 calcd. for $C_{16}H_{25}NO_2Na$ $[M+Na]^+$, found 453.4068.

2.6 Synthesis of the indolizidine alkaloides

2.6.1 *tert*-Butyl (S)-2-((R)-4-(*tert*-butoxy)-4-oxo-1-(4,4,5,5-tetramethyl-1,3,2-dioxaborolan-2-yl)butyl)pyrrolidine-1-carboxylate / *tert*-Butyl (S)-2-((S)-4-(*tert*-butoxy)-4-oxo-1-(4,4,5,5-tetramethyl-1,3,2-dioxaborolan-2-yl)butyl)pyrrolidine-1-carboxylate (6)

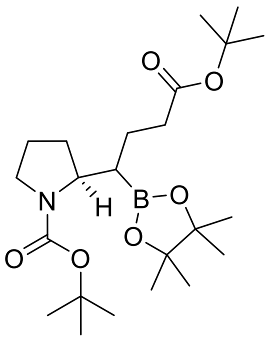

In analogy to a procedure of STUDER and coworkers,[35] vinyl bromide (1.0 M in THF, 3.9 mL, 3.9 mmol, 1.3 equiv.) was dissolved in Et_2O (15 mL) and *tert*-BuLi (1.7 M in pentane, 4.6 mL, 7.8 mmol, 2.3 equiv.) was added dropwise over 5 min at -78 °C. The mixture was stirred at this temperature for 30 min and boronic ester **1m** (892 mg, 3.0 mmol, 1.0 equiv.) in Et_2O (5.0 mL) was added dropwise at -78 °C. The mixture was allowed to warm to room temperature and stirring was continued at that temperature for another 30 min. Subsequently, the solvent was carefully removed *in vacuo* and the resulting residue was taken up in MeCN (20 mL). After the addition of *tert*-butyl 2-iodoacetate (1.5 g, 6.0 mmol, 2.0 equiv.) the tube was sealed and the mixture was stirred under irradiation with a 400 W lamp (temperature up to 50 °C) for 24 h. Removal of the solvent *in vacuo* and FC (P:Et_2O = 4:1) of the crude material afforded the desired boronic ester **6** (688 mg, 1.57 mmol, 52%) as an inseparable mixture of diastereomers (dr = 1.8:1.0) as a colorless oil. **IR** (neat): ν (cm^{-1}) 2975, 2933, 2873, 1729, 1690, 1479, 1458, 1390, 1365, 1316, 1254, 1213, 1140, 1107, 852, 773, 671. **^{1}H NMR (**600 MHz, DMSO-*d*6, 363 K, both diastereomers) δ = 3.91 – 3.77 (m, 1H), 3.46 – 3.39 (m, 1H), 3.16 – 3.09 (m, 1H), 2.30 – 2.21 (m, 1H), 2.17 – 2.08 (m, 1H), 1.94 – 1.86 (m, 1H), 1.84 – 1.75 (m, 1H), 1.72 – 1.47 (m, 5H), 1.43 – 1.39 (m, 18H), 1.22 – 1.19 (m, 12H). **^{13}C NMR (**151 MHz, DMSO-*d*6, 363 K, both diastereomers) δ = 171.7, 171.6, 153.4, 153.3, 82.3, 82.3, 78.8, 78.7, 77.7, 77.6, 57.7, 57.7, 46.6, 45.6, 34.6, 34.3, 29.5, 27.8, 27.8, 27.4, 27.4, 24.2, 24.2, 24.1, 24.1, 23.2, 22.7, 20.9. The signal of the α-B-carbon was not observed. **^{11}B NMR (**192 MHz, DMSO-*d*6, 299 K, both diastereomers) δ = 32.2. **HRMS** (ESI) *m/z* = 462.2997 calcd. for $C_{23}H_{42}BO_6Na$ $[M+Na]^+$, found: 462.3013.

2.6.2 *tert*-Butyl (*S*)-2-((*S*)-5-ethoxy-5-oxo-2-(4,4,5,5-tetramethyl-1,3,2-dioxaborolan-2-yl)pentan-2-yl)pyrrolidine-1-carboxylate / *tert*-Butyl (*S*)-2-((*R*)-5-ethoxy-5-oxo-2-(4,4,5,5-tetramethyl-1,3,2-dioxaborolan-2-yl)pentan-2-yl)pyrrolidine-1-carboxylate (10)

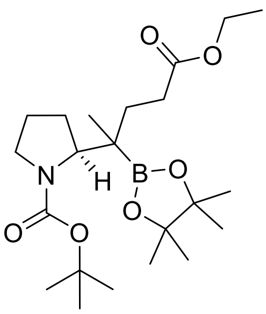

In analogy to a procedure of *Studer* and coworkers,[35] 2-bromopropane (69 µL, 0.78 mmol, 1.3 equiv) was dissolved in Et_2O (3.0 mL) and *tert*-BuLi (1.7 M, pentane, 0.92 mL, 1.6 mmol, 2.6 equiv.) was added dropwise over 5 min at -78 °C. The mixture was stirred at this temperature for 30 min and boronic ester **1m** (178 mg, 60.0 µmol, 1.0 equiv.) in Et_2O (1.0 mL) was added dropwise at -78 °C. The mixture was allowed to warm to room temperature and stirring was continued at that temperature for another 30 min. Subsequently, the solvent was carefully removed *in vacuo* and the resulting residue was taken up in MeCN (4 mL). After the addition of ethyl-2-iodacetate (0.36 mL, 3.0 mmol, 5.0 equiv.) the tube was sealed and the mixture was stirred under irradiation with a 400 W lamp (temperature up to 50 °C) for 24 h. Removal of the solvent *in vacuo* and FC (P:Et_2O = 5:1→4:1) of the crude material afforded the desired boronic ester **10** (160 mg, 0.39 mmol, 65%) as an inseparable mixture of diastereomers (dr = 1.5:1.0) as a colorless oil.

IR (neat): ν (cm^{-1}) 2976, 2932, 2337, 1734, 1685, 1559, 1457, 1448, 1419, 1366, 1341, 1311, 1281, 1251, 1209, 1163, 1144, 1112, 1102, 1073, 1036, 968, 935, 913, 856, 772, 668. **^{1}H NMR (**500 MHz, DMSO-*d*6, 363 K, both diastereomers) δ = 4.07 – 4.02 (m, 2H), 4.00 – 3.76 (m, 1H), 3.64 – 3.51 (m, 1H), 3.12 – 3.03 (m, 1H), 2.39 – 2.19 (m, 2H), 1.95 – 1.60 (m, 5H), 1.51 – 1.43 (m, 1H), 1.44 – 1.39 (m, 9H), 1.23 – 1.20 (m, 12H), 1.18 (t, *J*=7.1 Hz, 3H), 0.82 – 0.73 (m, 3H). **^{13}C NMR (**126 MHz, DMSO-*d*6, 363 K, both diastereomers) δ = 172.8, 172.8, 154.6, 154.2, 82.3 (2x), 81.9 (2x), 78.4, 78.2, 61.9 (2x), 59.0, 58.9, 47.1, 46.8, 30.3 (2x), 30.3, 30.1 (2x), 30.1, 27.7 (3x), 27.7 (3x), 24.8 (2x), 24.7 (2x), 24.3 (2x), 24.3 (2x), 23.4, 23.2, 16.6 (2x), 13.5 (2x). The signal of the α-B-carbon was not observed. **^{11}B NMR (**96 MHz, DMSO-*d*6, 299 K, both diastereomers) δ = 28.4. **HRMS** (ESI) *m/z* = 448.2841 calcd. for $C_{22}H_{40}BNO_6Na$ $[M+Na]^+$, found: 448.2853.

2.6.3 *tert*-Butyl (*R*)-2-(4-(*tert*-butoxy)-4-oxobutyl)pyrrolidine-1-carboxylate (7)

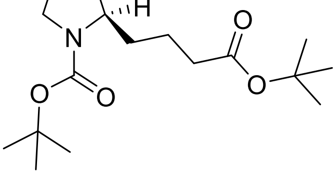

To a solution of boronic ester **6** (44 mg, 0.10 mmol, 1.0 equiv.) in Et_2O (1.0 mL) was added phenyllithium (1.9 M, Bu_2O, 60 µL, 0.11 mmol, 1.1 equiv.) at -78° C and the mixture was stirred for 5 min at that temperature. The mixture was allowed to warm up to room temperature, stirred for 30 min at that temperature and subsequently subjected to **GP4** with thiophenol (11 µL, 0.11 mmol, 1.1 equiv.) and Ir(dFCF$_3$ppy)$_2$(dtbbpy)PF$_6$ (2.5 mg, 2.5 µmol,

2.5 mol%) in acetone/MeOH (1:1, 1.0 mL). FC (P:Et_2O = 5:1) afforded compound **7** (19 mg, 61 µLmol, 61%) as a colorless oil.

$[\alpha]_D^{25}$= -29.6 (*c* 1.0 CH_2Cl_2). **IR** (neat): ν (cm^{-1}) 2973, 2933, 2872, 1729, 1691, 1479, 1456, 1391, 1365, 1253, 1146, 1118, 1101, 953, 916, 879, 850, 772, . **^{1}H NMR (**600 MHz, DMSO-*d*6, 363 K) δ = 3.72 – 3.65 (m, 1H), 3.34 – 3.28 (m, 1H), 3.25 – 3.16 (m, 1H), 2.19 (t, *J*=7.2 Hz, 2H), 1.95 – 1.87 (m, 1H), 1.84 – 1.71 (m, 2H), 1.67 – 1.57 (m, 2H), 1.53 – 1.45 (m, 2H), 1.42 (s, 18H), 1.37 – 1.30 (m, 1H). **^{13}C NMR (**151 MHz, DMSO-*d*6, 363 K) δ = 171.5, 153.2, 78.9, 77.6, 56.1, 45.5, 34.5, 33.2, 29.6, 27.8 (3x), 27.4 (3x), 22.5, 21.0. **HRMS** (ESI) *m/z* = 336.2145 calcd. for $C_{17}H_{31}NO_4Na$ $[M+Na]^+$, found: 336.2162.

2.6.4 (*R*)-Hexahydroindolizidine-5(1*H*)-one (8)

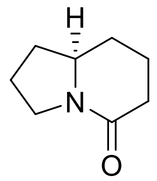

Pyrrolidine **7** (45 mg, 0.14 mmol, 1.0 equiv.) was dissolved in a HCl-solution (4 M, in dioxane, 4.5 mL) and stirred at room temperature for 8 h. The solvent was removed *in vacuo* and the residue was taken up in CH_2Cl_2 (3 mL). Subsequently, diisopropylethylamine (48 µL, 0.28 mmol, 2.0 equiv.), *N*-(3-dimethylaminopropyl)-*N'*-ethylcarbodiimide hydrochloride (54 mg, 0.28 mmol, 2.0 equiv.) and 4-(4-dimethylamino)-pyridine (3.4 mg, 28 µmol, 20 mol%) was added and the reaction mixture was stirred at room temperature for 16 h. Removal of the solvent *in vacuo* and FC (Et_2O:MeOH = 33:1) afforded the desired indolizidine **8** (17 mg, 0.12 mmol, 87%) as a colorless oil.

$[\alpha]_D^{25}$= -4.4 (*c* 0.5 CH_2Cl_2). **^{1}H NMR (**500 MHz, $CDCl_3$, 299 K) δ = 3.64 – 3.55 (m, 1H), 3.50 – 3.34 (m, 2H), 2.43 (dd, *J*=18.0, 6.6 Hz, 1H), 2.27 (dddd, *J*=18.0, 11.8, 7.2, 1.0 Hz, 1H), 2.13 – 2.06 (m, 2H), 1.99 – 1.89 (m, 2H), 1.79 – 1.62 (m, 2H), 1.47 – 1.35 (m, 1H), 1.30 – 1.17 (m, 1H). **^{13}C NMR (**126 MHz, $CDCl_3$, 299 K) δ = 169.3, 59.4, 45.0, 33.7, 31.09, 29.2, 22.3, 21.3. **HRMS** (ESI) *m/z* = 162.0889 calcd. for $C_8H_{13}NONa$ $[M+Na]^+$, found: 162.0888. Spectroscopic data are in accordance with those described in the literature.[36]

2.6.5 (8*R*,8a*S*)-8-Methyl-8-(4,4,5,5-tetramethyl-1,3,2-dioxaborolan-2-yl) hexahydroindolizidine-5(1*H*)-one / (8*S*,8a*S*)-8-Methyl-8-(4,4,5,5-tetramethyl-1,3,2-dioxaborolan-2-yl)hexahydroindolizidin-5(1*H*)-one (11)

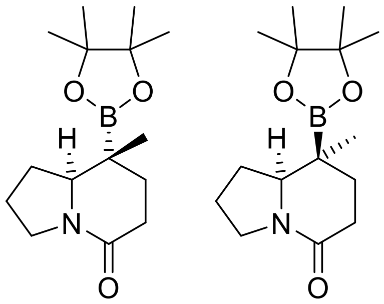

1.5 : 1.0

To a solution of boronic ester **10** (43 mg, 0.10 mmol, 1.0 equiv.) in CH_2Cl_2 (1.0 mL) was added trifluoroacetic acid (76 µL, 1.0 mmol, 10 equiv.) dropwise at 0 °C and the mixture was stirred at that temperature for 15 min. The solution was allowed to warm up to room temperature and stirred for another 2 h. The volatiles were removed *in vacuo* and the residue was taken up in CH_2Cl_2 (1.0 mL). After dropwise addition of triethylamine (0.28 mL, 2.0 mmol, 20 equiv.) at 0 °C, the mixture was allowed to warm to room temperature and stirred for 14 h. Removal of the volatiles and FC (CH_2Cl_2:MeOH = 100:1) afforded the desired boronic ester **11** (19 mg, 70 µmol, 68%) as an inseparable mixture of diastereomers (*dr* = 1.5:1.0) as a colorless oil. The assignment of the diastereomers is based on 1D NOESY NMR-experiments (see spectral data). **IR** (neat): ν (cm^{-1}) 2973, 2929, 2878, 1639, 1458, 1414, 1373, 1350, 1316, 1213, 1201, 1144, 1167, 1111, 1058, 1008, 967, 918, 853, 805, 703, 669, 682, 609. **^{1}H NMR (**500 MHz, $CDCl_3$, 299 K, both diastereomers) δ = 3.61 (dd, *J*=11.3 Hz, 5.3 Hz, 1H, CH_{major}), 3.57 – 3.36 (m, 2H), 3.06 (dd, *J*=10.0, 6.2 Hz, 1H, CH_{minor}), 2.53 – 2.28 (m, 2H), 2.00 – 1.63 (m, 5H), 1.55 – 1.34 (m, 1H), 1.23 – 1.20 (m, 12H, 4x$CH_{3\ major}$), 1.17 (s, 12H, 4x$CH_{3\ minor}$), 1.00 (s, 3H, $CH_{3\ minor}$), 0.85 (s, 3H, $CH_{3\ major}$). **^{13}C NMR** (126 MHz, $CDCl_3$, 299 K, both diastereomers) δ = 169.5, 169.0, 83.6 (2x), 83.4 (2x), 67.6, 63.6, 45.9, 45.4, 34.0, 30.9, 30.1, 30.0, 29.0, 27.0, 24.8 (2x), 24.8 (2x), 24.8 (2x), 24.7 (2x), 22.7, 22.3, 21.8 , 12.1. The signal of the α-B-carbon was not observed. **^{11}B NMR (**96 MHz, $CDCl_3$, 299 K, both diastereomers) δ = 33.6. **HRMS** (ESI) *m/z* = 302.1898 calcd. for $C_{15}H_{26}BNO_3Na$ $[M+Na]^+$, found: 302.1898.

2.6.6 (8*R*,8a*S*)-8-Methylhexahydroindolizine-5(1*H*)-one / (8*S*,8a*S*)-8-Methylhexahydroindolizine-5(1*H*)-one (12)

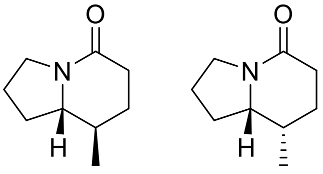

5 : 1

The reaction was performed according to a modified **GP4.** To a solution of 1-bromo-3,5-bis(trifluoromethyl)benzene (24 µL, 014 mmol, 1.4 equiv.) in THF (1.0 mL) was added *n*-BuLi (1.6 M, hexane, 90 µL, 014 mmol, 1.4 equiv.) at -78 °C over 5 min and the mixture was stirred at that temperature for 1 h. Subsequently, boronic ester **11** (28 mg, 0.10 mmol, 1.0 equiv.) in THF (1.0 mL) was added at -78 °C and the reaction was stirred at that temperature for 1 h. The reaction mixture was allowed to warm to room temperature and stirred for 1 h. The solvent was removed *in vacuo* and the residue was taken up in MeOH/acetone (1:1, 1.0 mL). After the addition of thiophenol (14 *µ*L, 0.14 mmol, 1.4 equiv.) and $Ir(dFCF_3ppy)_2(dtbbpy)PF_6$ (2.5 mg, 2.5 µmol, 2.5 mol%)

the reaction mixture was stirred under irradiation with a blue LED for 24 h. Removal of the solvent and FC (Et_2O:MeOH = 100:1→25:1) afforded the desired indolizidine **12** (9.0 mg, 59 µmol, 59%) as an inseparable mixture of diastereomers (dr = 5:1) as a colorless oil. The assignment of the diastereomers is based on 1D NOESY NMR-experiments (see spectral data) and reference spectral data from the literature.[37,38] **^{1}H NMR (**600 MHz, $CDCl_3$, 299 K, both diastereomers) δ = 3.61 – 3.53 (m, 1H), 3.52 – 3.44 (m, 1H), 3.04 – 2.95 (m, 1H), 2.51 – 2.30 (m, 2H), 2.20 – 2.11 (m, 1H), 1.98 – 1.91 (m, 1H), 1.89 – 1.80 (m, 1H), 1.78 – 1.68 (m, 1H), 1.63 – 1.33 (m, 3H), 1.01 (d, *J*=5.9 Hz, 3H, CH_{3major}), 0.87 (d, *J*=7.0 Hz, 3H, CH_{3minor}).**^{13}C NMR (**151 MHz, $CDCl_3$, 299 K, both diastereomers) δ = 169.4 (C_{major}), 169.2 (C_{minor}), 65.4 (CH_{major}), 62.1 (CH_{minor}), 45.5 (CH_{2major}), 45.5 (CH_{2minor}), 35.5 (CH_{major}), 32.4 (CH_{2major}), 31.6 (CH_{2major}), 30.2 (CH_{2major}), 29.3 (CH_{2minor}), 28.5 (CH_{minor}), 28.3 (CH_{2minor}), 27.2 (CH_{2minor}), 22.4 (CH_{2minor}), 22.3 (CH_{2minor}), 18.3 (CH_{3major}), 11.4 (CH_{3minor}). **HRMS** (ESI) *m/z* = 176.1046 calcd. for $C_9H_{15}NONa$ $[M+Na]^+$, found: 176.1030.

2.7 Mechanistic Studies

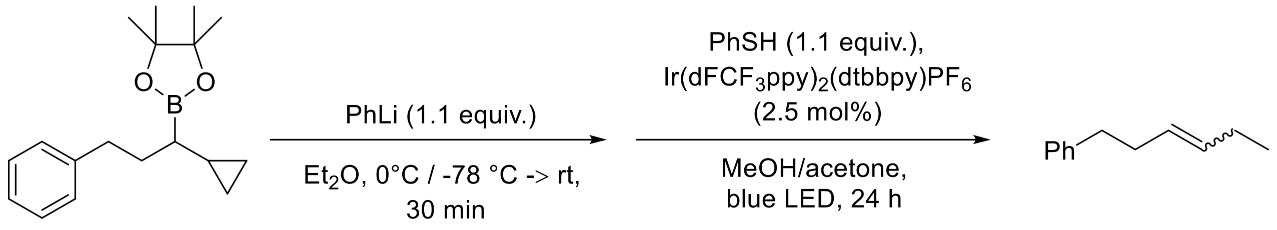

1o **2o**

The radical probe experiment was performed according to **GP4** with boronic ester **1o** (57 mg, 0.20 mmol, 1.0 equiv.), phenyllithium (1.9 M, Bu_2O, 0.12 mL, 0.22 mmol, 1.1 equiv.), thiophenol (23 µL, 0.22 mmol, 1.1 equiv) and $Ir(dFCF_3ppy)_2(dtbbpy)PF_6$ (5.0 mg, 5.0 µmol, 2.5 mol%) in acetone/MeOH (1:1, 2.0 mL). FC (P) afforded hex-3-en-1-ylbenzene (**2o**) (27 mg, 0.17 mmol, 84%) as an inseparable isomeric mixture (*E/Z* = 4:1) as a colorless oil. **^{1}H NMR** (300 MHz, $CDCl_3$, 299 K, both isomers) δ = 7.34 – 7.26 (m, 2H), 7.25 – 7.17 (m, 3H), 5.58 – 5.46 (m, 1.6H), 5.46 – 5.38 (m, 0.4H), 2.75 – 2.63 (m, 2H), 2.43 – 2.27 (m, 2H), 2.12 – 1.96 (m, 2H), 0.99 (t, *J*=7.5 Hz, 2.4H), 0.93 (t, *J*=7.5 Hz, 0.6H). **^{13}C NMR** (75 MHz, $CDCl_3$, 299 K, both isomers) δ = 142.4 (C_{major}), 142.3 (C_{minor}), 132.8 (CH_{major}), 132.5 (CH_{minor}), 128.6 (4xCH_{both}), 128.5 (CH_{major}), 128.4 (4xCH_{both}), 128.2 (CH_{minor}), 125.9 (CH_{minor}), 125.8 (CH_{major}), 36.3 (CH_{2major}), 36.2 (CH_{2minor}), 34.6 (CH_{2major}), 29.2 (CH_{2minor}), 25.7 (CH_{2major}), 20.7 (CH_{2minor}), 14.4 (CH_{3minor}), 14.0 (CH_{3major}). Spectroscopic data are in accordance with those described in the literature.[39]

References

[1] R. Soundararajan, D. S. Matteson, “Hydroboration with boron halides and trialkylsilanes,” DOI 10.1021/jo00295a004can be found under https://pubs.acs.org/doi/abs/10.1021/jo00295a004, **2002**.
[2] A. Bonet, M. Odachowski, D. Leonori, S. Essafi, V. K. Aggarwal, *Nat. Chem.* **2014**, *6*, 584–589.
[3] Y. Cheng, C. Mück-Lichtenfeld, A. Studer, *Angew. Chem. Int. Ed.* **2018**, *57*, 16832–16836.

[4] N. Stuhr-Hansen, *Heteroat. Chem.* **2018**, *29*, e21438.
[5] A. Cabré, G. Sciortino, G. Ujaque, X. Verdaguer, A. Lledós, A. Riera, *Org. Lett.* **2018**, *20*, 5747–5751.
[6] J. Chen, T. Xi, X. Ren, B. Cheng, J. Guo, Z. Lu, *Org. Chem. Front.* **2014**, *1*, 1306–1309.
[7] A. Edwards, P. Ryabchuk, A. Barkov, M. Rubina, M. Rubin, *Tetrahedron Asymmetry* **2014**, *25*, 1537–1549.
[8] R. Larouche-Gauthier, T. G. Elford, V. K. Aggarwal, *J. Am. Chem. Soc.* **2011**, *133*, 16794–16797.
[9] J. L. Stymiest, G. Dutheuil, A. Mahmood, V. K. Aggarwal, *Angew. Chem. Int. Ed.* **2007**, *46*, 7491–7494.
[10] B. Chen, P. Cao, Y. Liao, M. Wang, J. Liao, *Org. Lett.* **2018**, *20*, 1346–1349.
[11] K. Yang, Q. Song, *Green Chem.* **2016**, *18*, 932–936.
[12] Y. Wen, J. Xie, C. Deng, C. Li, *J. Org. Chem.* **2015**, *80*, 4142–4147.
[13] S. Kisan, V. Krishnakumar, C. Gunanathan, *ACS Catal.* **2017**, *7*, 5950–5954.
[14] A. Varela, L. K. B. Garve, D. Leonori, V. K. Aggarwal, *Angew. Chem. Int. Ed.* **2017**, *56*, 2127–2131.
[15] L. Wen, F. Cheng, H. Li, S. Zhang, X. Hong, F. Meng, *Asian J. Org. Chem.* **2018**, *7*, 103–106.
[16] R. Corberán, N. W. Mszar, A. H. Hoveyda, *Angew. Chem. Int. Ed.* **2011**, *50*, 7079–7082.
[17] M. A. Reichle, B. Breit, *Angew. Chem. Int. Ed.* **2012**, *51*, 5730–5734.
[18] J. M. Brunel, *Tetrahedron* **2007**, *63*, 3899–3906.
[19] C.-T. Yang, Z.-Q. Zhang, Y.-C. Liu, L. Liu, *Angew. Chem. Int. Ed.* **2011**, *50*, 3904–3907.
[20] C. Lohre, T. Dröge, C. Wang, F. Glorius, *Chem. – Eur. J.* **2011**, *17*, 6052–6055.
[21] P. S. Engel, L. Pan, Y. Ying, L. B. Alemany, *J. Am. Chem. Soc.* **2001**, *123*, 3706–3715.
[22] F. Schoenebeck, J. A. Murphy, S. Zhou, Y. Uenoyama, Y. Miclo, T. Tuttle, *J. Am. Chem. Soc.* **2007**, *129*, 13368–13369.
[23] J. Le Bras, J. Muzart, *Tetrahedron* **2007**, *63*, 7942–7948.
[24] J. Barluenga, M. Tomás-Gamasa, F. Aznar, C. Valdés, *Nat. Chem.* **2009**, *1*, 494–499.
[25] M. E. Sloan, A. Staubitz, K. Lee, I. Manners, *Eur. J. Org. Chem.* **2011**, *2011*, 672–675.
[26] F.-X. Felpin, E. Fouquet, *Chem. – Eur. J.* **2010**, *16*, 12440–12445.
[27] S. C. Farmer, S. H. Berg, *Molecules* **2008**, *13*, 1345–1352.
[28] S. R. Gogoi, J. J. Boruah, G. Sengupta, G. Saikia, K. Ahmed, K. K. Bania, N. S. Islam, *Catal. Sci. Technol.* **2014**, *5*, 595–610.
[29] R. K. Dieter, S. Li, *J. Org. Chem.* **1997**, *62*, 7726–7735.
[30] A. L. Hansen, J.-P. Ebran, T. M. Gøgsig, T. Skrydstrup, *J. Org. Chem.* **2007**, *72*, 6464–6472.
[31] M. Okajima, K. Soga, T. Nokami, S. Suga, J. Yoshida, *Org. Lett.* **2006**, *8*, 5005–5007.
[32] B. L. H. Taylor, E. C. Swift, J. D. Waetzig, E. R. Jarvo, *J. Am. Chem. Soc.* **2011**, *133*, 389–391.
[33] J. J. Eisch, J. E. Galle, *J. Organomet. Chem.* **1988**, *341*, 293–313.
[34] A. Joshi-Pangu, M. Ganesh, M. R. Biscoe, *Org. Lett.* **2011**, *13*, 1218–1221.
[35] C. Gerleve, M. Kischkewitz, A. Studer, *Angew. Chem. Int. Ed.* **2018**, *57*, 2441–2444.
[36] S. H. Park, H. J. Kang, S. Ko, S. Park, S. Chang, *Tetrahedron Asymmetry* **2001**, *12*, 2621–2624.
[37] H. Wu, M. Yu, Y. Zhang, G. Zhao, *Chin. J. Chem.* **2009**, *27*, 183–188.
[38] F. Abels, C. Lindemann, E. Koch, C. Schneider, *Org. Lett.* **2012**, *14*, 5972–5975.
[39] R. Rasappan, V. K. Aggarwal, *Nat. Chem.* **2014**, *6*, 810–814.

3 Appendix

3.1 Chiral HPLC

3.1.1 2-(2-(benzo[d][1,3]dioxol-5-yl)pentyl)-4,4,5,5-tetramethyl-1,3,2-dioxaborolane

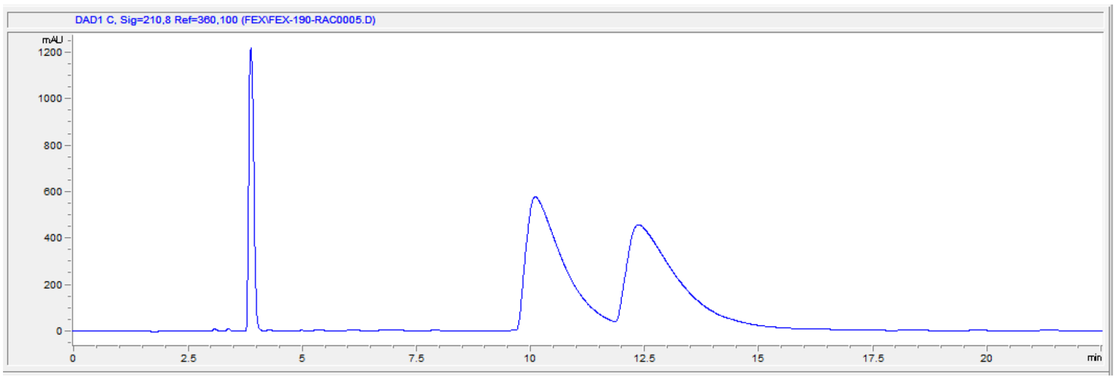

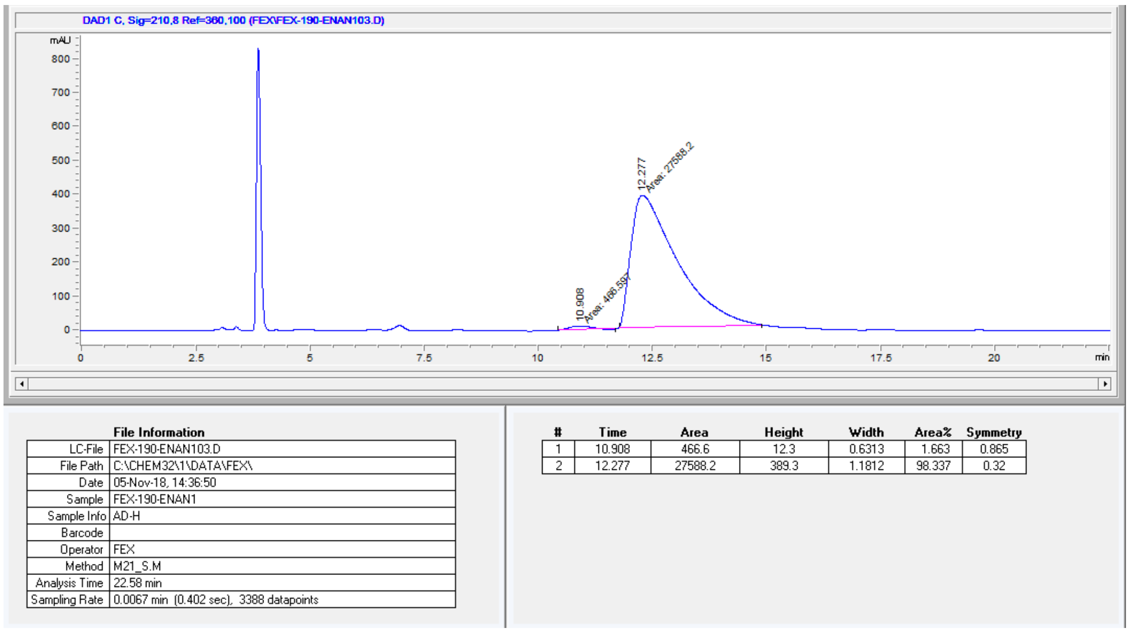

File Information

LC-File	FEX-190-ENAN103.D
File Path	C:\CHEM32\1\DATA\FEX\
Date	05-Nov-18, 14:36:50
Sample	FEX-190-ENAN1
Sample Info	AD-H
Barcode	
Operator	FEX
Method	M21_S.M
Analysis Time	22.58 min
Sampling Rate	0.0067 min (0.402 sec), 3388 datapoints

#	Time	Area	Height	Width	Area%	Symmetry
1	10.908	466.6	12.3	0.6313	1.663	0.865
2	12.277	27588.2	389.3	1.1812	98.337	0.32

3.1.2 (*S*)-1-Methoxy-4-(pentan-2-yl)benzene (2k)

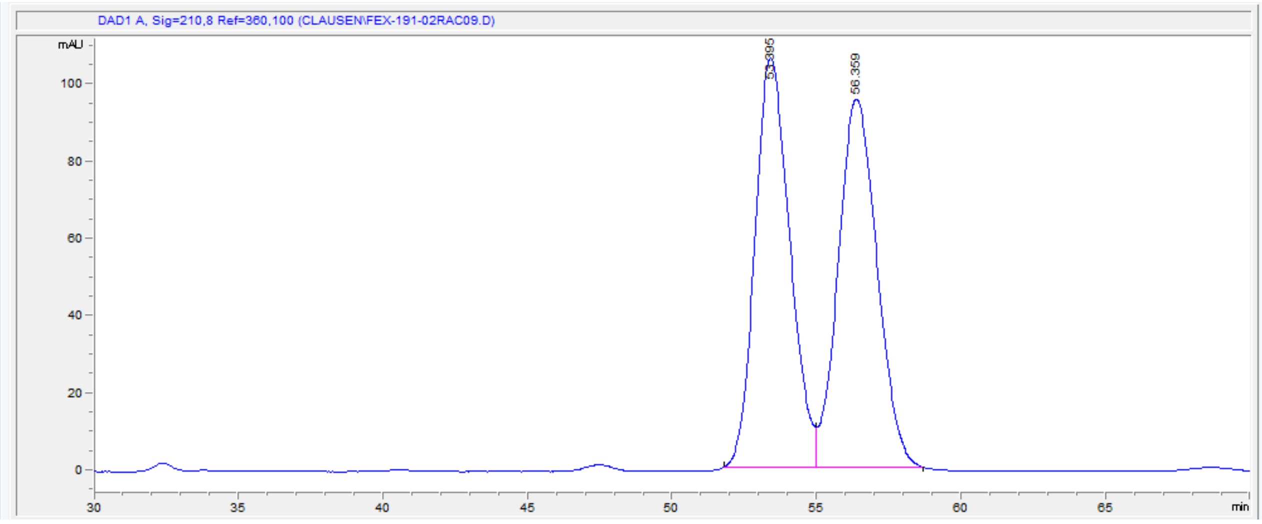

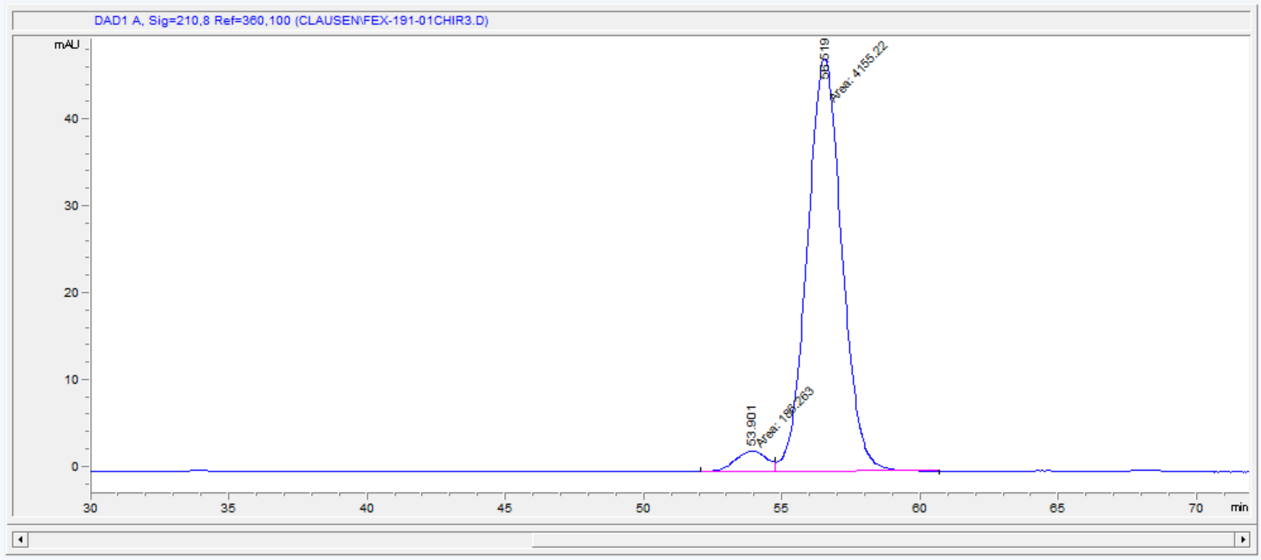

File Information

LC-File	FEX-191-01CHIR3.D
File Path	C:\CHEM32\1\DATA\CLAUSEN\
Date	13-Nov-18, 09:23:39
Sample	FEX-191-01chir
Sample Info	AD-RH
	sample in ACN
Barcode	
Operator	prekel
Method	RP CHIRAL_40-60_T30.M
Analysis Time	71.893 min
Sampling Rate	0.0067 min (0.402 sec), 10785 datapoints

#	Time	Area	Height	Width	Area%	Symmetry
1	53.901	186.3	2.4	1.3126	4.290	1.133
2	56.519	4155.2	47.5	1.4573	95.710	0.958

3.2 NMR Spectra

3.2.1 Starting materials

Bis(4-methoxy)-1,1-ethylene **^{1}H NMR**

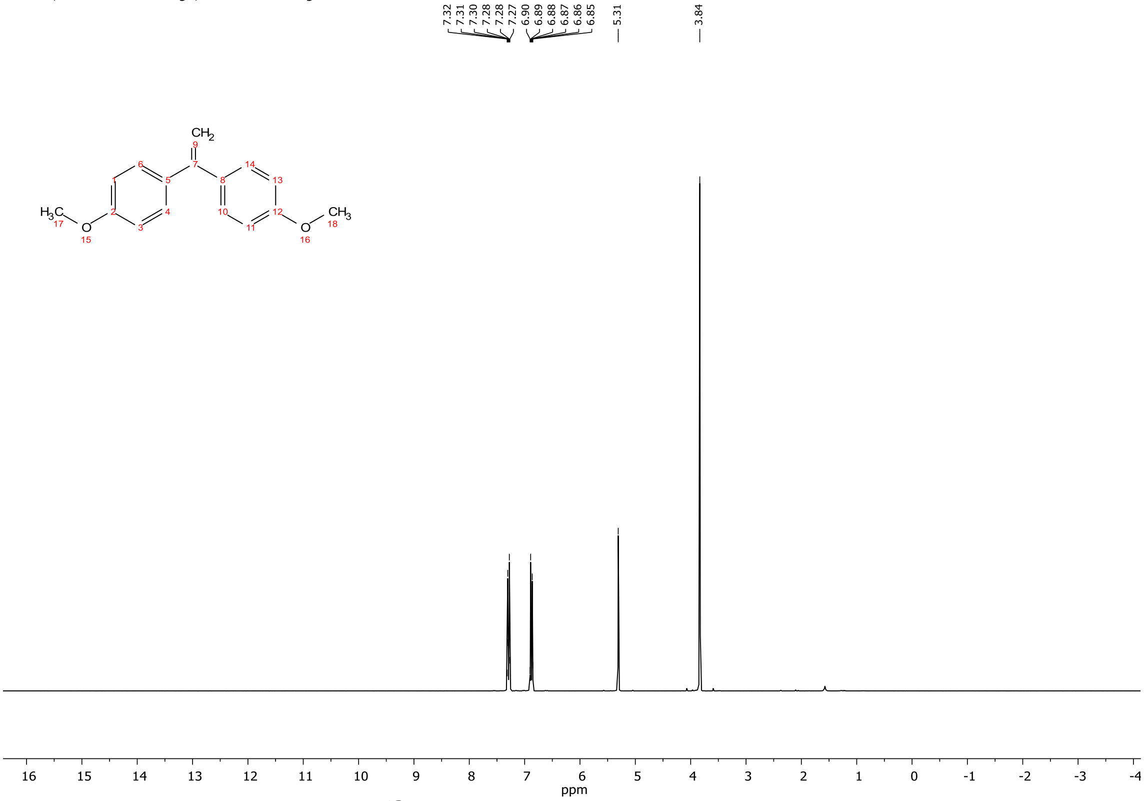

Bis(4-methoxy)-1,1-ethylene ^{13}H NMR

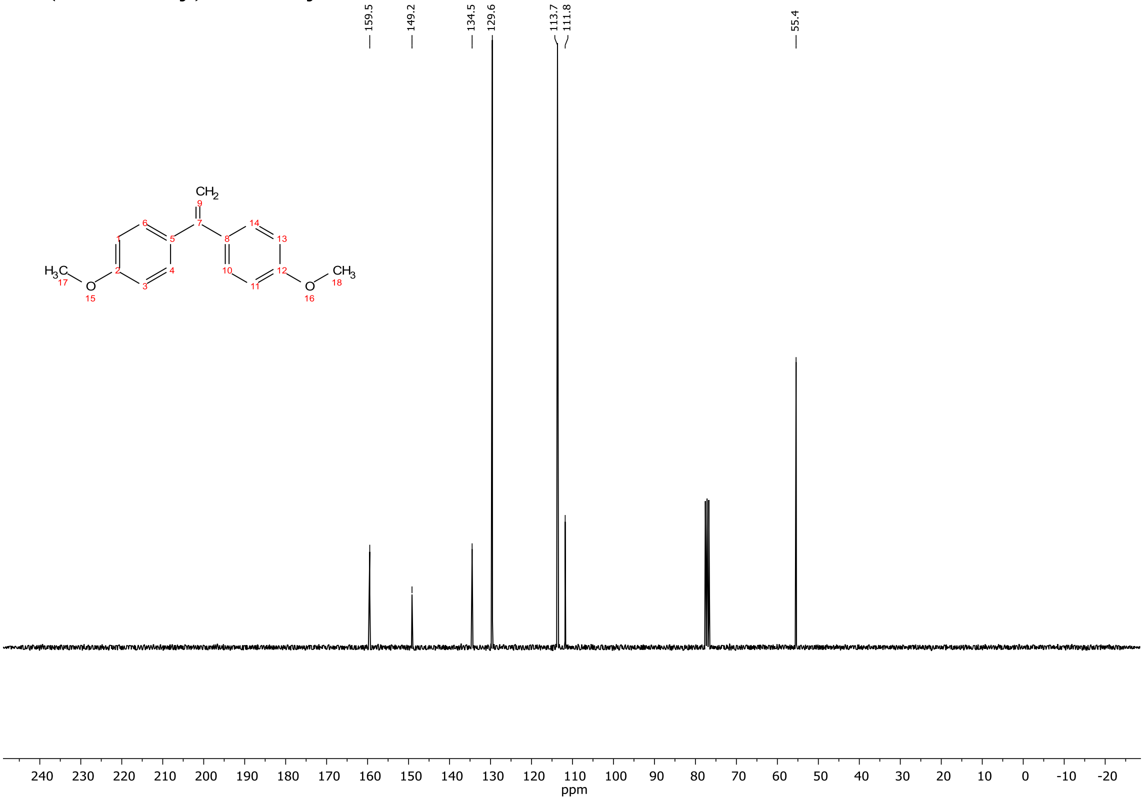

1-Methoxy-2-(prop-1-en-2-yl)benzene **^{1}H NMR**

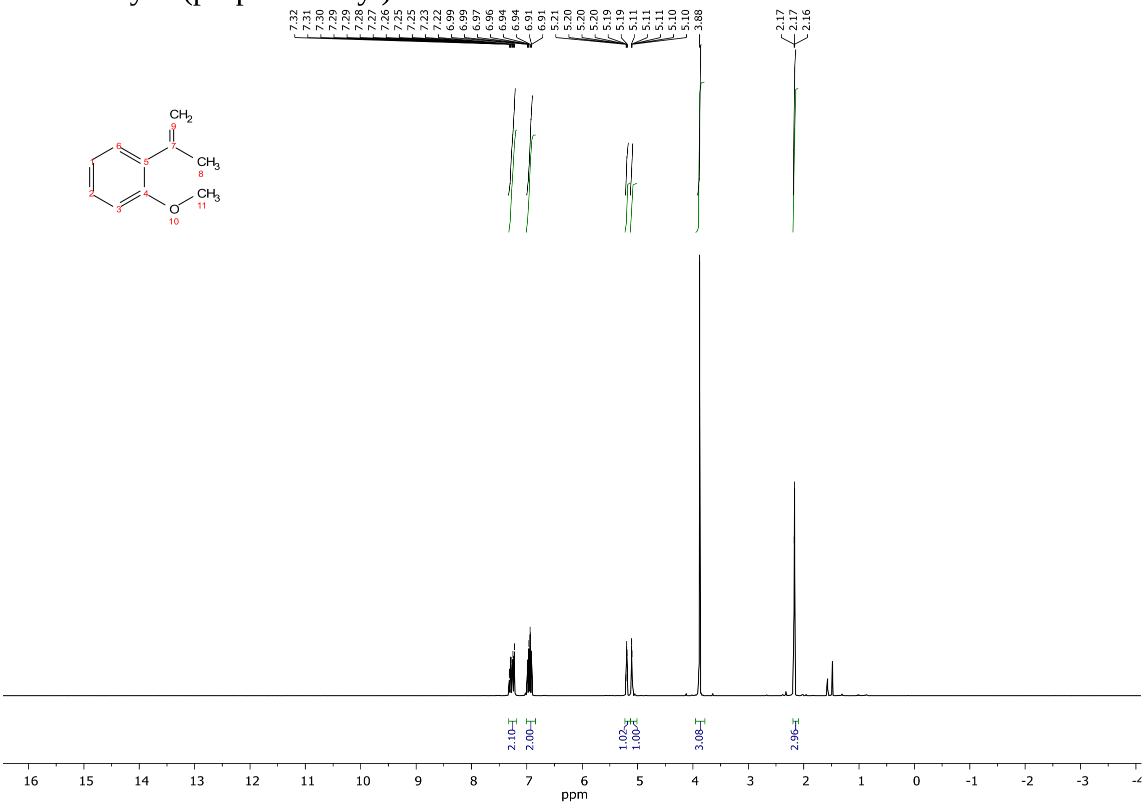

1-Methoxy-2-(prop-1-en-2-yl)benzene **^{13}C NMR**

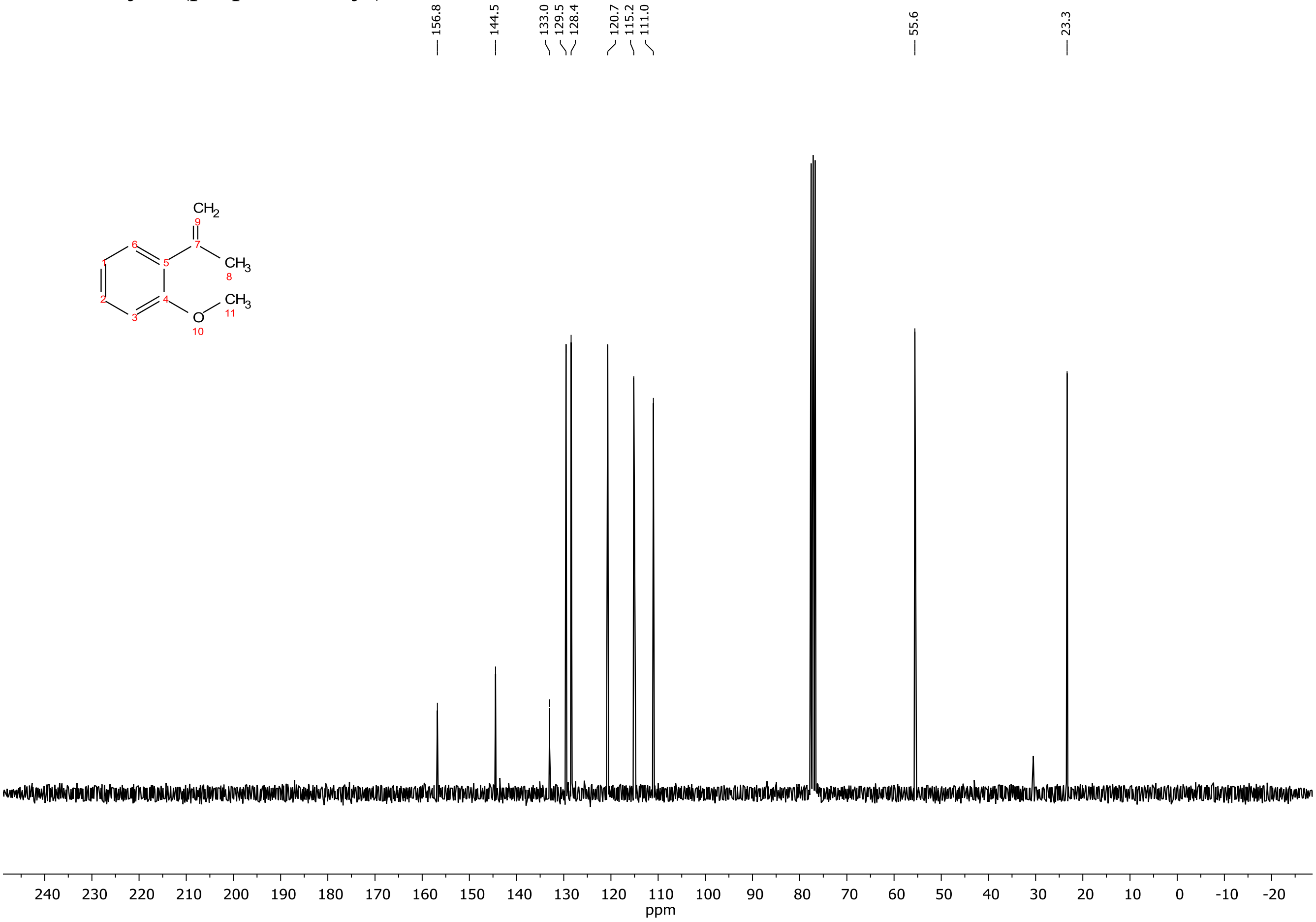

5-(Pent-1-en-2-yl)benzo[d][1,3]dioxole ^{1}H NMR

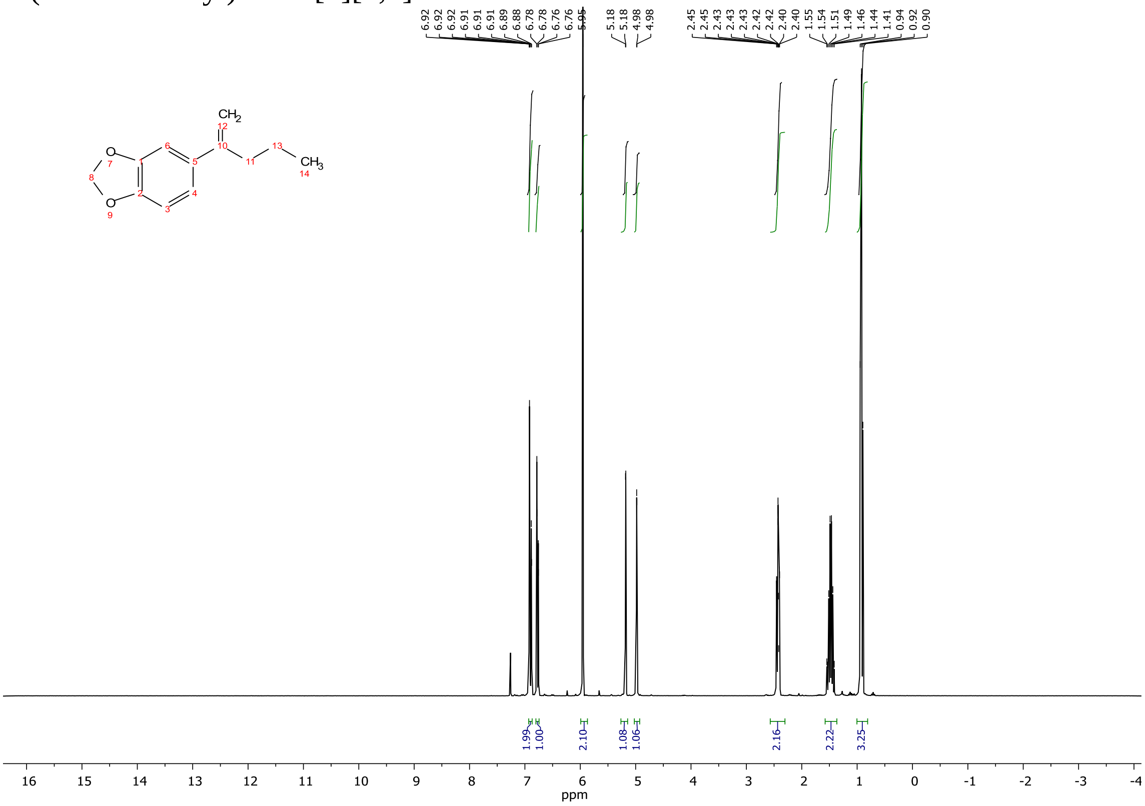

5-(Pent-1-en-2-yl)benzo[d][1,3]dioxole ^{13}C NMR

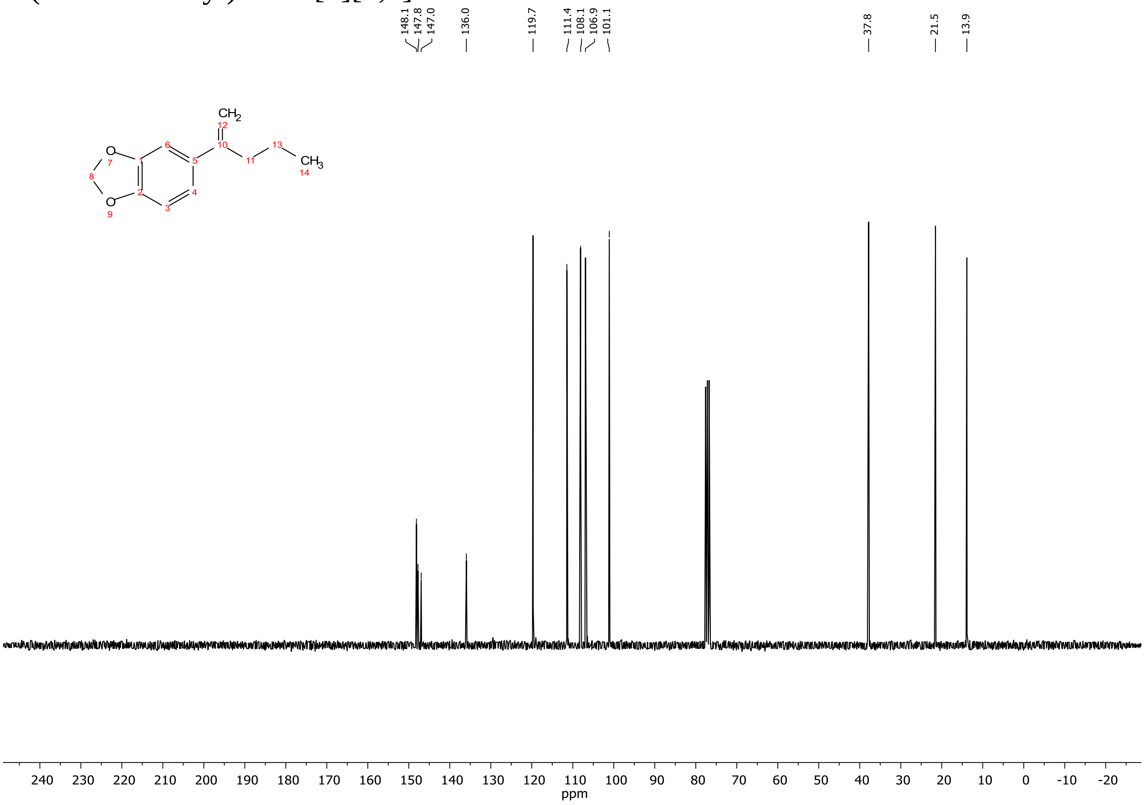

1,2-Dimethyl-4-(prop-1-en-2-yl)benzene **^{1}H NMR**

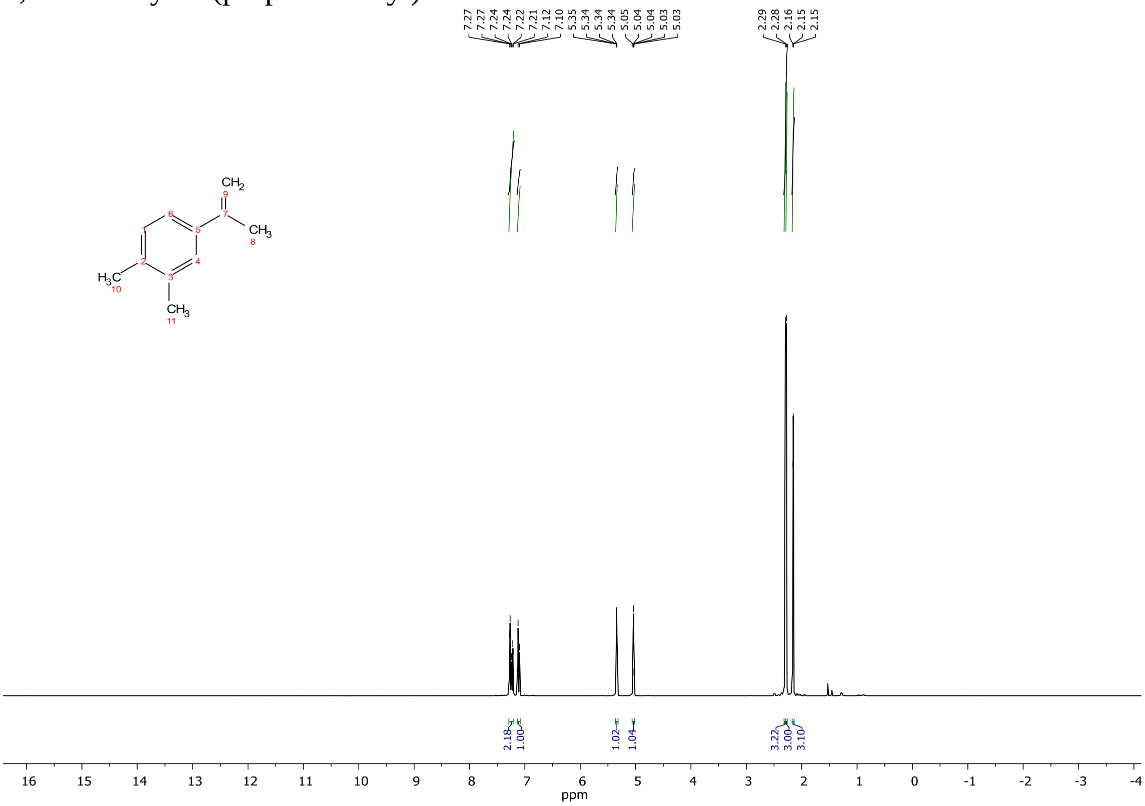

1,2-Dimethyl-4-(prop-1-en-2-yl)benzene **^{13}C NMR**

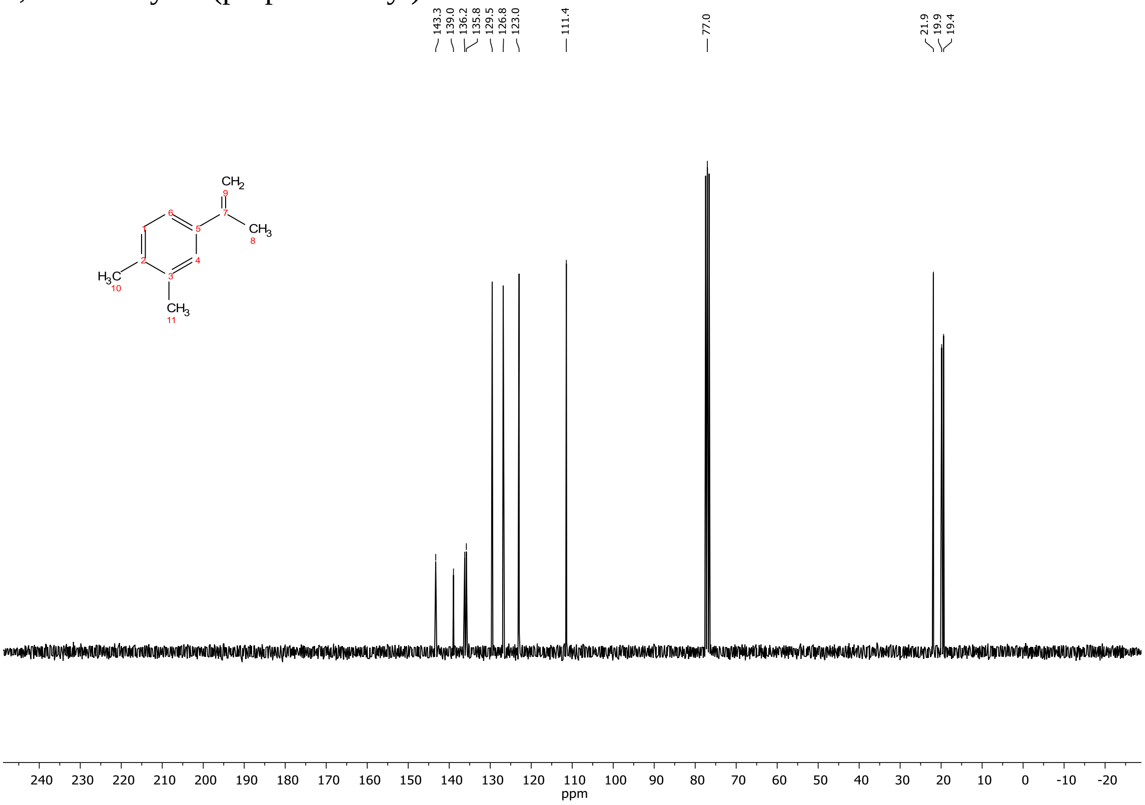

(*R*)-2-(6-Bromopyridin-2-yl)-4-isopropyl-4,5-dihydrooxazole ^{1}**H NMR**

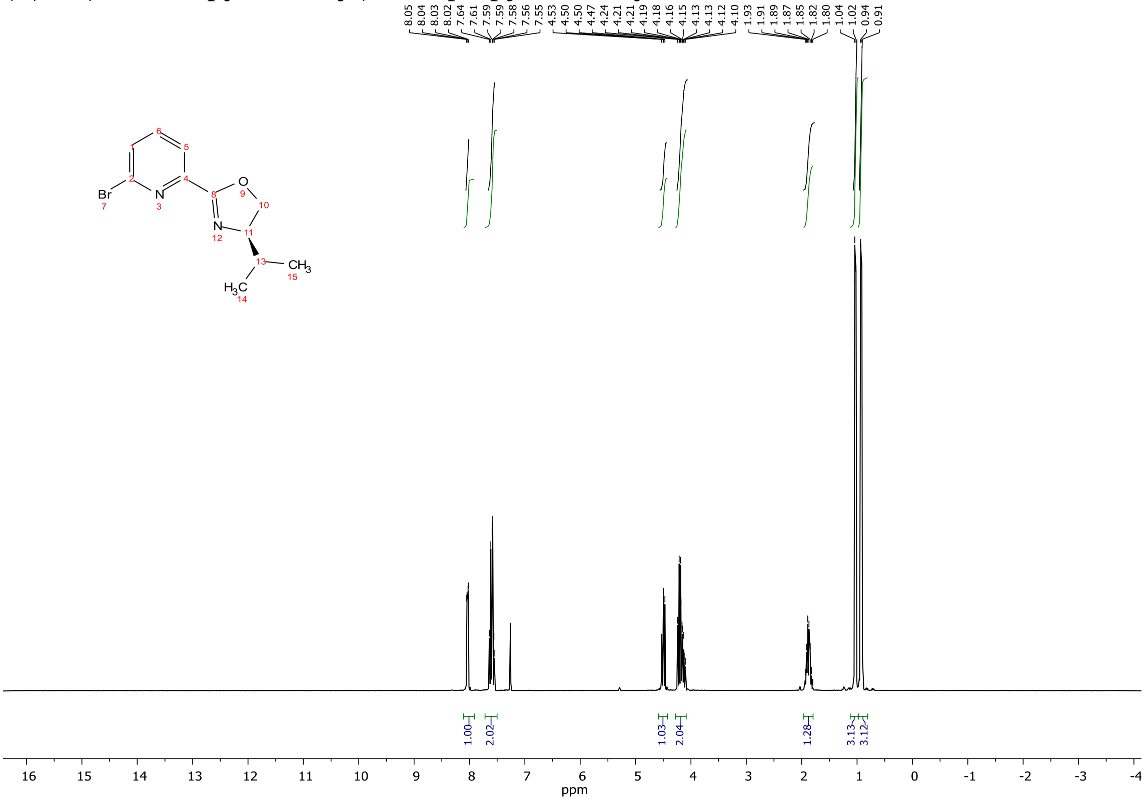

(*R*)-2-(6-Bromopyridin-2-yl)-4-isopropyl-4,5-dihydrooxazole ^{13}**C NMR**

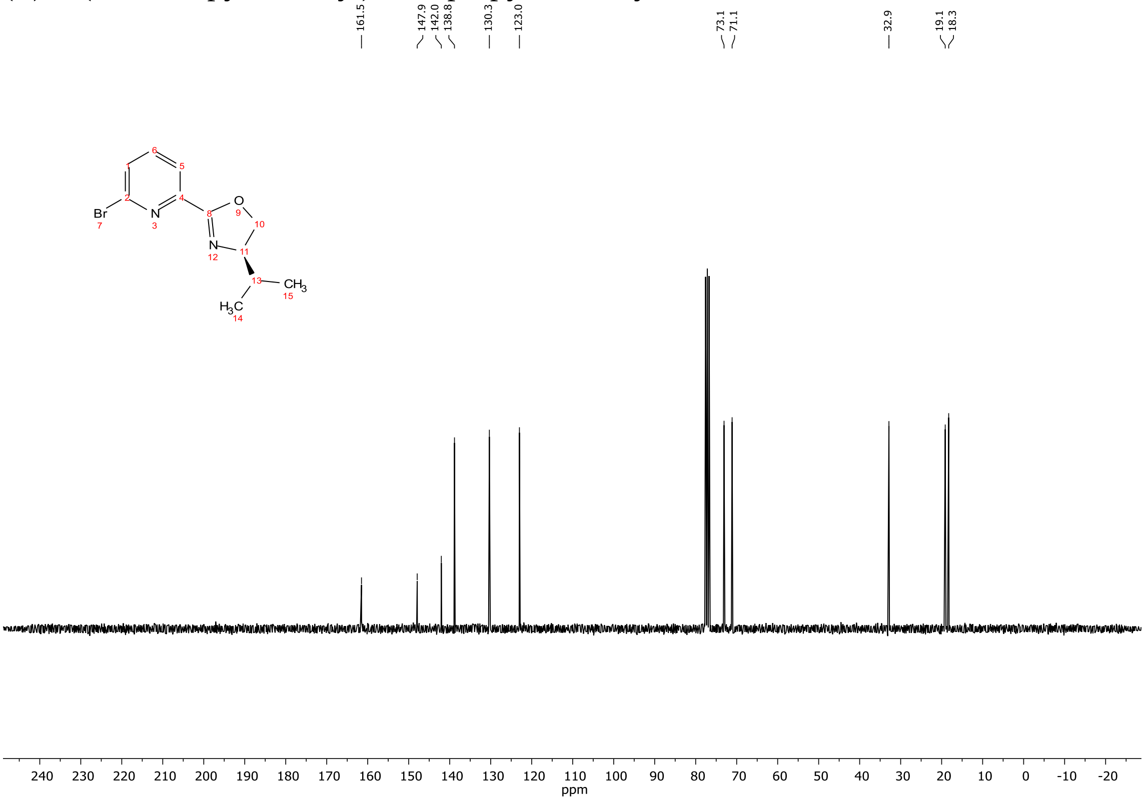

(*R*)-1-(6-(4-Isopropyl-4,5-dihydrooxazol-2-yl)pyridin-2-yl)ethan-1-one **^{1}H NMR**

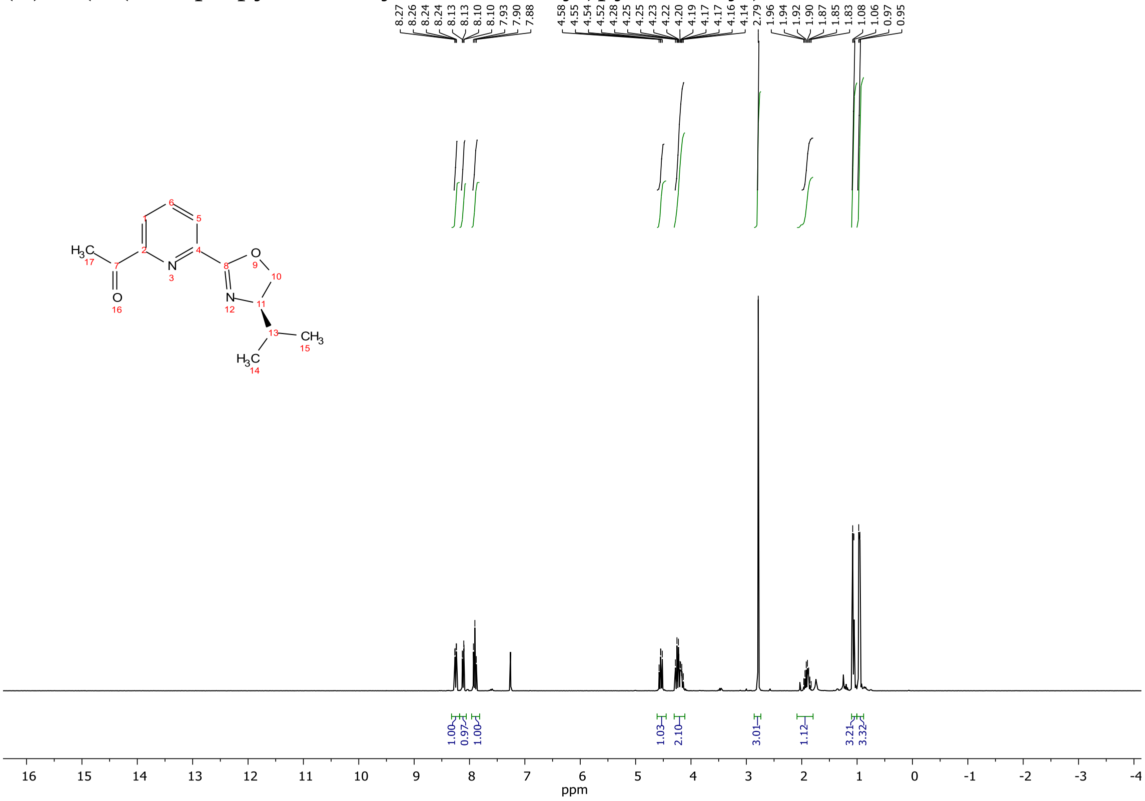

(*R*)-1-(6-(4-Isopropyl-4,5-dihydrooxazol-2-yl)pyridin-2-yl)ethan-1-one **^{13}C NMR**

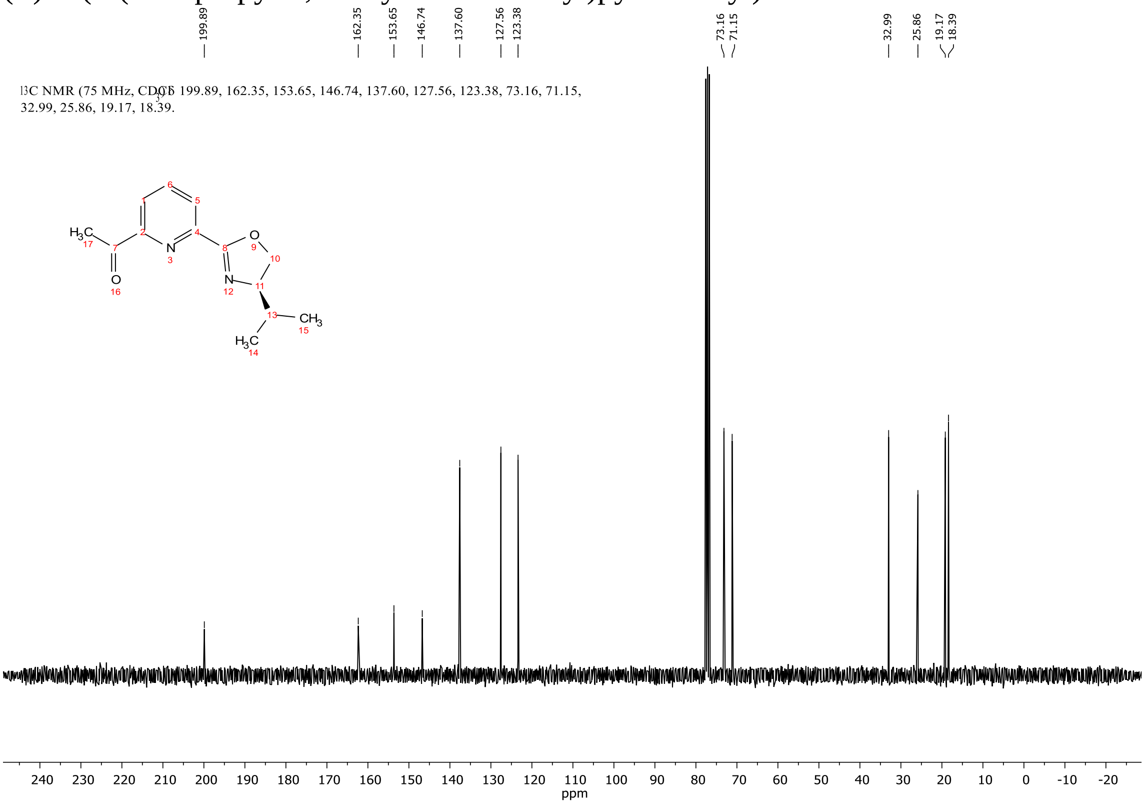

(*R*,*E*)-N-(2,6-Diisopropylphenyl)-1-(6-(4-isopropyl-4,5-dihydrooxazol-2-yl)pyridin-2-yl)ethan-1-imine

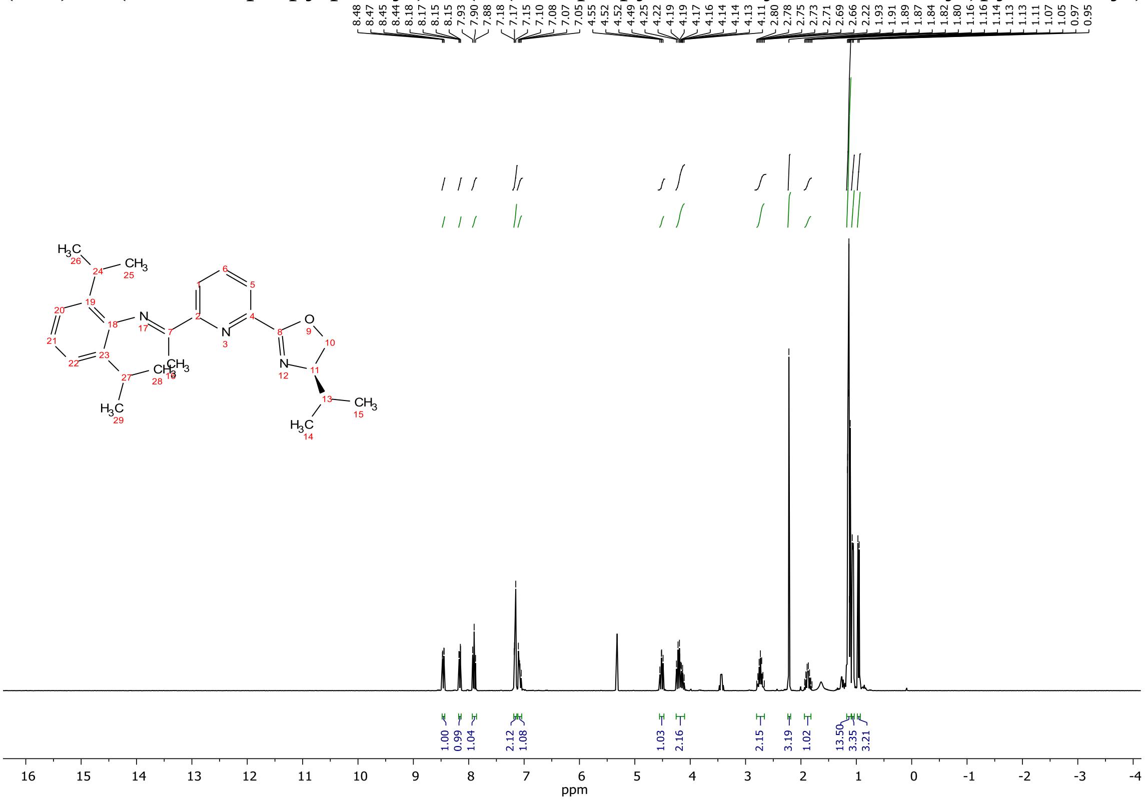

(*R*,*E*)-N-(2,6-Diisopropylphenyl)-1-(6-(4-isopropyl-4,5-dihydrooxazol-2-yl)pyridin-2-yl)ethan-1-imine

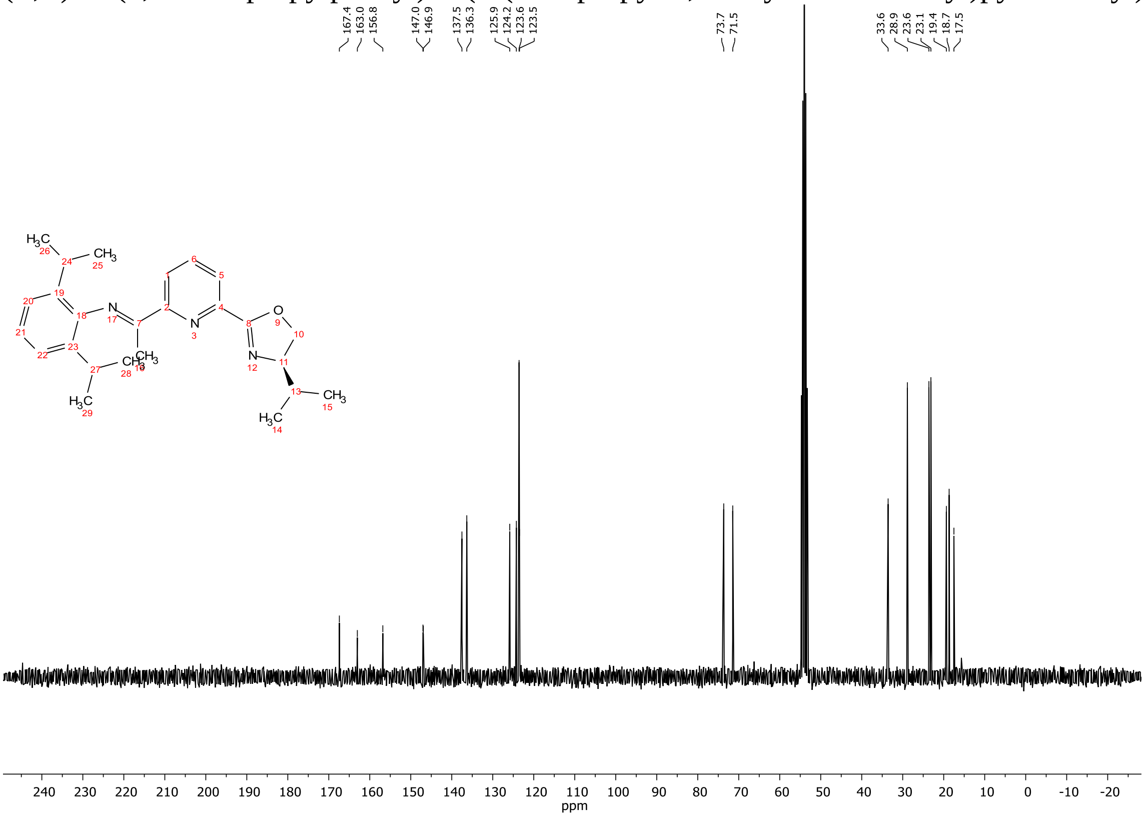

3-Phenylpropyl diisopropylcarbamate (**CARB1**) ^{1}**H NMR**

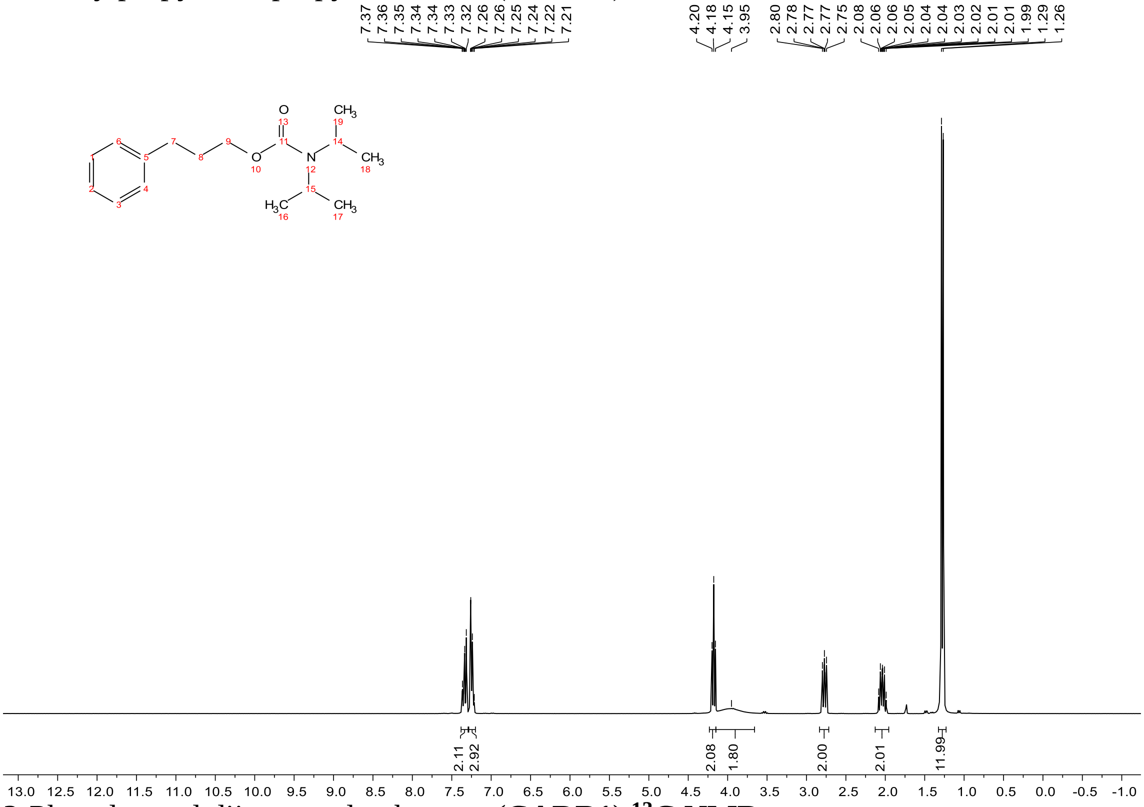

3-Phenylpropyl diisopropylcarbamate (**CARB1**) ^{13}**C NMR**

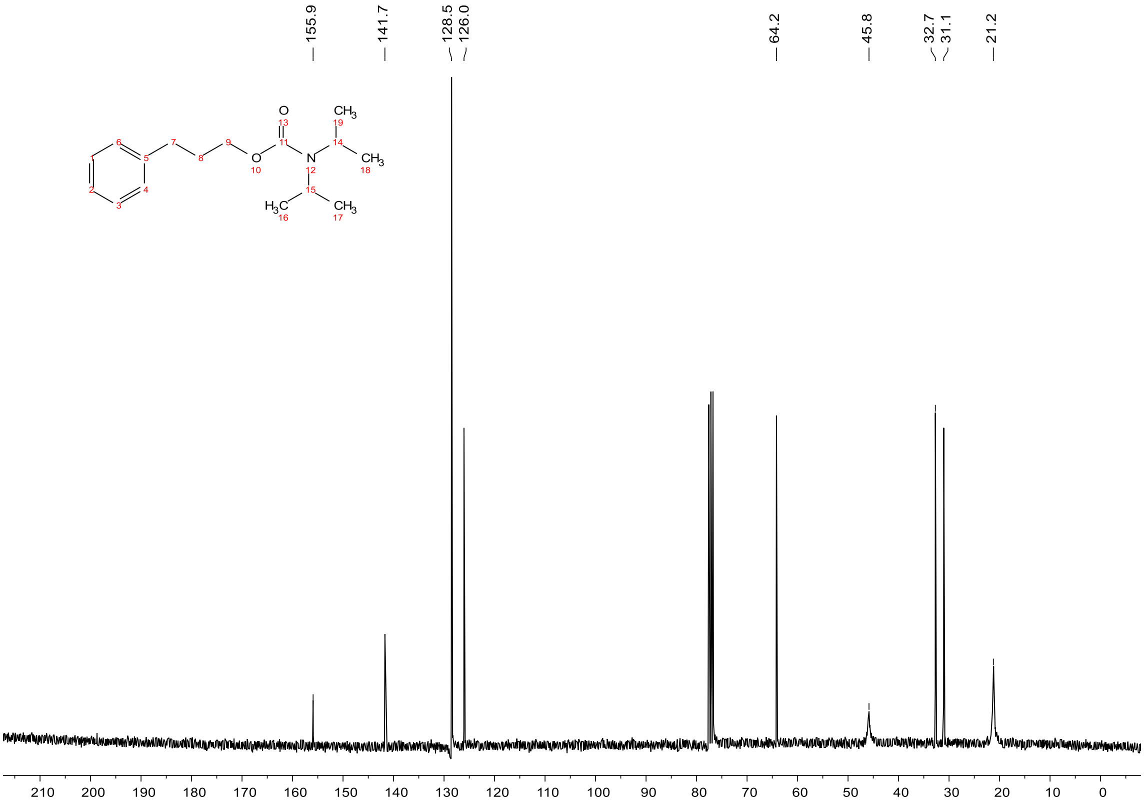

3.2.2 Boronic esters

2-(3-(3,4-Dimethoxyphenyl)propyl)-4,4,5,5-tetramethyl-1,3,2-dioxaborolane (**1c**) **^{1}H NMR**

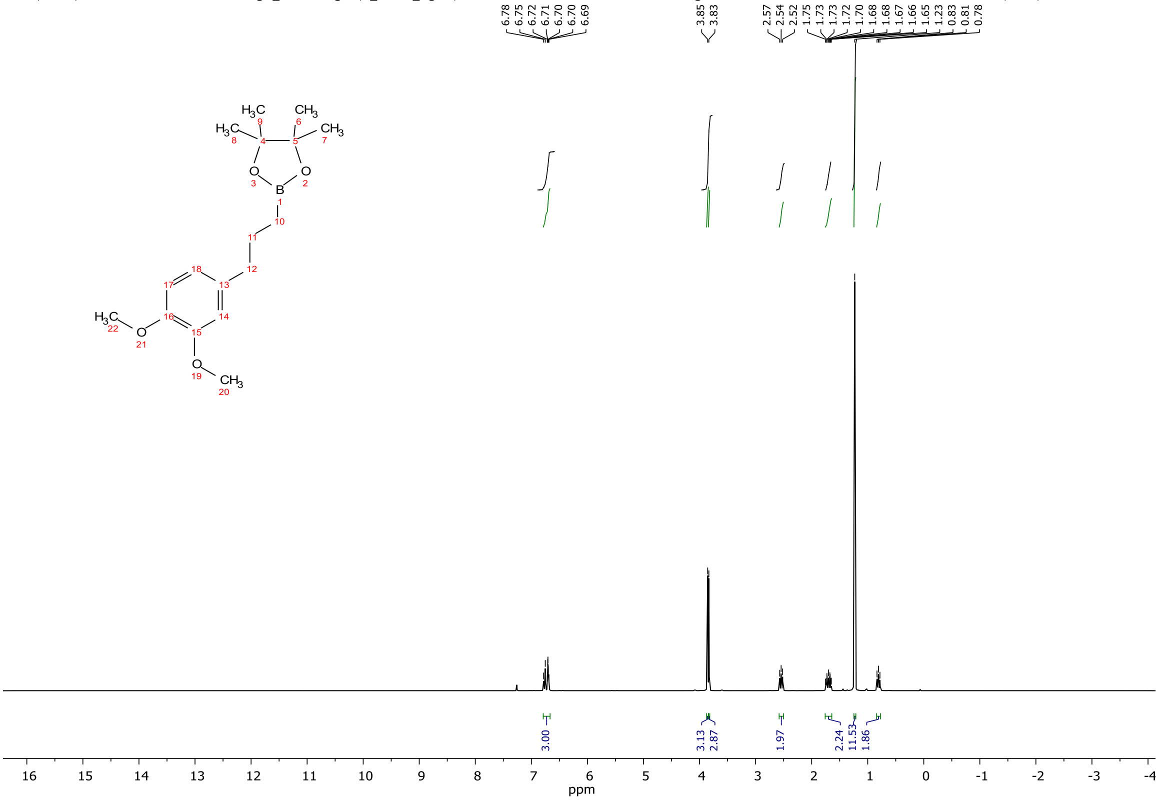

2-(3-(3,4-Dimethoxyphenyl)propyl)-4,4,5,5-tetramethyl-1,3,2-dioxaborolane (**1c**) **^{13}C NMR**

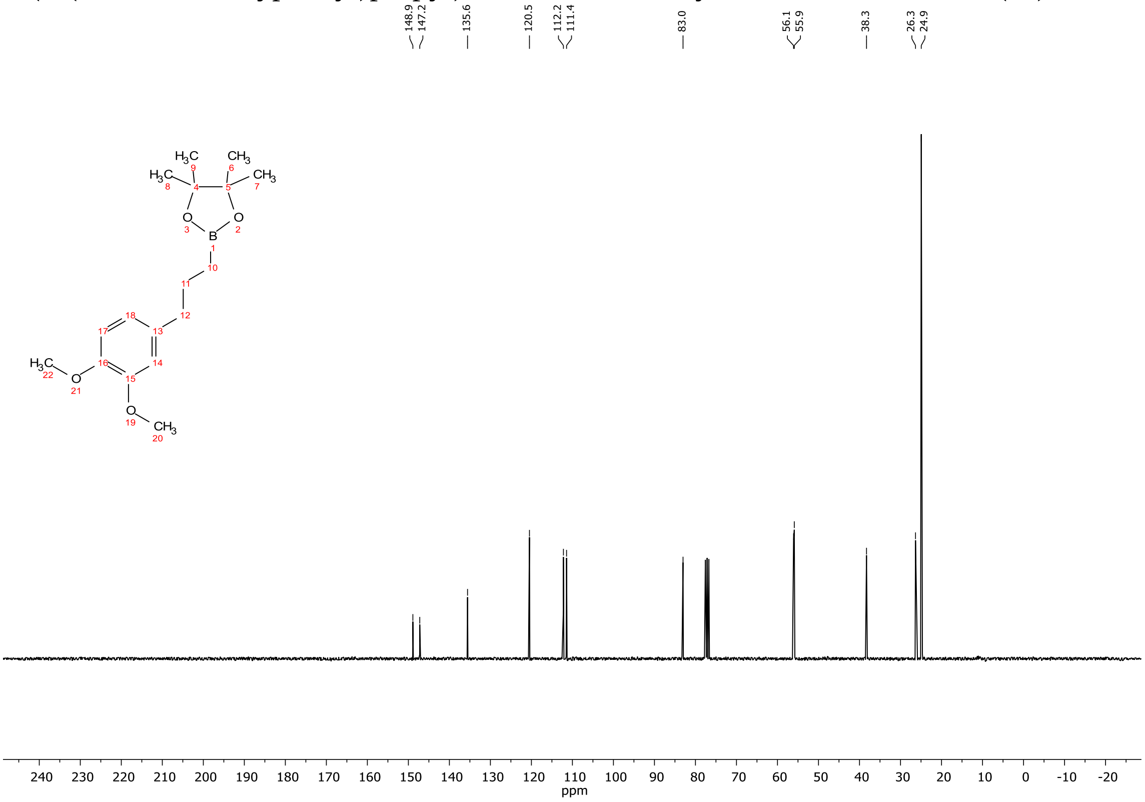

2-(2-([1,1’-Biphenyl]-4-yl)-propyl)-4,4,5,5-tetramethyl-1,3,2-dioxaborolan (**1d, 14c**) **^{1}H NMR**

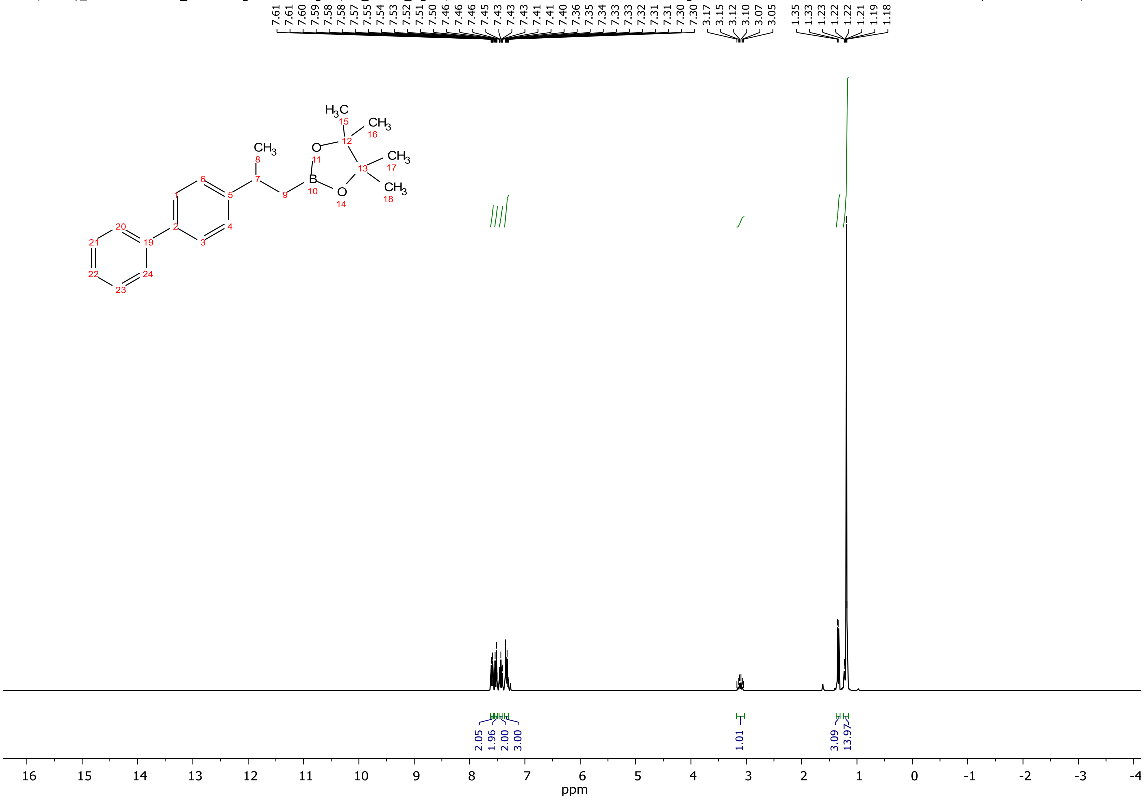

2-(2-([1,1’-Biphenyl]-4-yl)-propyl)-4,4,5,5-tetramethyl-1,3,2-dioxaborolan (**1d, 14c**) **^{13}C NMR**

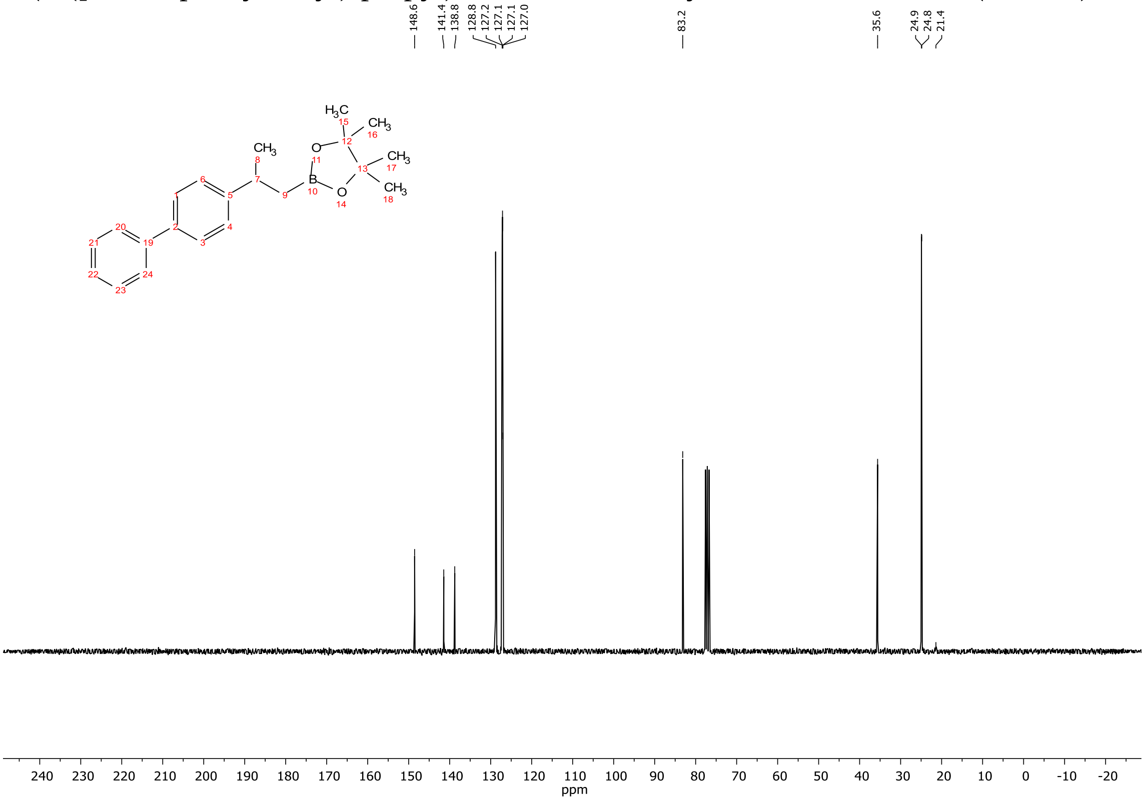

2-(2-(4-(*tert*-Butyl)phenyl)propyl)-4,4,5,5-tetramethyl-1,3,2-dioxaborolan (**1e**) **^{1}H NMR**

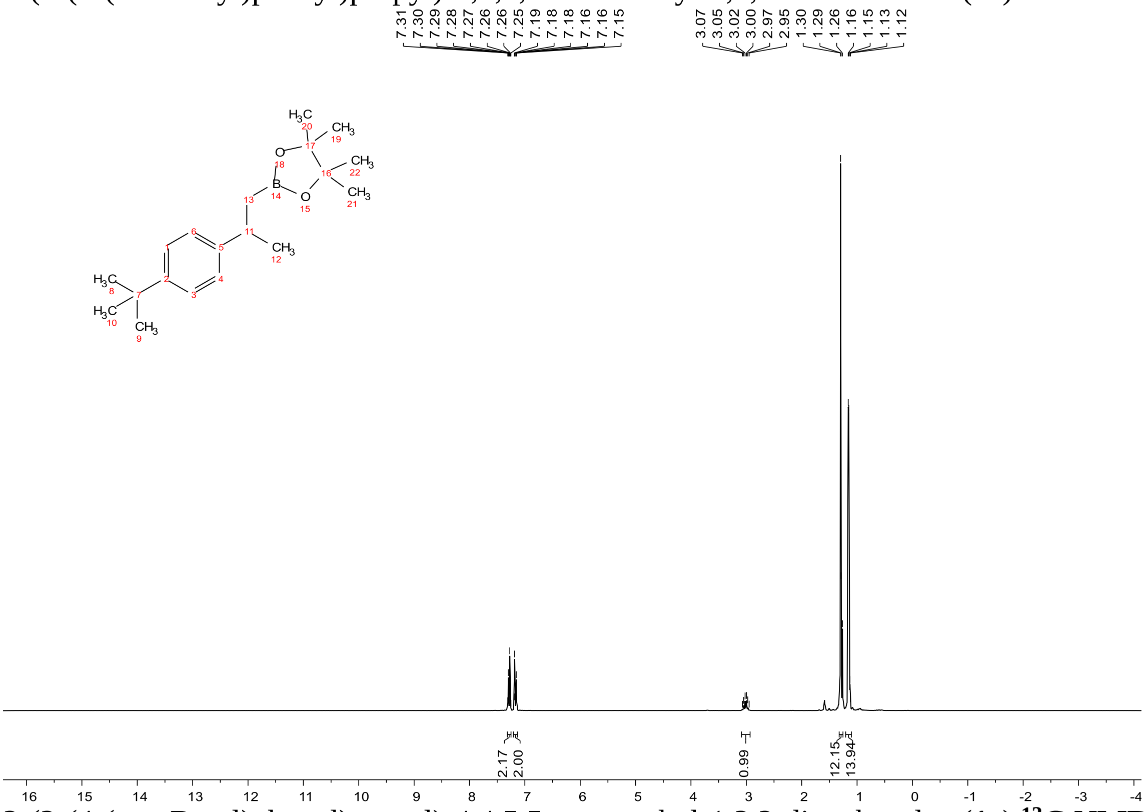

2-(2-(4-(*tert*-Butyl)phenyl)propyl)-4,4,5,5-tetramethyl-1,3,2-dioxaborolan (**1e**) **^{13}C NMR**

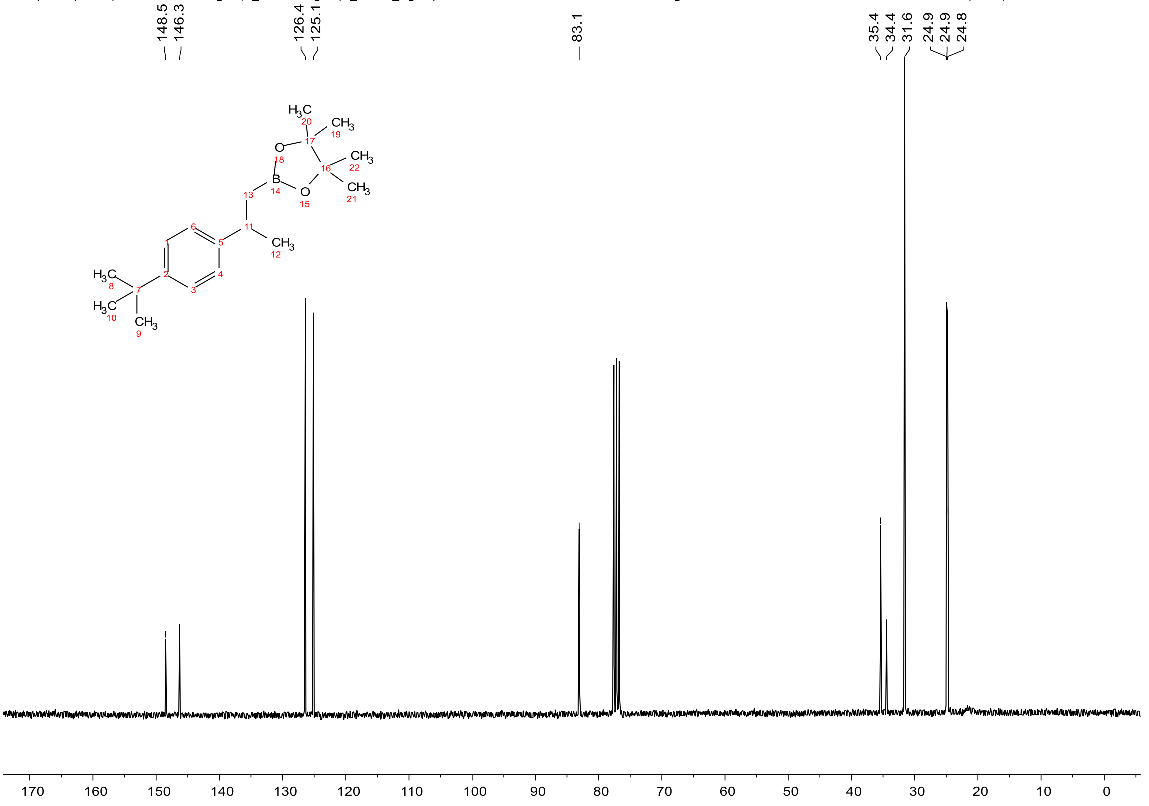

2-(2-(4-(*tert*-Butyl)phenyl)propyl)-4,4,5,5-tetramethyl-1,3,2-dioxaborolan (**1e**) **^{11}B NMR**

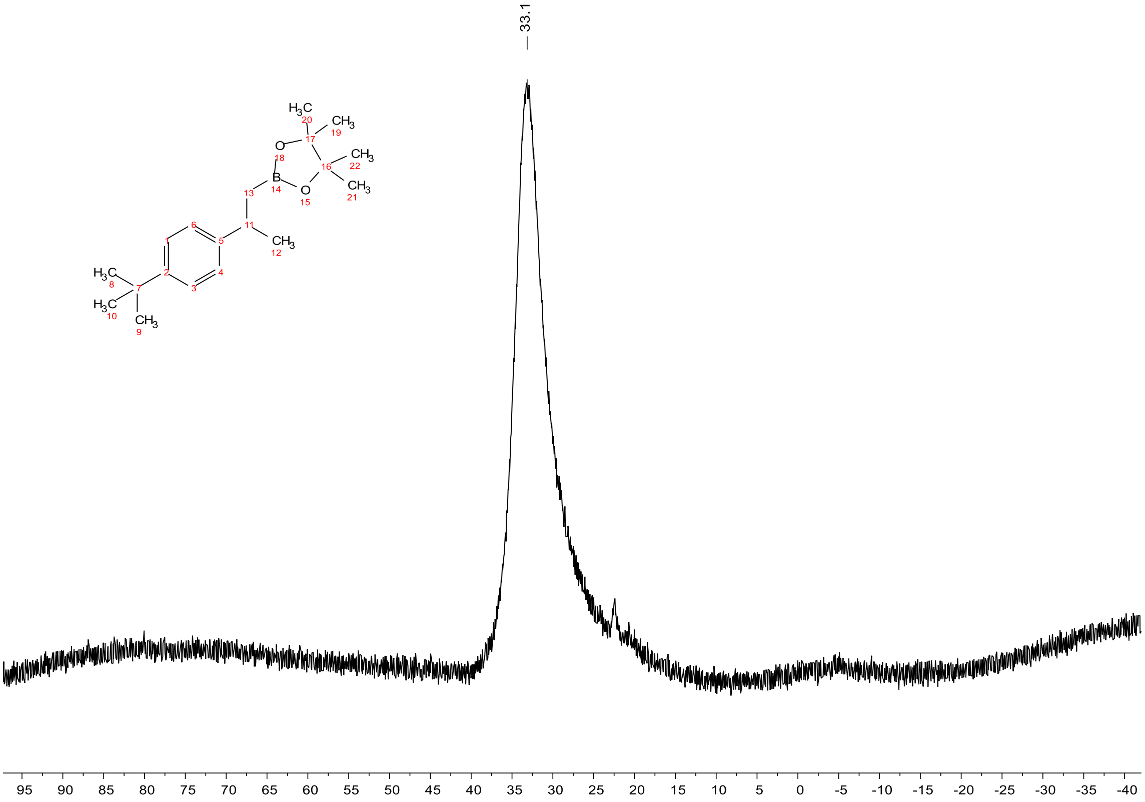

2-(2,2-Diphenylethyl)-4,4,5,5-tetramethyl-1,3,2-dioxaborolane (**1f**) **^{1}H NMR**

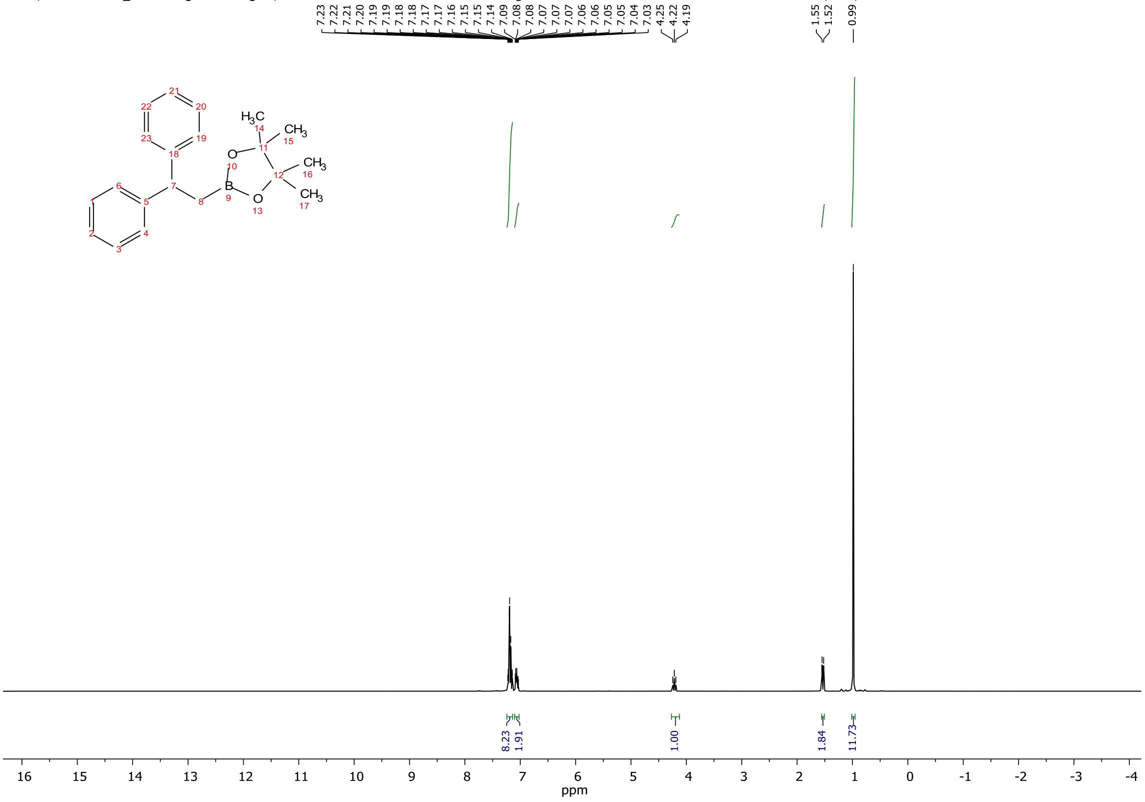

2-(2,2-Diphenylethyl)-4,4,5,5-tetramethyl-1,3,2-dioxaborolane (**1f**) **^{13}C NMR**

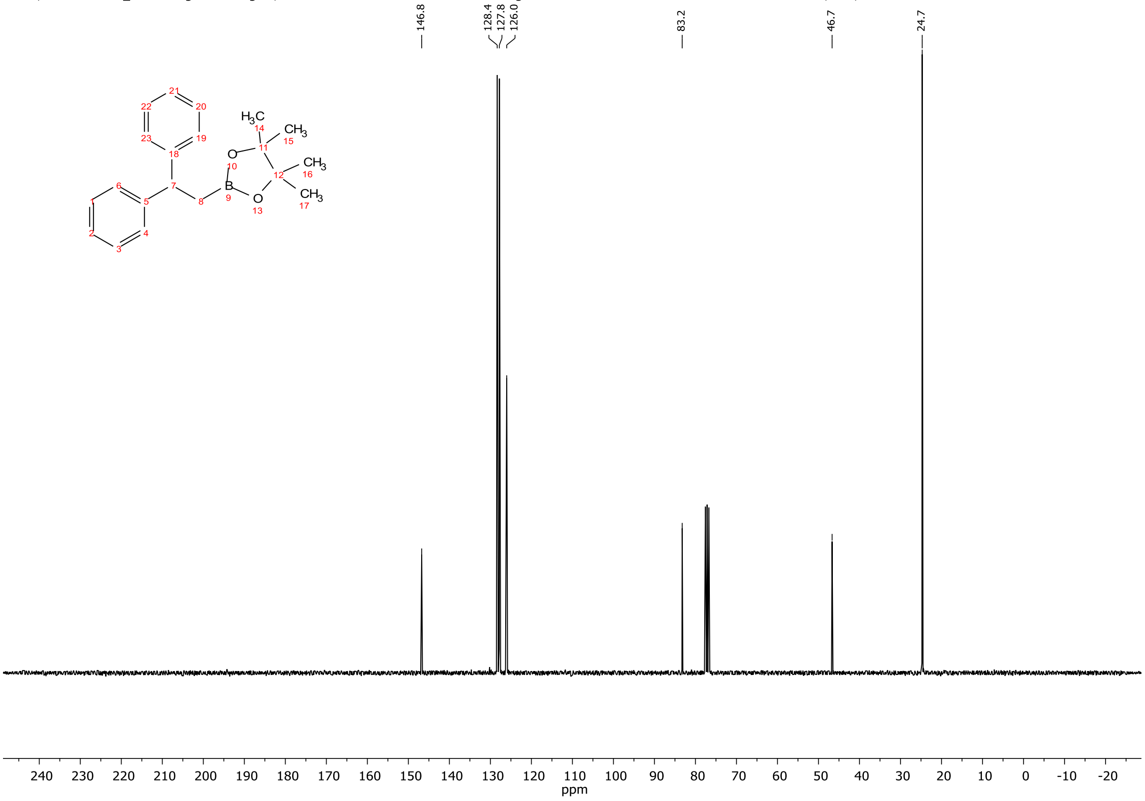

2-(2,2-Bis(4-methoxyphenyl)ethyl)-4,4,5,5-tetramethyl-1,3,2-dioxaborolane (**1g**) **^{1}H NMR**

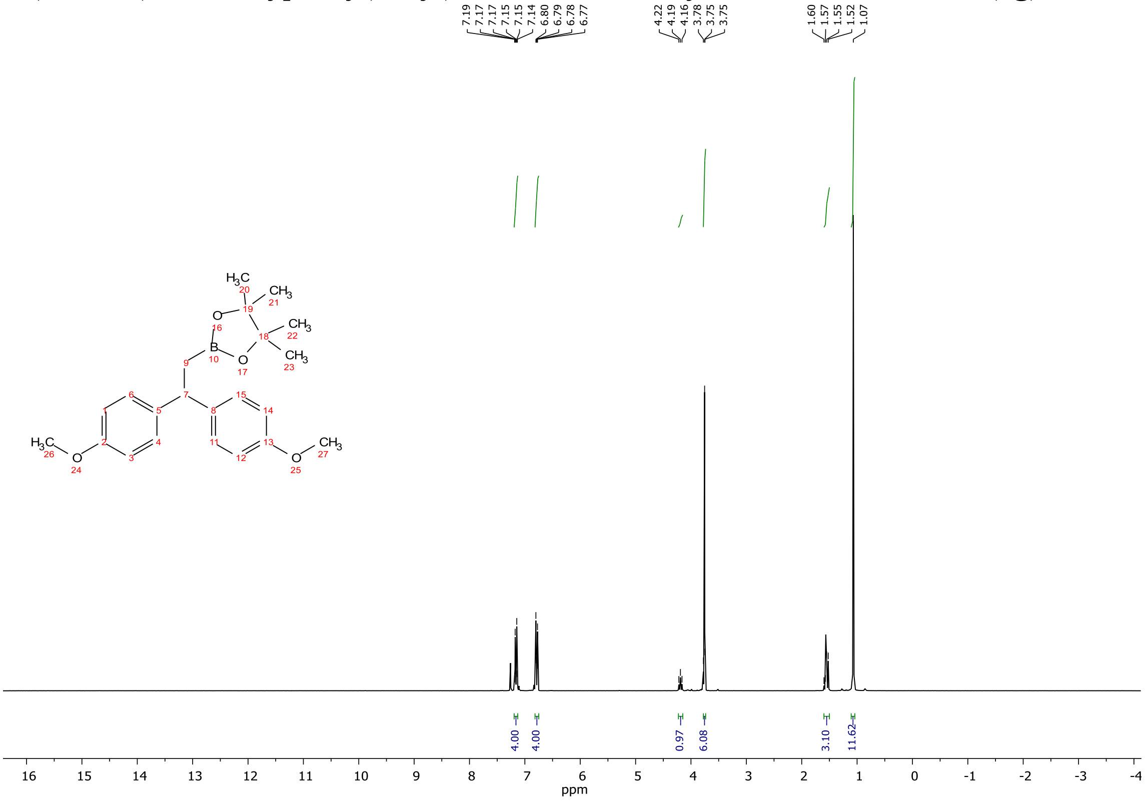

2-(2,2-Bis(4-methoxyphenyl)ethyl)-4,4,5,5-tetramethyl-1,3,2-dioxaborolane (**1g**) **^{13}C NMR**

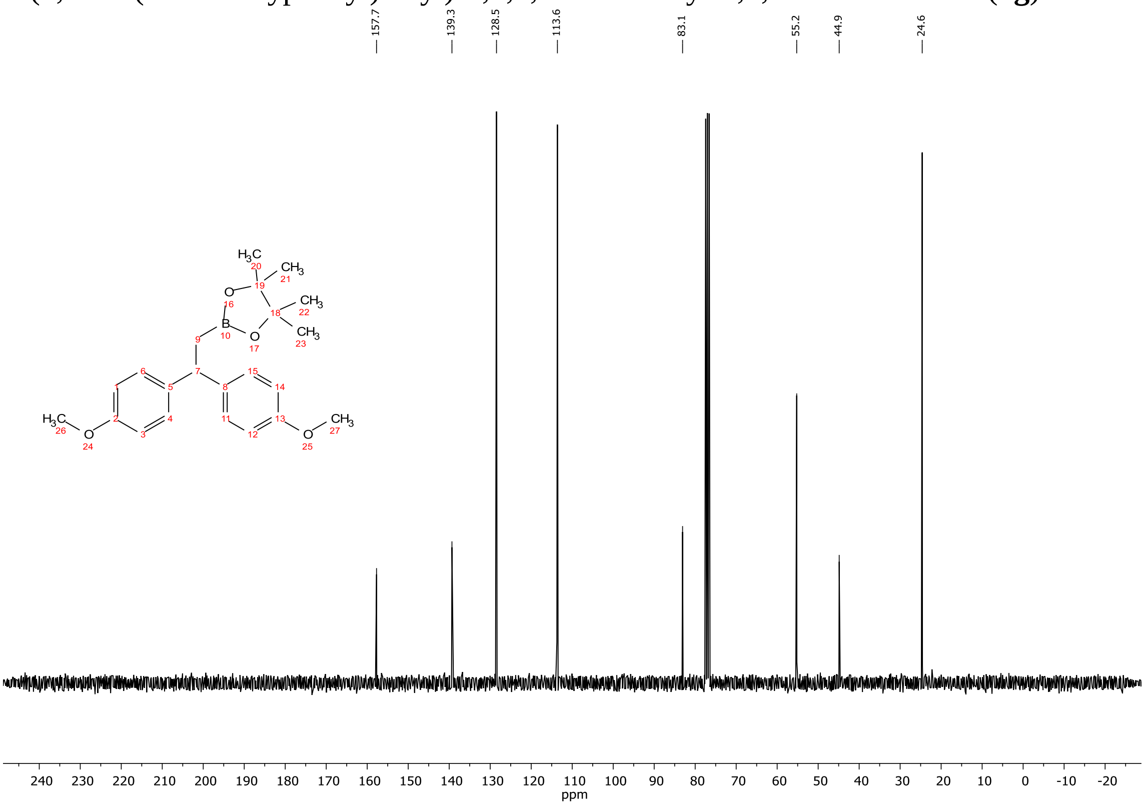

4,4,5,5-Tetramethyl-2-(2-(naphthalen-2-yl)ethyl)-1,3,2-dioxaborolane (**1h**) **^{1}H NMR**

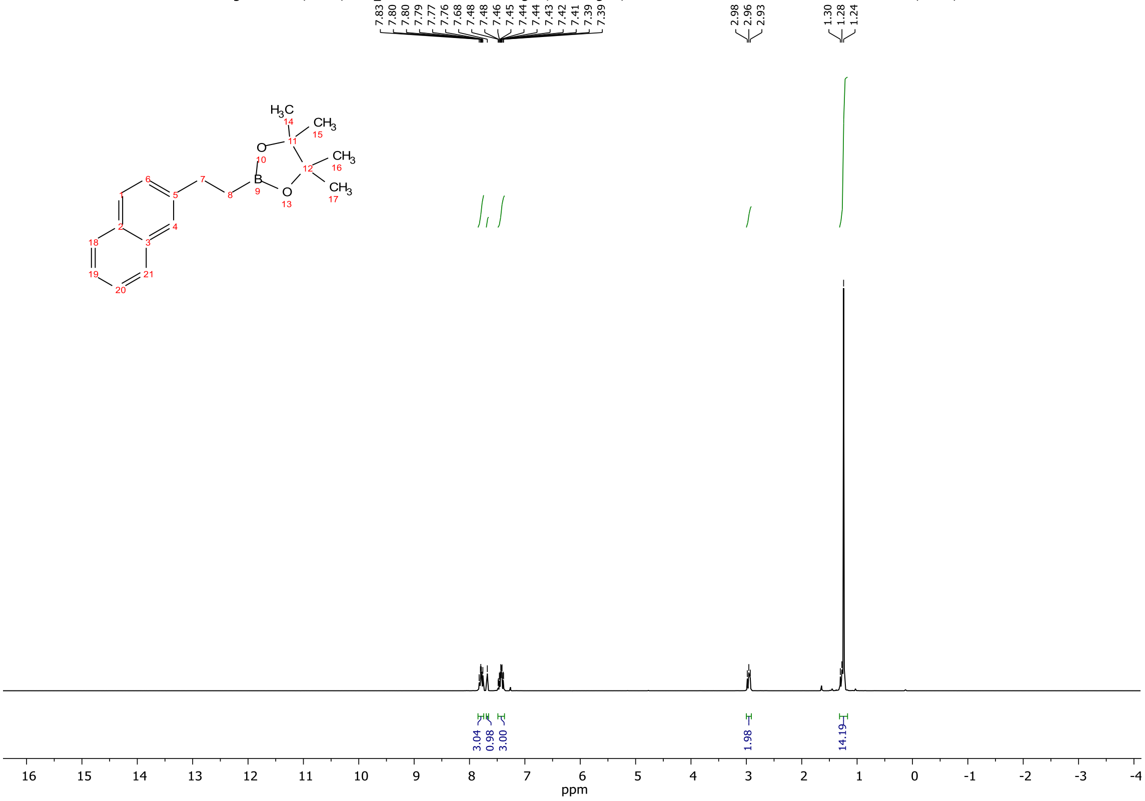

4,4,5,5-Tetramethyl-2-(2-(naphthalen-2-yl)ethyl)-1,3,2-dioxaborolane (**1h**) **^{13}C NMR**

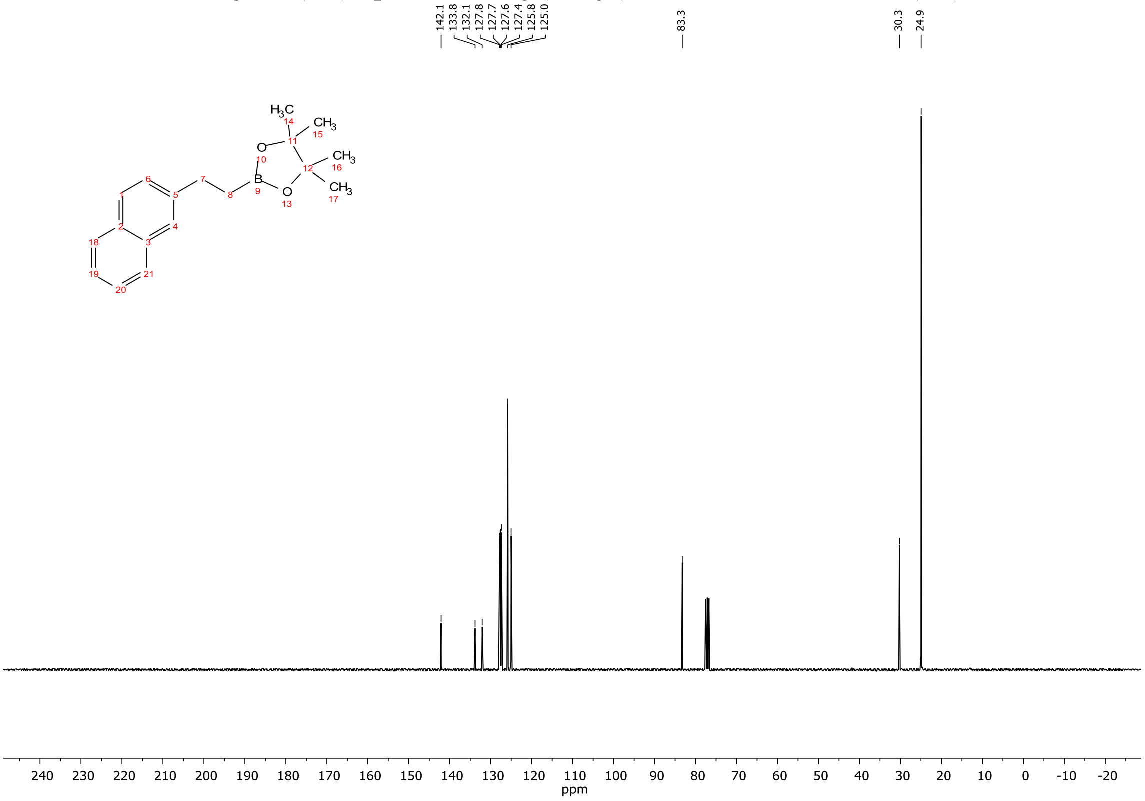

Triphenyl(3-(4,4,5,5-tetramethyl-1,3,2-dioxaborolan-2-yl)propyl)silane (**1i**, **14g**)

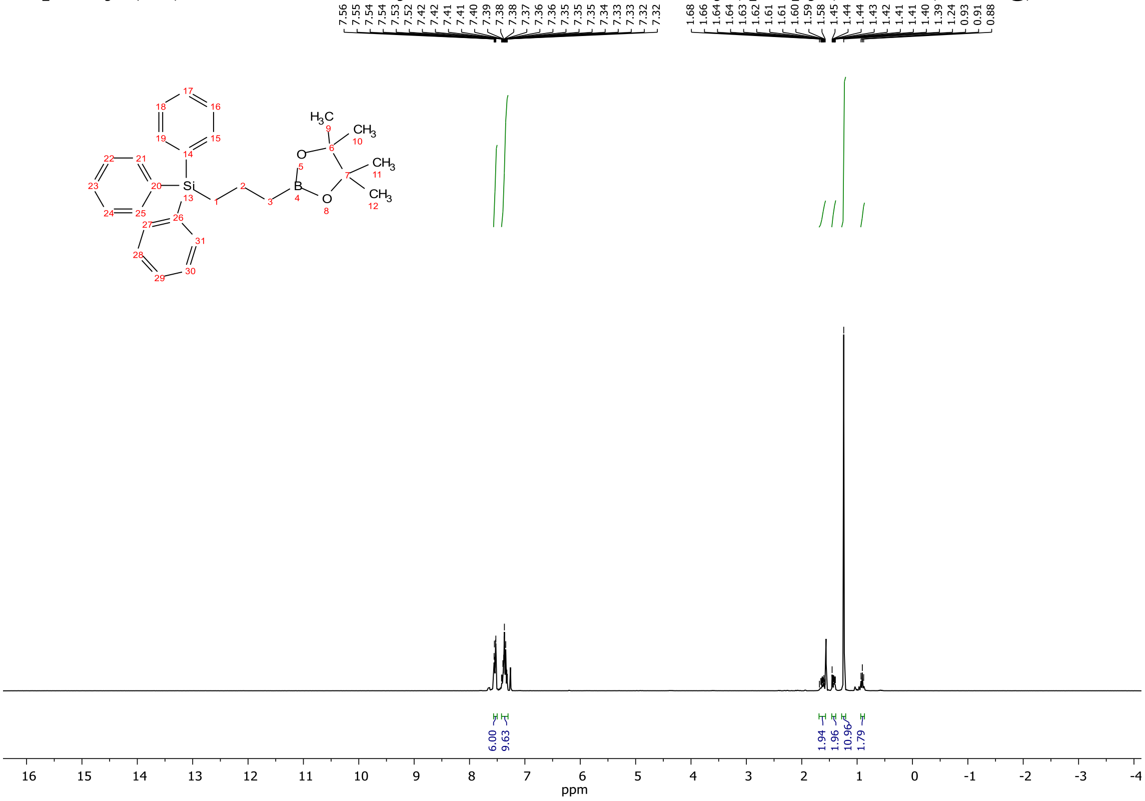

Triphenyl(3-(4,4,5,5-tetramethyl-1,3,2-dioxaborolan-2-yl)propyl)silane (**1i**, **14g**)

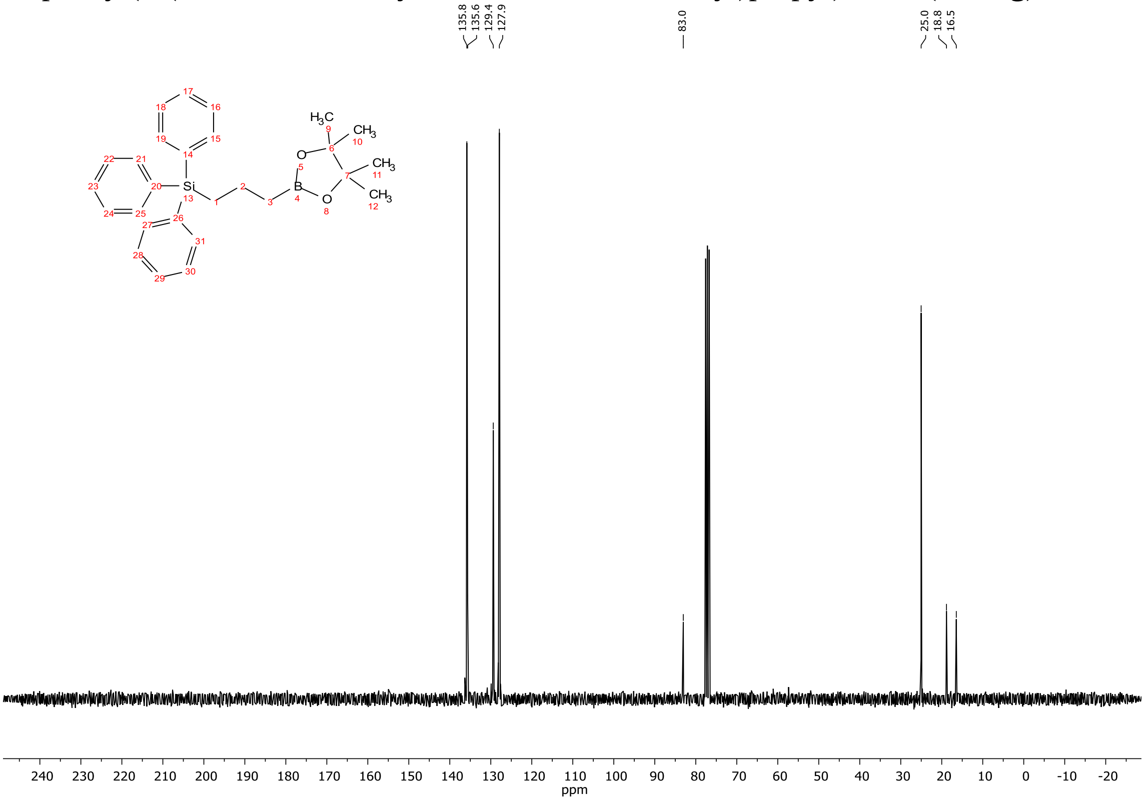

2-(2-(Benzo[d][1,3]dioxol-5-yl)pentyl)-4,4,5,5-tetramethyl-1,3,2-dioxaborolane (**1k**) **^{1}H NMR**

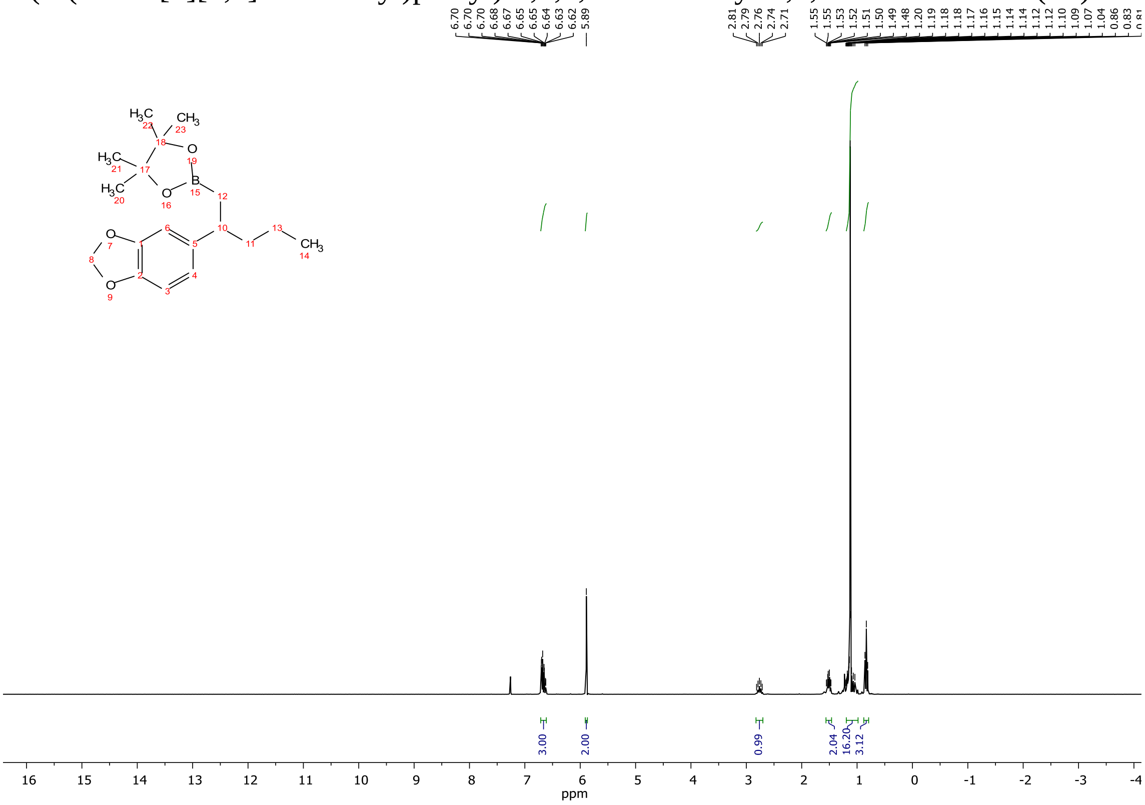

2-(2-(Benzo[d][1,3]dioxol-5-yl)pentyl)-4,4,5,5-tetramethyl-1,3,2-dioxaborolane (**1k**) ^{13}**C NMR**

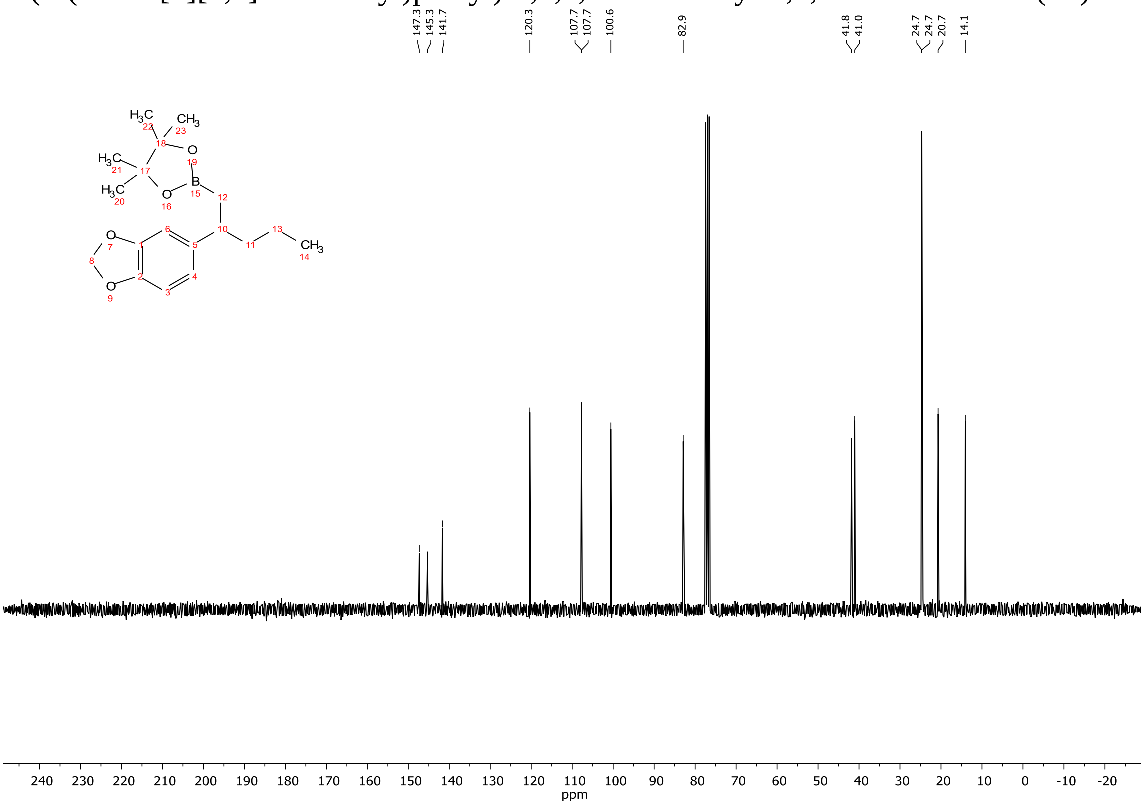

4,4,5,5-Tetramethyl-2-(1-(phenylsulfonyl)ethyl)-1,3,2-dioxaborolane (**1l**) ^{1}**H NMR**

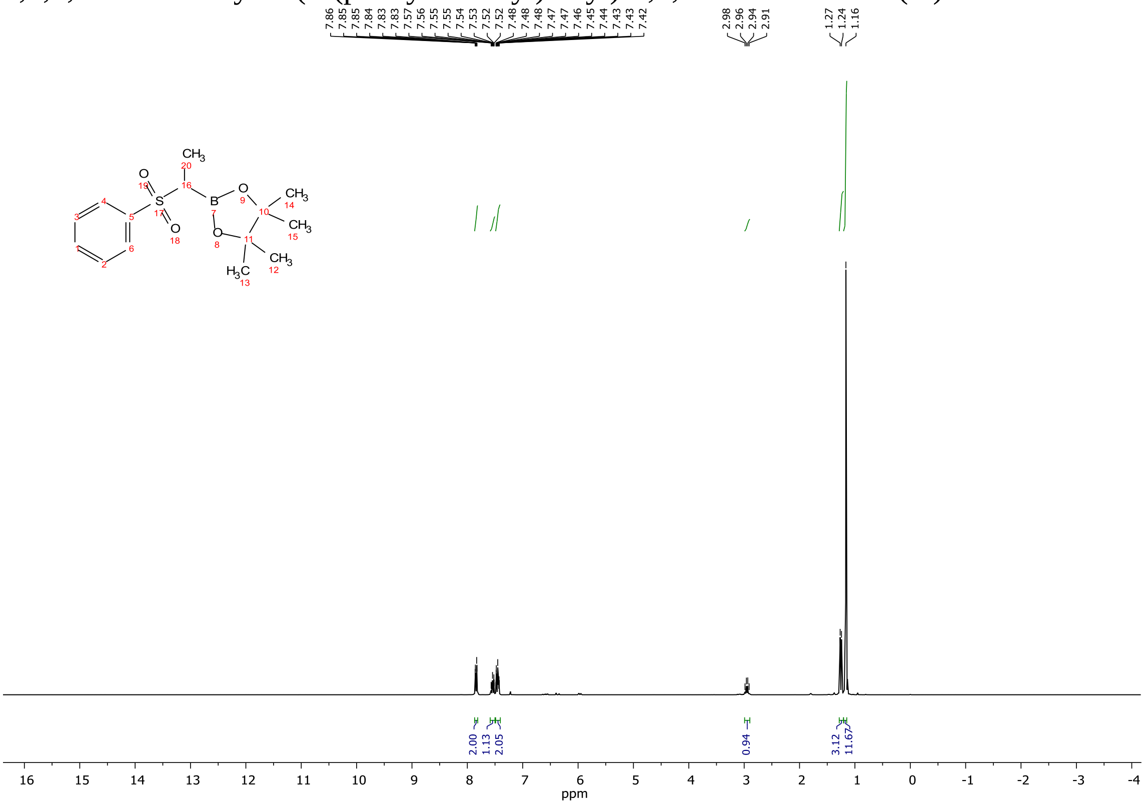

4,4,5,5-Tetramethyl-2-(1-(phenylsulfonyl)ethyl)-1,3,2-dioxaborolane (**1l**) ^{13}**C NMR**

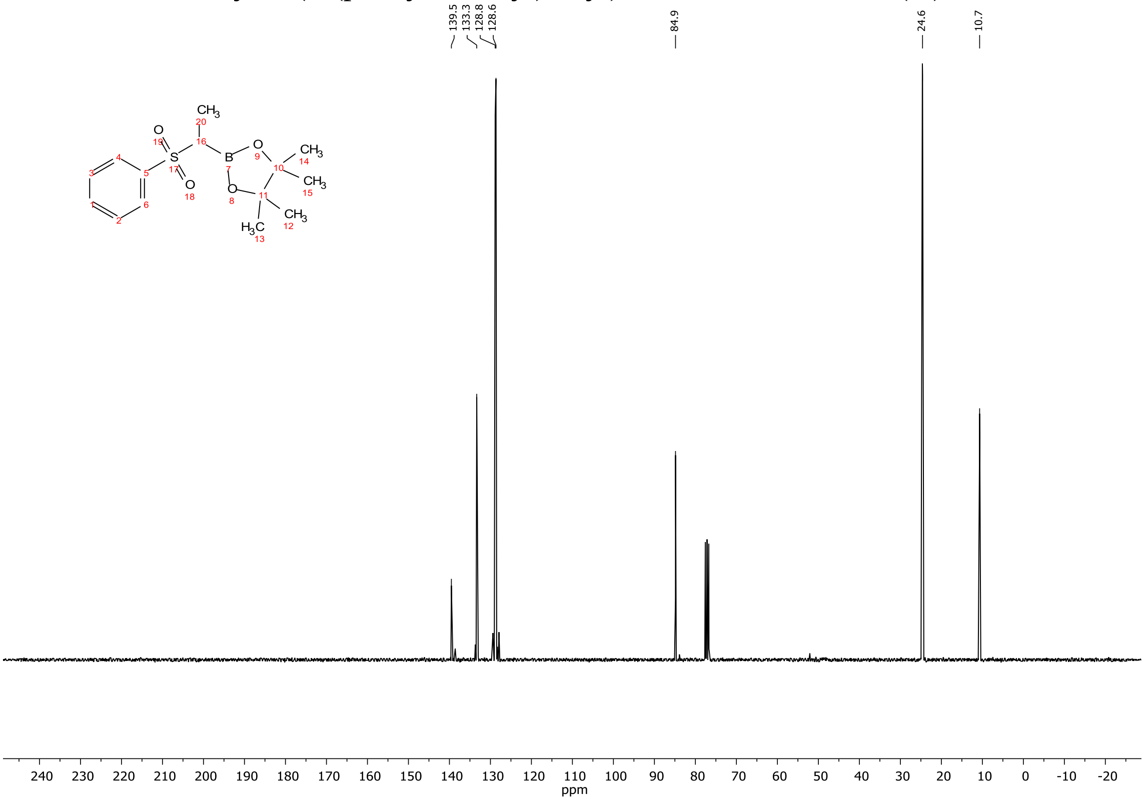

tert-Butyl (*R*)-2-(4,4,5,5-tetramethyl-1,3,2-dioxaborolan-2-yl)pyrrolidine-1-carboxylate (**1m**) ^{1}**H NMR**

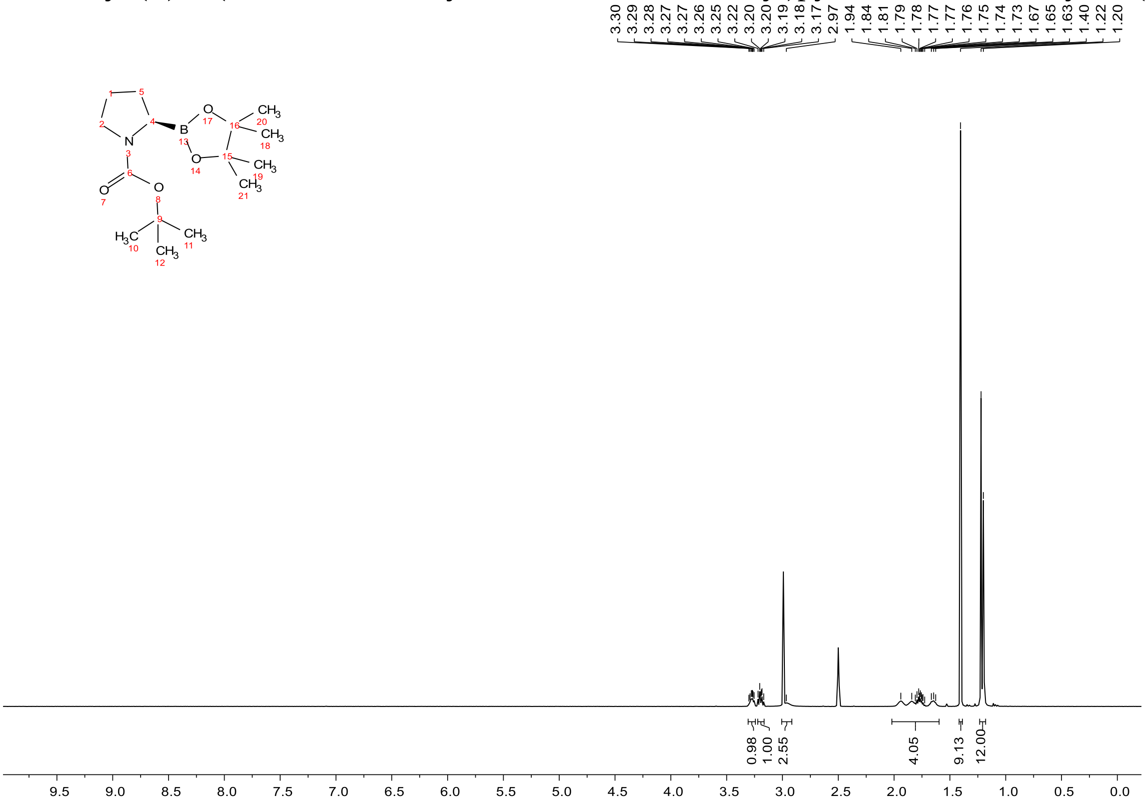

tert-Butyl (*R*)-2-(4,4,5,5-tetramethyl-1,3,2-dioxaborolan-2-yl)pyrrolidine-1-carboxylate (**1m**)
^{13}C NMR

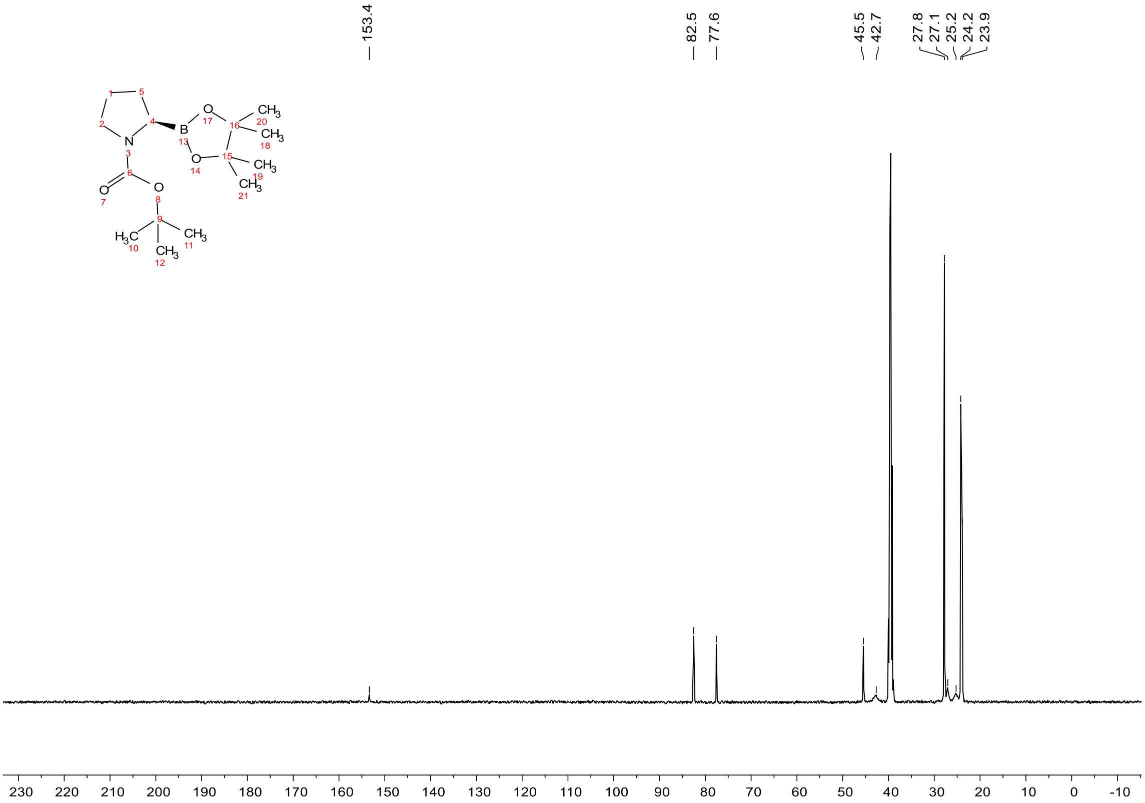

tert-Butyl (*R*)-2-(4,4,5,5-tetramethyl-1,3,2-dioxaborolan-2-yl)pyrrolidine-1-carboxylate (**1m**) **^{11}B NMR**

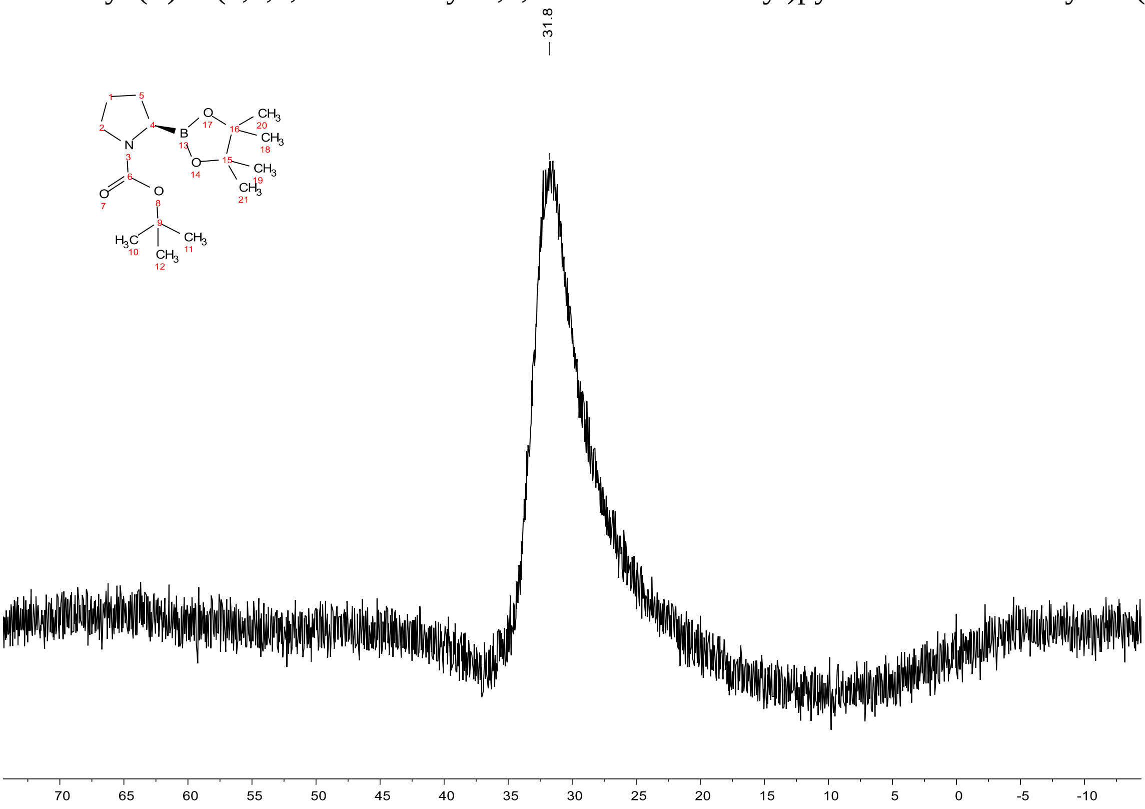

tert-Butyl 4-methyl-4-(4,4,5,5-tetramethyl-1,3,2-dioxaborolan-2-yl)octanoate (**1n**) **^{1}H NMR**

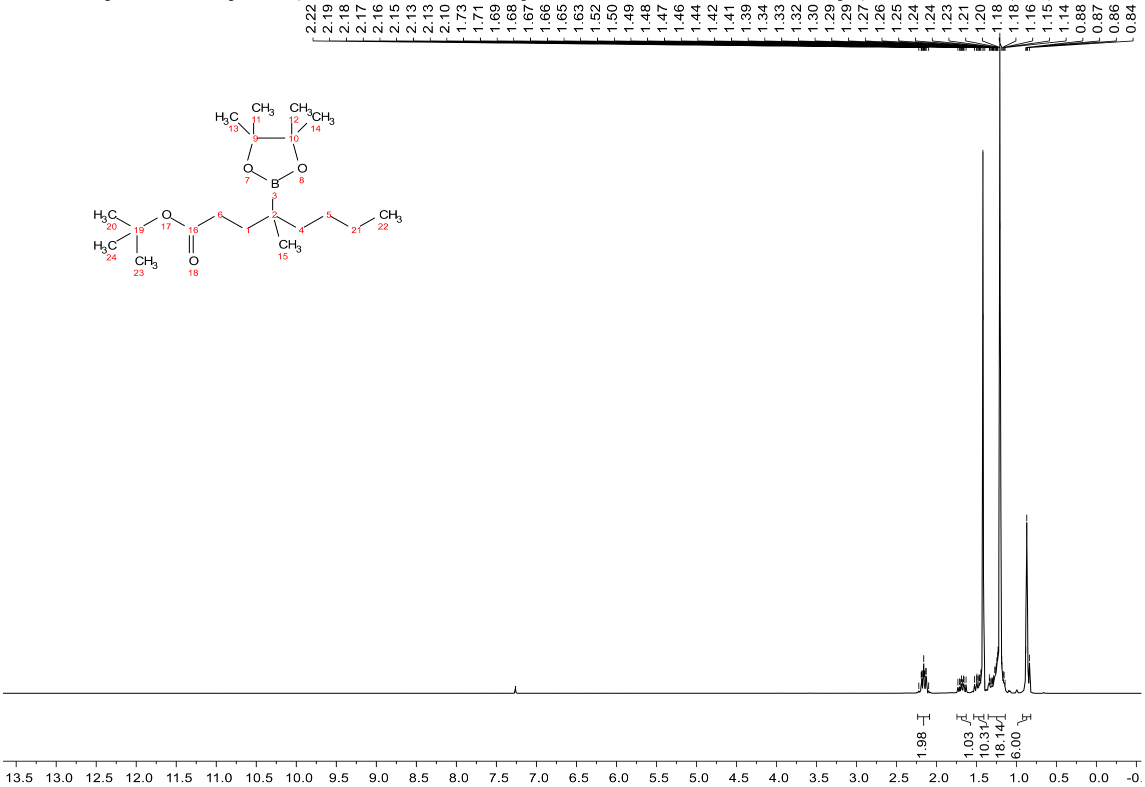

tert-Butyl 4-methyl-4-(4,4,5,5-tetramethyl-1,3,2-dioxaborolan-2-yl)octanoate (**1n**) **^{13}C NMR**

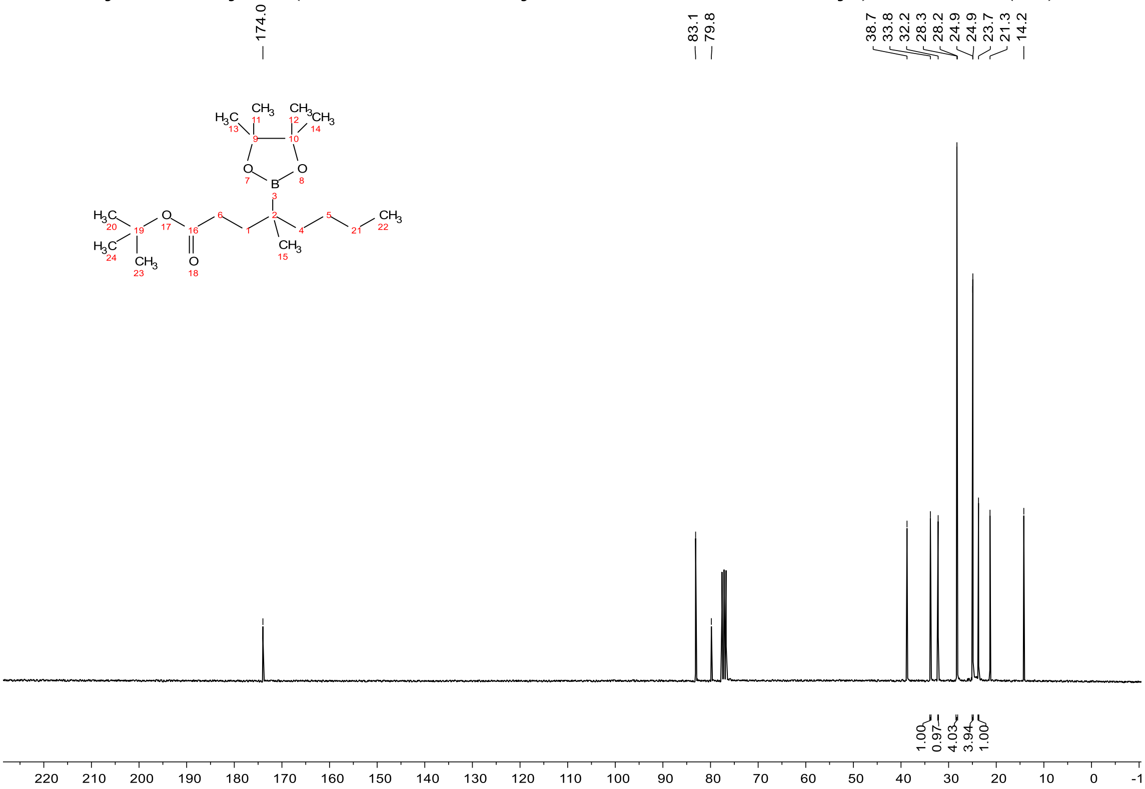

2-(1-Cyclopropyl-3-phenylpropyl)-4,4,5,5-tetramethyl-1,3,2-dioxaborolan (1o) **^{1}H NMR**

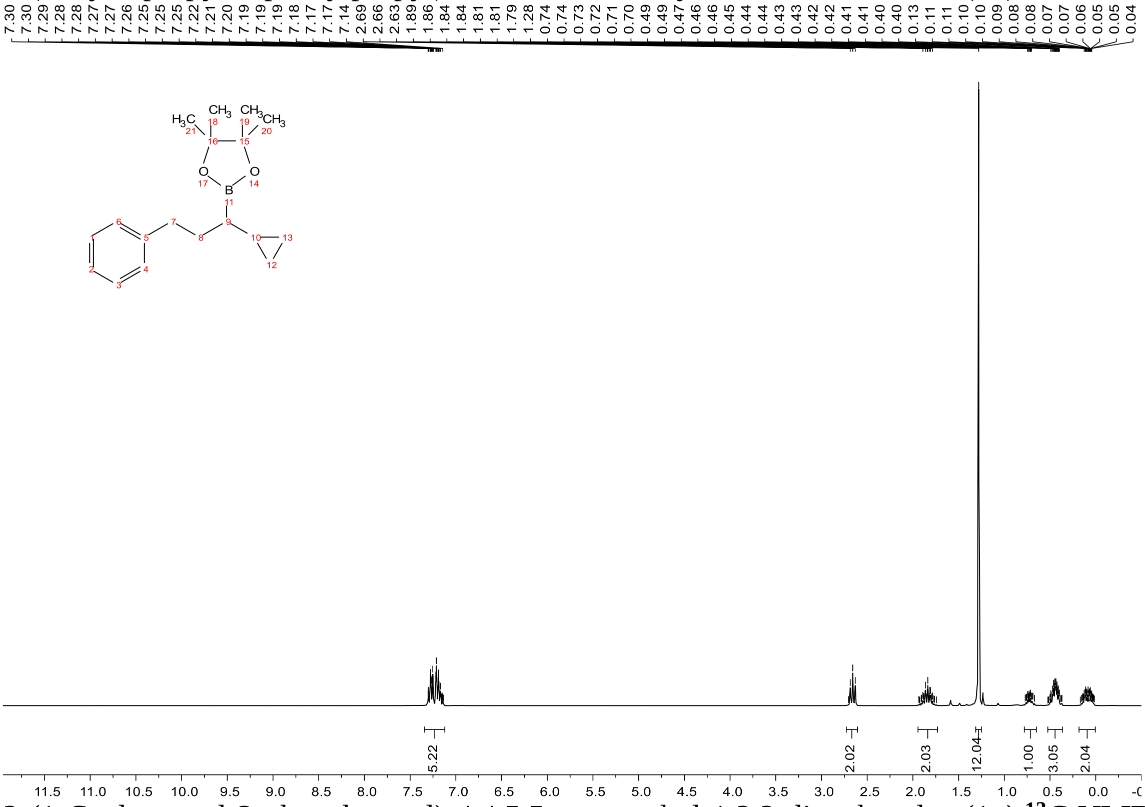

2-(1-Cyclopropyl-3-phenylpropyl)-4,4,5,5-tetramethyl-1,3,2-dioxaborolan (1o) **^{13}C NMR**

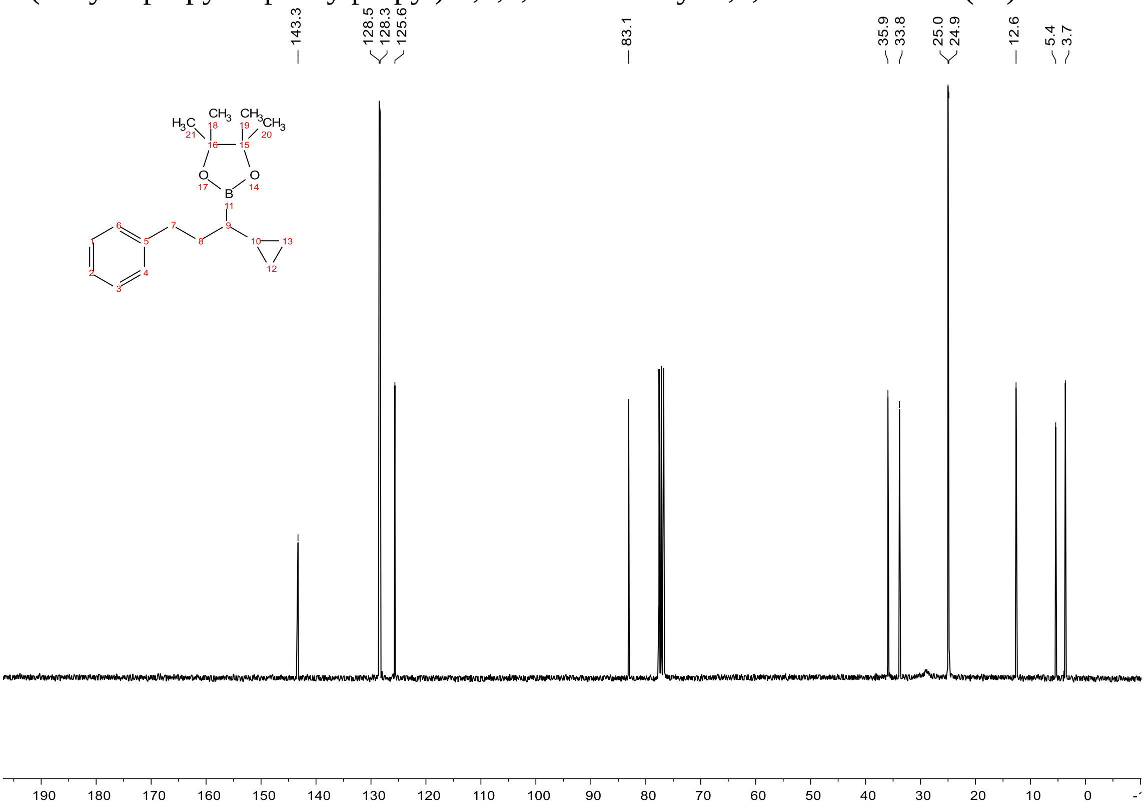

2-(1-Cyclopropyl-3-phenylpropyl)-4,4,5,5-tetramethyl-1,3,2-dioxaborolan (1o) **^{11}B NMR**

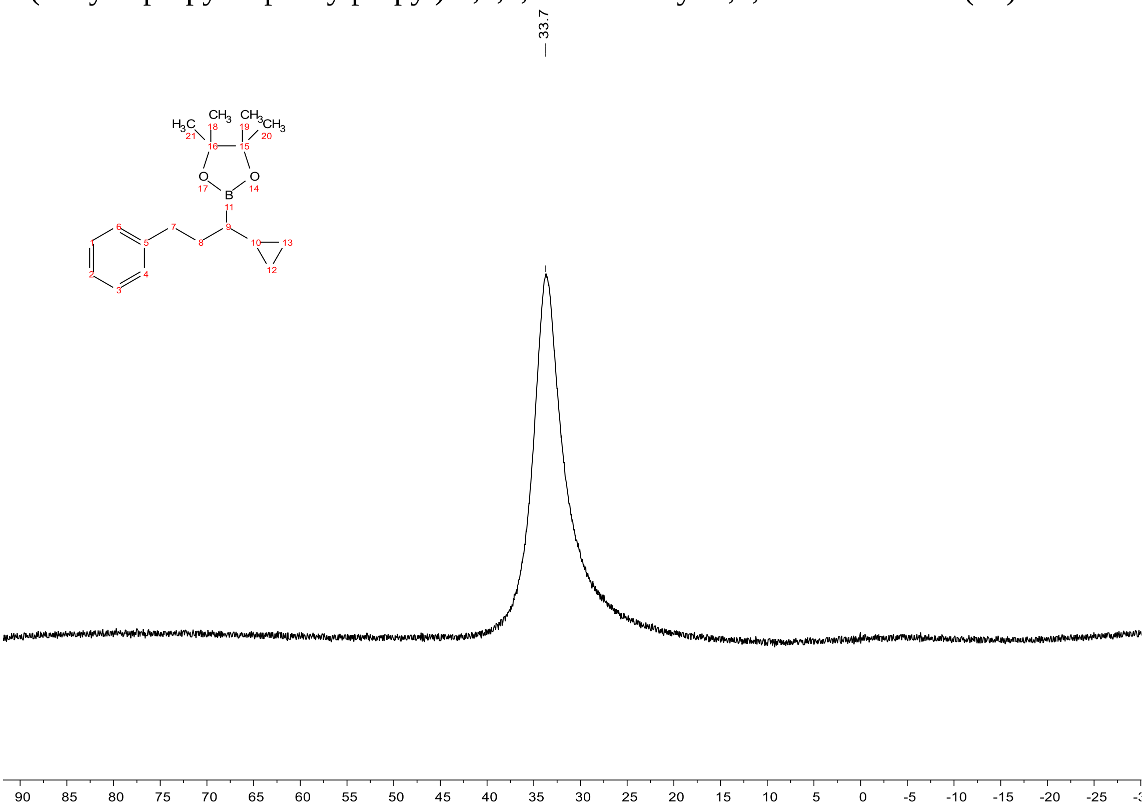

2-(2-(3,4-Dimethylphenyl)propyl)-4,4,5,5-tetramethyl-1,3,2-dioxaborolane (**14a**) **^{1}H NMR**

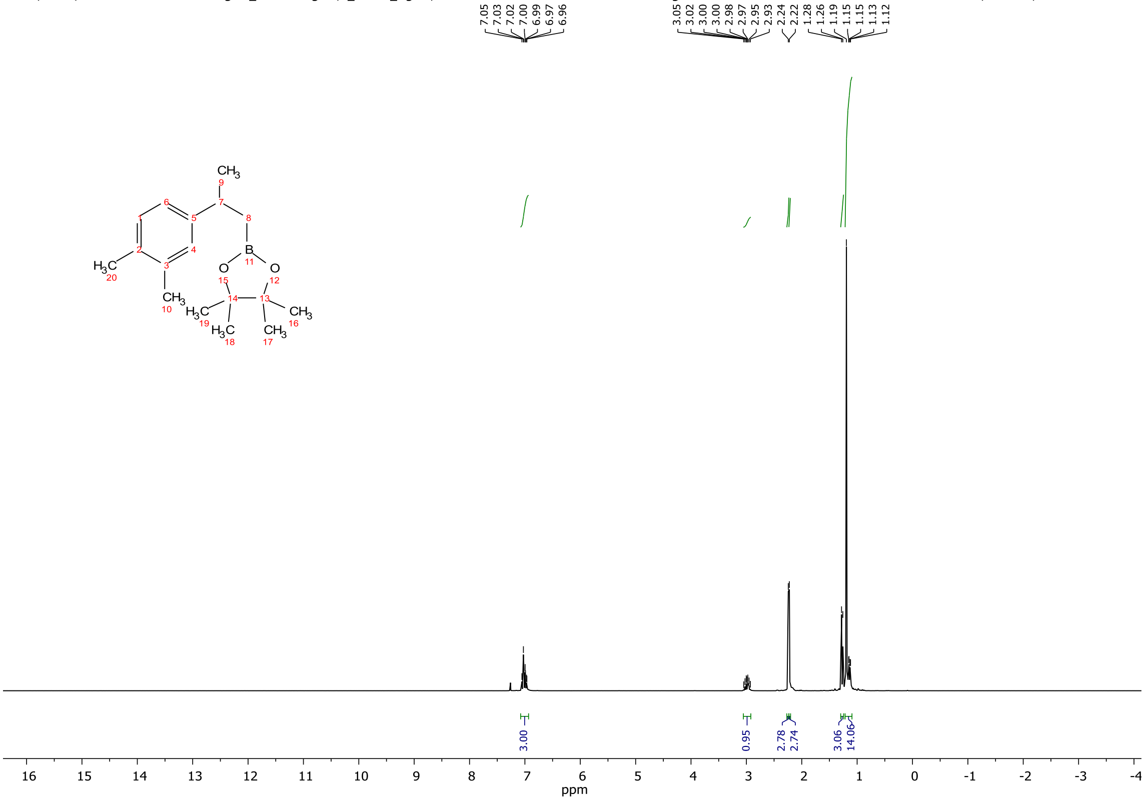

2-(2-(3,4-Dimethylphenyl)propyl)-4,4,5,5-tetramethyl-1,3,2-dioxaborolane (**14a**) ^{13}C **NMR**

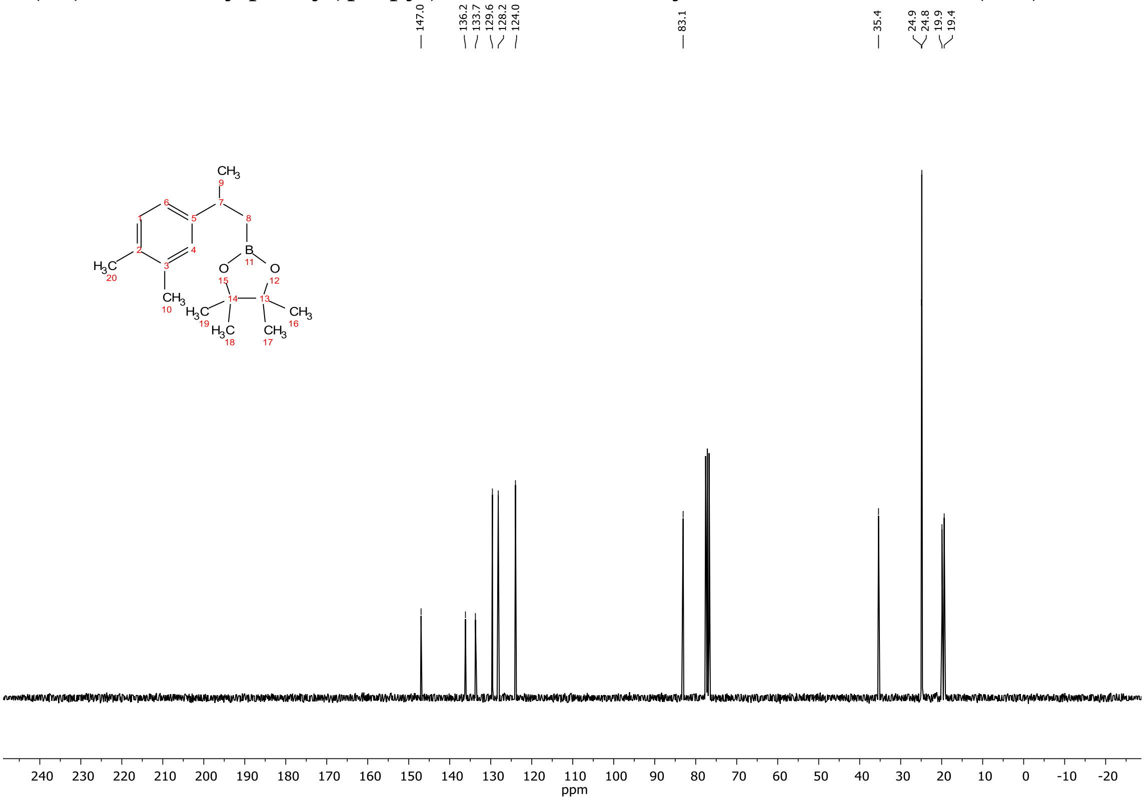

2-(2-(2-Methoxyphenyl)propyl)-4,4,5,5-tetramethyl-1,3,2-dioxaborolane (**14b**) ^{1}H **NMR**

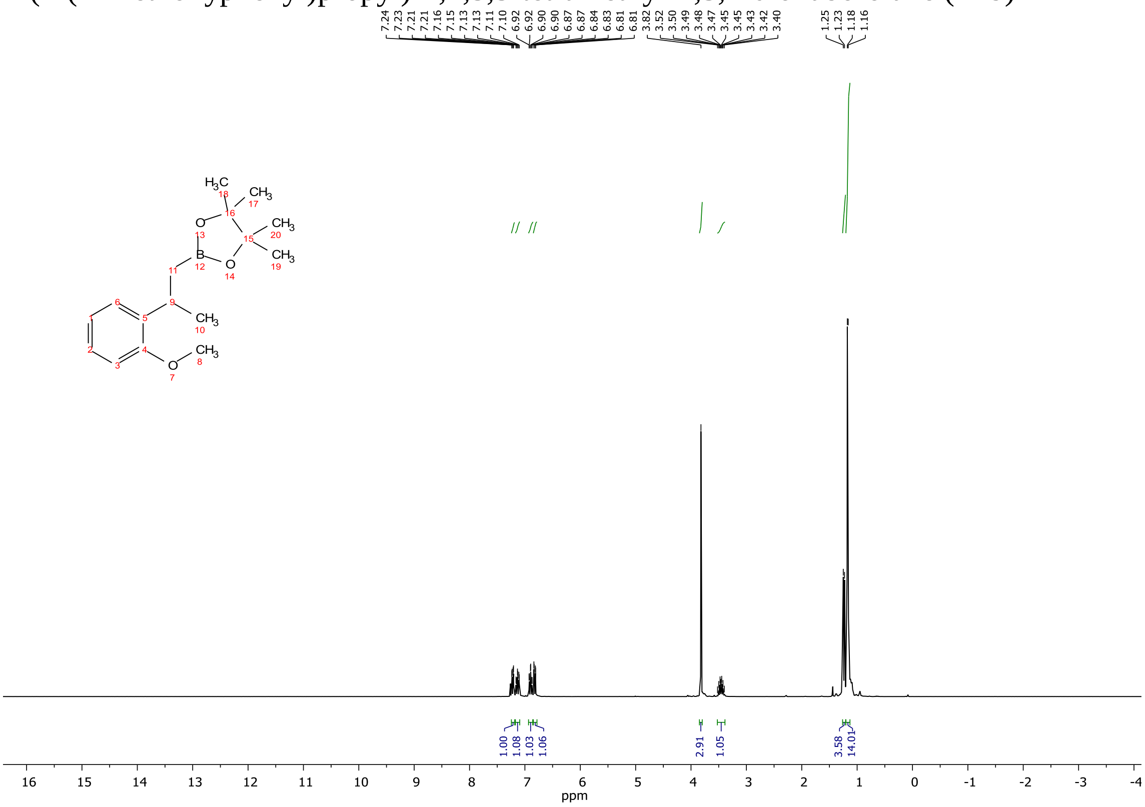

2-(2-(2-Methoxyphenyl)propyl)-4,4,5,5-tetramethyl-1,3,2-dioxaborolane (**14b**) ^{13}C NMR

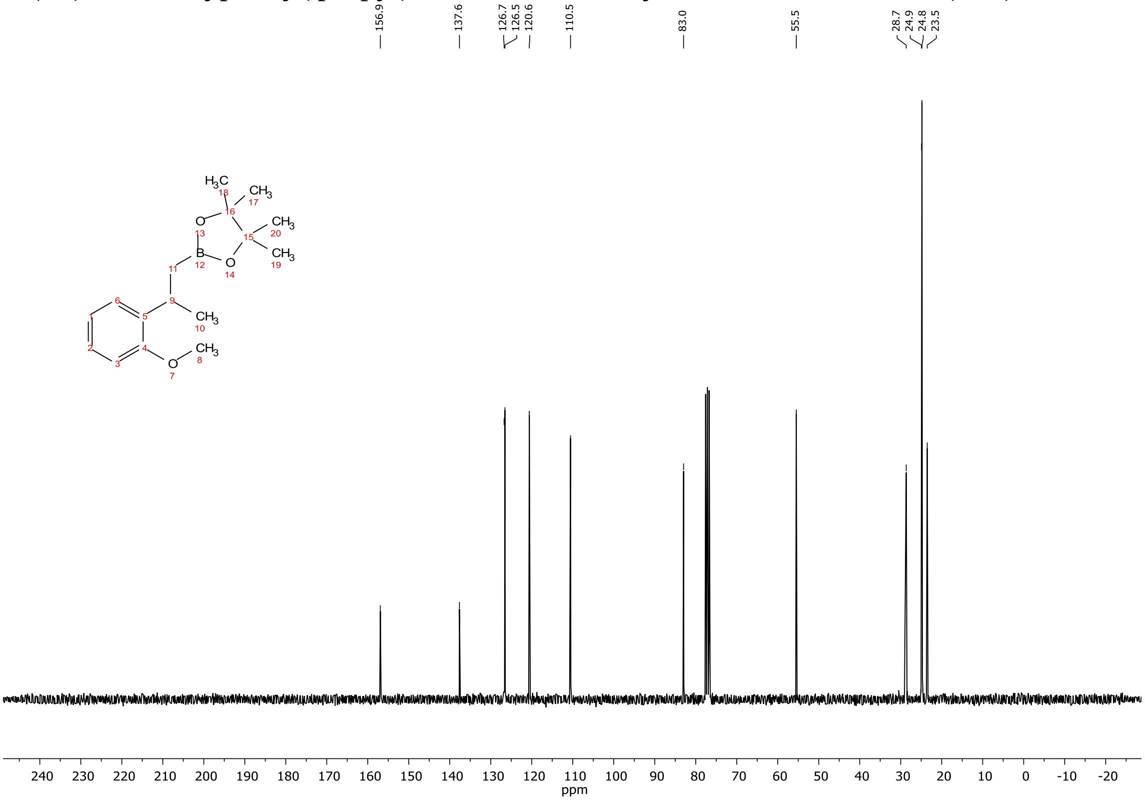

2-(2,2-Bis(4-fluorophenyl)ethyl)-4,4,5,5-tetramethyl-1,3,2-dioxaborolane (**14d**) ^{1}H NMR

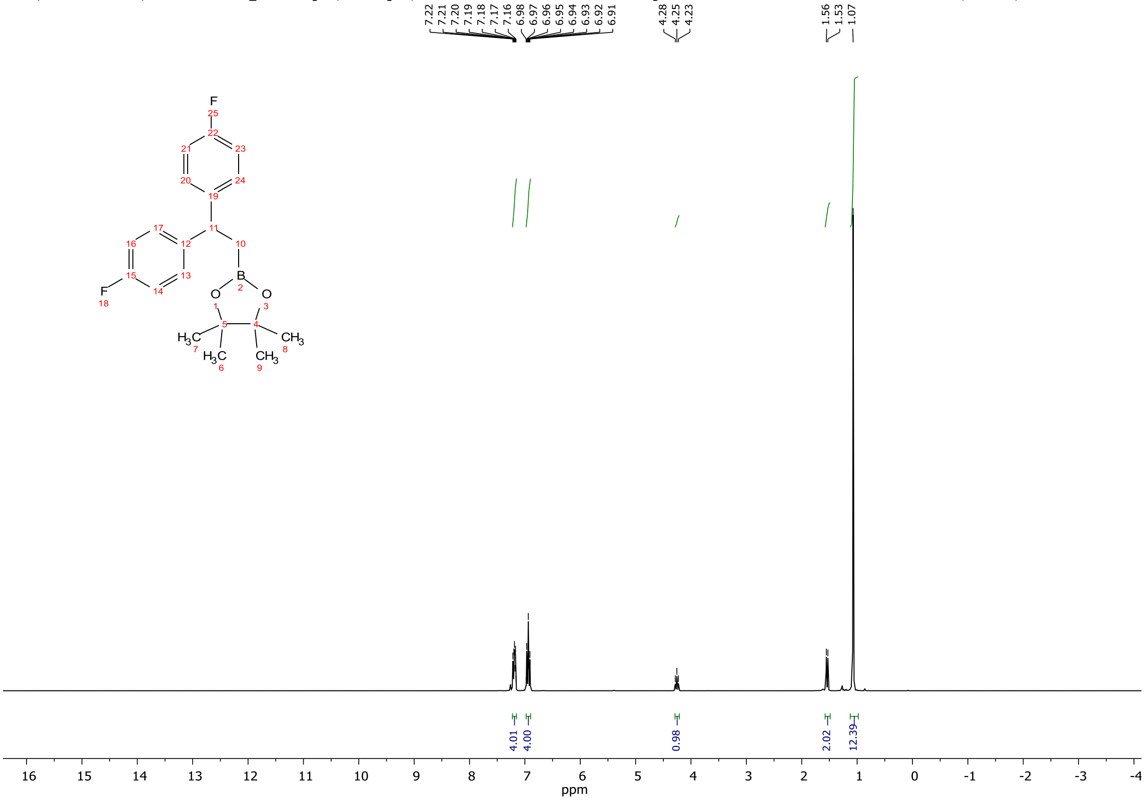

2-(2,2-Bis(4-fluorophenyl)ethyl)-4,4,5,5-tetramethyl-1,3,2-dioxaborolane (**14d**) ^{13}**C NMR**

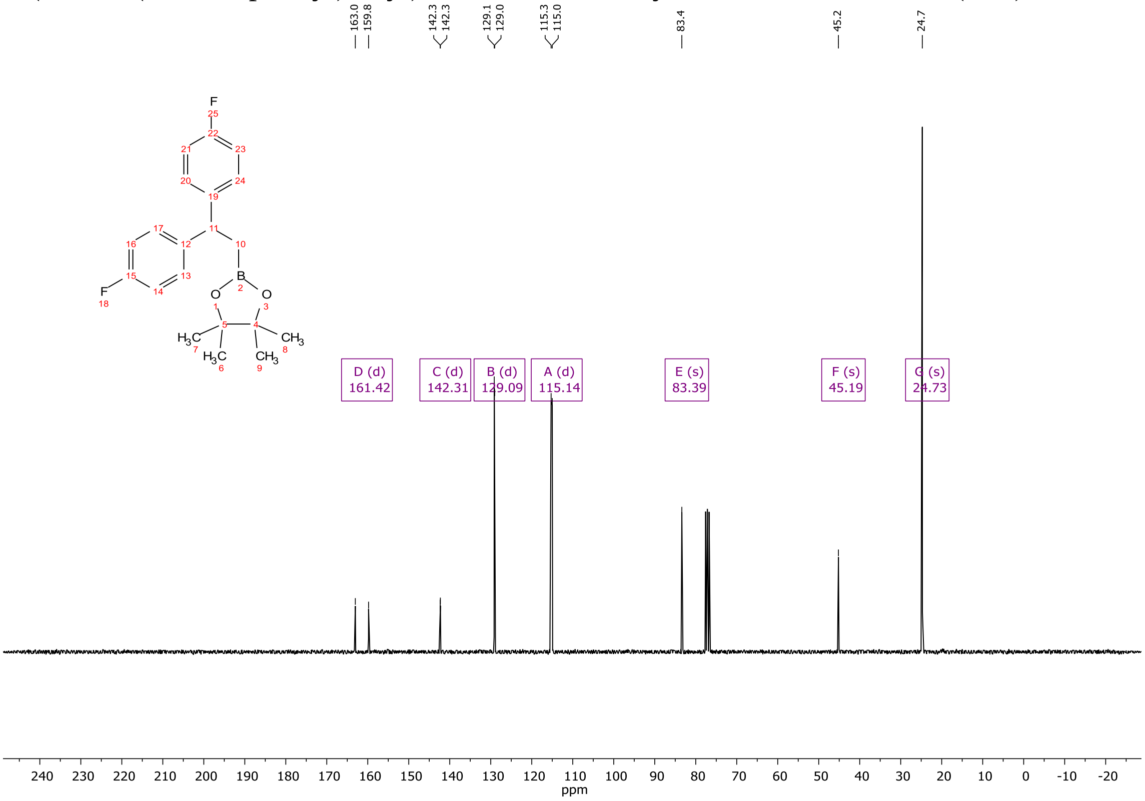

2-(2-(Benzothiophen-2-yl)propyl)-4,4,5,5-tetramethyl-1,3,2-dioxaborolane (**14e**) ^{1}**H NMR**

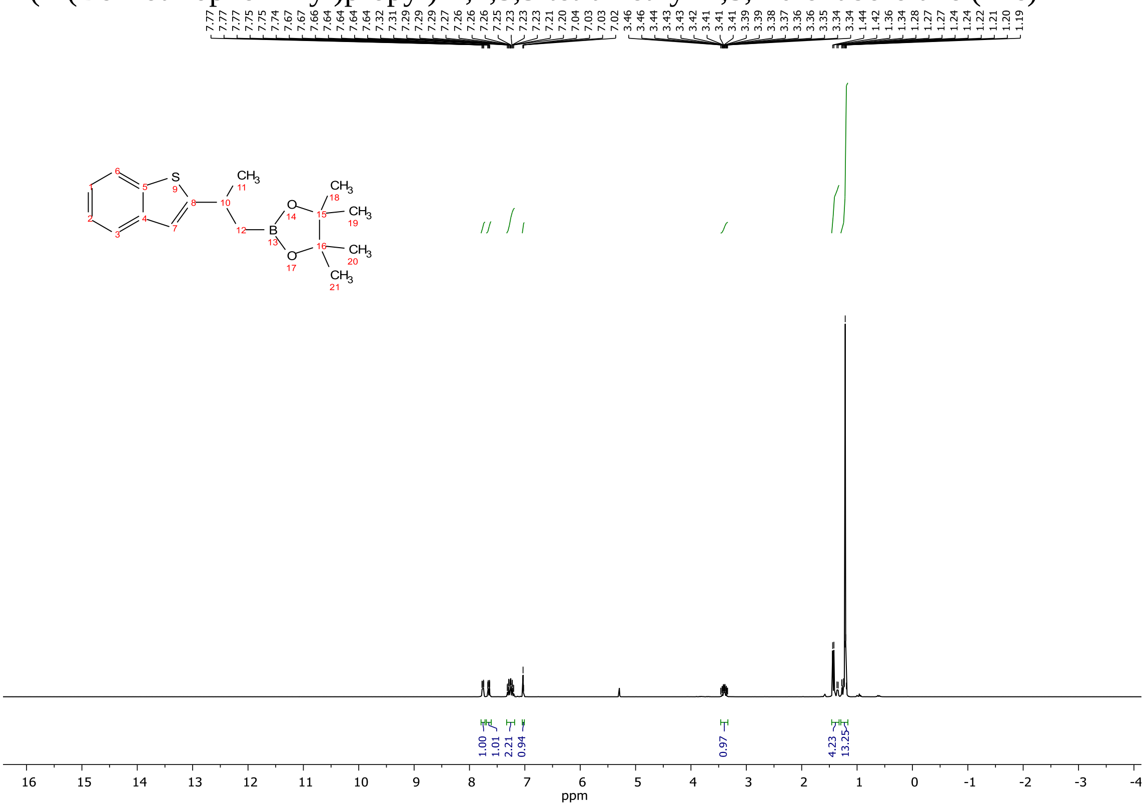

2-(2-(Benzothiophen-2-yl)propyl)-4,4,5,5-tetramethyl-1,3,2-dioxaborolane (**14e**) ^{13}C NMR

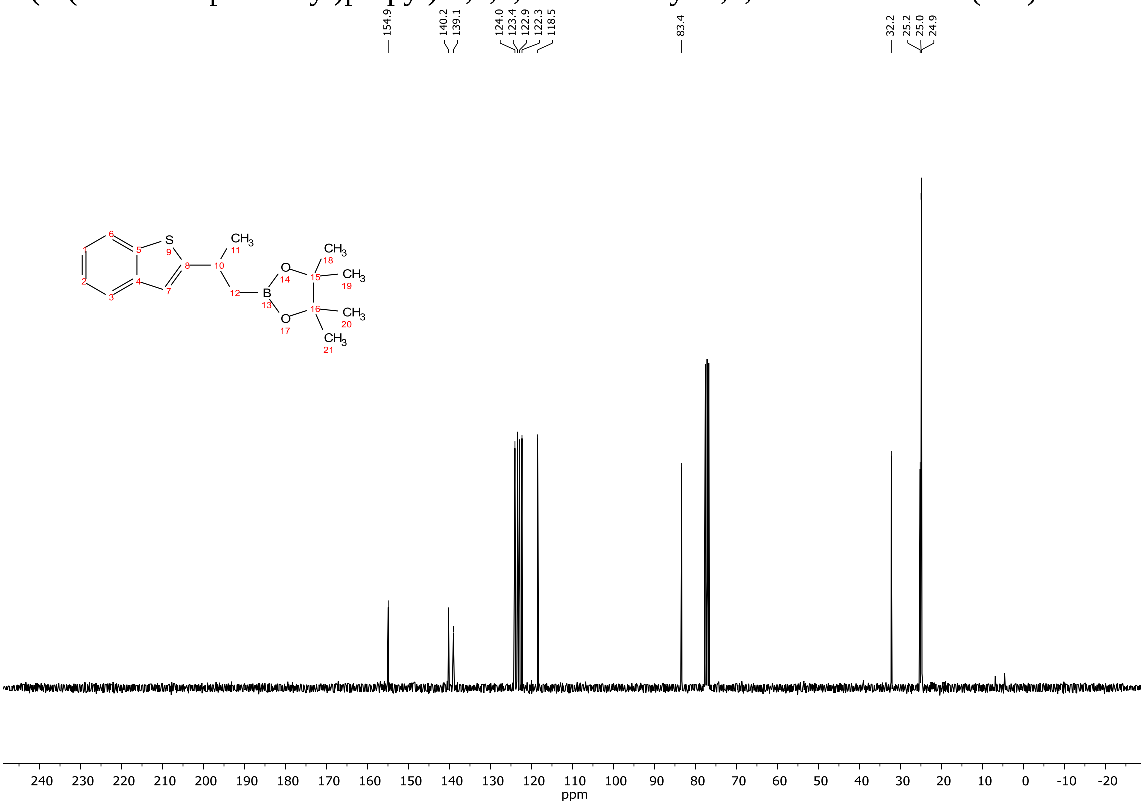

2-(2-(Benzofuran-2-yl)propyl)-4,4,5,5-tetramethyl-1,3,2-dioxaborolane (**14f**) ^{1}H NMR

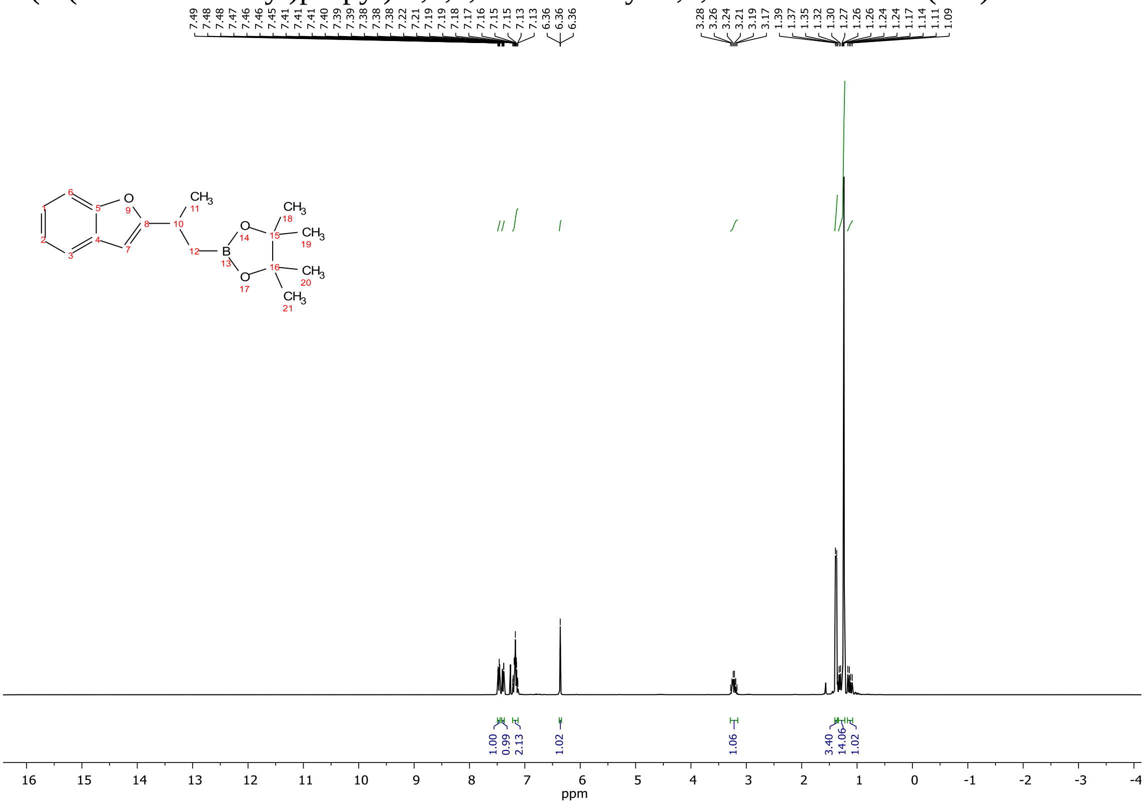

2-(2-(Benzofuran-2-yl)propyl)-4,4,5,5-tetramethyl-1,3,2-dioxaborolane (**14f**) ^{13}C NMR

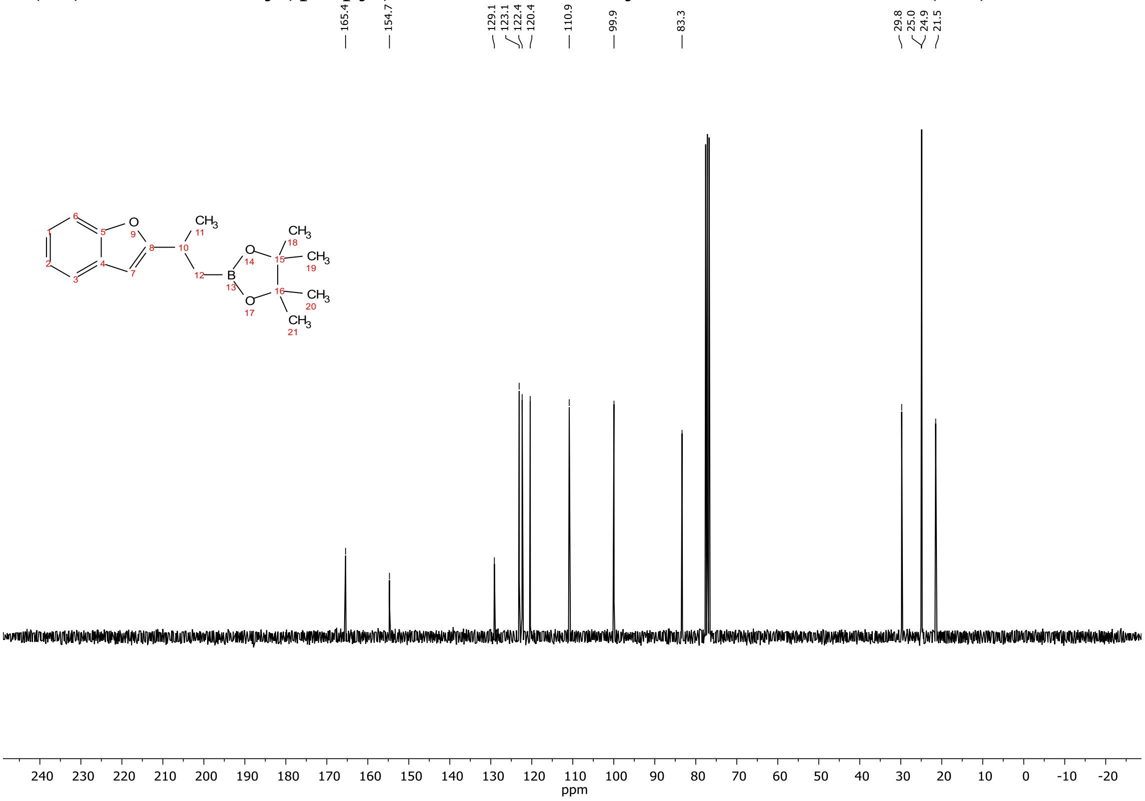

4-(1-(4,4,5,5-Tetramethyl-1,3,2-dioxaborolan-2-yl)propan-2-yl)phenyl 4-methylbenzenesulfonate (**14h**) ^{1}H NMR

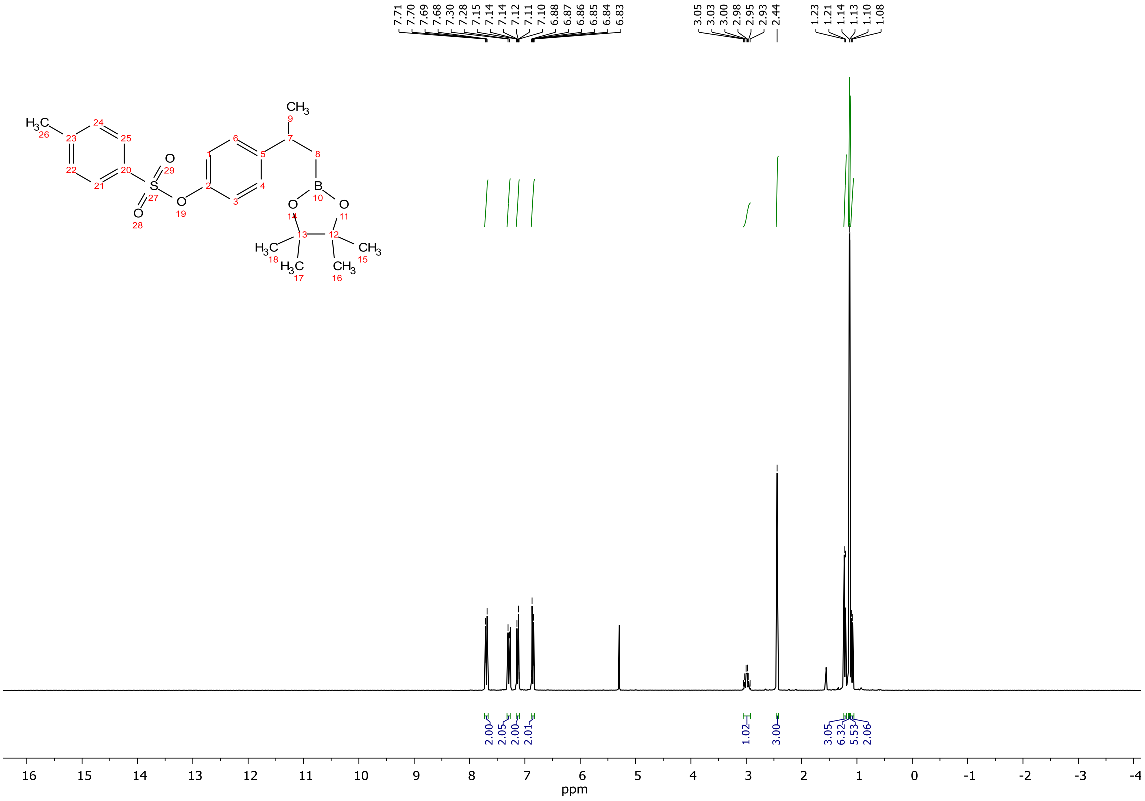

4-(1-(4,4,5,5-Tetramethyl-1,3,2-dioxaborolan-2-yl)propan-2-yl)phenyl 4-methylbenzenesulfonate (**14h**)
^{13}C NMR

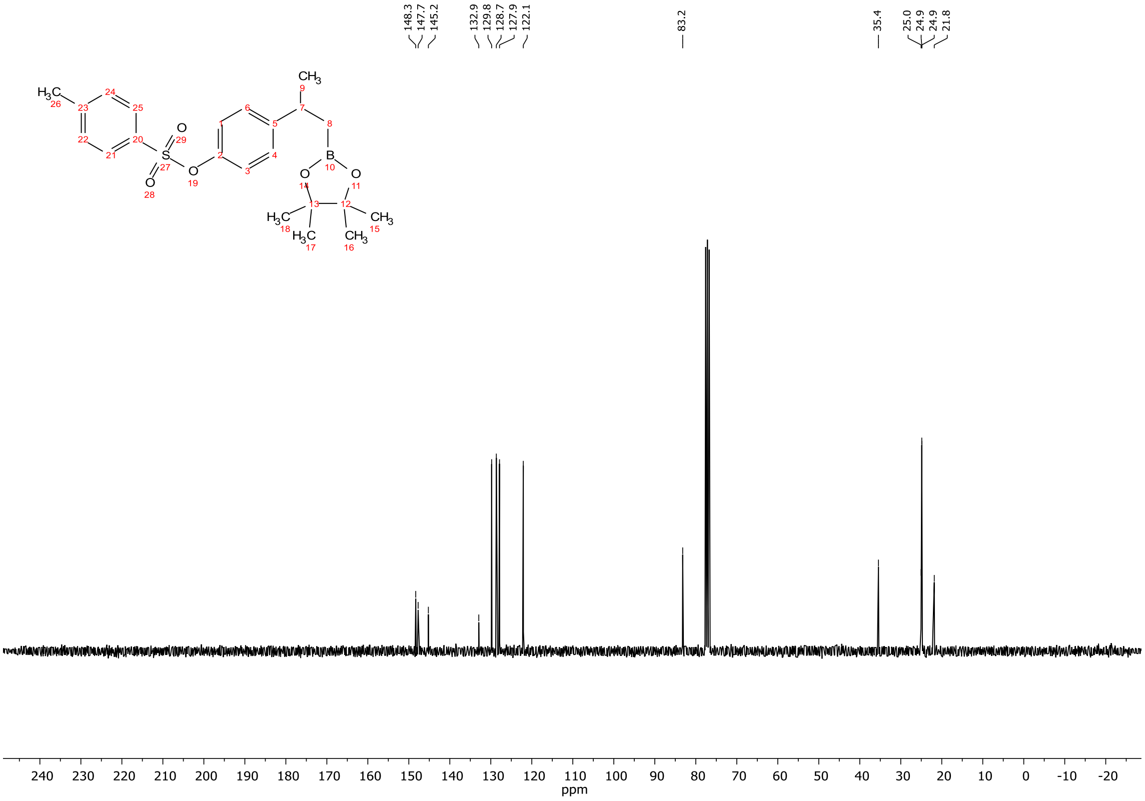

rac-((1*S*,2*S*)-2-Methylcyclohexyl)benzene (**14i**) **^{1}H NMR**

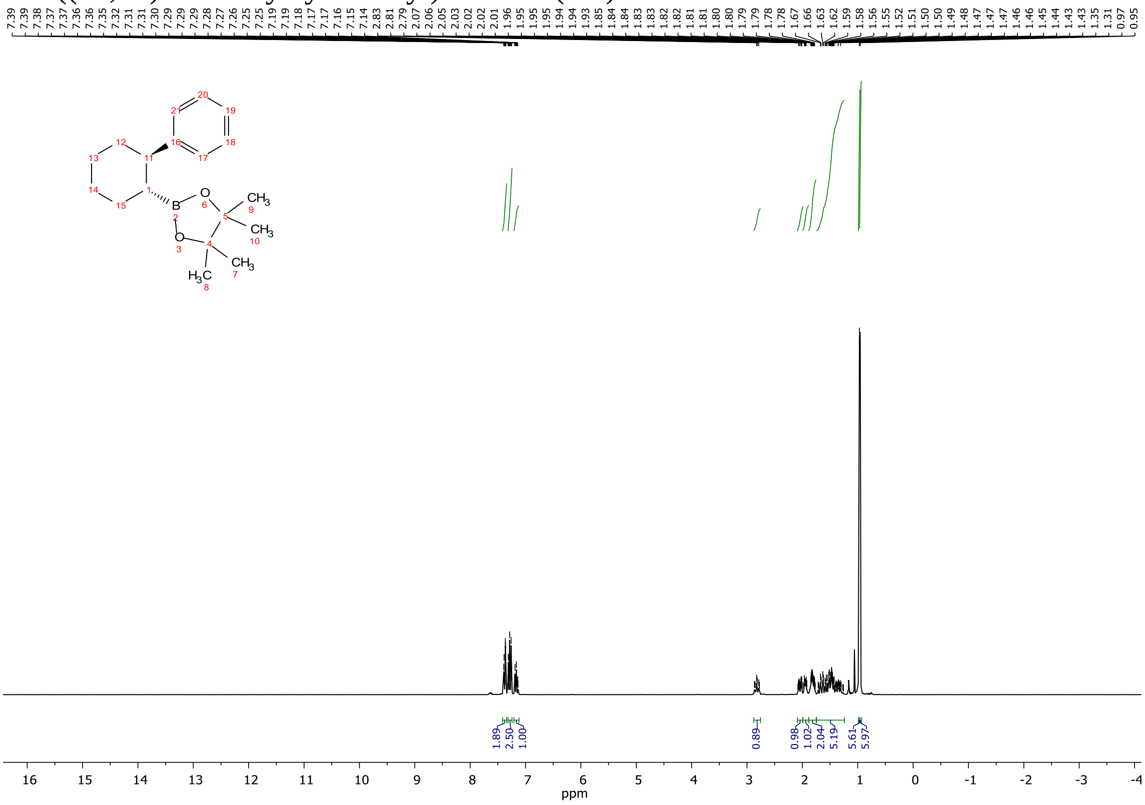

rac-((1*S*,2*S*)-2-Methylcyclohexyl)benzene (**14i**) ^{13}**C NMR**

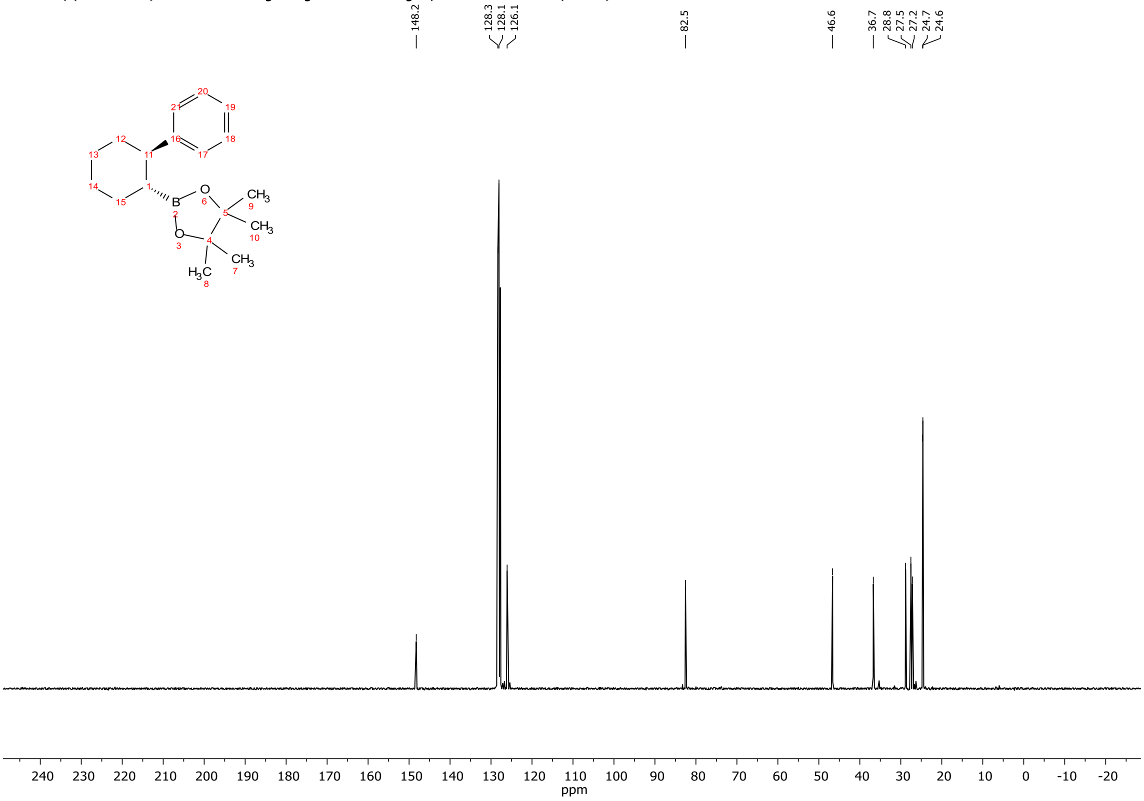

2-((6a*R*,8*S*,9*S*,10a*R*)-1-Methoxy-6,6,9-trimethyl-3-pentyl-6a,7,8,9,10,10a-hexahydro-6*H*-benzo[c]chromen-8-yl)-4,4,5,5-tetramethyl-1,3,2-dioxaborolane (**14j**) ^{1}**H NMR**

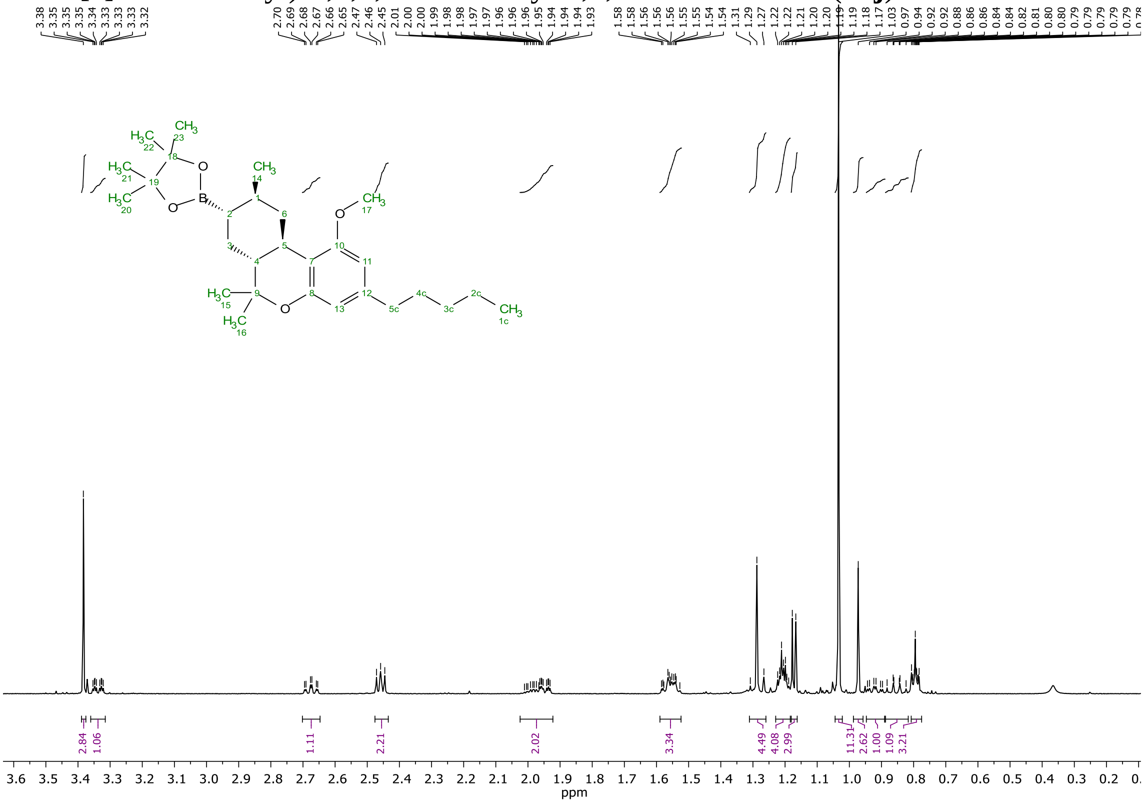

2-((6a*R*,8*S*,9*S*,10a*R*)-1-Methoxy-6,6,9-trimethyl-3-pentyl-6a,7,8,9,10,10a-hexahydro-6*H*-benzo[c]chromen-8-yl)-4,4,5,5-tetramethyl-1,3,2-dioxaborolane (**14j**) **^{1}H NMR**

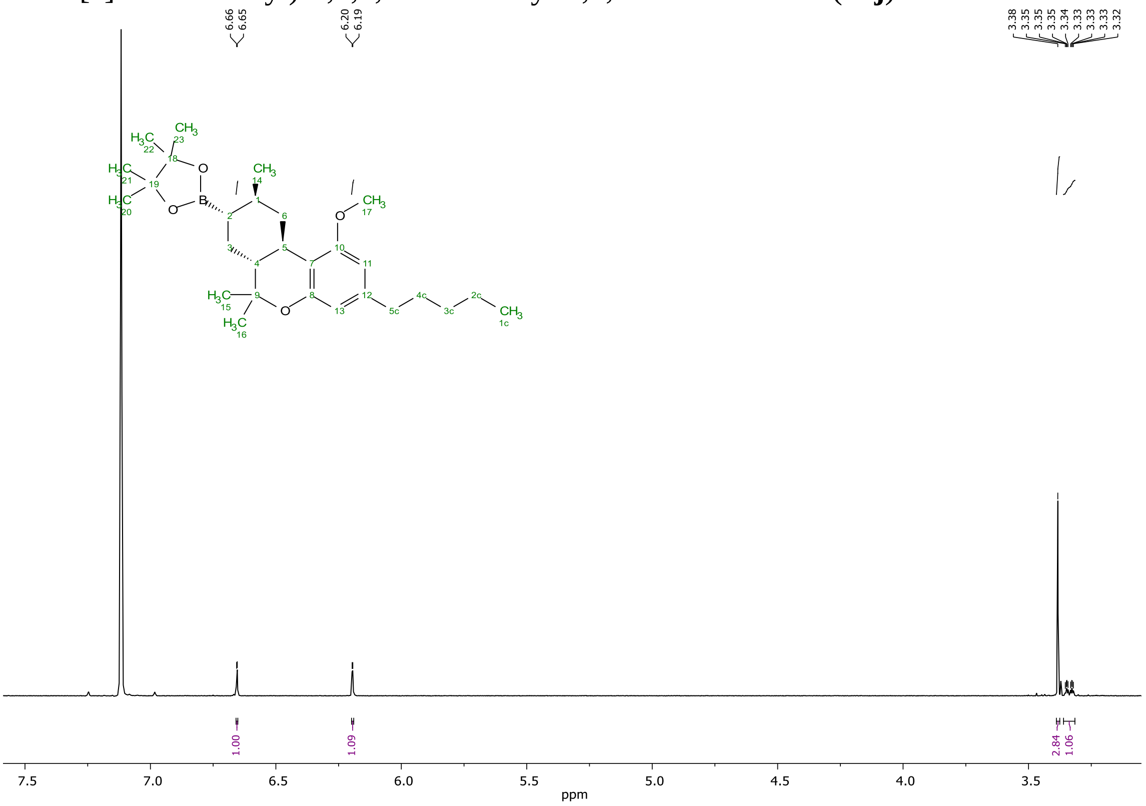

2-((6a*R*,8*S*,9*S*,10a*R*)-1-Methoxy-6,6,9-trimethyl-3-pentyl-6a,7,8,9,10,10a-hexahydro-6*H*-benzo[c]chromen-8-yl)-4,4,5,5-tetramethyl-1,3,2-dioxaborolane (**14j**) **^{1}H NMR**

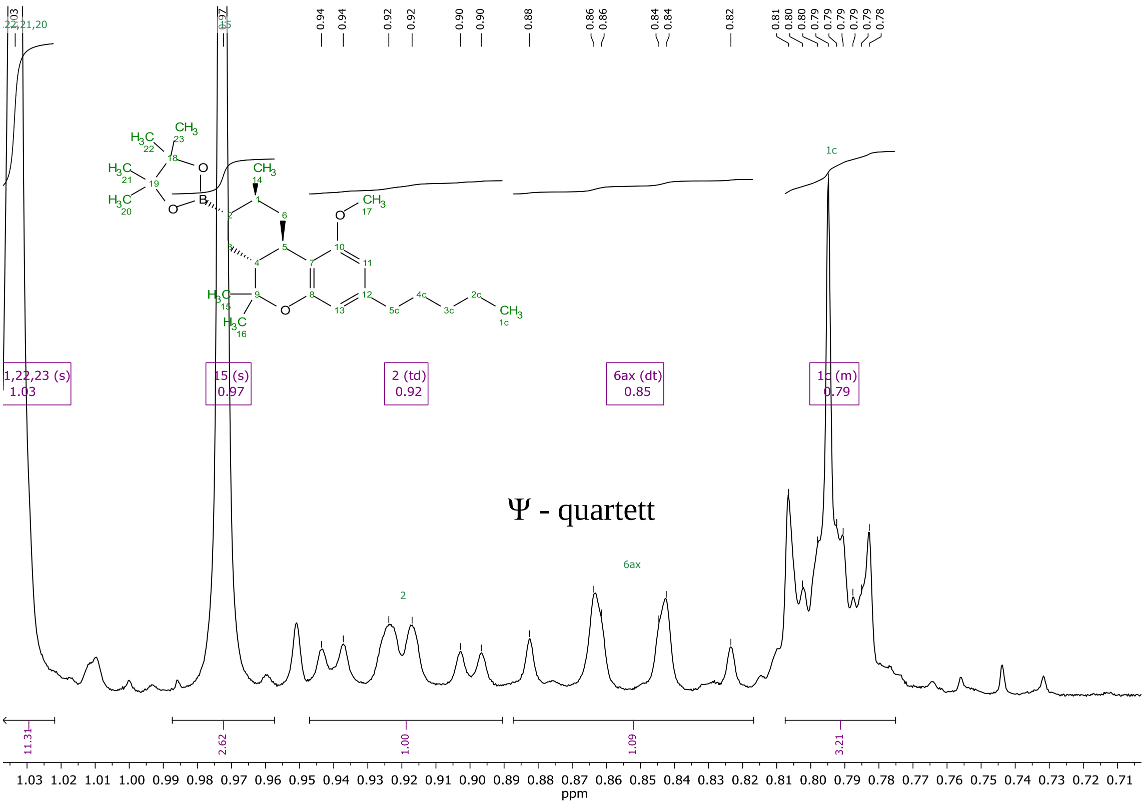

2-((6a*R*,8*S*,9*S*,10a*R*)-1-Methoxy-6,6,9-trimethyl-3-pentyl-6a,7,8,9,10,10a-hexahydro-6*H*-benzo[c]chromen-8-yl)-4,4,5,5-tetramethyl-1,3,2-dioxaborolane (**14j**) **^{13}C NMR**

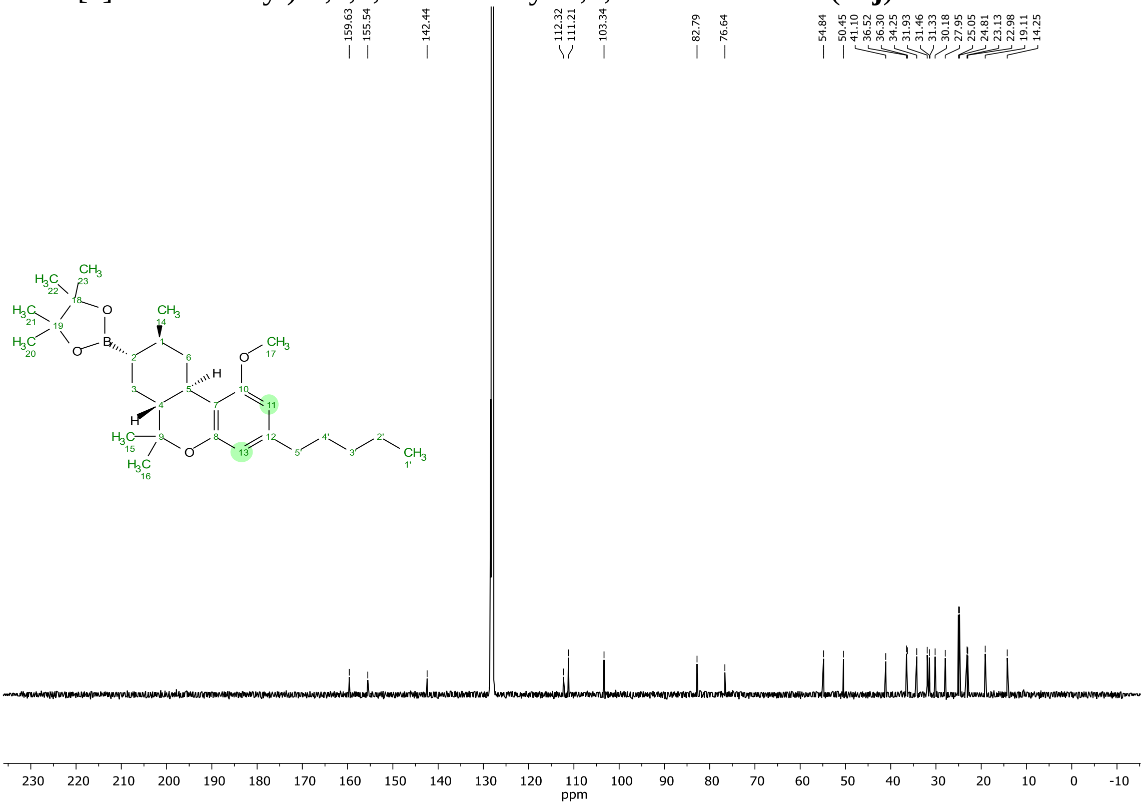

2-((3*S*,5*R*,6*S*,8*S*,9*S*,10*R*,13*R*,14*S*,17*R*)-3-Methoxy-5,10,13-trimethyl-17-((*R*)-6-methylheptan-2-yl)hexadecahydro-1*H*-cyclopenta[a]phenanthren-6-yl)-4,4,5,5-tetramethyl-1,3,2-dioxaborolane (**14k**) **^{1}H NMR**

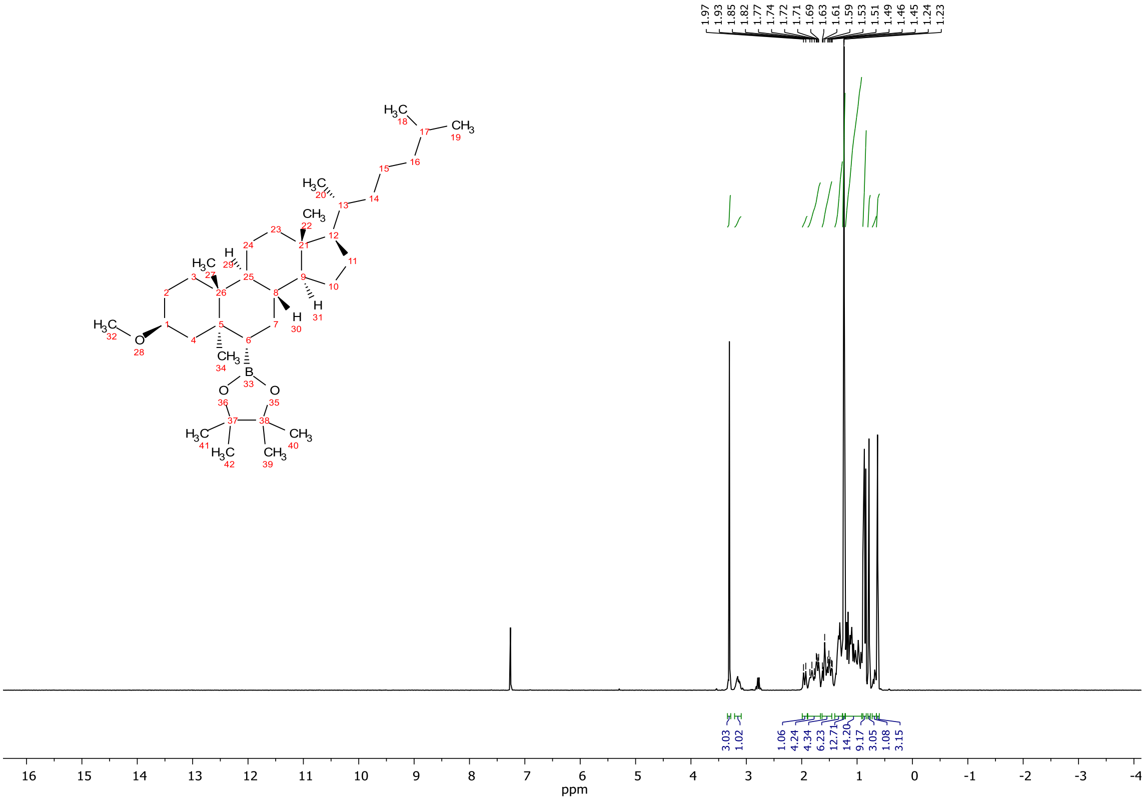

2-((3*S*,5*R*,6*S*,8*S*,9*S*,10*R*,13*R*,14*S*,17*R*)-3-Methoxy-5,10,13-trimethyl-17-((*R*)-6-methylheptan-2-yl)hexadecahydro-1*H*-cyclopenta[a]phenanthren-6-yl)-4,4,5,5-tetramethyl-1,3,2-dioxaborolane (**14k**) **^{13}C NMR**

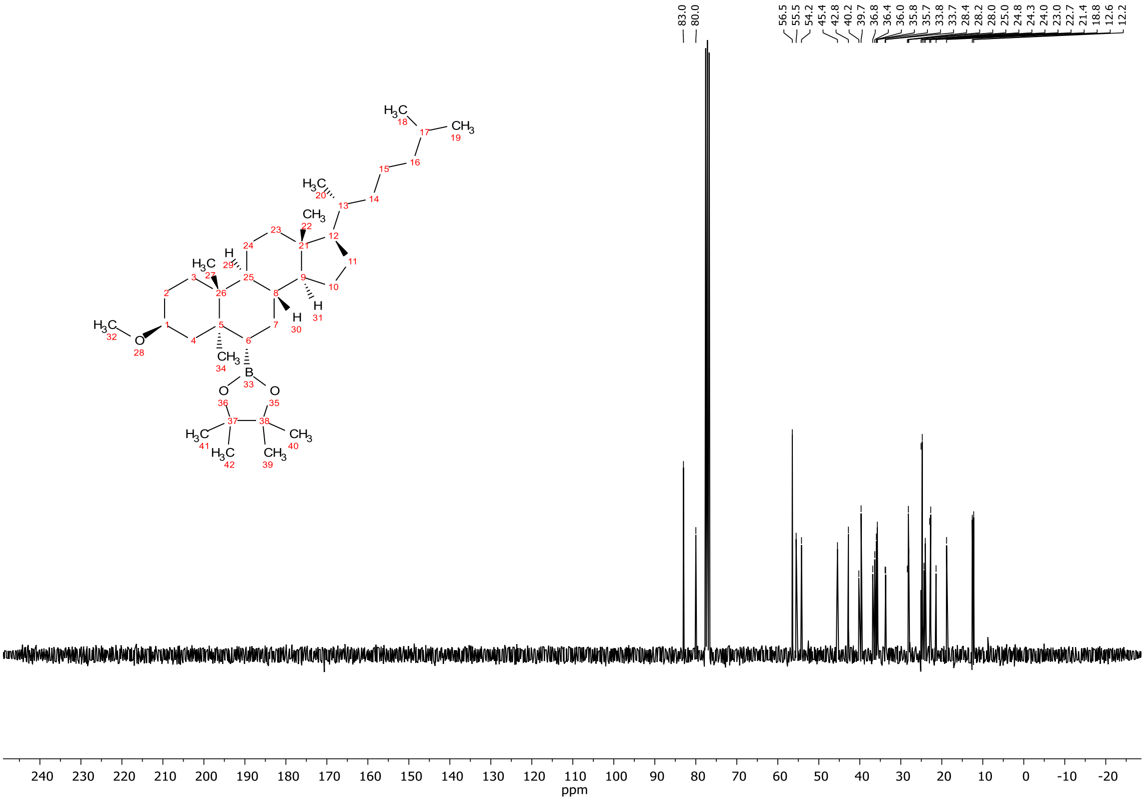

3.2.3 Protodeboronation reactions

1-Methoxy-4-propylbenzene (**2b**) **^{1}H NMR**

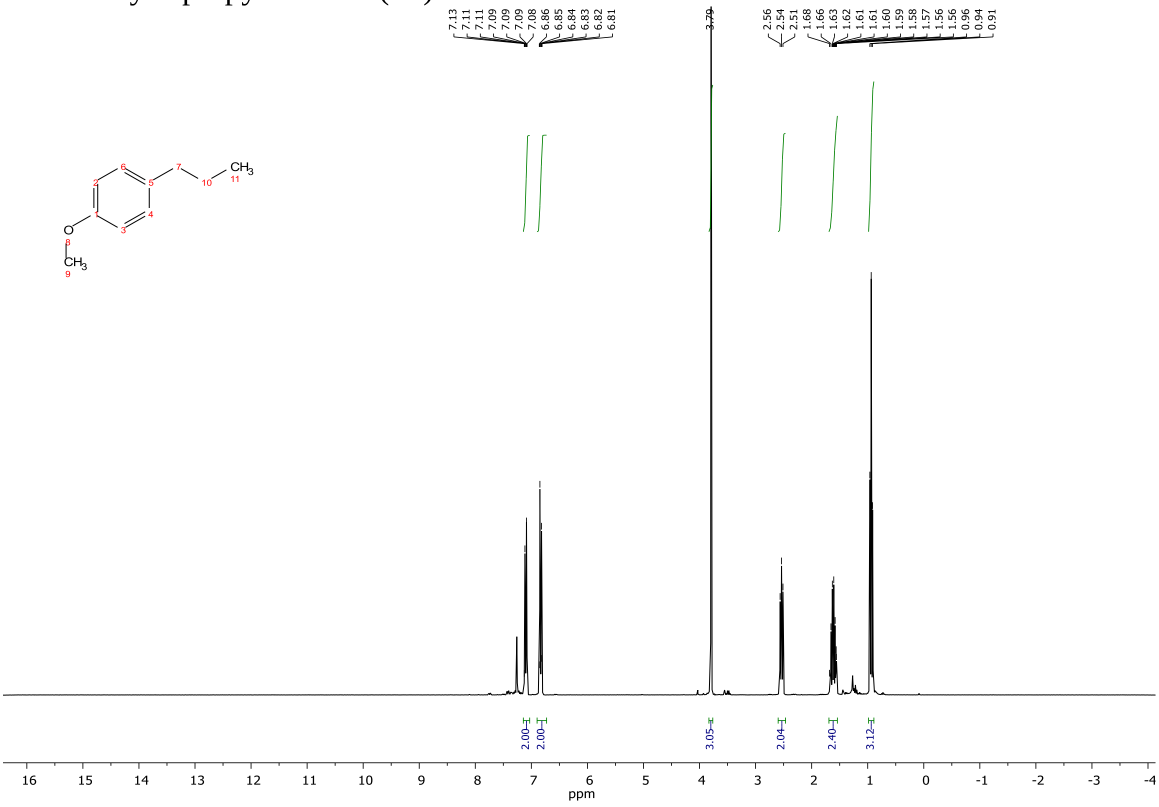

1-Methoxy-4-propylbenzene (**2b**) **^{13}C NMR**

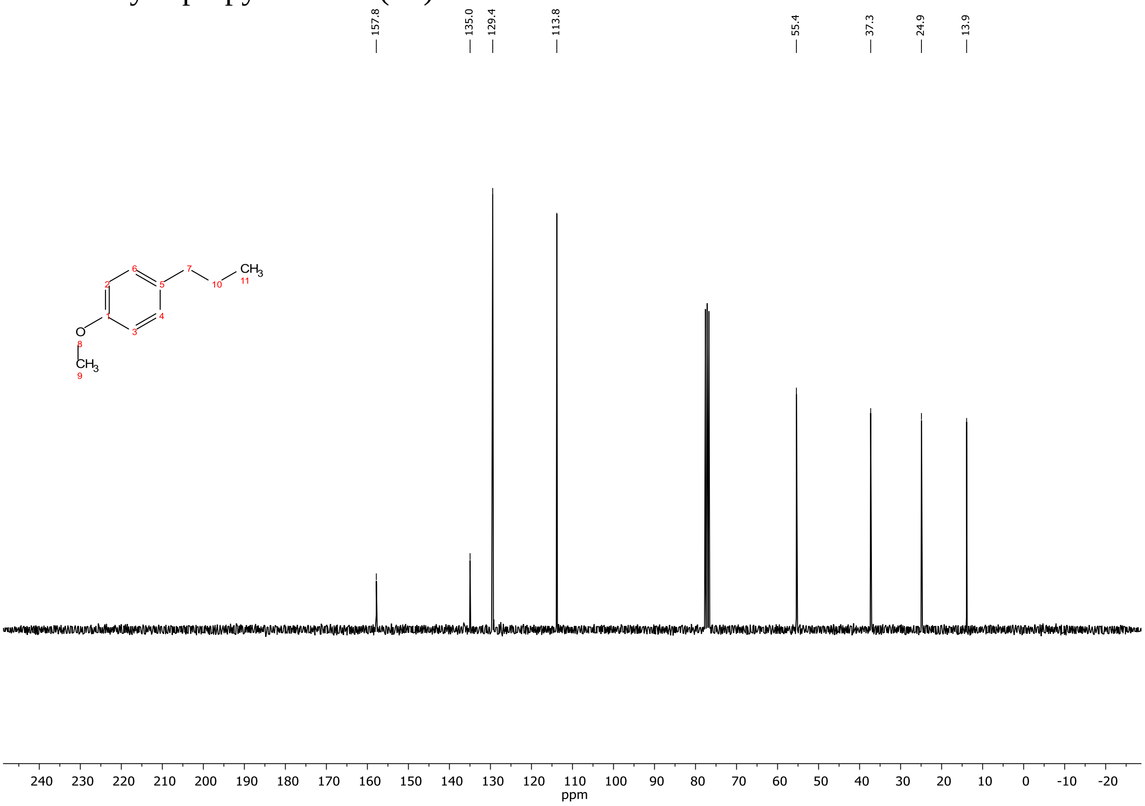

1,2-Dimethoxy-4-propylbenzene (**2c**) **^{1}H NMR**

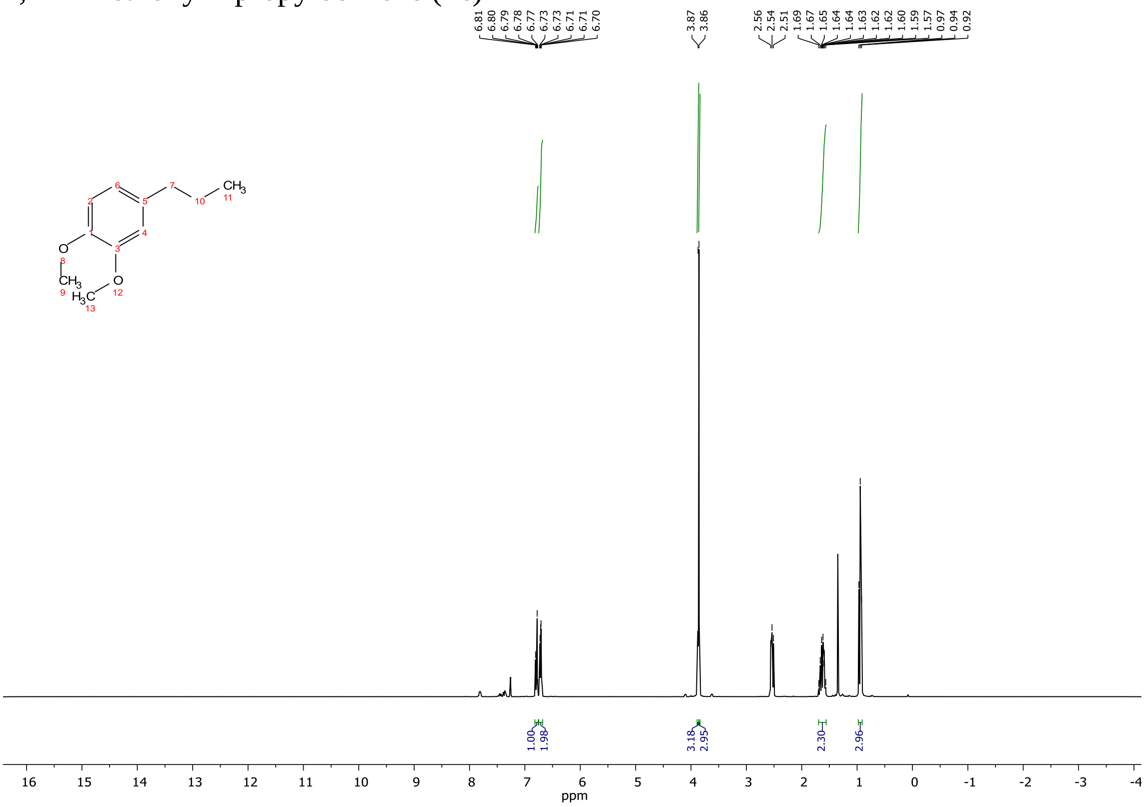

1,2-Dimethoxy-4-propylbenzene (**2c**) ^{13}C NMR

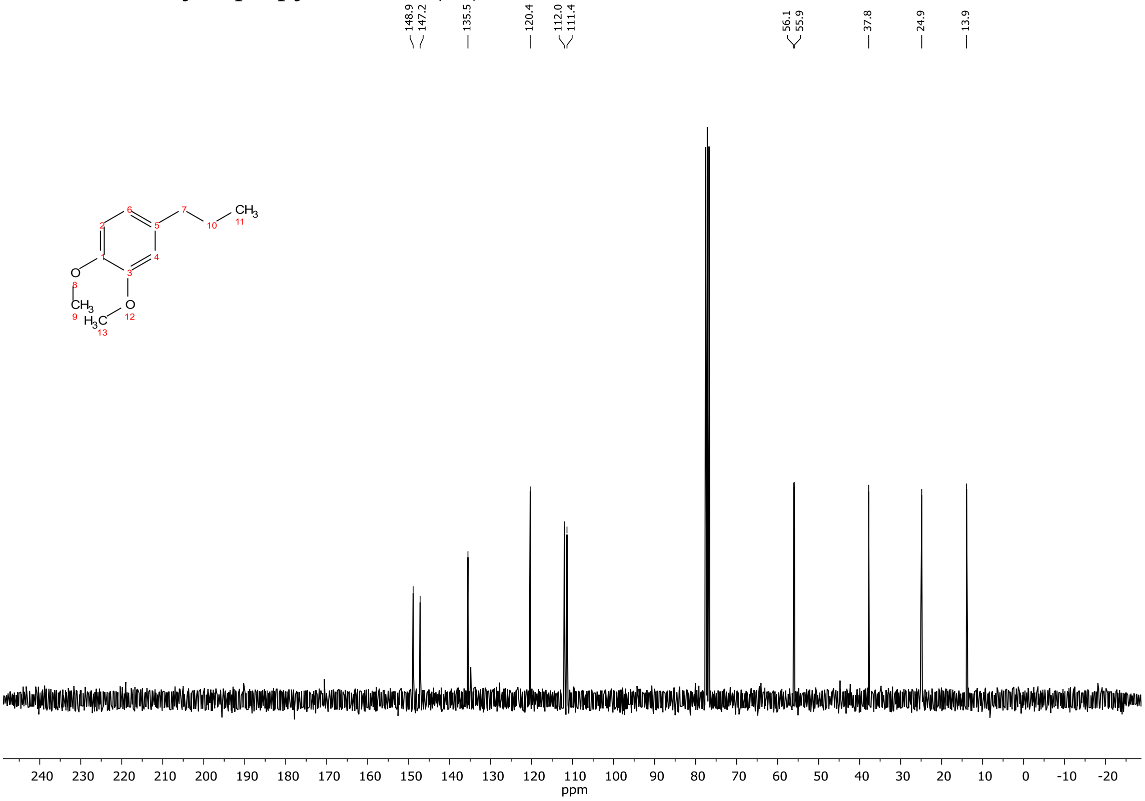

4-Isopropyl-1,1'-biphenyl (**2d**) ^{1}H NMR

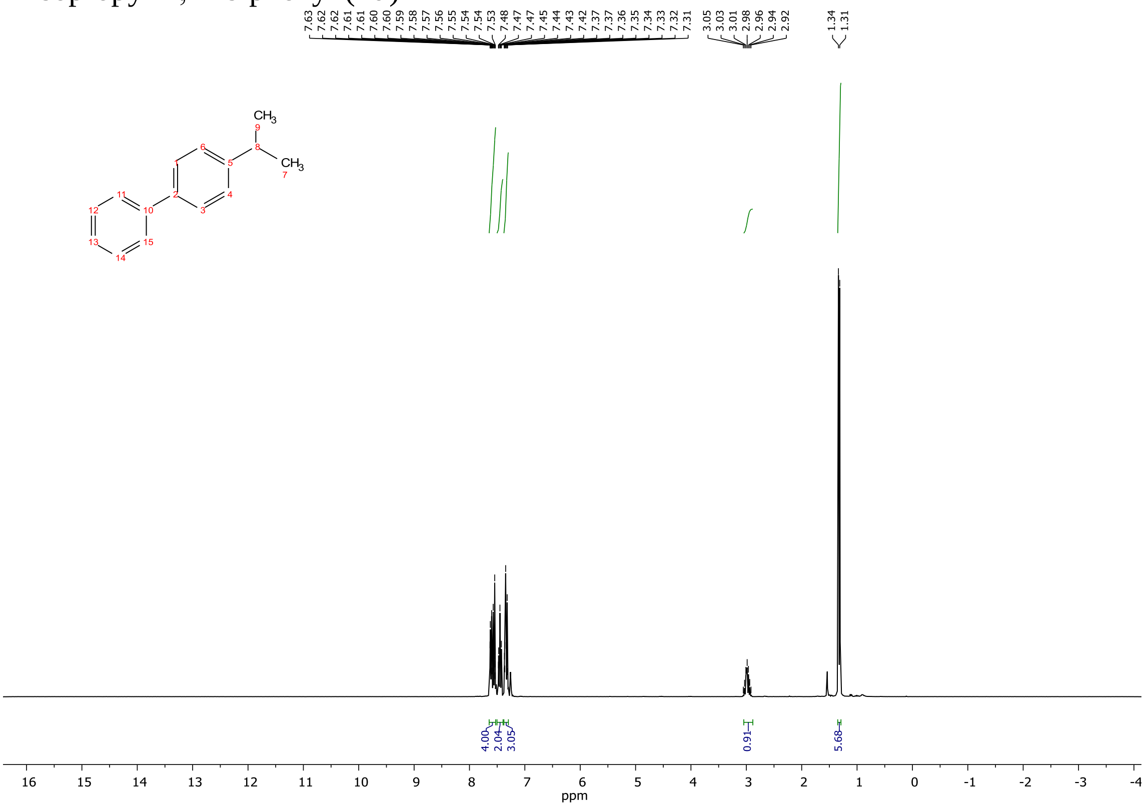

4-Isopropyl-1,1'-biphenyl (**2d**) ^{13}C **NMR**

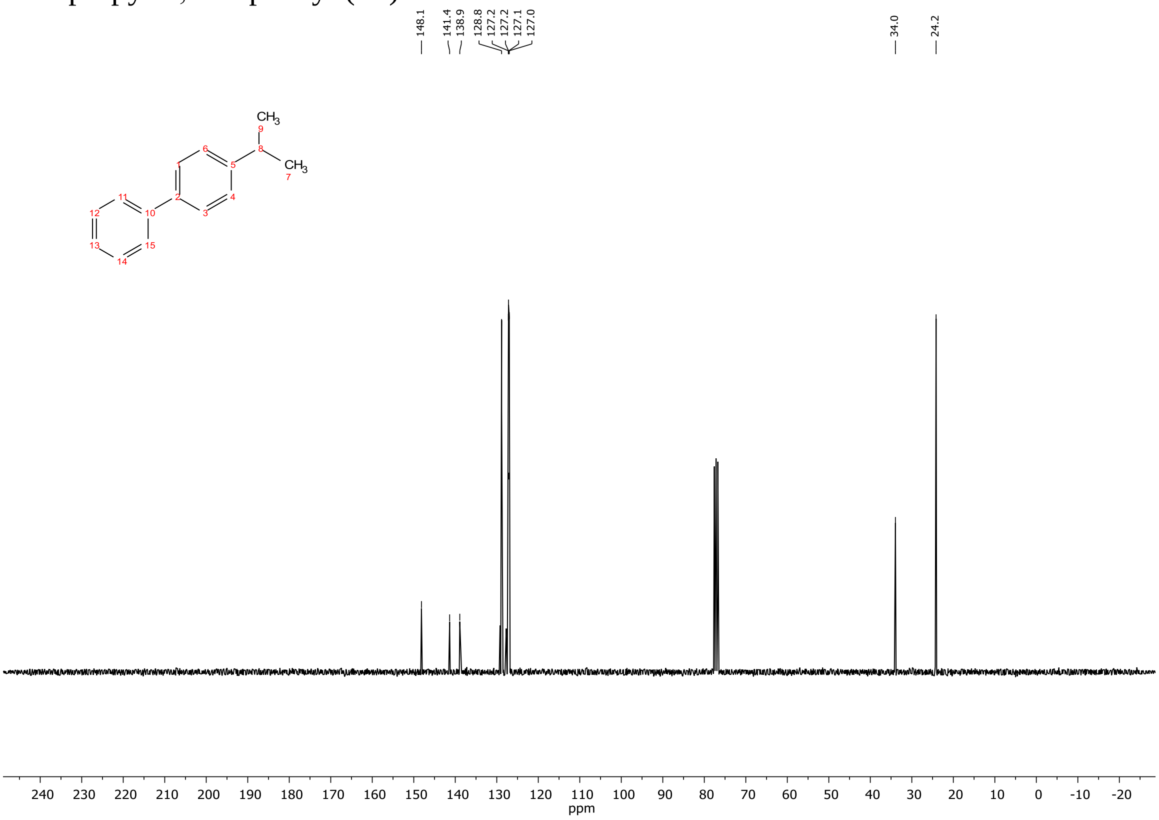

1-(*tert*-Butyl)-4-isopropylbenzene (**2e**) ^{1}H **NMR**

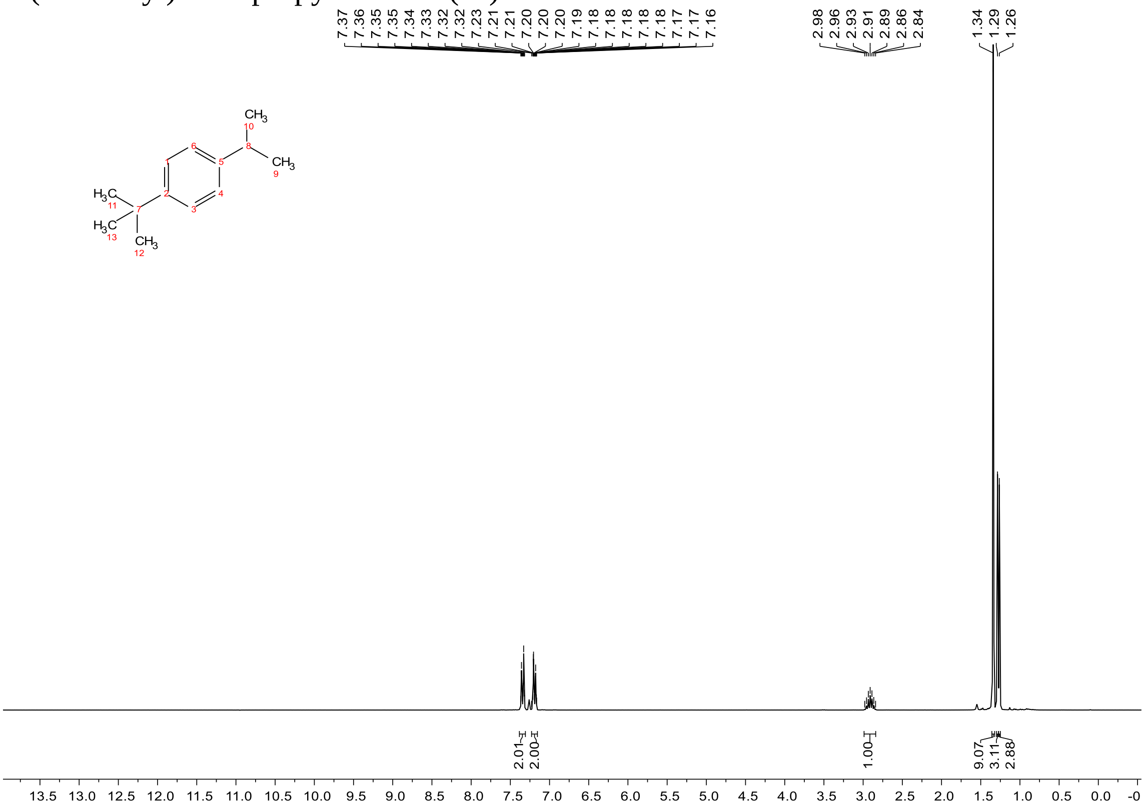

1-(*tert*-Butyl)-4-isopropylbenzene (**2e**) ^{13}C **NMR**

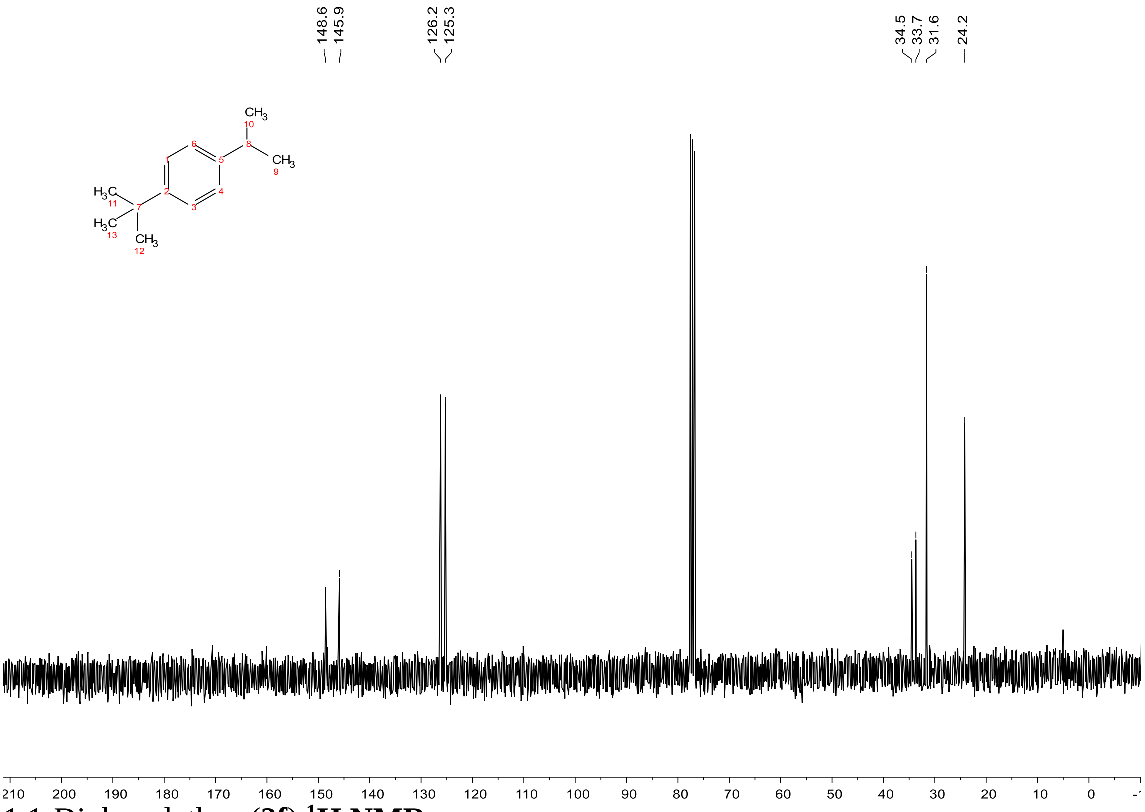

1,1-Diphenylethan (**2f**) ^{1}**H NMR**

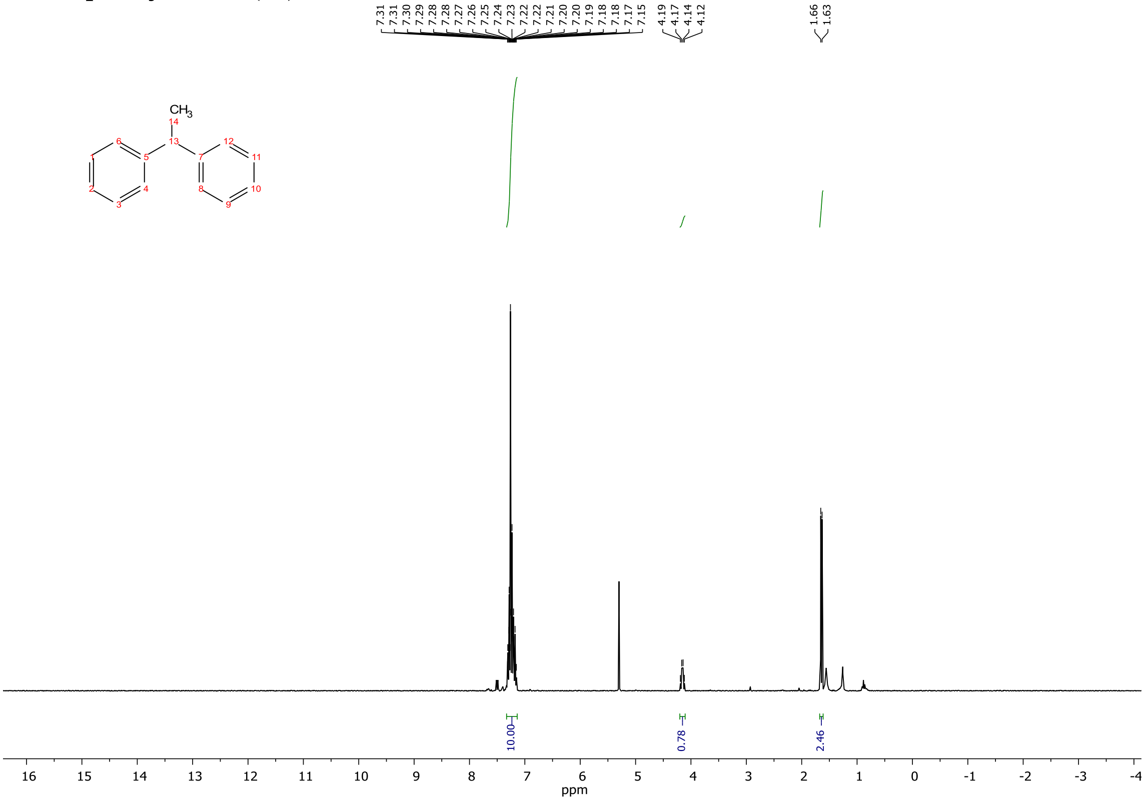

1,1-Diphenylethan (**2f**) ^{13}**C NMR**

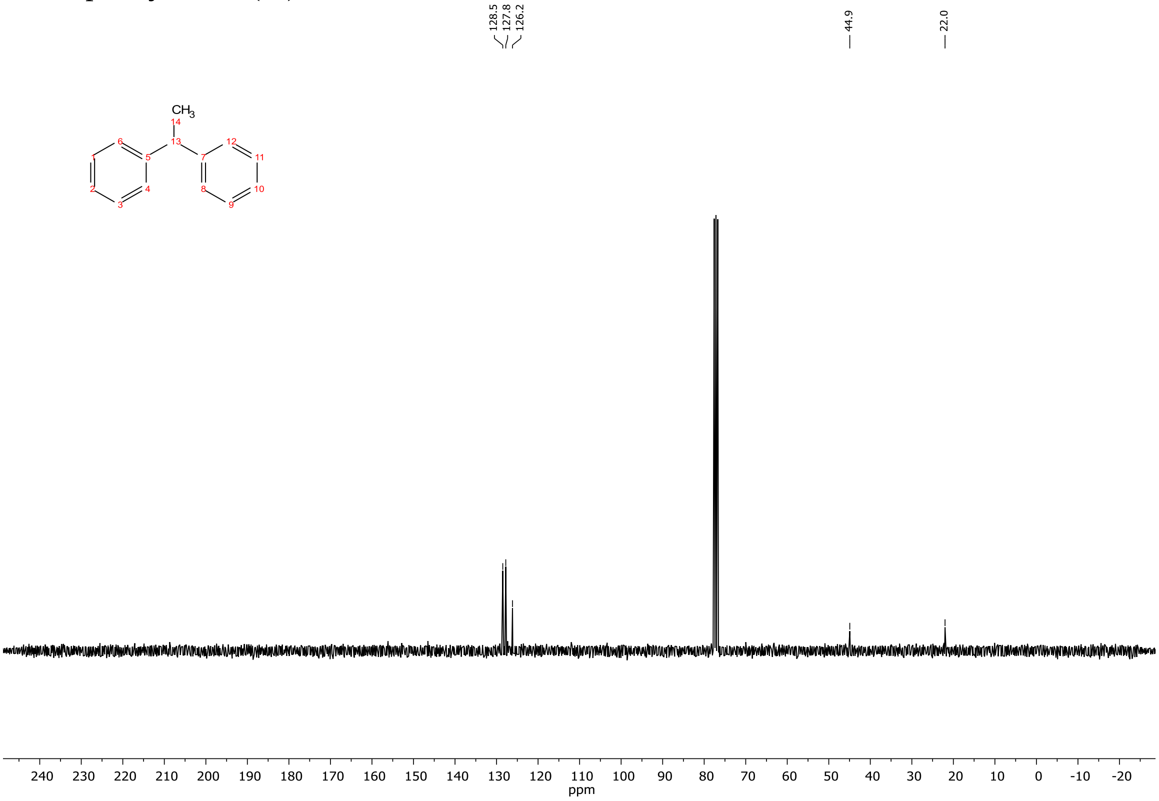

1,1-Bis(4-methoxyphenyl)ethan (**2g**) ^{1}**H NMR**

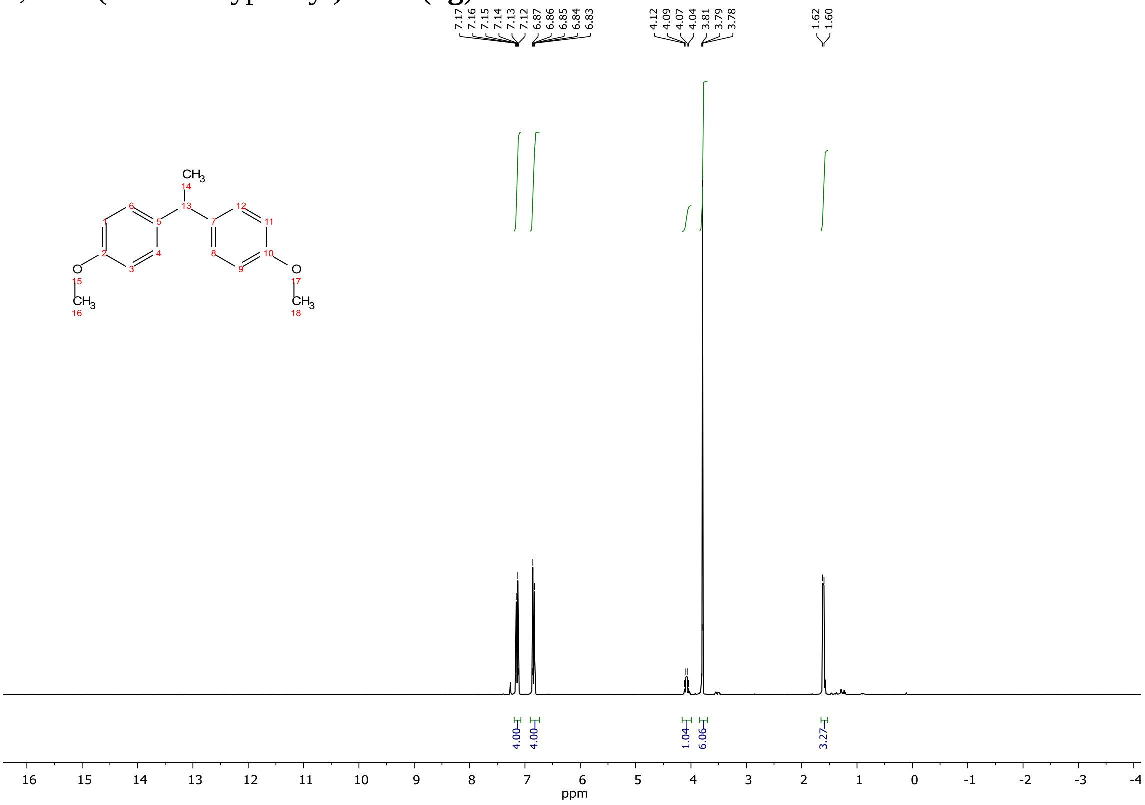

1,1-Bis(4-methoxyphenyl)ethan (**2g**) **^{1}H NMR**

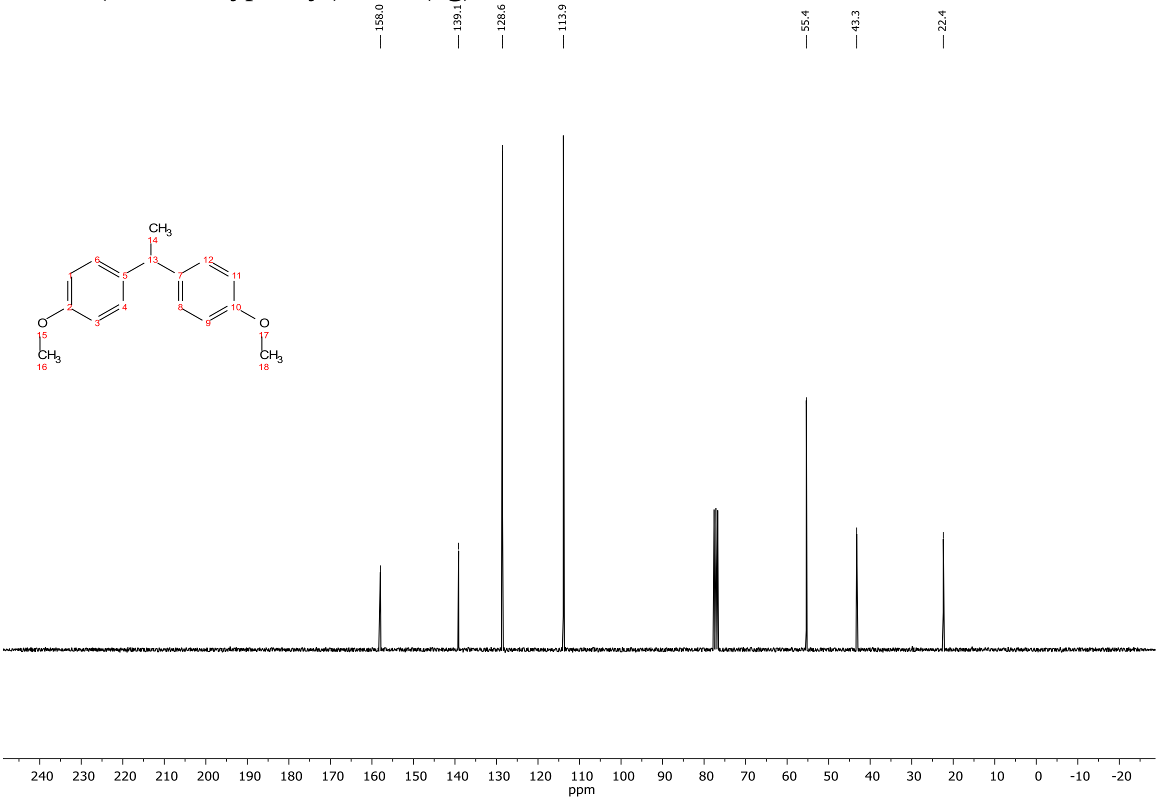

2-Ethylnaphthalene (**2h**) **^{1}H NMR**

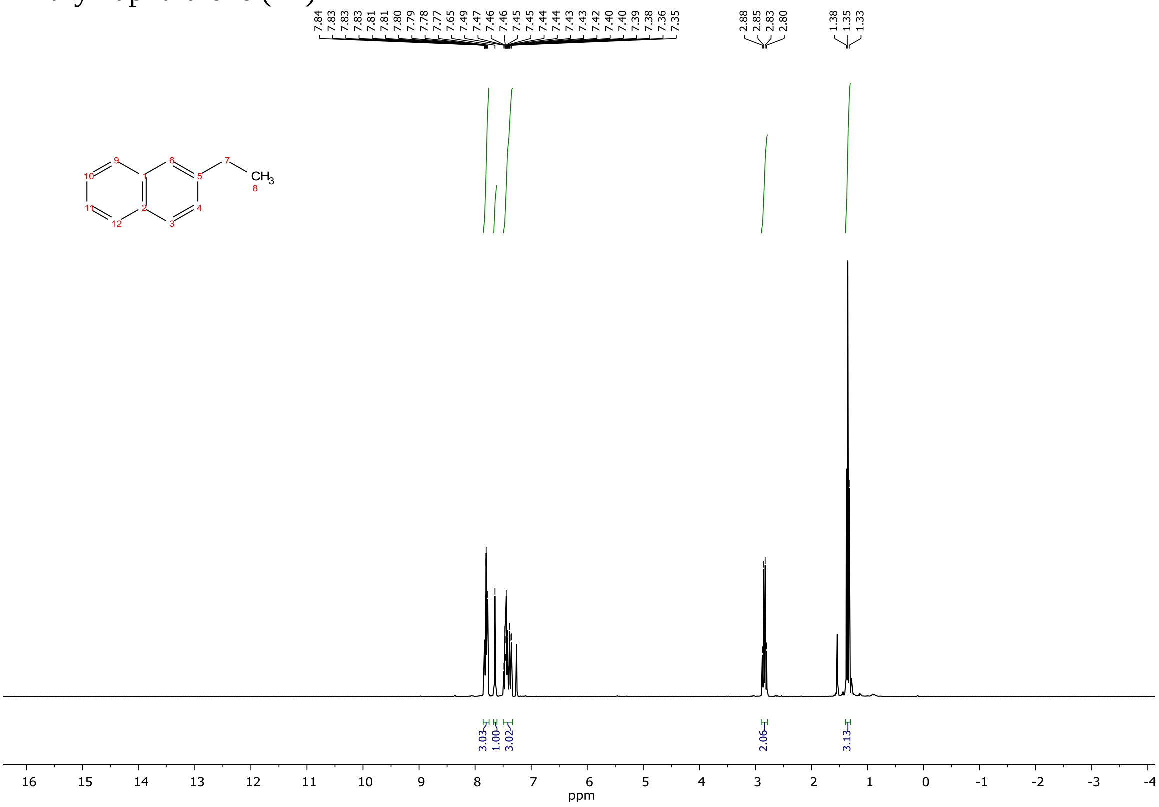

2-Ethylnaphthalene (**2h**) **^{13}C NMR**

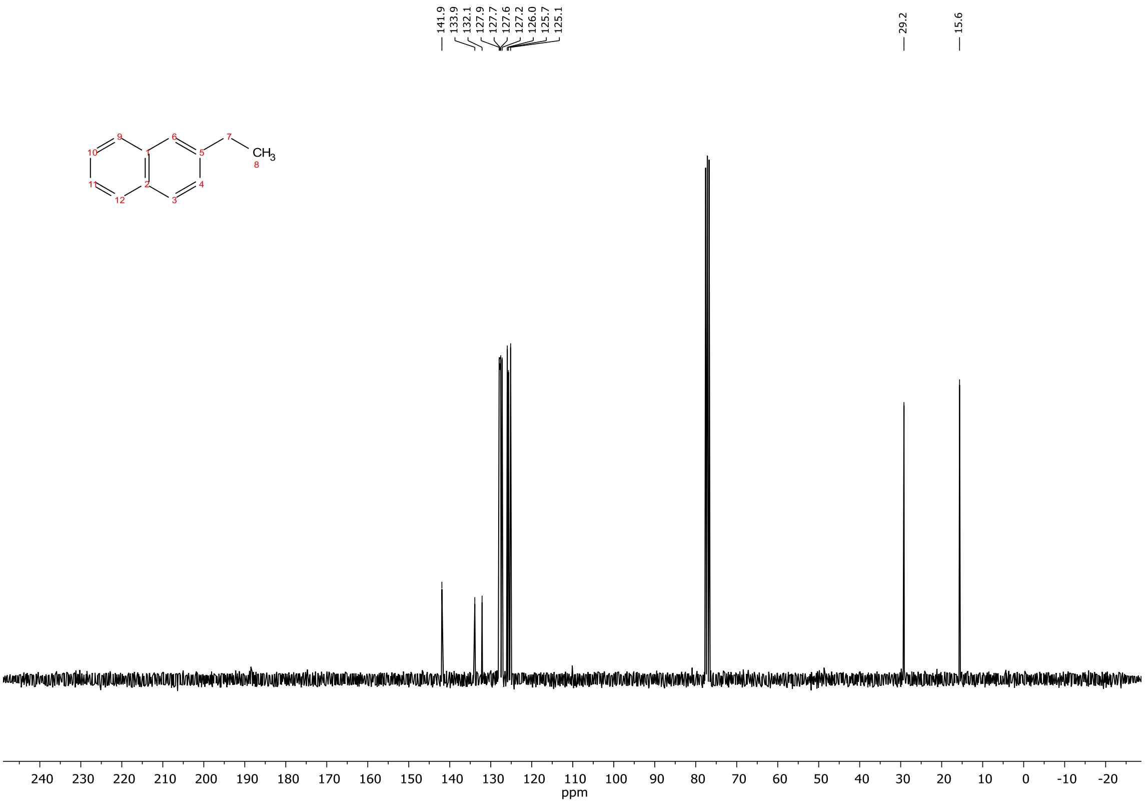

Triphenylpropylsilane (**2i**) ^{1}H NMR

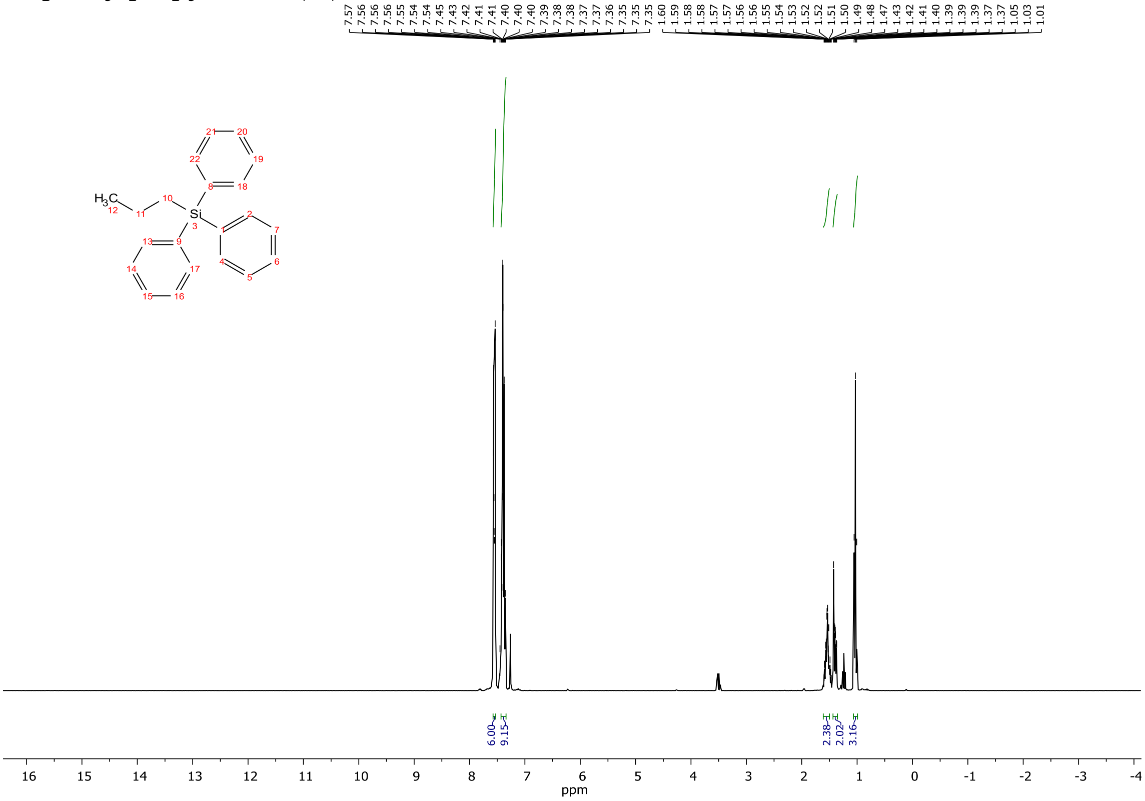

Triphenylpropylsilane (**2i**) **^{13}C NMR**

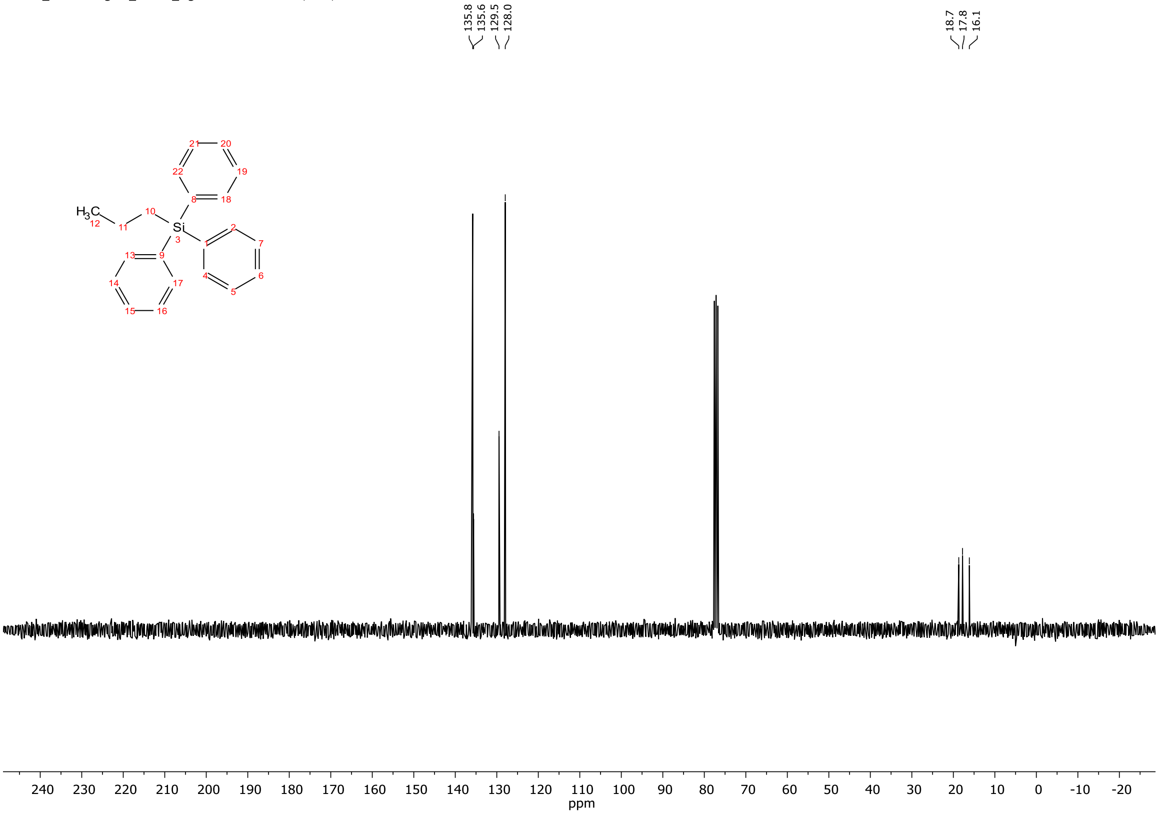

N-Methylcarbazole (**2j**) **^{1}H NMR**

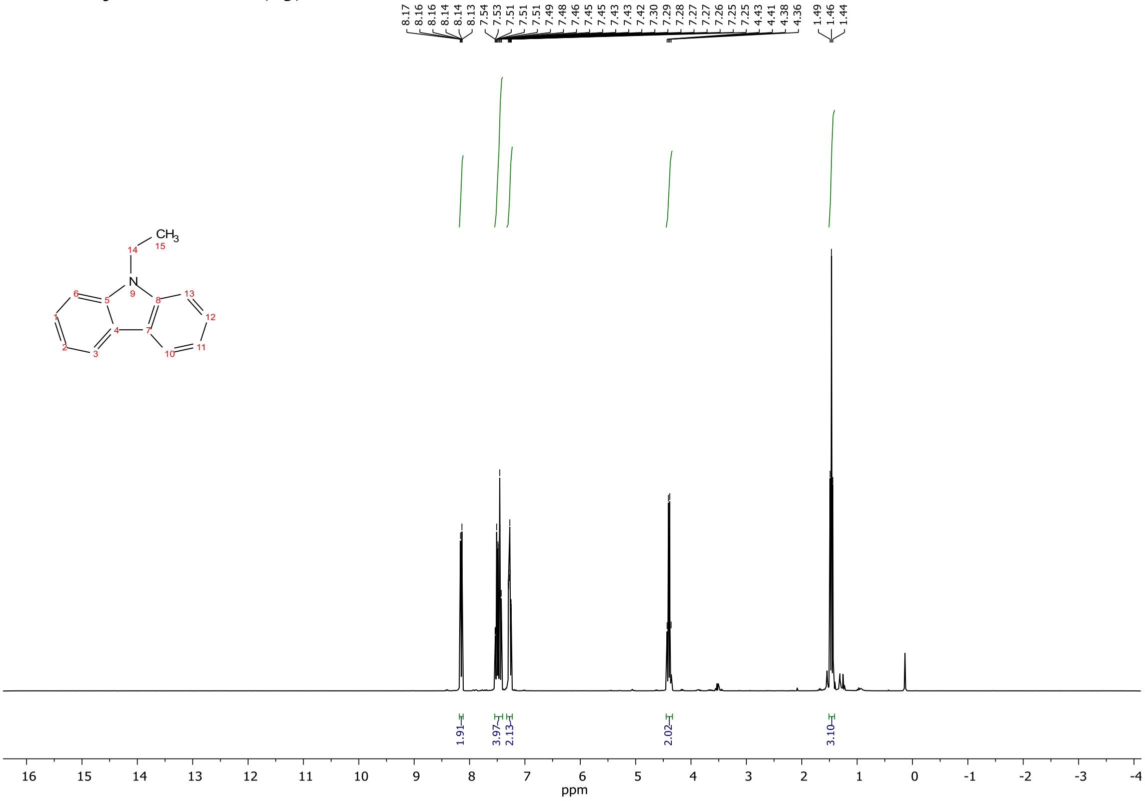

N-Methylcarbazole (**2j**) ^{13}C NMR

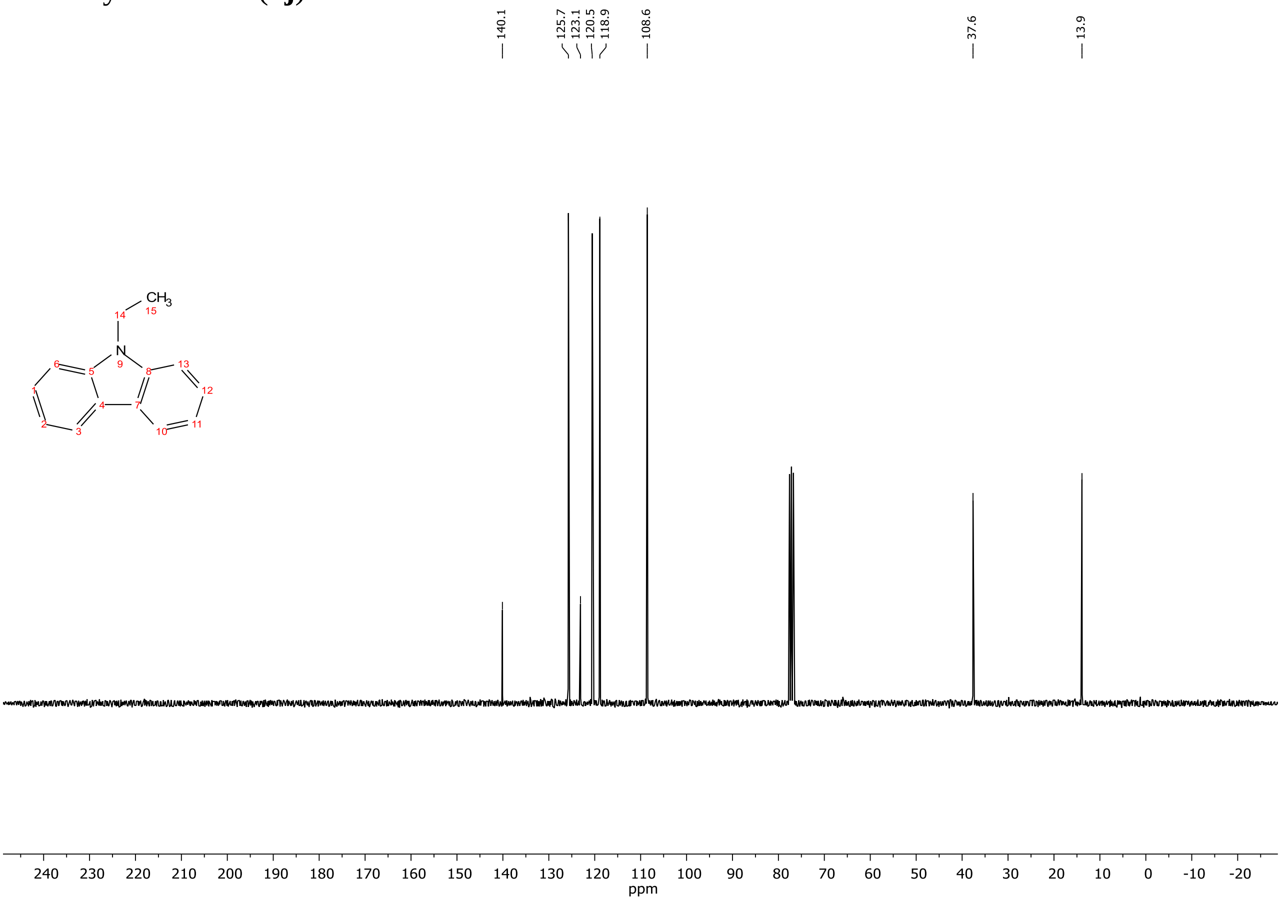

1-Methoxy-4-(pentan-2-yl)benzene (**2k**) ^{1}H NMR

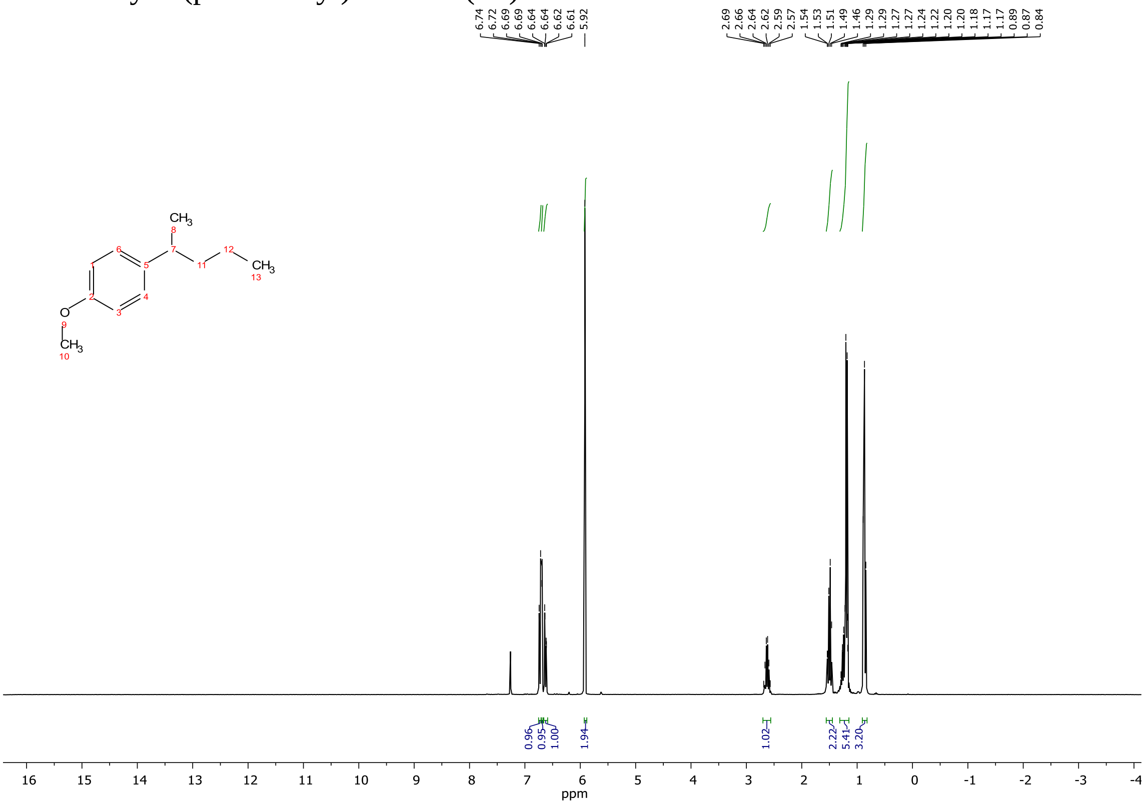

1-Methoxy-4-(pentan-2-yl)benzene (**2k**) ^{13}C NMR

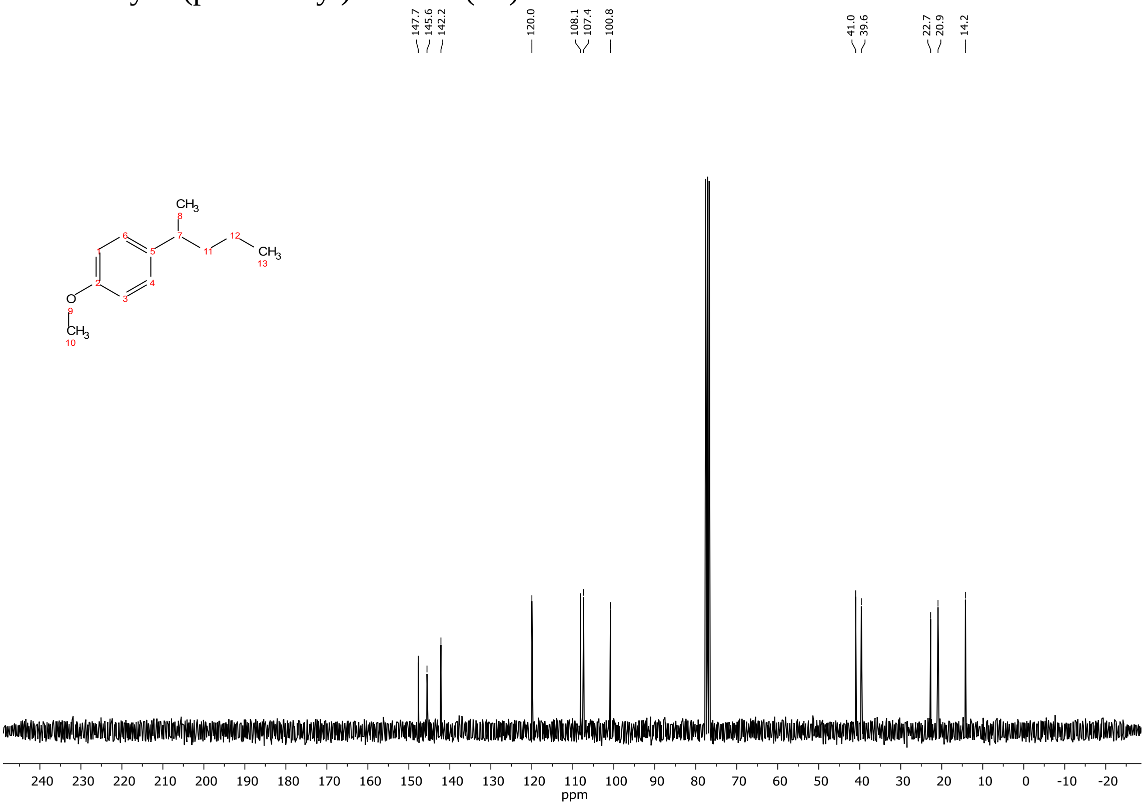

Ethylphenylsulfone (**2l**) ^{1}H NMR

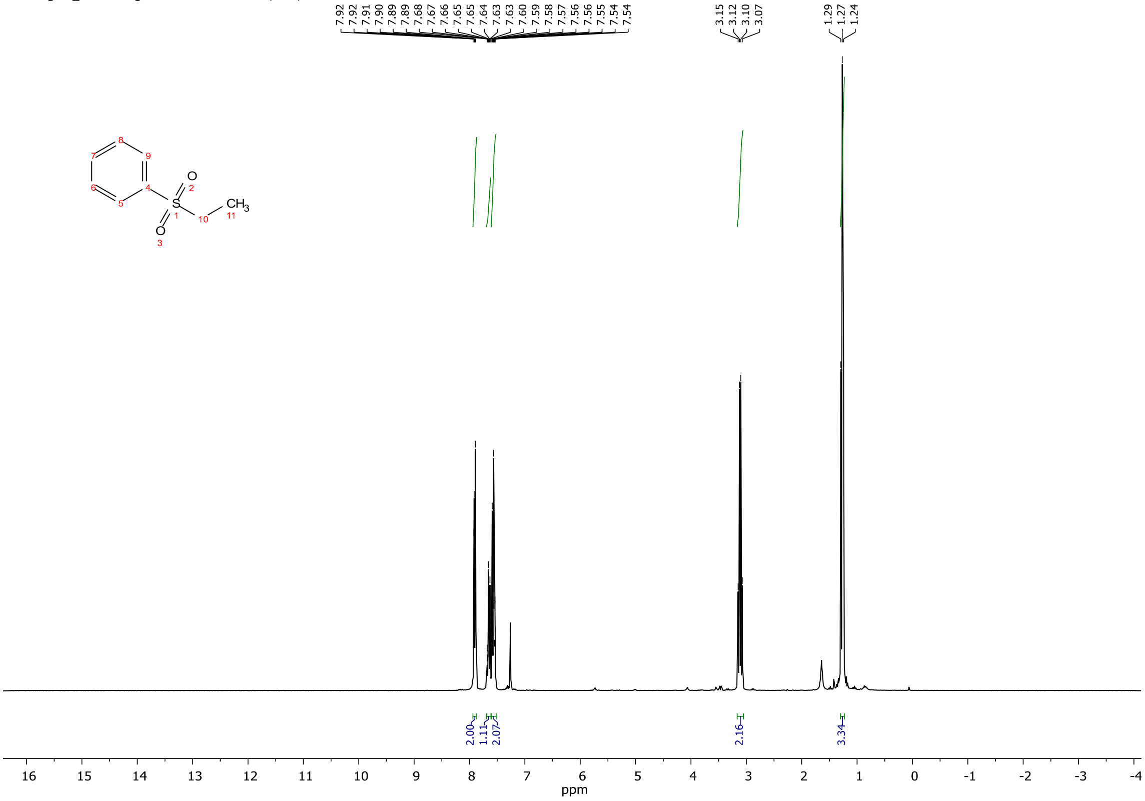

Ethylphenylsulfone (**2l**) ^{13}C NMR

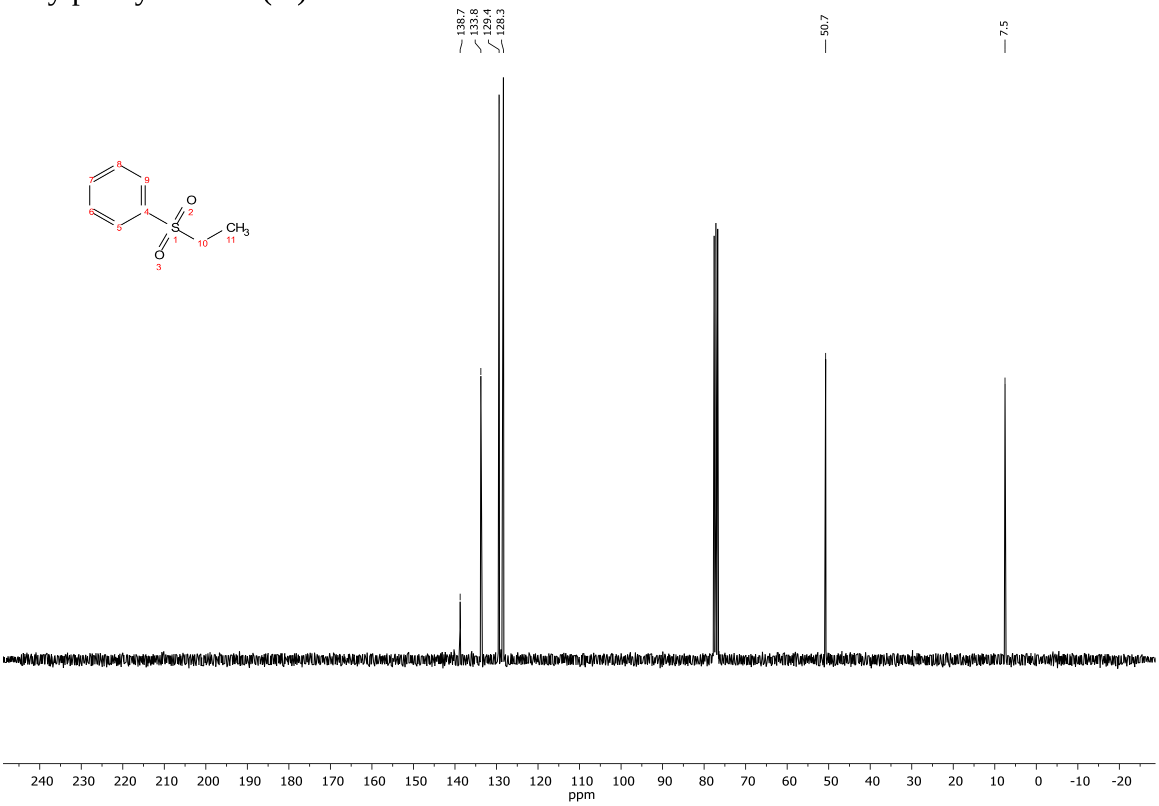

tert-Butyl pyrrolidine-1-carboxylate (**2m**) ^{1}H NMR

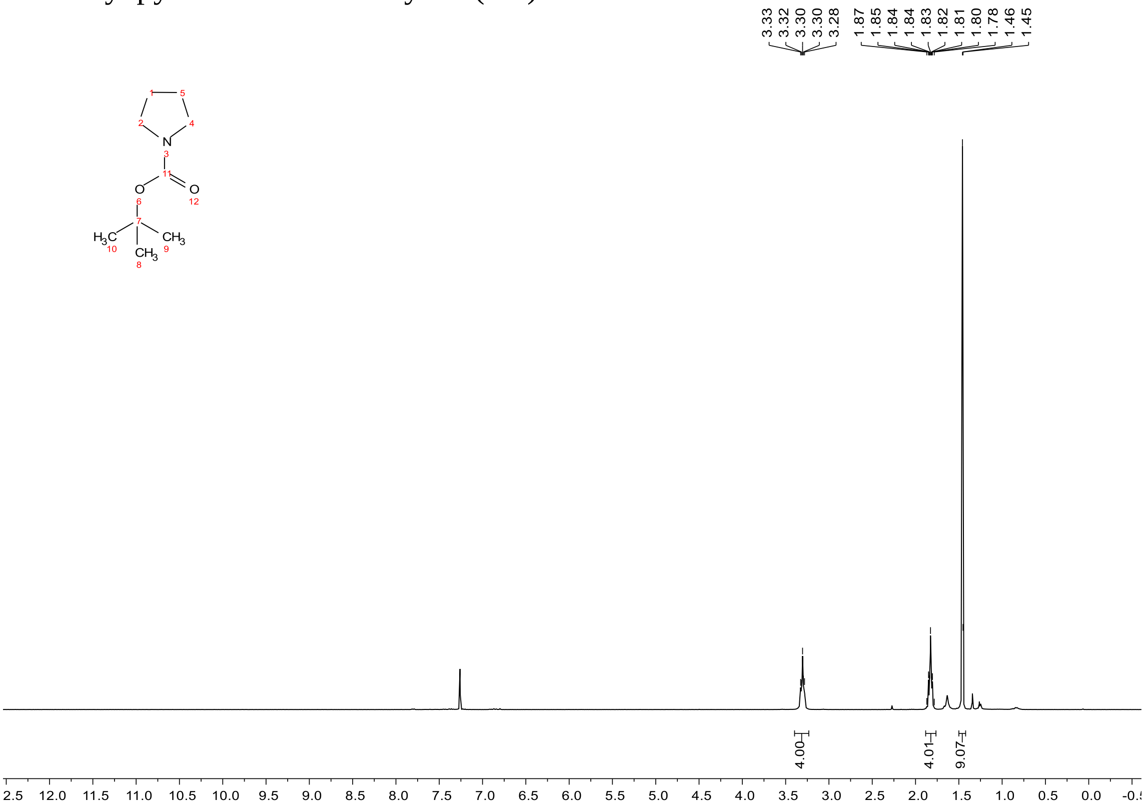

tert-Butyl pyrrolidine-1-carboxylate (**2m**) ^{13}C NMR

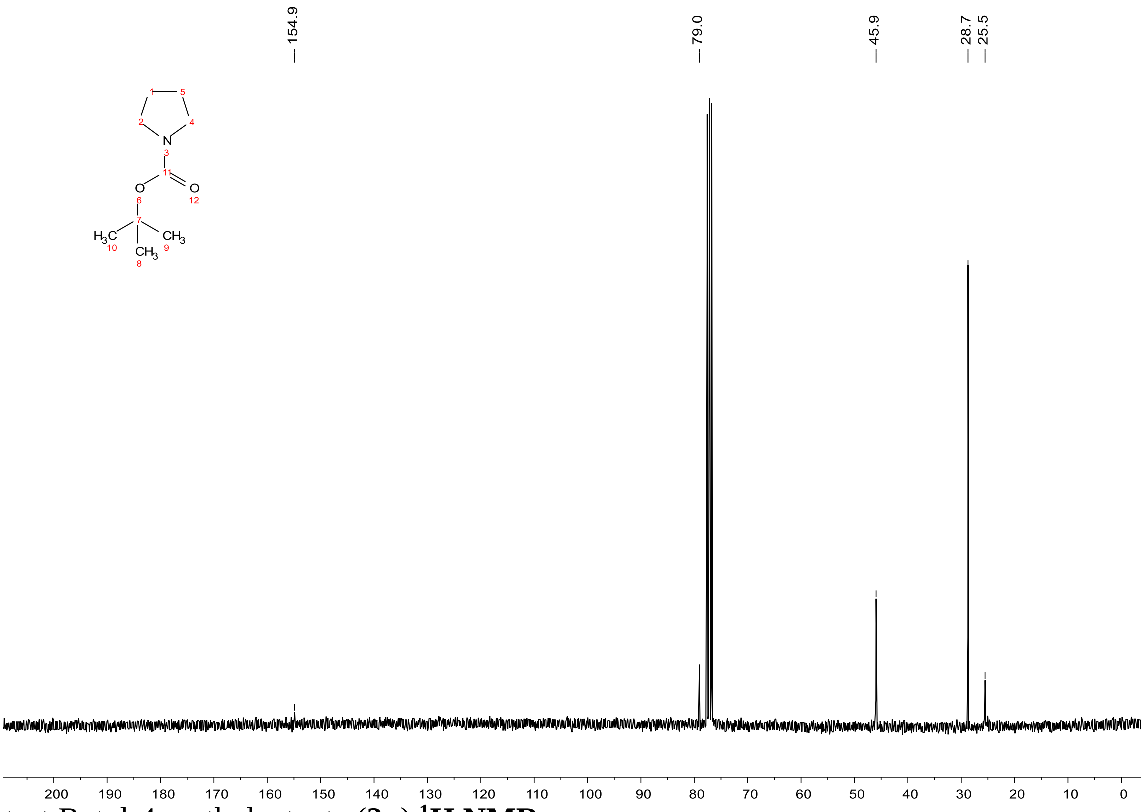

tert-Butyl-4-methyloctoate (**2n**) **^{1}H NMR**

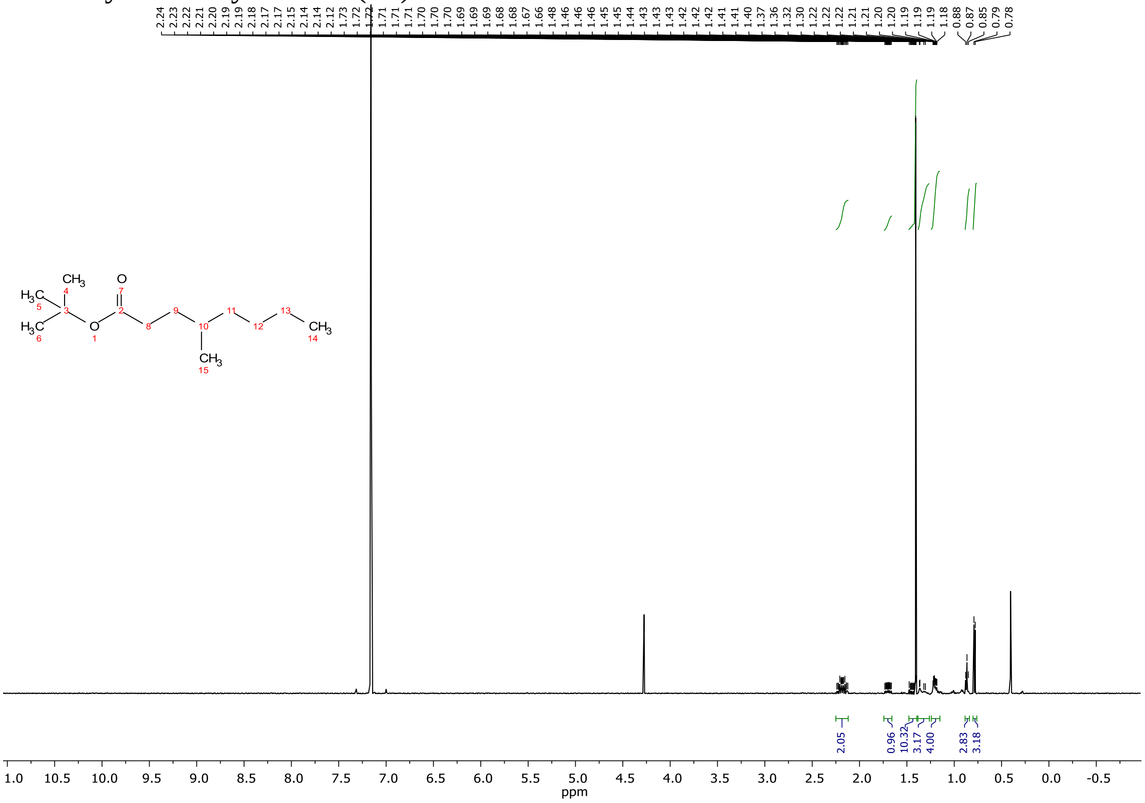

tert-Butyl-4-methyloctoate (**2n**) ^{13}**C NMR**

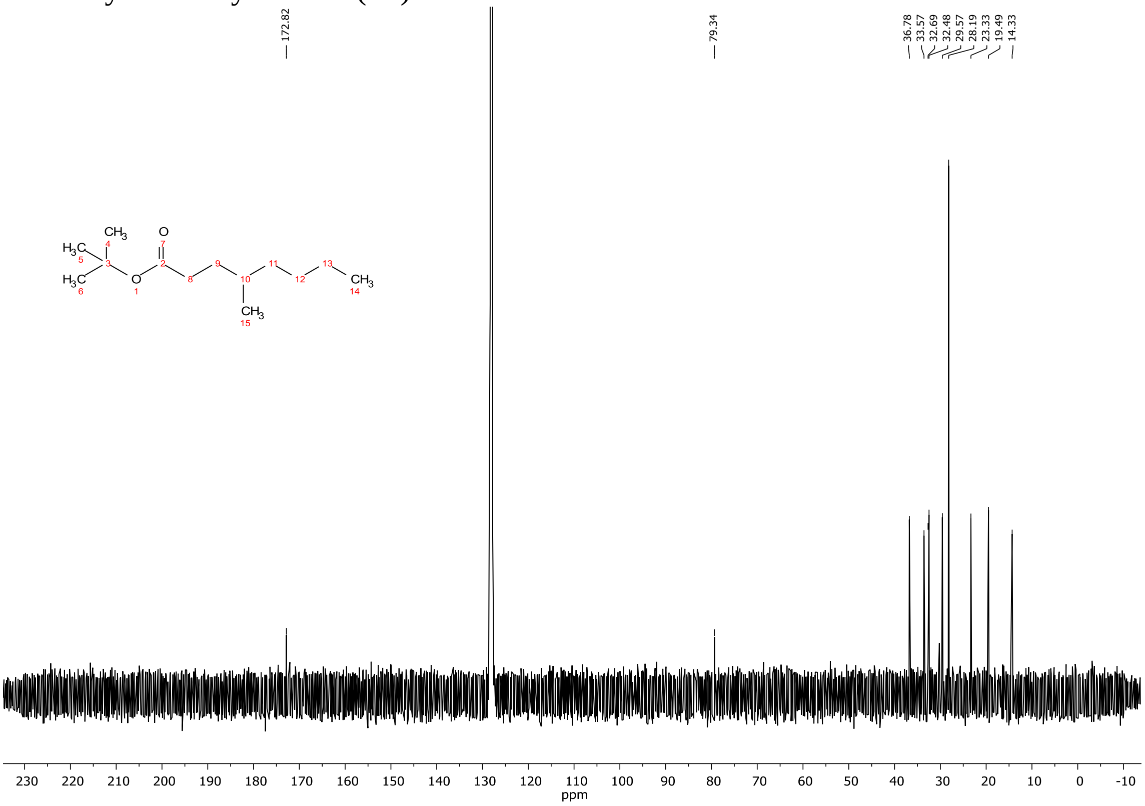

1-(*sec*-Butyl)-3,4-dimethylbenzene (**16a**) ^{1}**H NMR**

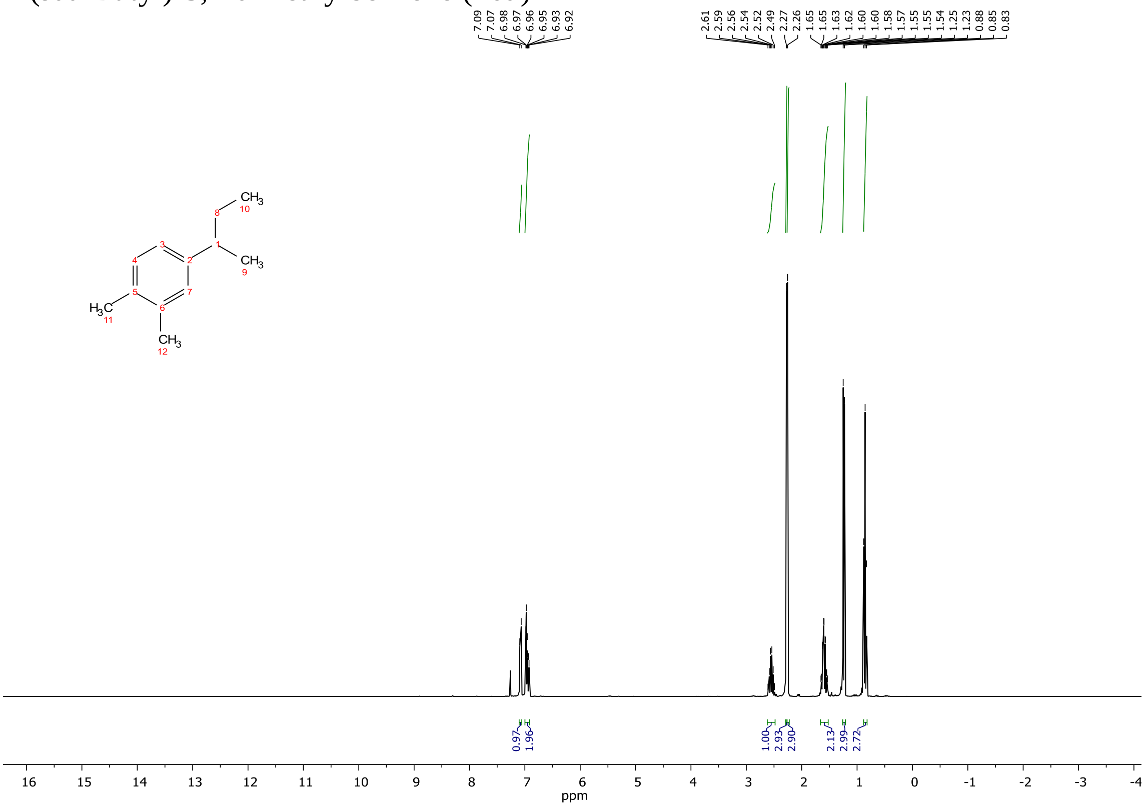

1-(*sec*-Butyl)-3,4-dimethylbenzene (**16a**) **^{13}C NMR**

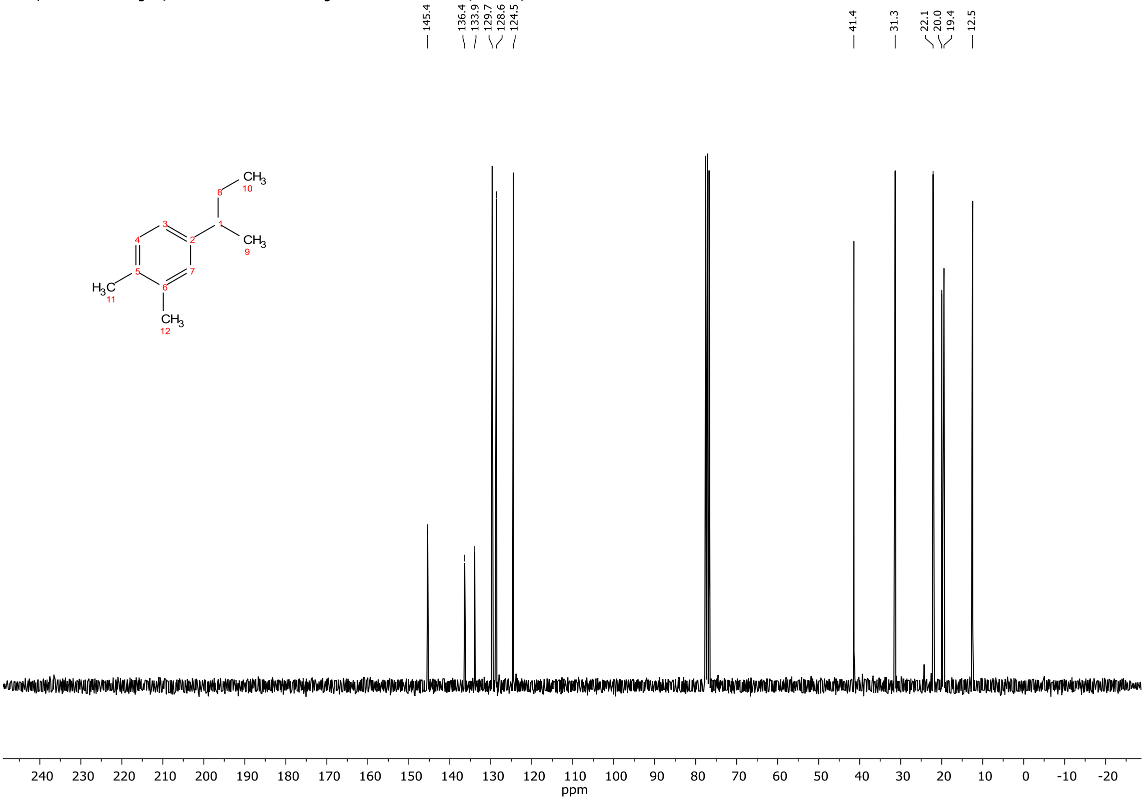

1-(*sec*-Butyl)-2-methoxybenzene (**16b**) **^{1}H NMR**

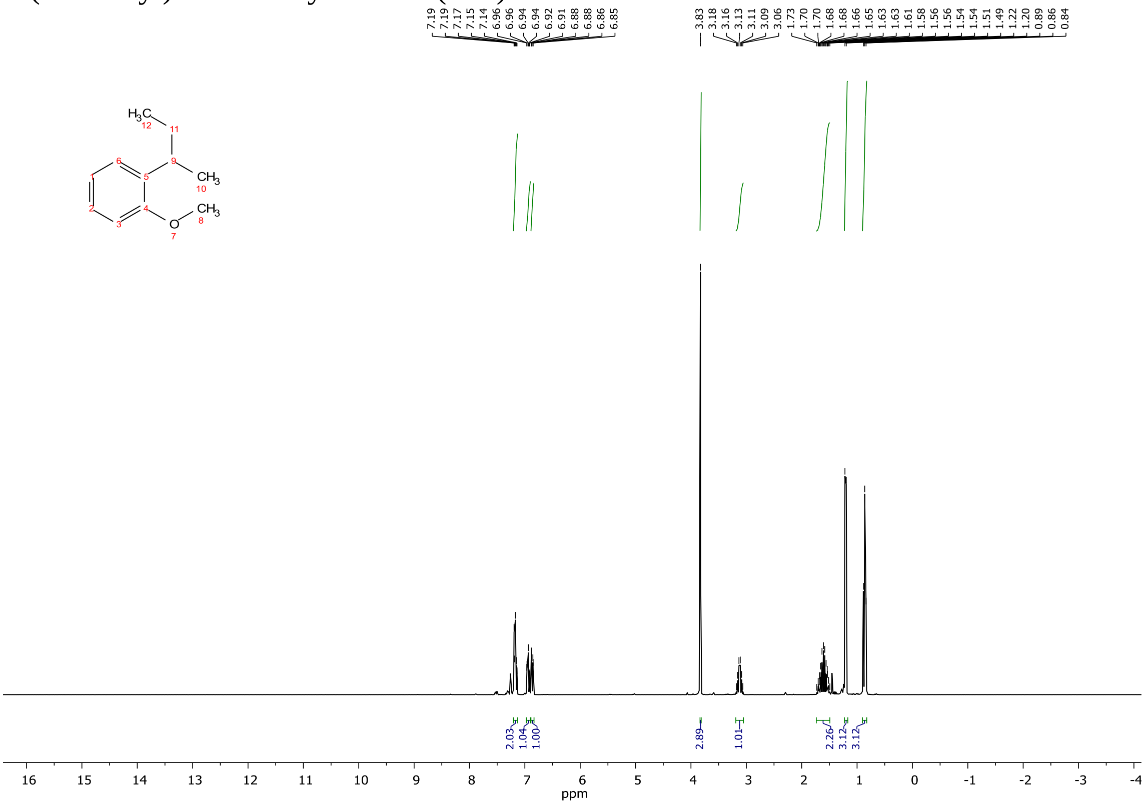

1-(*sec*-Butyl)-2-methoxybenzene (**16b**) ^{1}**H NMR**

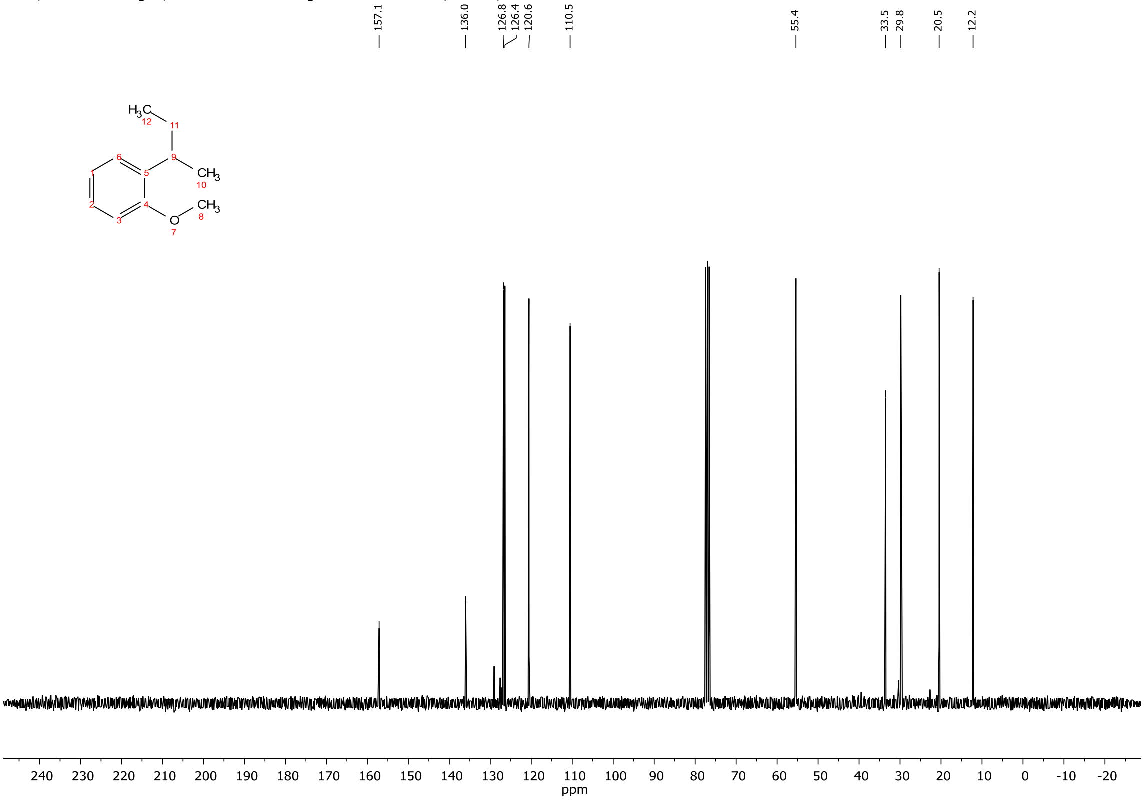

4-(*sec*-Butyl)-1,1'-biphenyl (**16c**) ^{1}**H NMR**

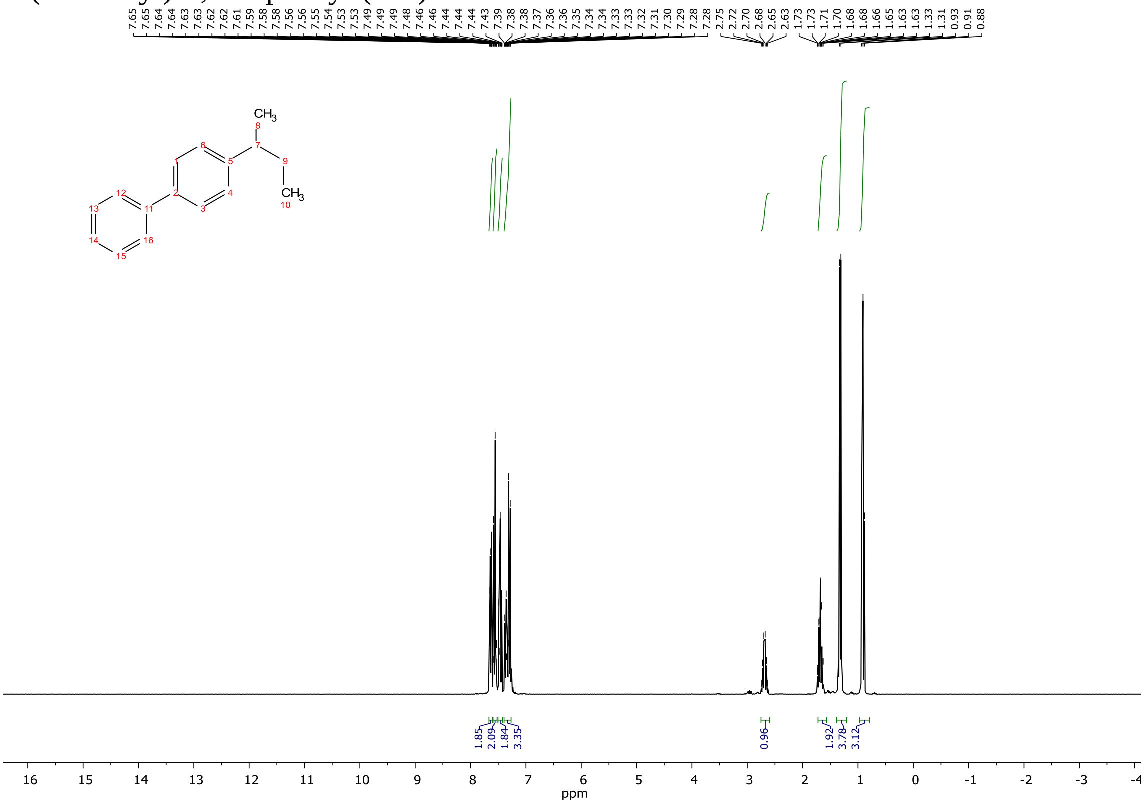

4-(*sec*-Butyl)-1,1'-biphenyl (**16c**) **^{13}C NMR**

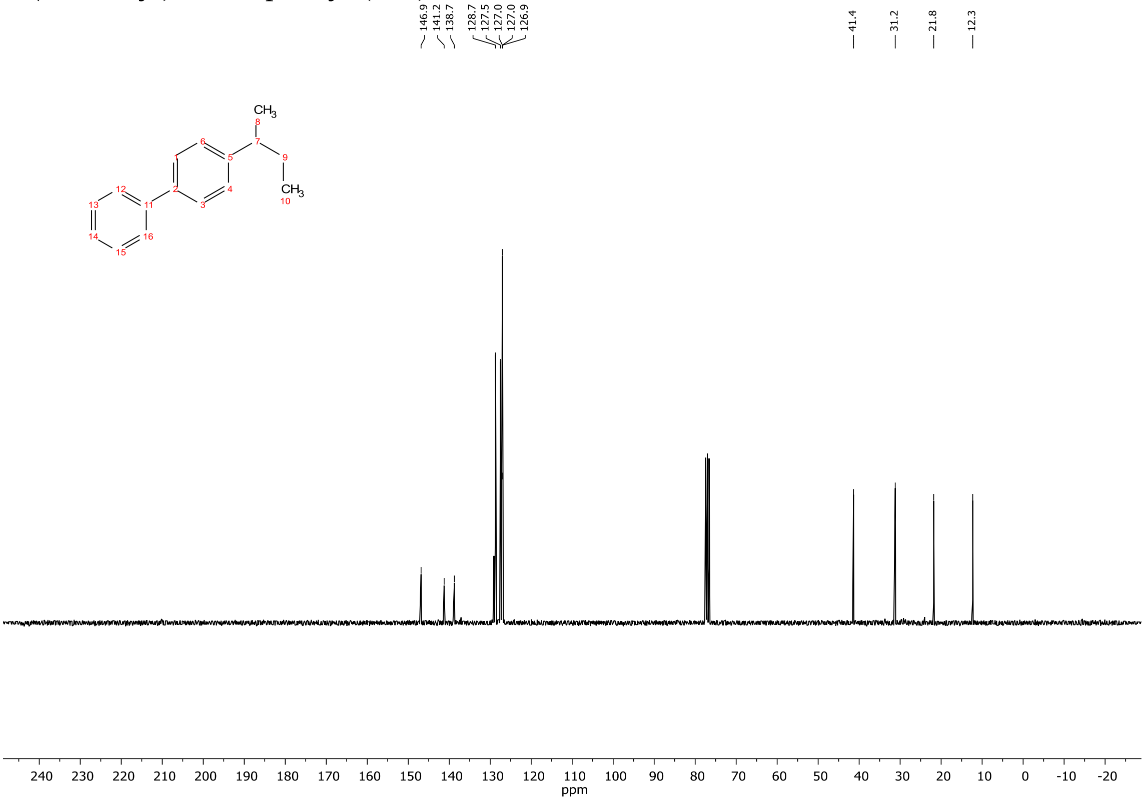

4,4'-(Propane-1,1-diyl)bis(fluorobenzene) (**16d**) **^{1}H NMR**

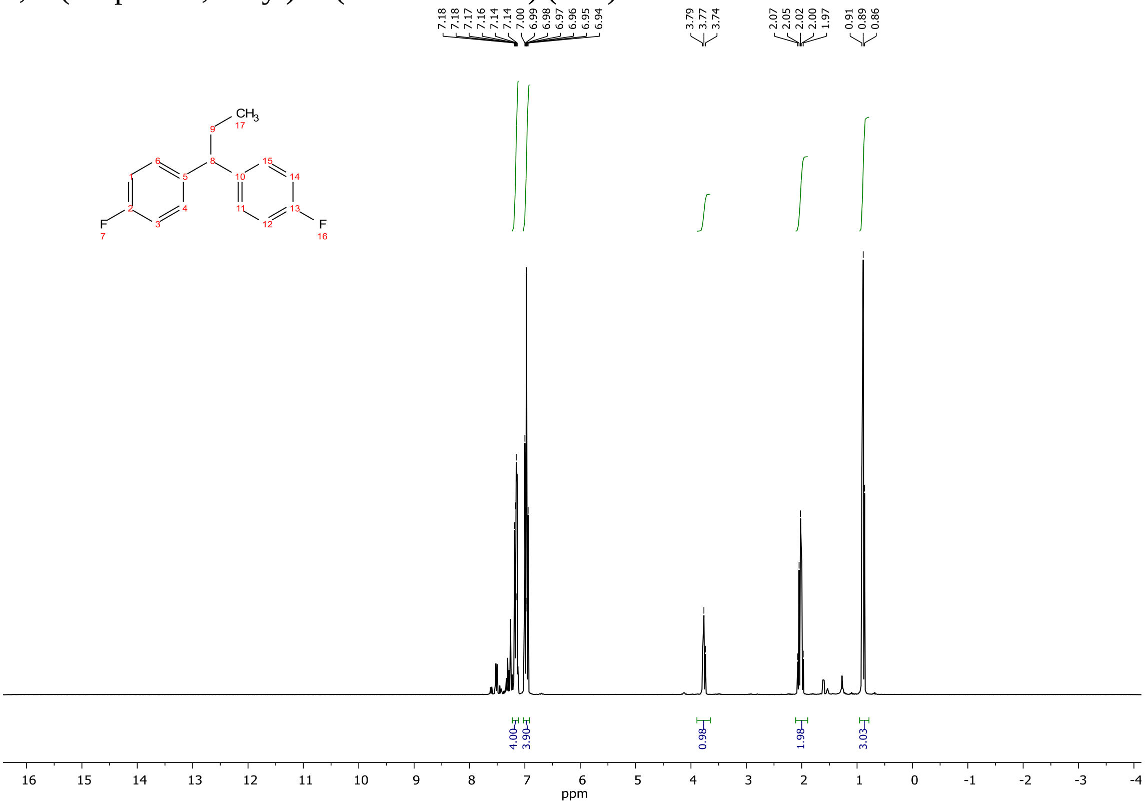

4,4'-(Propane-1,1-diyl)bis(fluorobenzene) (**16d**) ^{13}C NMR

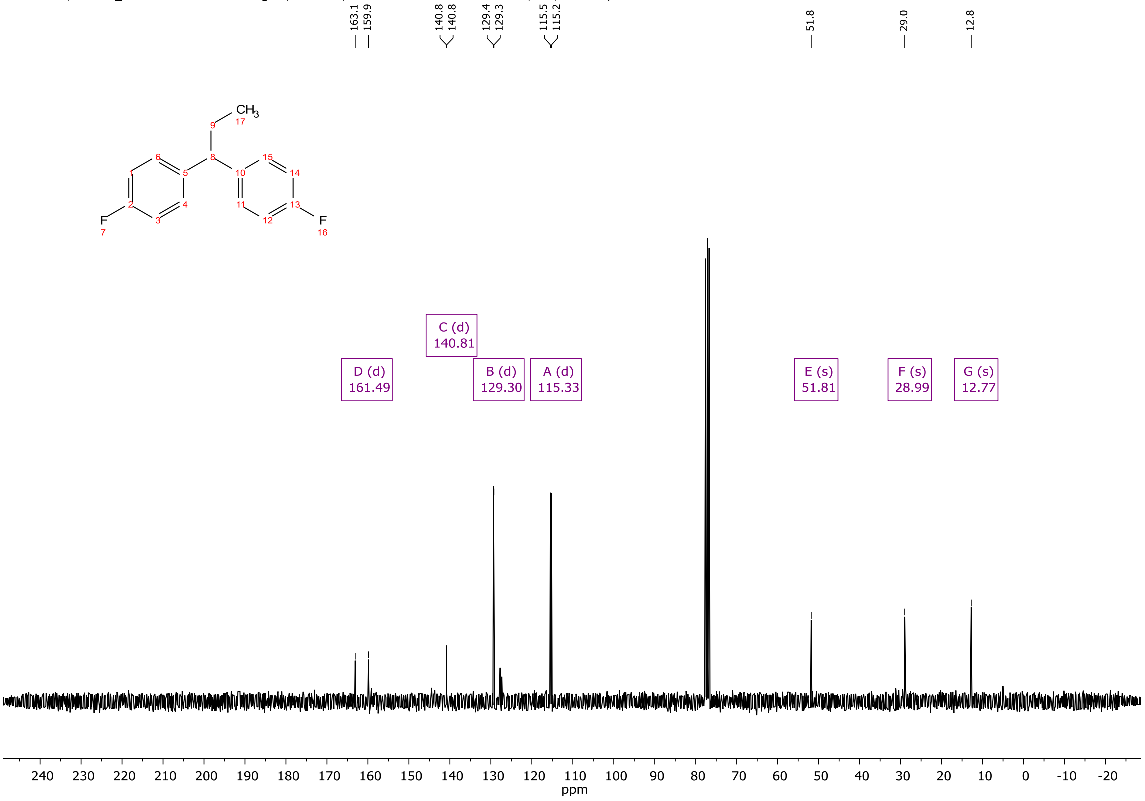

2-(*sec*-Butyl)benzo[b]thiophene (**16e**) ^{1}H NMR

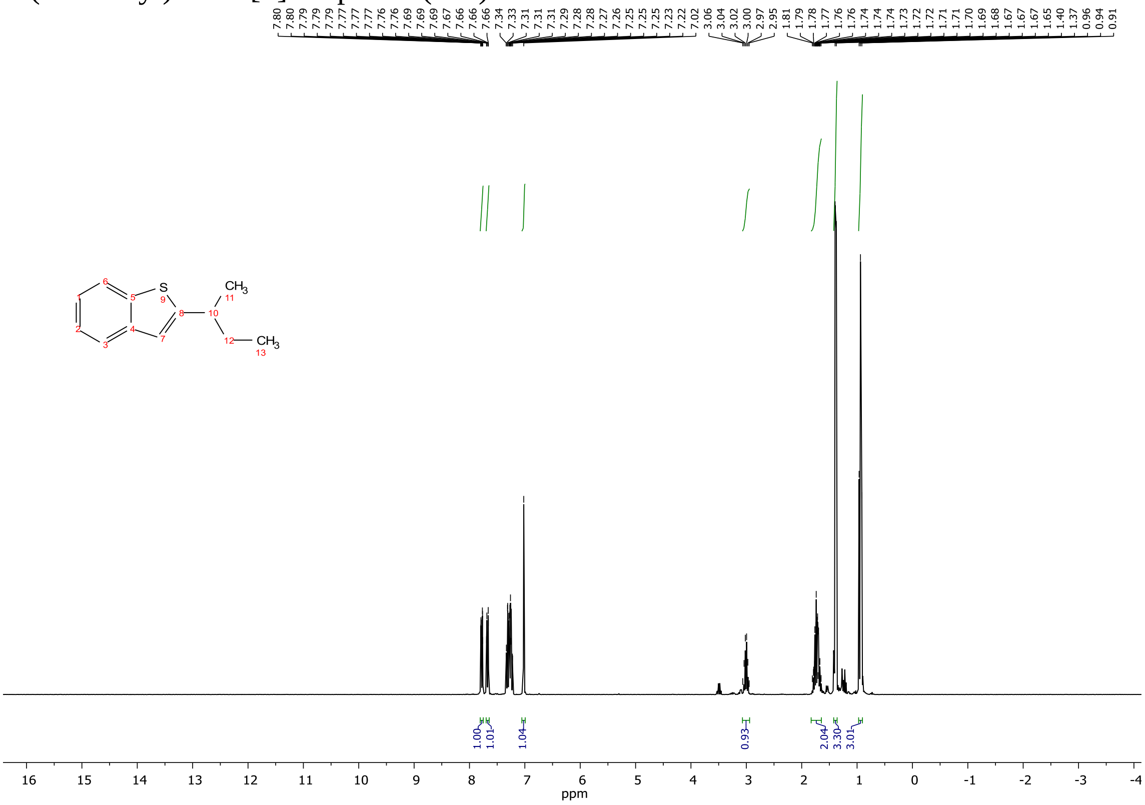

2-(*sec*-Butyl)benzo[b]thiophene (**16e**) **^{13}C NMR**

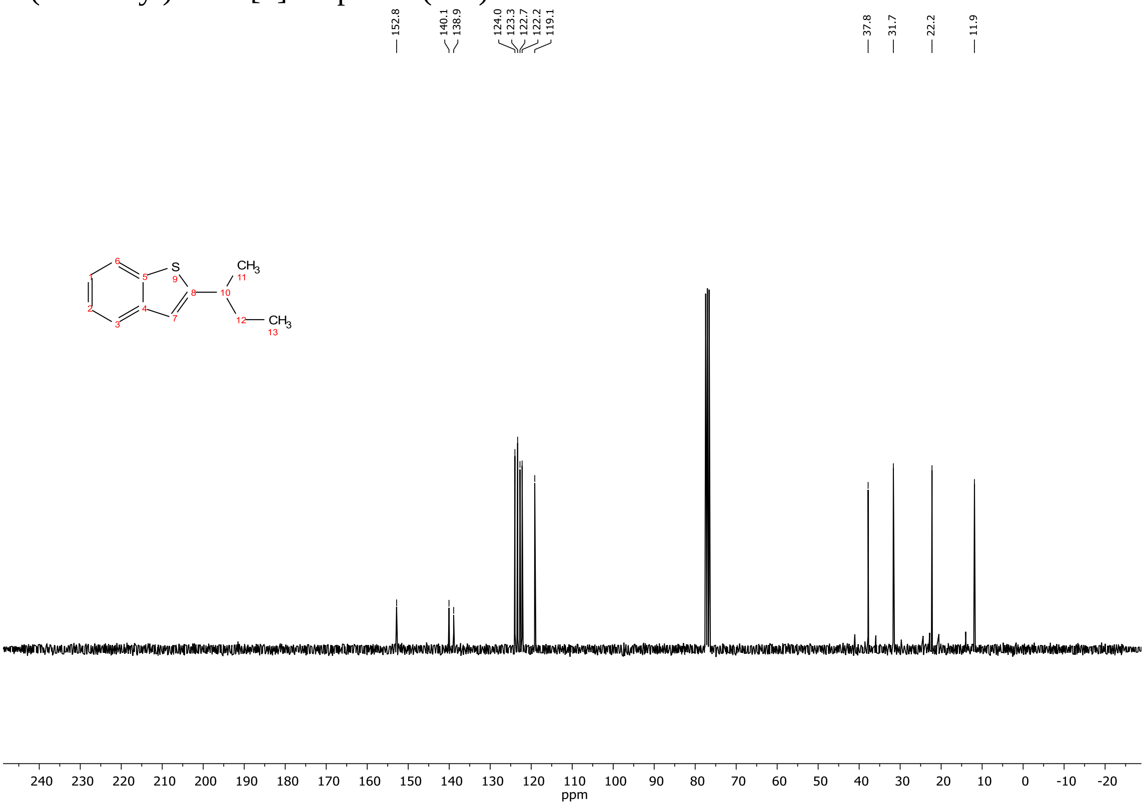

2-(*sec*-Butyl)benzofuran (**16f**) **^{1}H NMR**

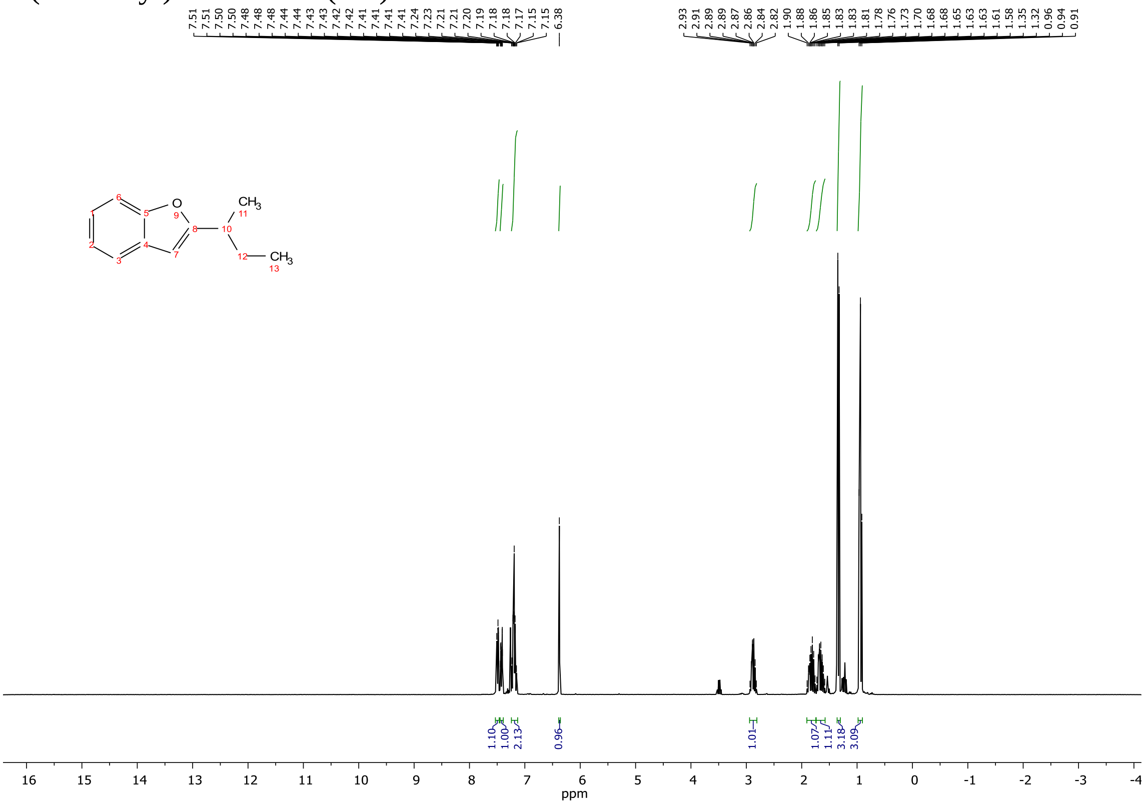

2-(*sec*-Butyl)benzofuran (**16f**) **¹H NMR**

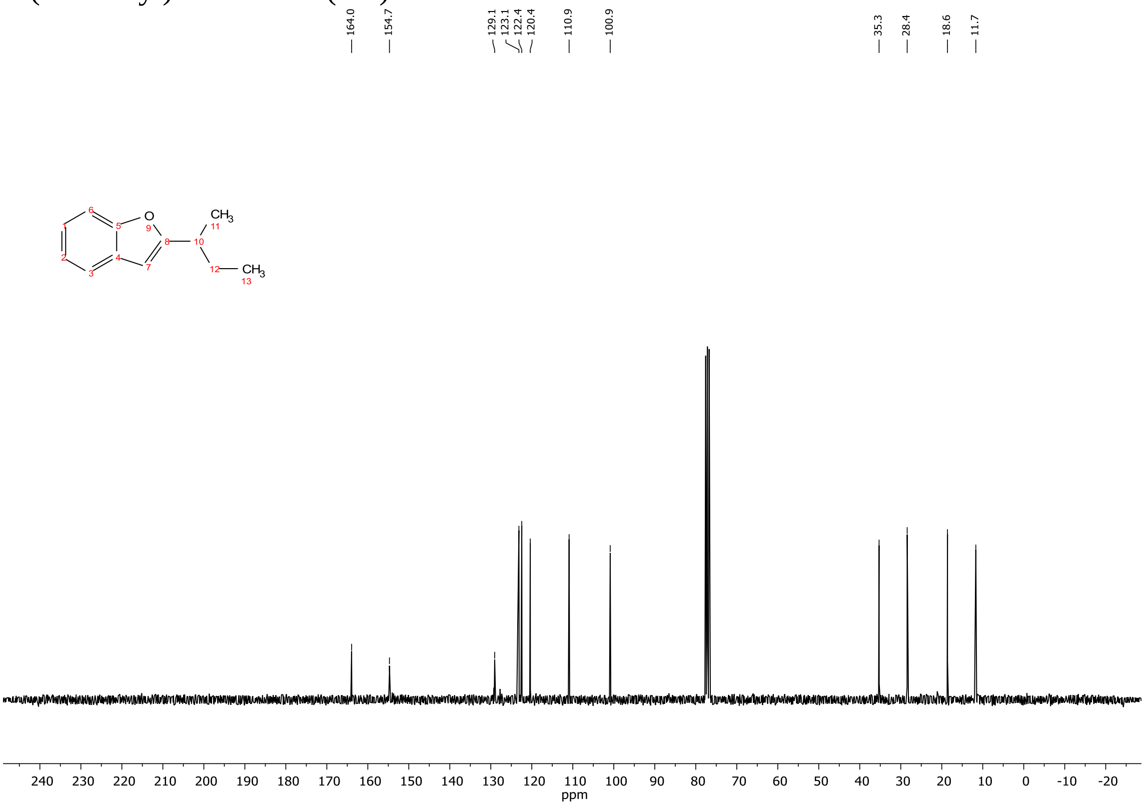

Triphenylbutylsilane (**16g**) **¹H NMR**

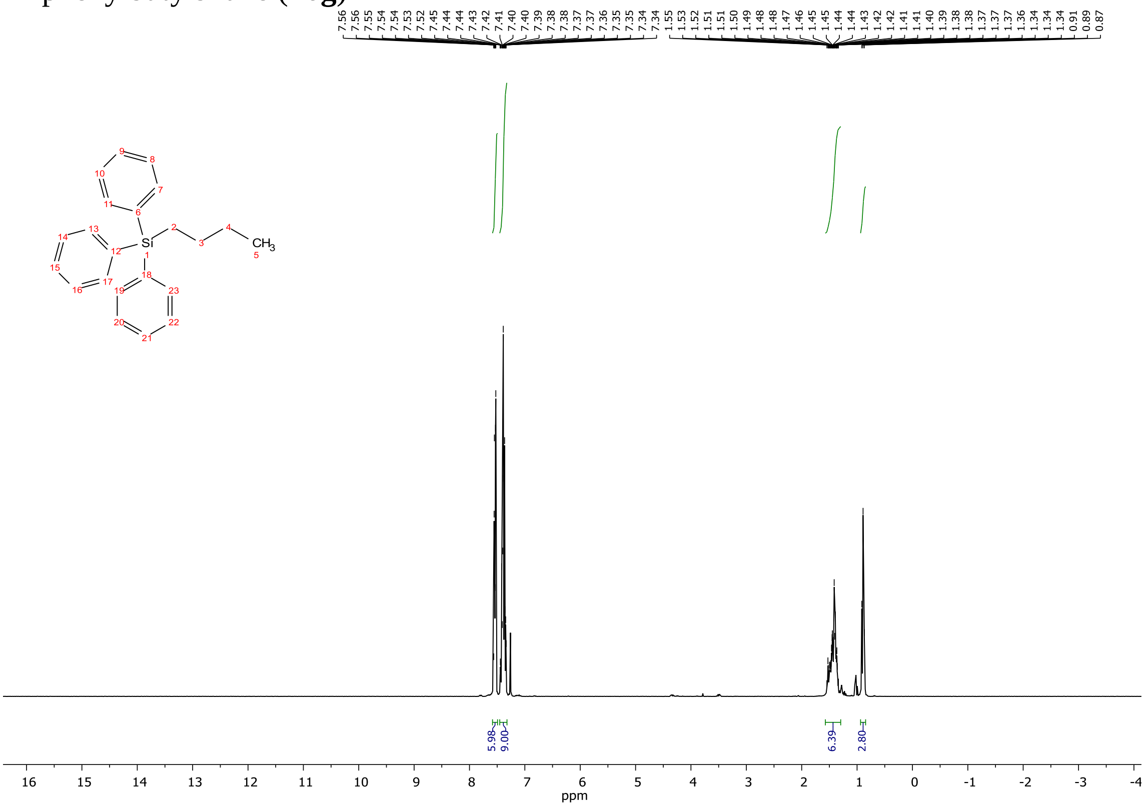

Triphenylbutylsilane (**16g**) ^{13}C NMR

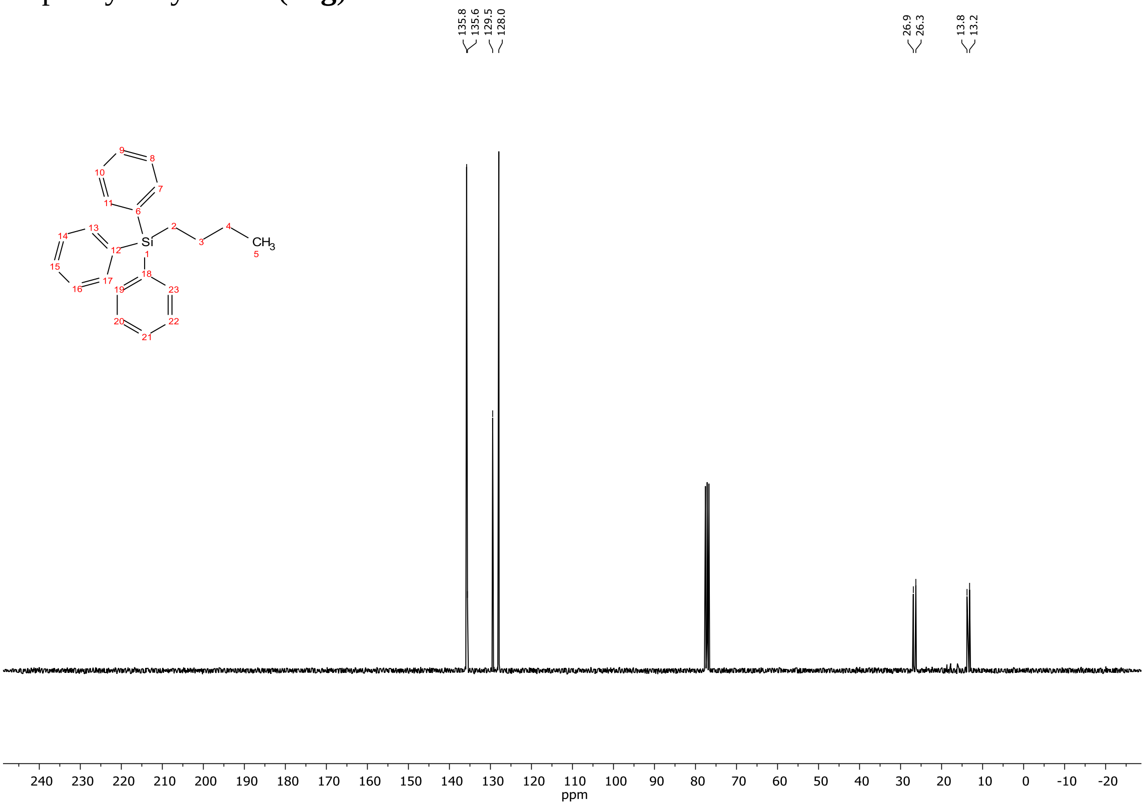

4-(*sec*-Butyl)phenol (**16h**) ^{1}H NMR

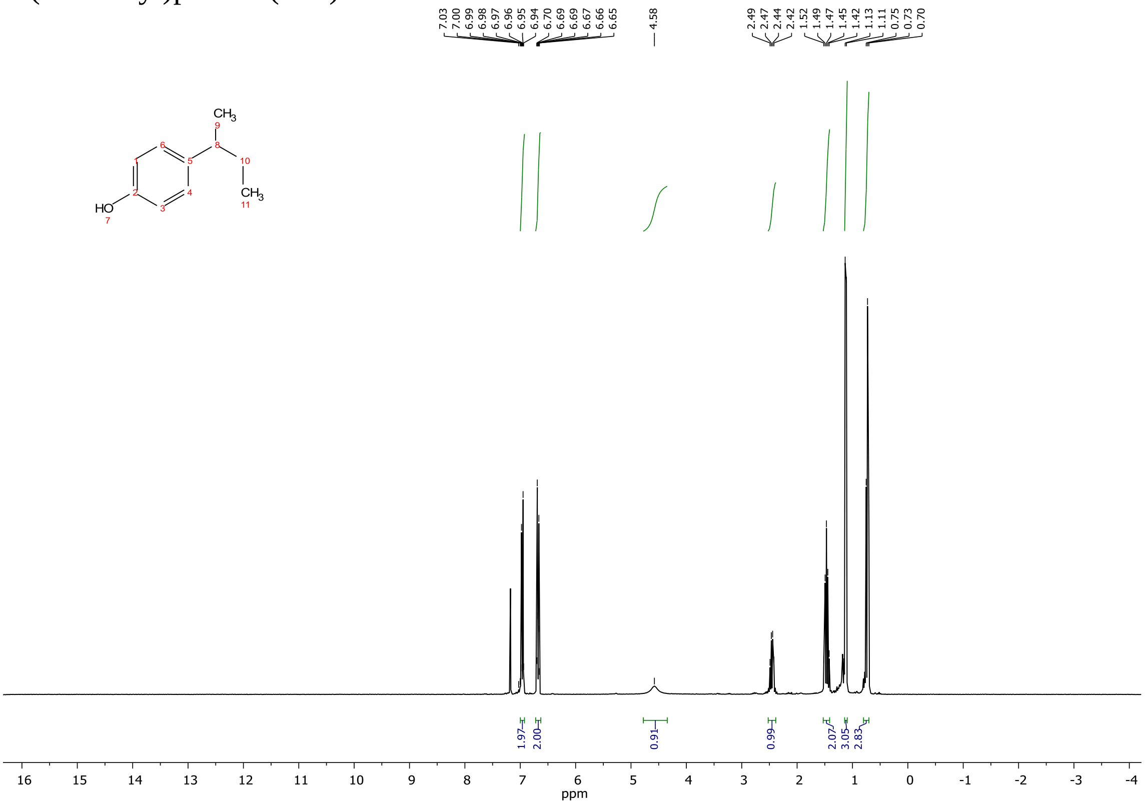

4-(*sec*-Butyl)phenol (**16h**) **^{1}H NMR**

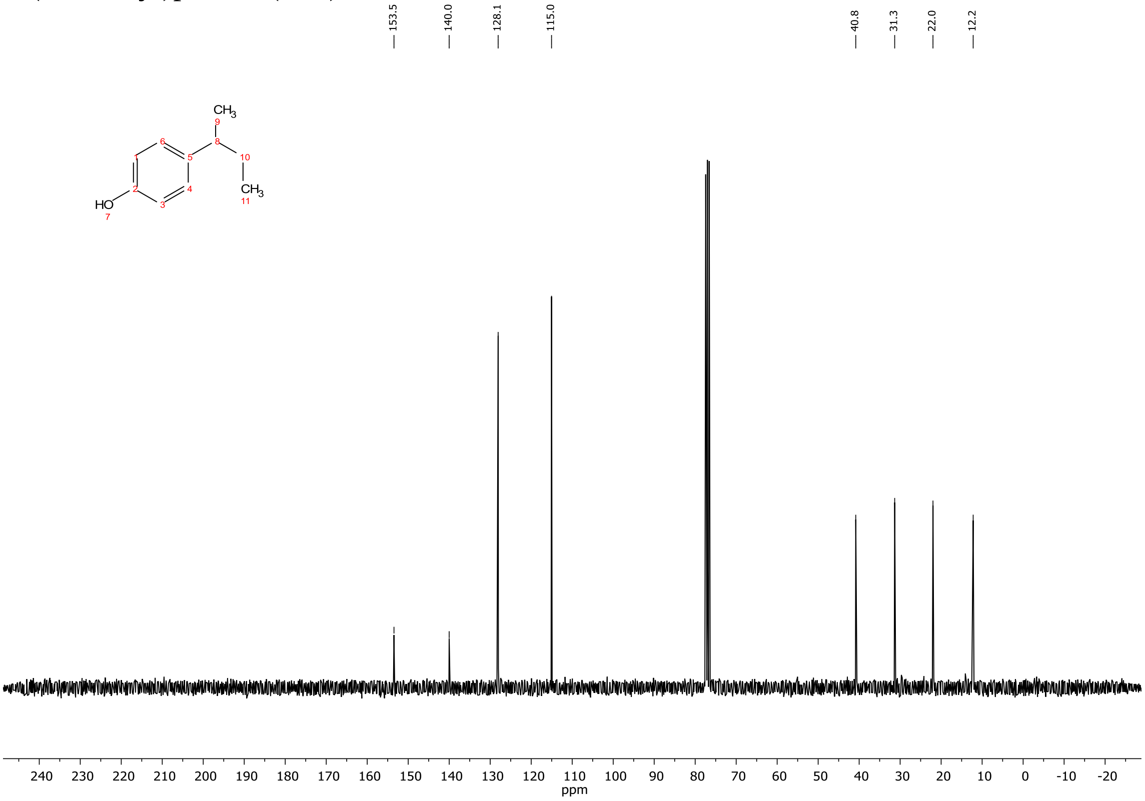

rac-((1*S*,2*S*)-2-Methylcyclohexyl)benzene (**16i**) **^{1}H NMR**

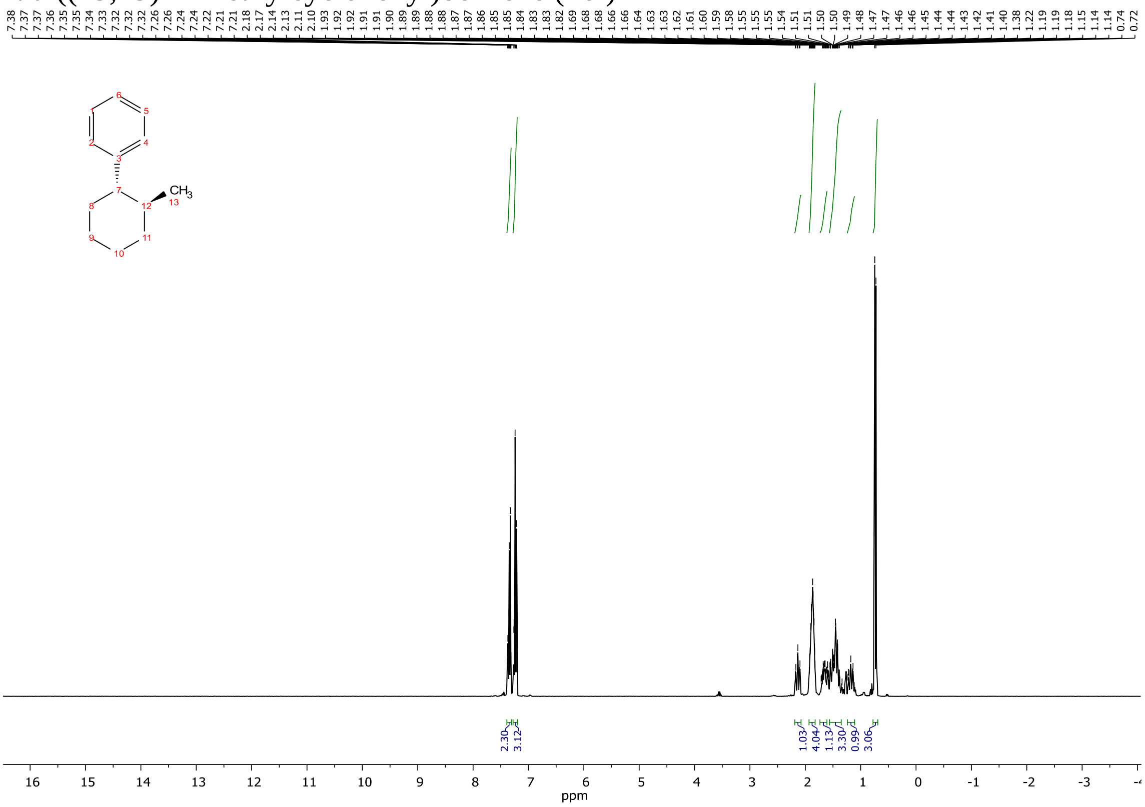

rac-((1*S*,2*S*)-2-Methylcyclohexyl)benzene (**16i**) **^{1}H NMR**

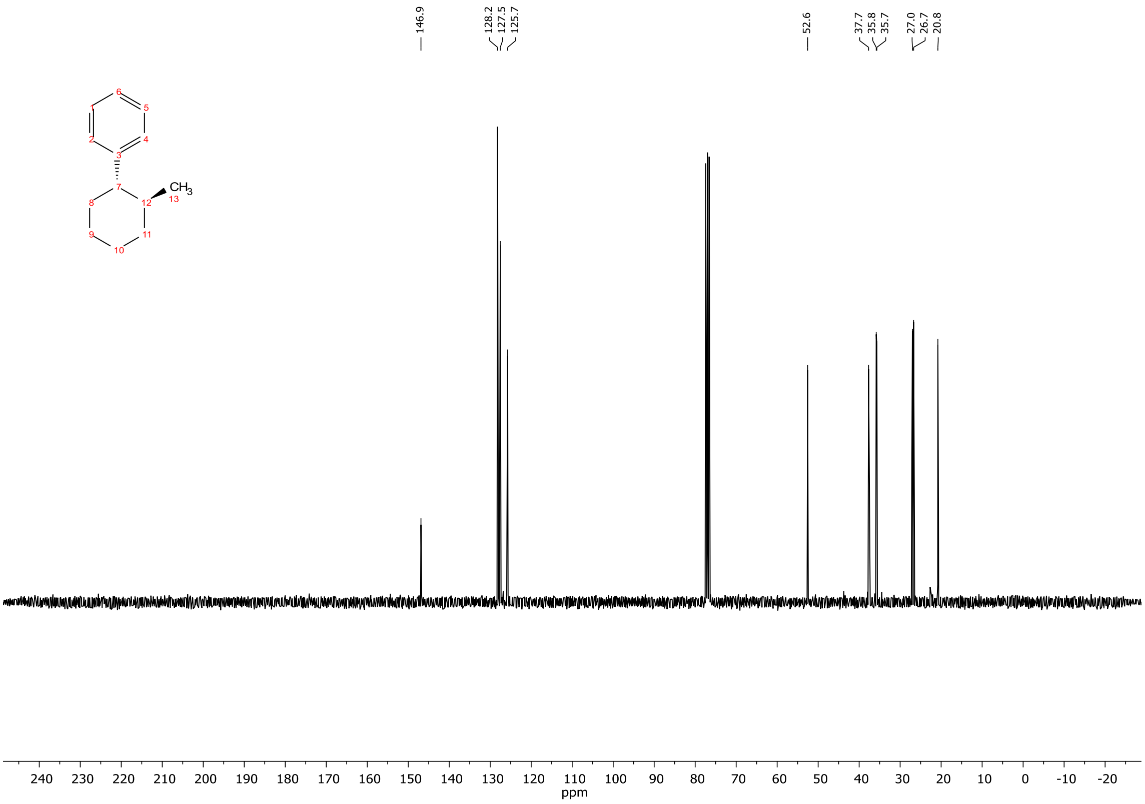

(6a*R*,8*S*,9*S*,10a*R*)-1-Methoxy-6,6,8,9-tetramethyl-3-pentyl-6a,7,8,9,10,10a-hexahydro-6*H*-benzo[c]chromene (**16j**) **^{1}H NMR**

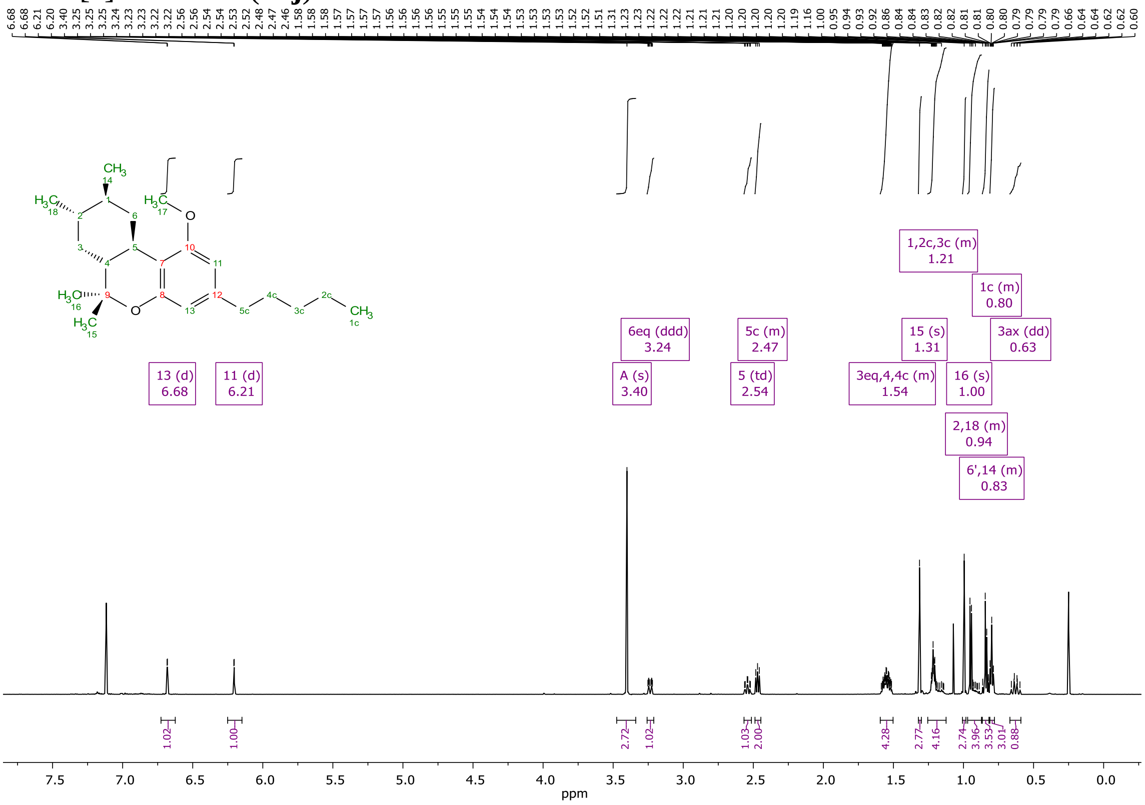

(6a*R*,8*S*,9*S*,10a*R*)-1-Methoxy-6,6,8,9-tetramethyl-3-pentyl-6a,7,8,9,10,10a-hexahydro-6*H*-benzo[c]chromene (**16j**) ^{13}**C NMR**

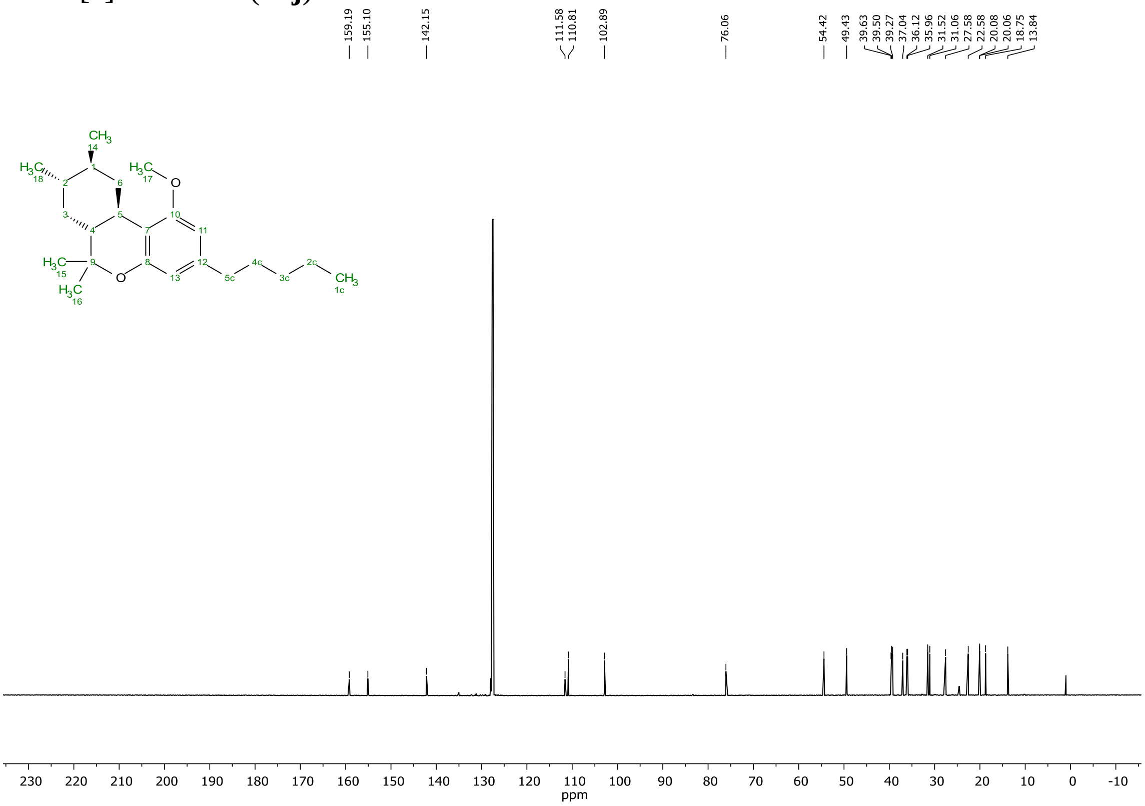

(6a*R*,8*S*,9*S*,10a*R*)-1-Methoxy-6,6,8,9-tetramethyl-3-pentyl-6a,7,8,9,10,10a-hexahydro-6*H*-benzo[c]chromene (**16j**) ^{1}**H NMR**

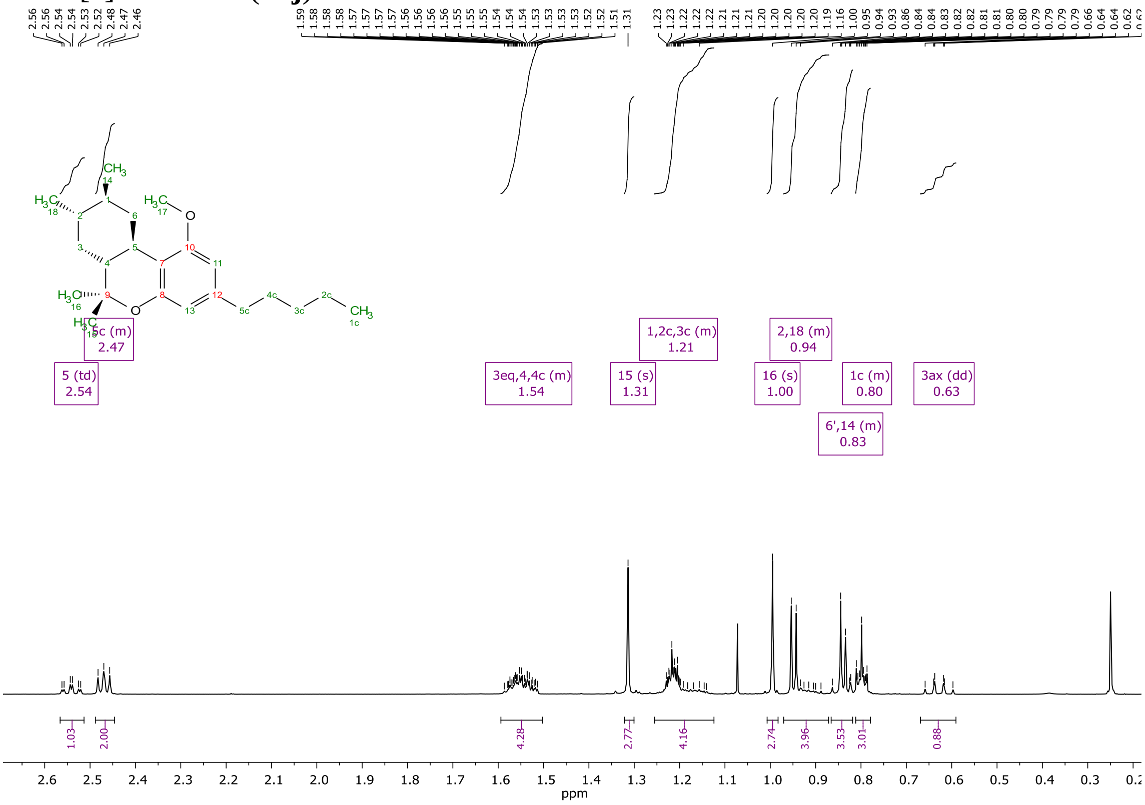

(6a*R*,8*S*,9*S*,10a*R*)-1-Methoxy-6,6,8,9-tetramethyl-3-pentyl-6a,7,8,9,10,10a-hexahydro-6*H*-benzo[c]chromene (**16j**) ^{1}**H NMR**

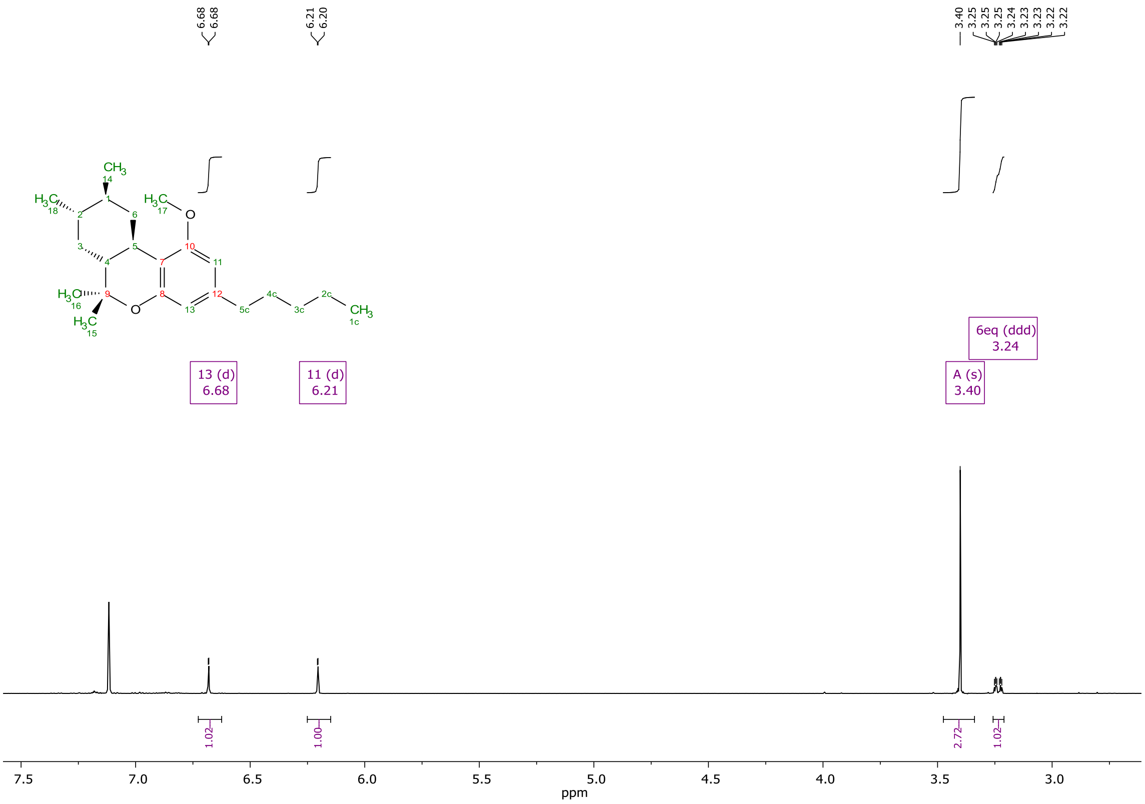

(3*S*,5*R*,6*S*,8*R*,9*S*,10*S*,13*R*,14*S*,17*R*)-3-Methoxy-6,10,13,14-tetramethyl-17-((*R*)-6-methylheptan-2-yl)hexadecahydro-1*H*-cyclopenta[a]phenanthrene (**16k**) **^{1}H NMR**

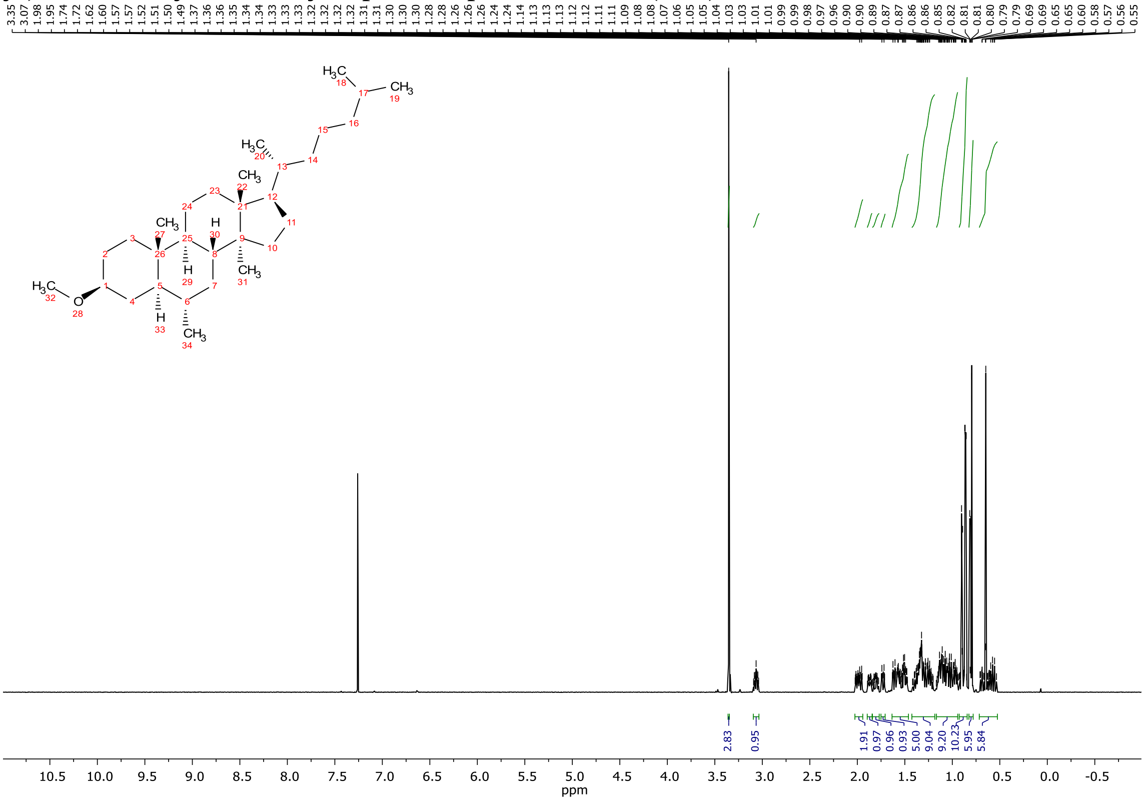

(3*S*,5*R*,6*S*,8*R*,9*S*,10*S*,13*R*,14*S*,17*R*)-3-Methoxy-6,10,13,14-tetramethyl-17-((*R*)-6-methylheptan-2-yl)hexadecahydro-1*H*-cyclopenta[a]phenanthrene (**16k**) ^{13}**C NMR**

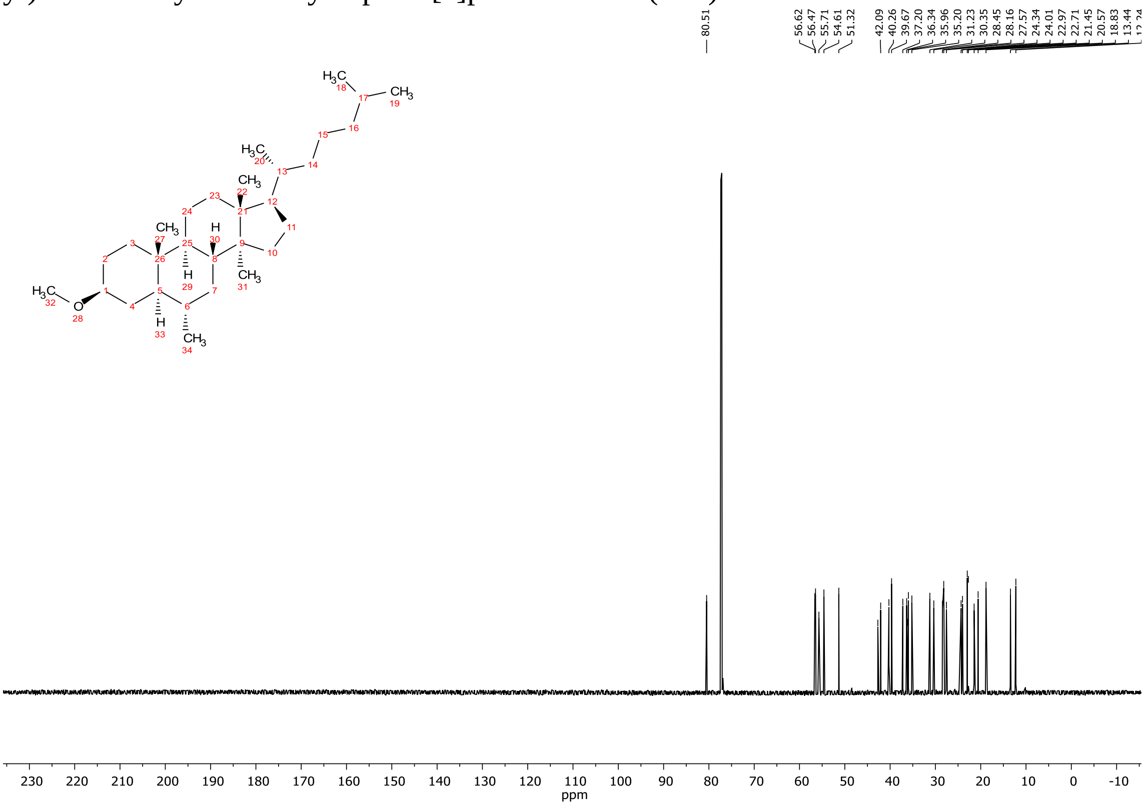

3.2.4 Mechanistic Studies

Hex-3-en-1-ylbenzene (**2o**) ^{1}**H NMR**

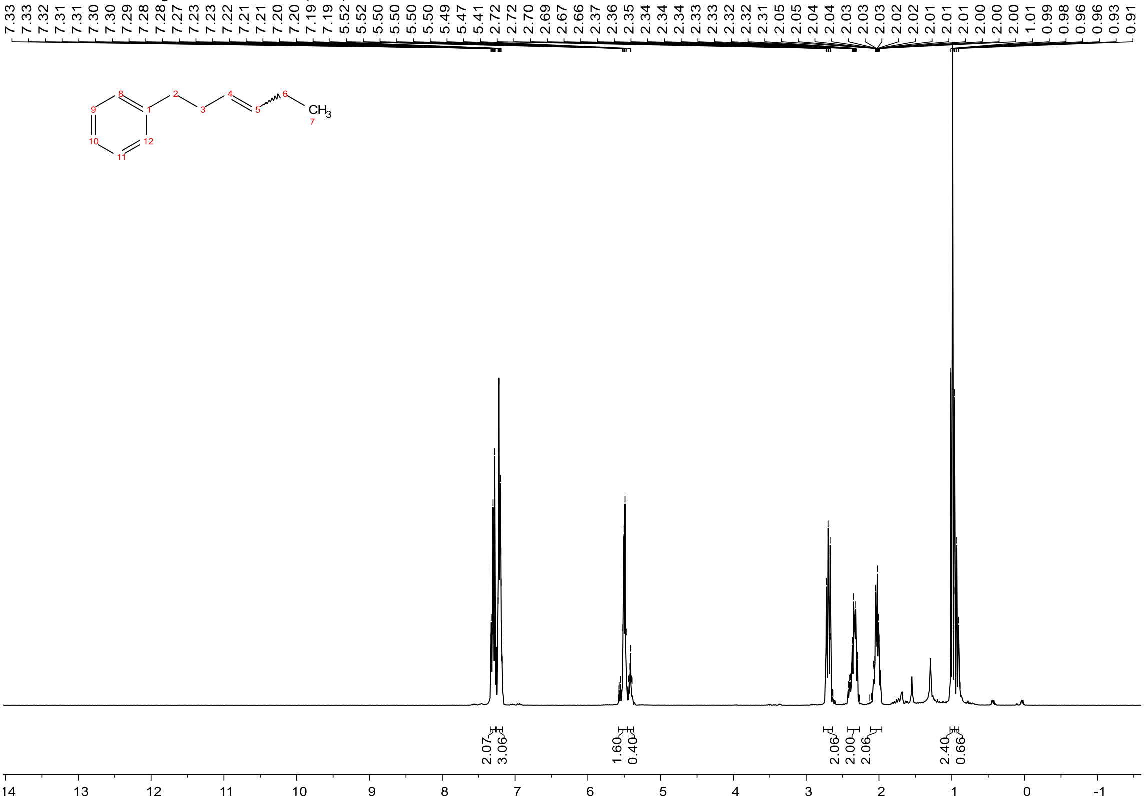

Hex-3-en-1-ylbenzene (**2o**) **^{13}C NMR**

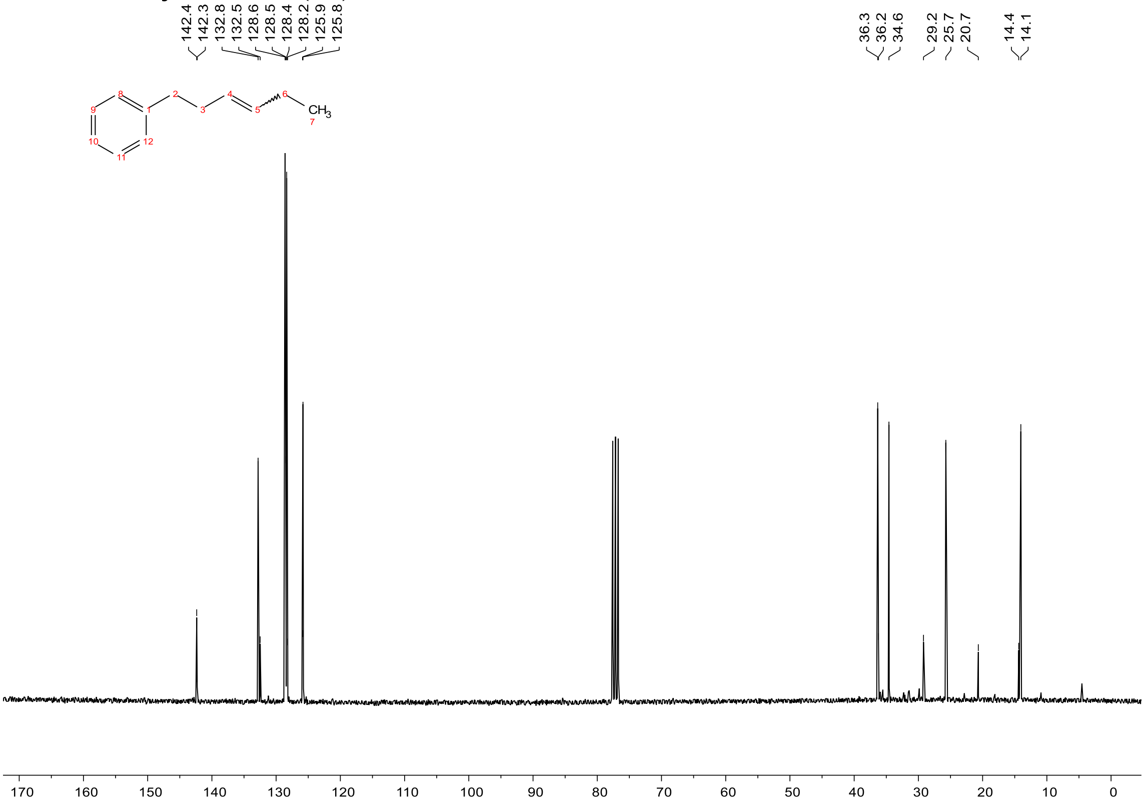

3.2.5 Synthesis of the indolizidine alkaloides

tert-Butyl (S)-2-((*R*/S)-4-(tert-butoxy)-4-oxo-1-(4,4,5,5-tetramethyl-1,3,2-dioxaborolan-2-yl)butyl)pyrrolidine-1-carboxylate (**6**) **^{1}H NMR**

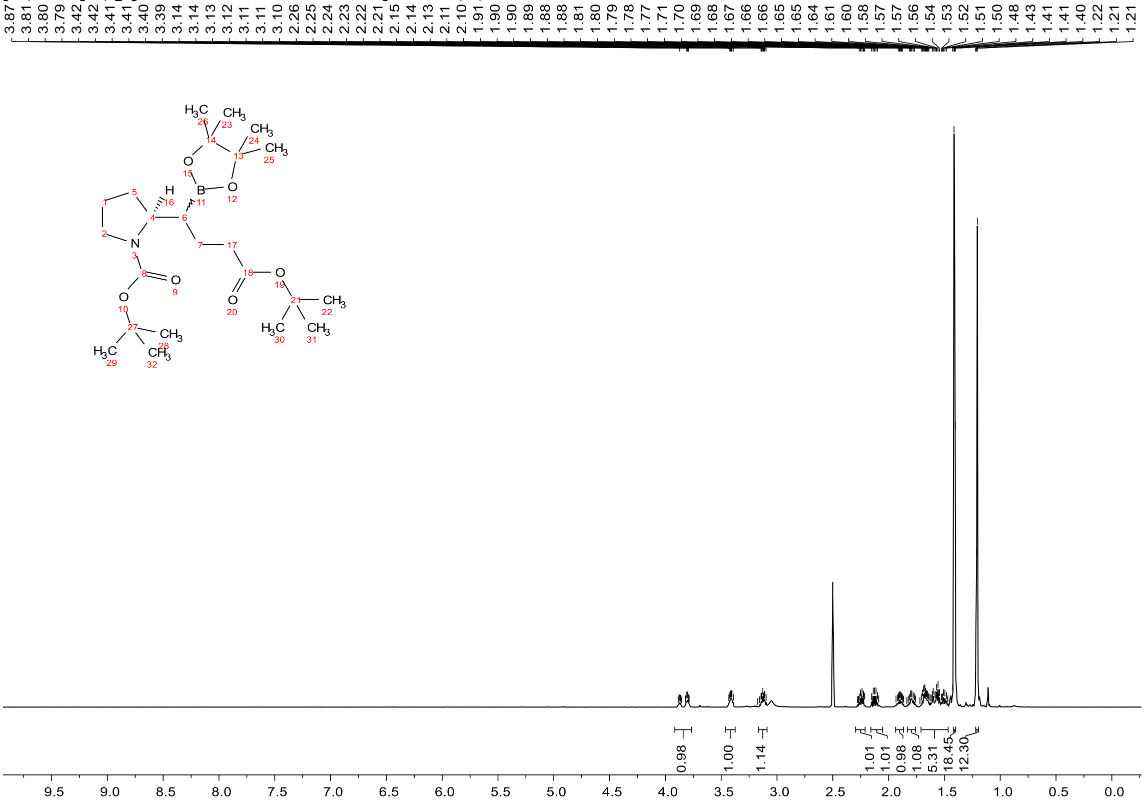

tert-Butyl (S)-2-((*R*/S)-4-(tert-butoxy)-4-oxo-1-(4,4,5,5-tetramethyl-1,3,2-dioxaborolan-2-yl)butyl)pyrrolidine-1-carboxylate (**6**) **^{13}C NMR**

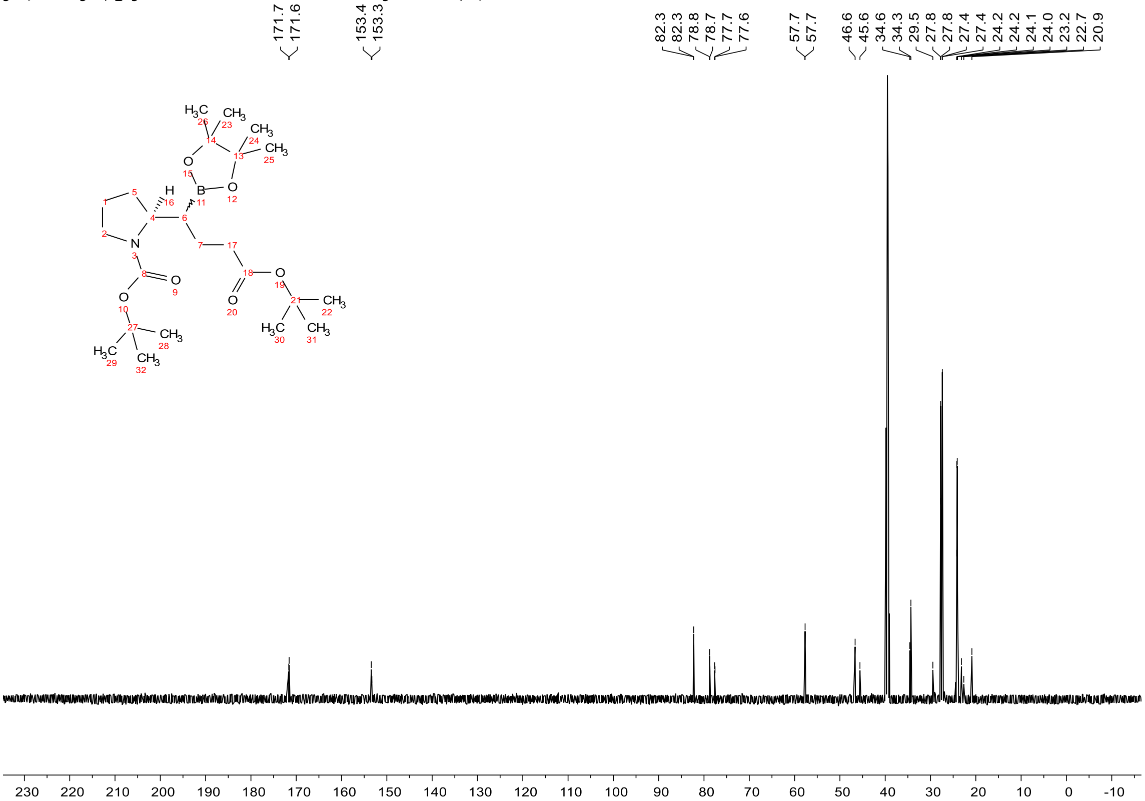

tert-Butyl (*S*)-2-((*R*/*S*)-4-(tert-butoxy)-4-oxo-1-(4,4,5,5-tetramethyl-1,3,2-dioxaborolan-2-yl)butyl)pyrrolidine-1-carboxylate (**6**) **^{11}B NMR**

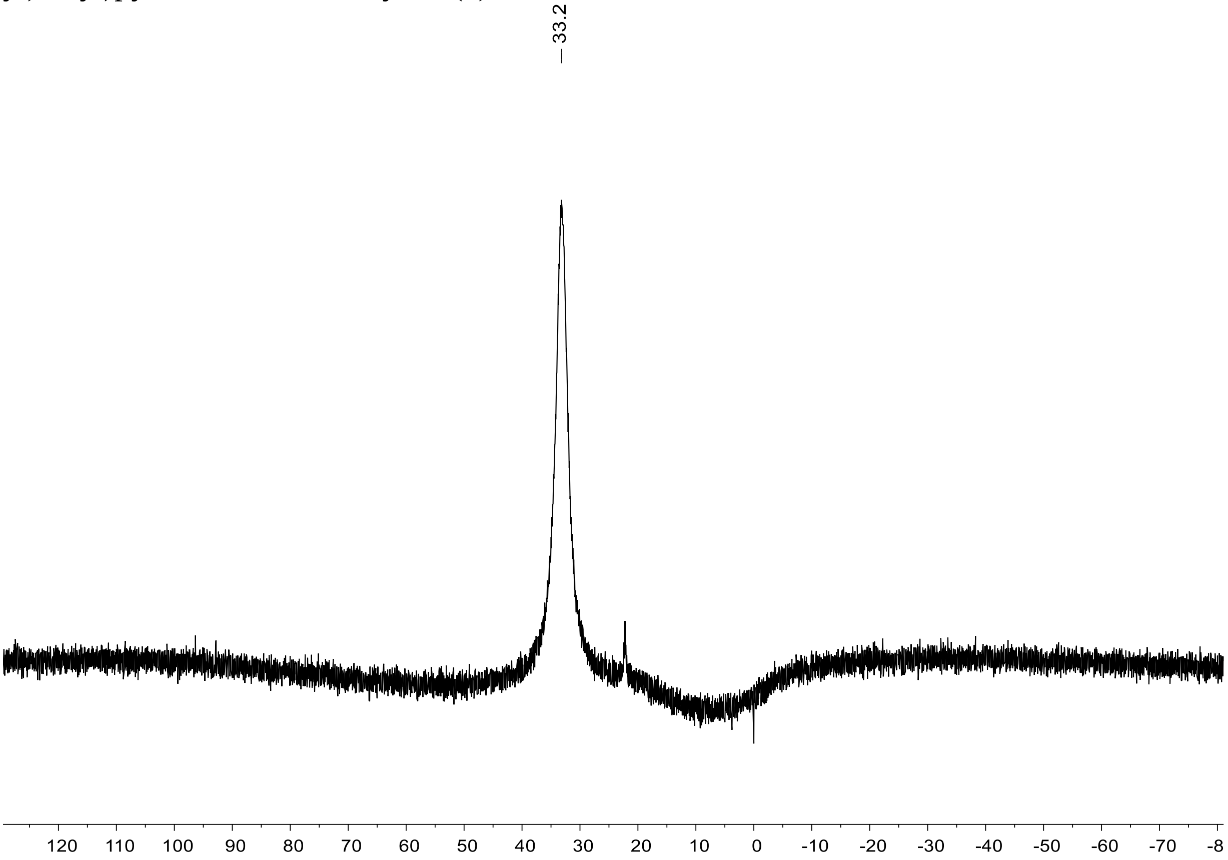

tert-Butyl (*S*)-2-((*S/R*)-5-ethoxy-5-oxo-2-(4,4,5,5-tetramethyl-1,3,2-dioxaborolan-2-yl)pentan-2-yl)pyrrolidine-1-carboxylate (**10**) **¹H NMR**

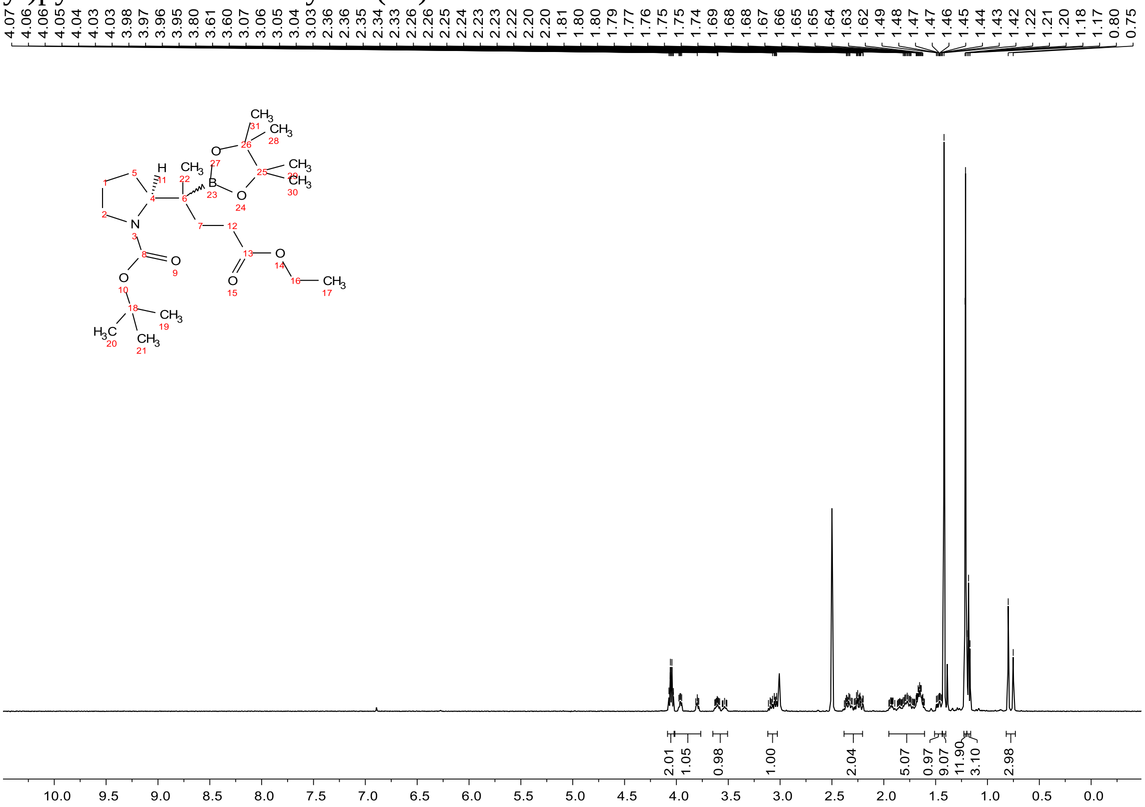

tert-Butyl (*S*)-2-((*S/R*)-5-ethoxy-5-oxo-2-(4,4,5,5-tetramethyl-1,3,2-dioxaborolan-2-yl)pentan-2-yl)pyrrolidine-1-carboxylate (**10**) **¹³C NMR**

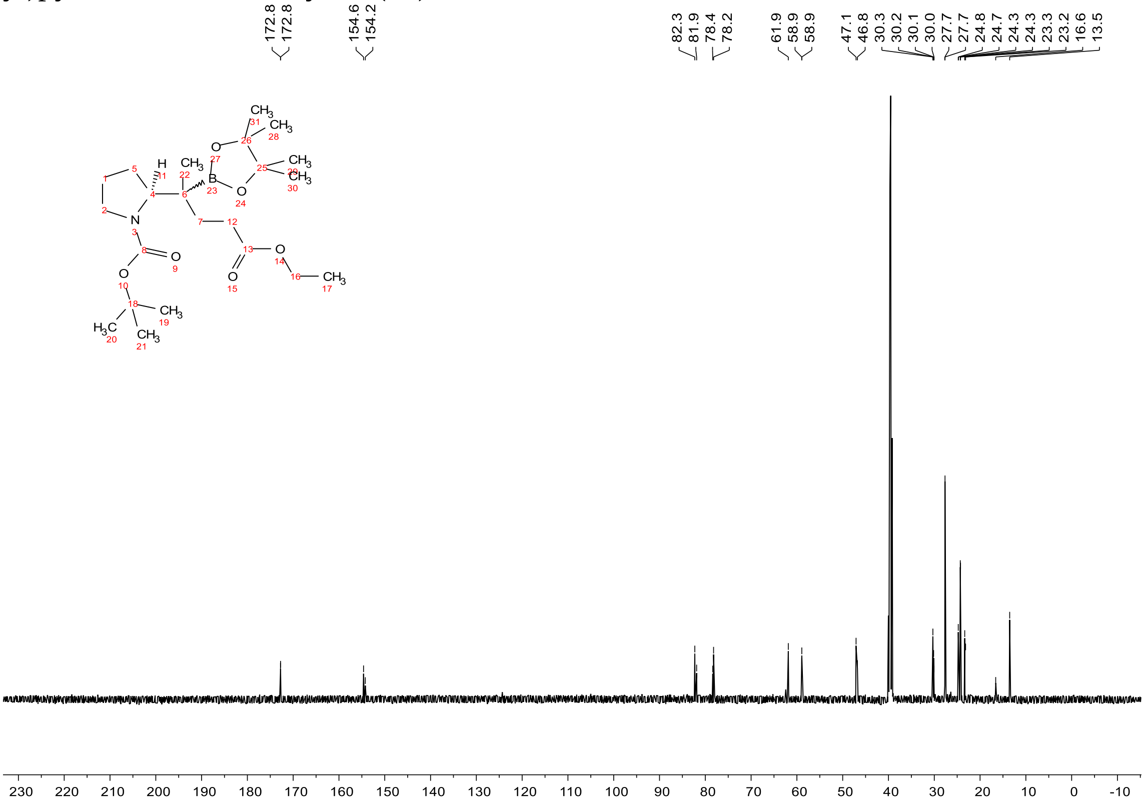

tert-Butyl (*S*)-2-((*S/R*)-5-ethoxy-5-oxo-2-(4,4,5,5-tetramethyl-1,3,2-dioxaborolan-2-yl)pentan-2-yl)pyrrolidine-1-carboxylate (**10**) **^{11}B NMR**

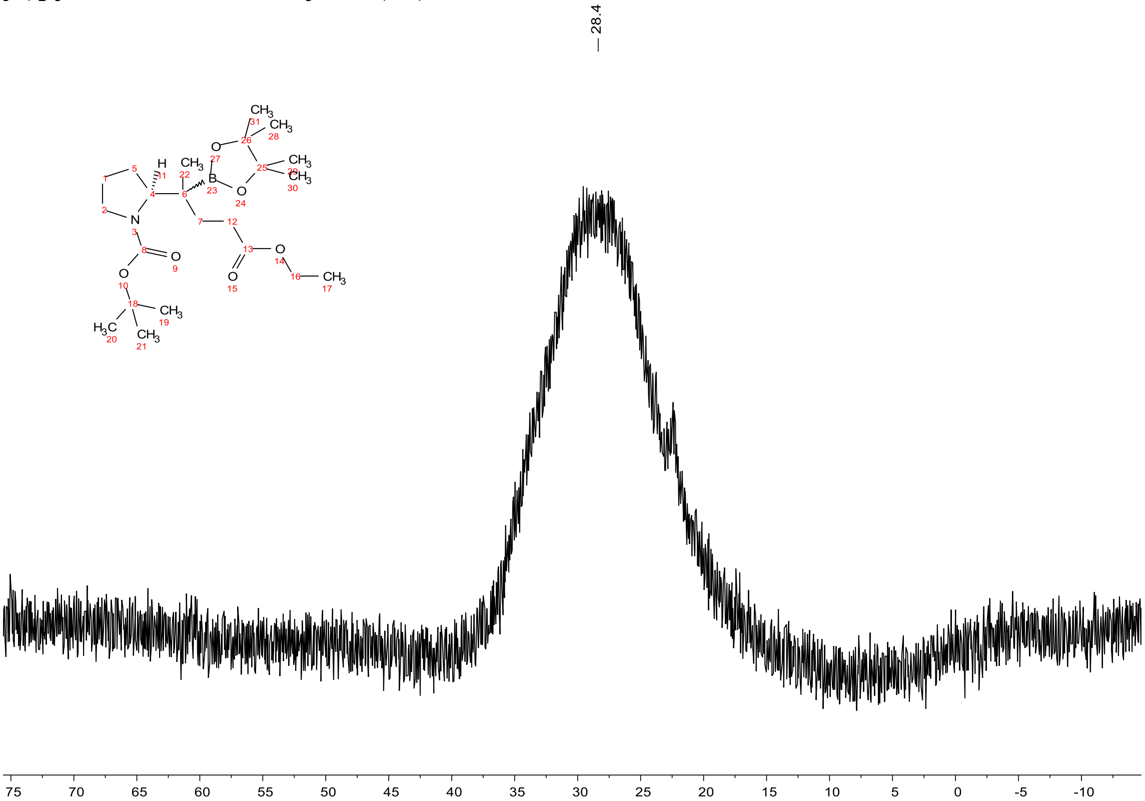

tert-Butyl (*R*)-2-(4-(*tert*-butoxy)-4-oxobutyl)pyrrolidine-1-carboxylate (**7**) **^{1}H NMR**

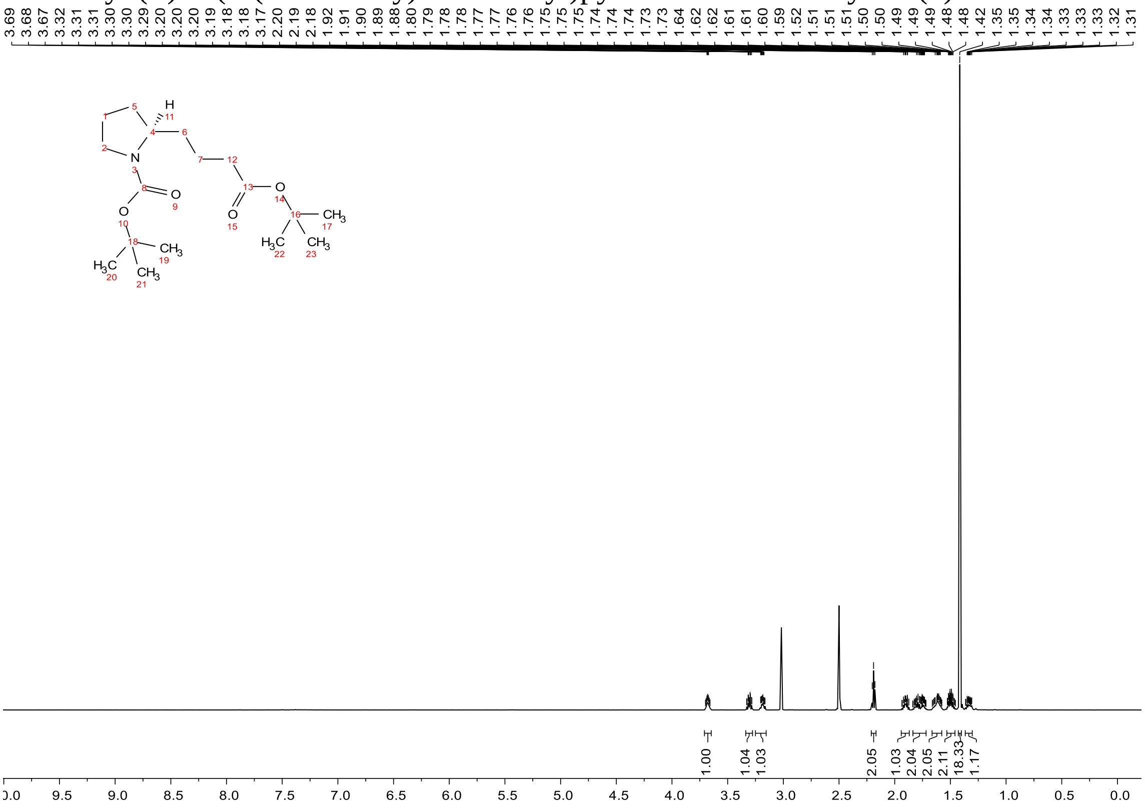

tert-Butyl (*R*)-2-(4-(*tert*-butoxy)-4-oxobutyl)pyrrolidine-1-carboxylate (**7**) ^{13}**C NMR**

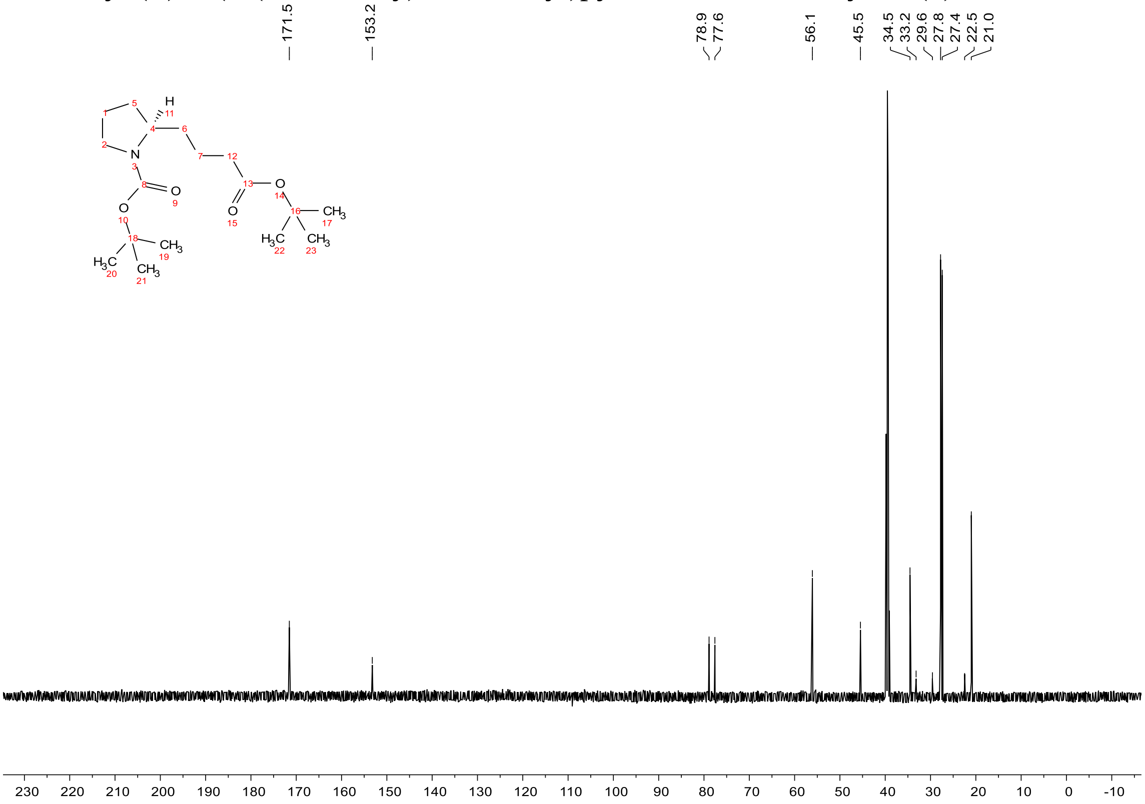

(*R*)-Hexahydroindolizidine-5(1*H*)-one (**8**) ^{1}**H NMR**

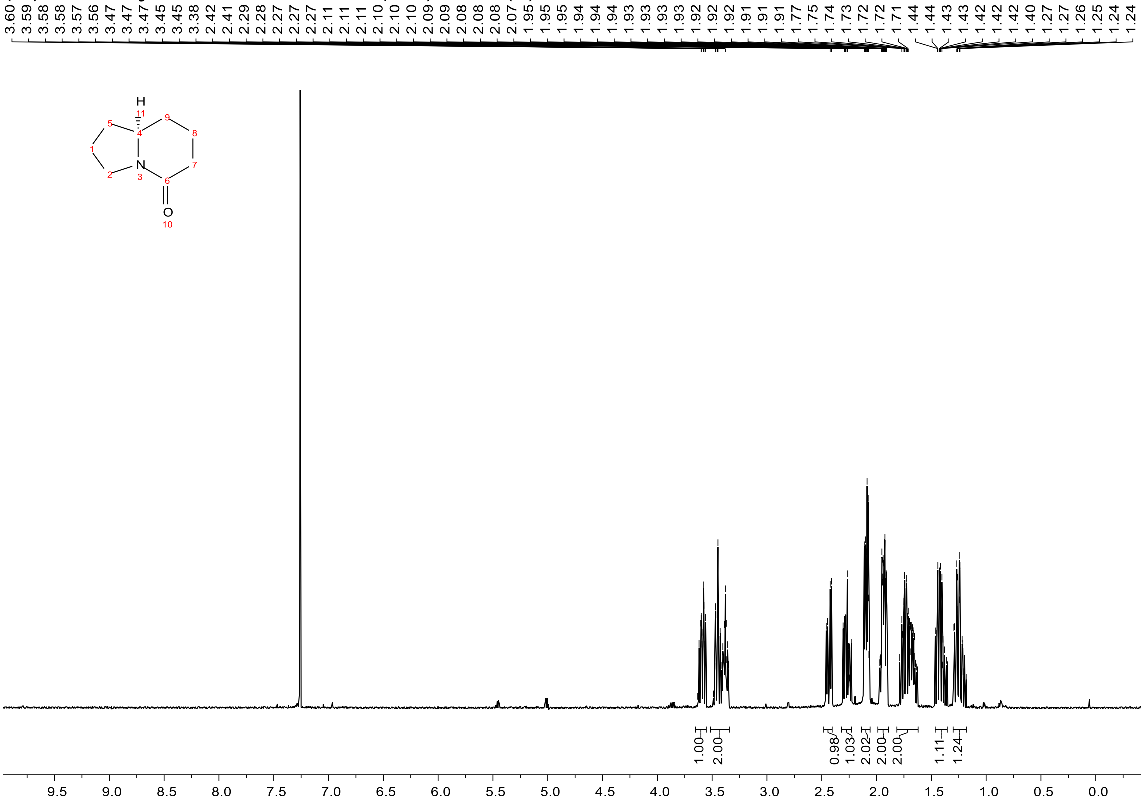

(*R*)-Hexahydroindolizidine-5(1*H*)-one (**8**) ^{13}**C NMR**

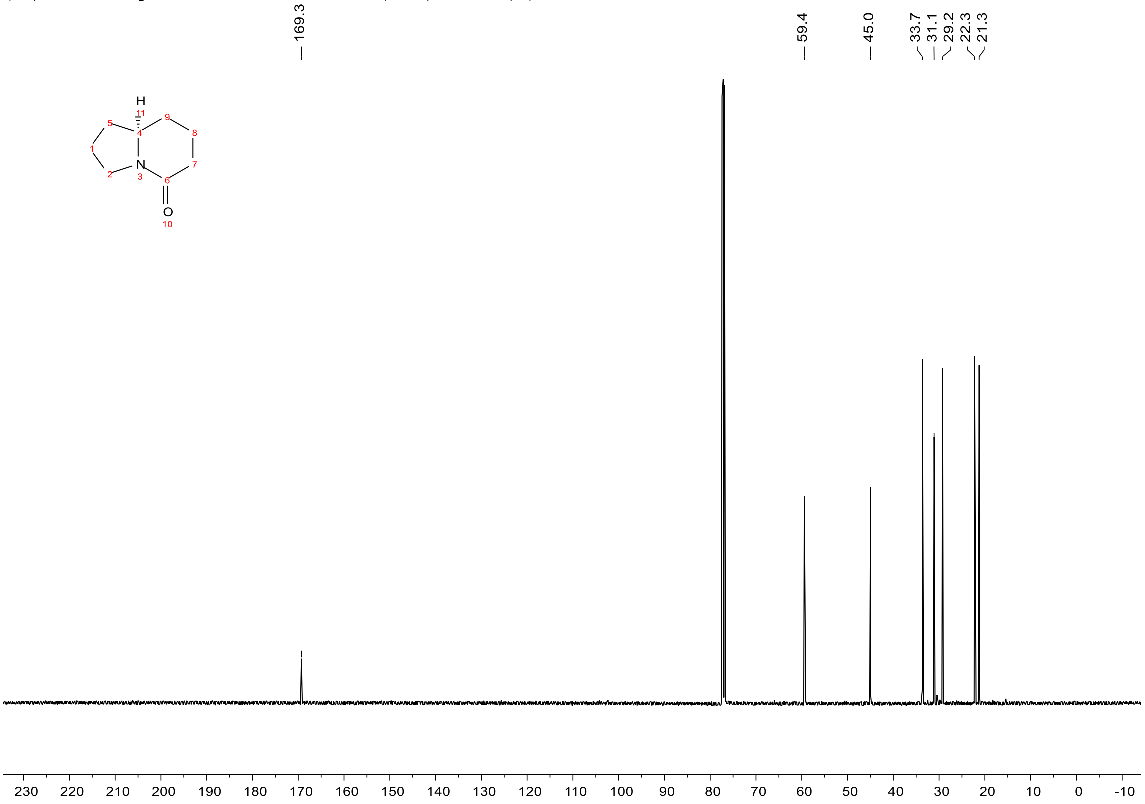

(8*S*/*R*,8a*S*)-8-Methyl-8-(4,4,5,5-tetramethyl-1,3,2-dioxaborolan-2-yl)hexahydroindolizidine-5(1H)-one (**11**) ^{1}**H NMR**

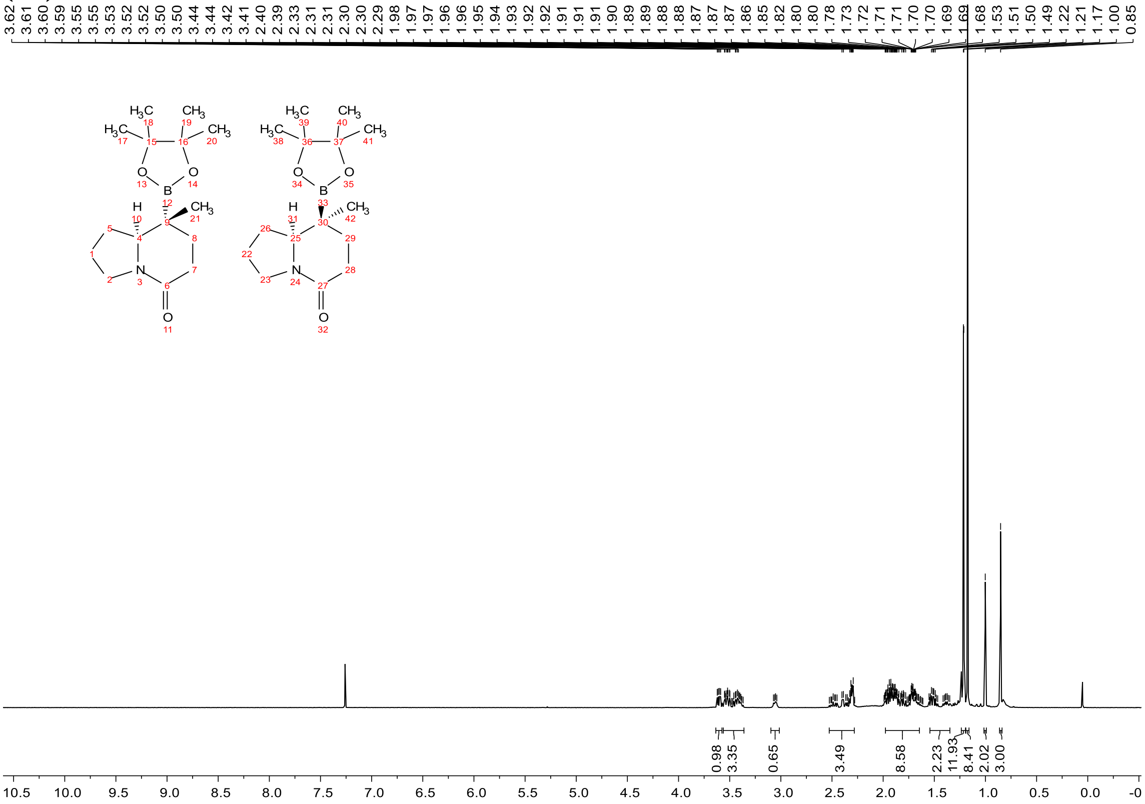

(8*S*/*R*,8a*S*)-8-Methyl-8-(4,4,5,5-tetramethyl-1,3,2-dioxaborolan-2-yl)hexahydroindolizidine-5(1H)-one **(11)** ^{13}**C NMR**

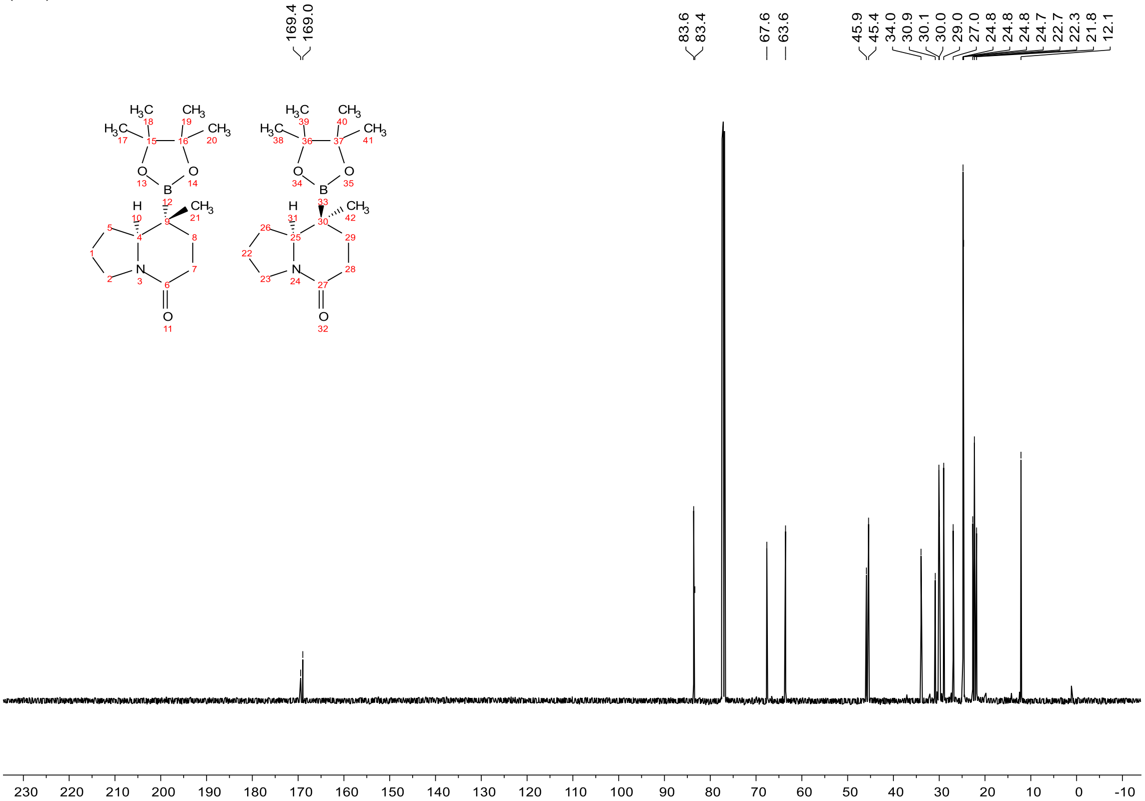

(8*S*/*R*,8a*S*)-8-Methyl-8-(4,4,5,5-tetramethyl-1,3,2-dioxaborolan-2-yl)hexahydroindolizidine-5(1H)-one **(11)** ^{11}**B NMR**

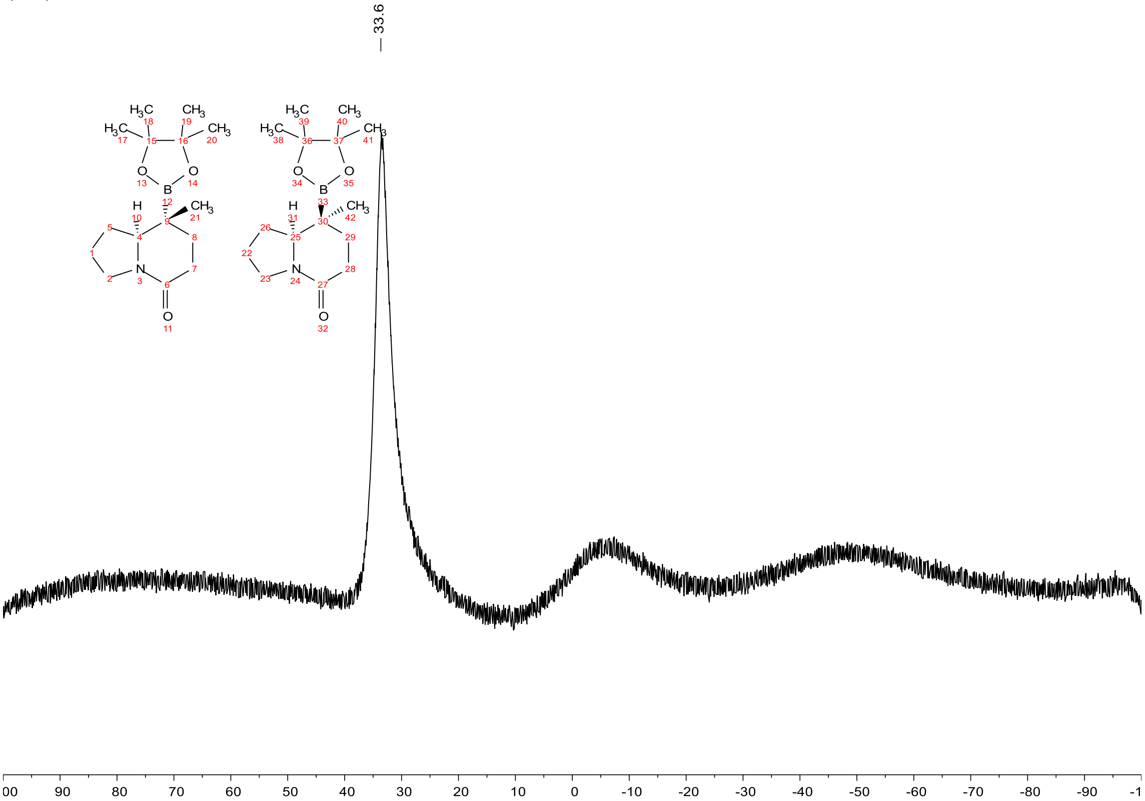

(8*S*/*R*,8a*S*)-8-Methyl-8-(4,4,5,5-tetramethyl-1,3,2-dioxaborolan-2-yl)hexahydroindolizidine-5(1H)-one (**11**) **^{1}H NMR** 1D-NOESY

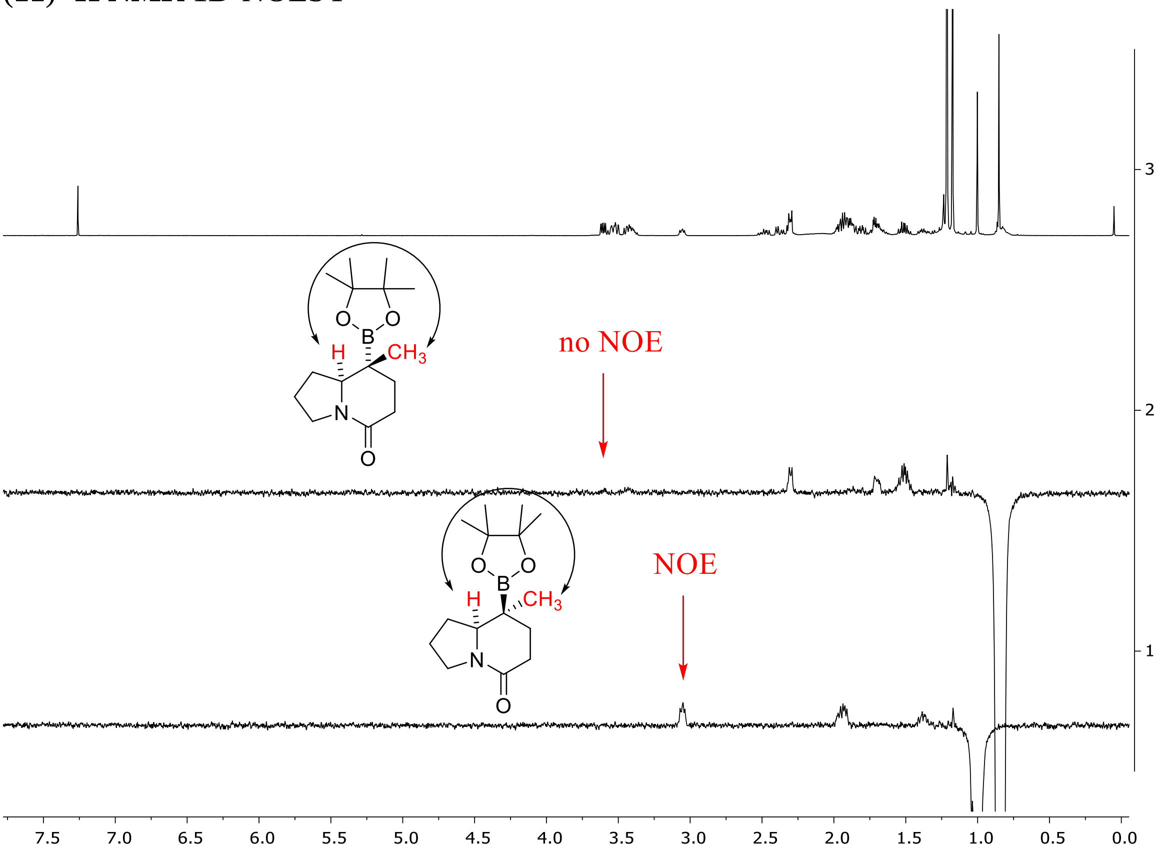

(8*R*/*S*,8a*S*)-8-Methylhexahydroindolizin-5(1)-on (**12**) **^{1}H NMR**

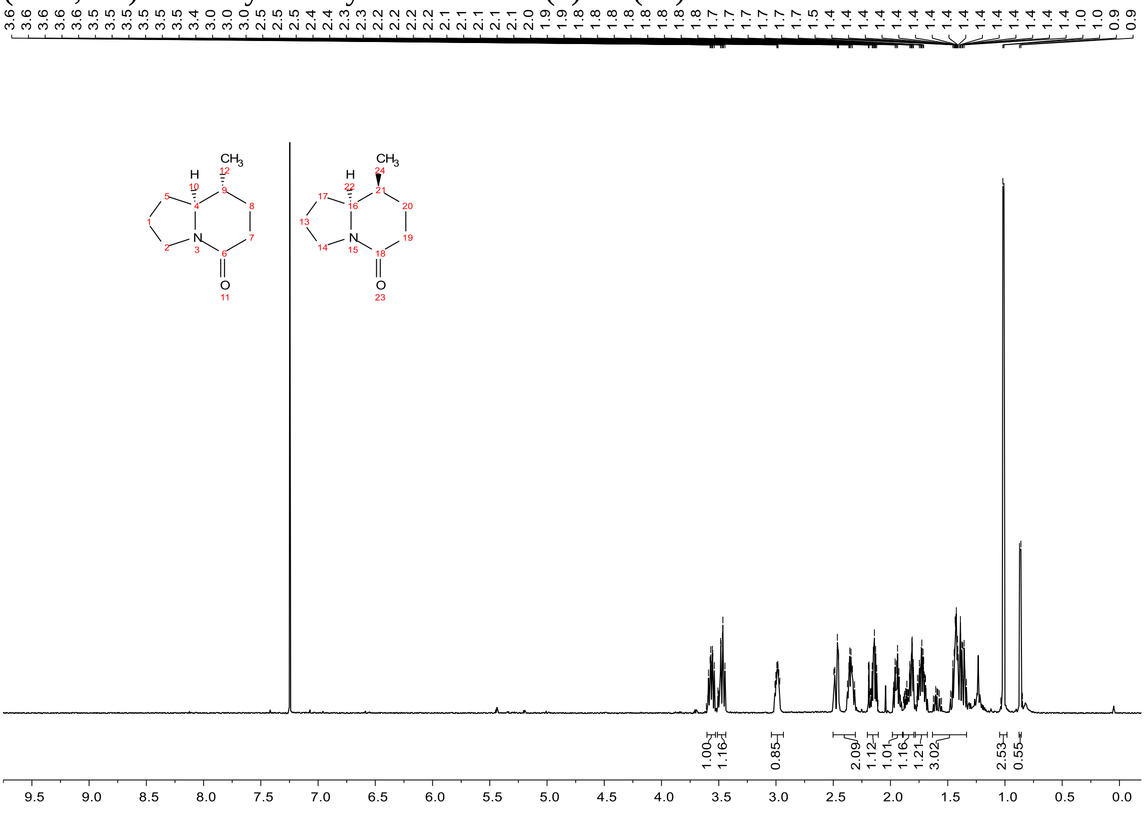

(8*R/S*,8a*S*)-8-Methylhexahydroindolizin-5(1)-on (**12**) **^{13}C NMR**

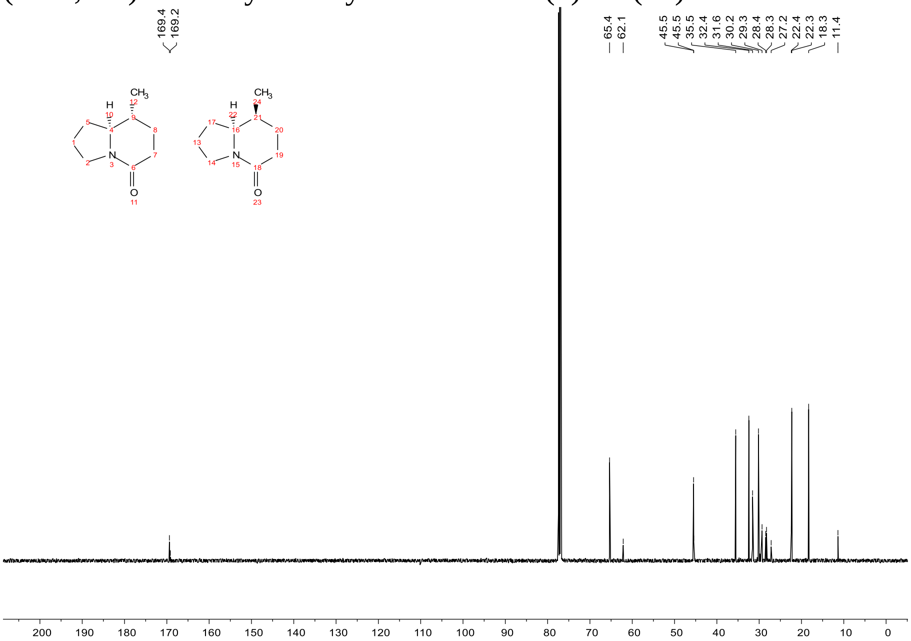

(8*R/S*,8a*S*)-8-Methylhexahydroindolizin-5(1)-on (**12**) **^{1}H NMR** 1D-NOESY

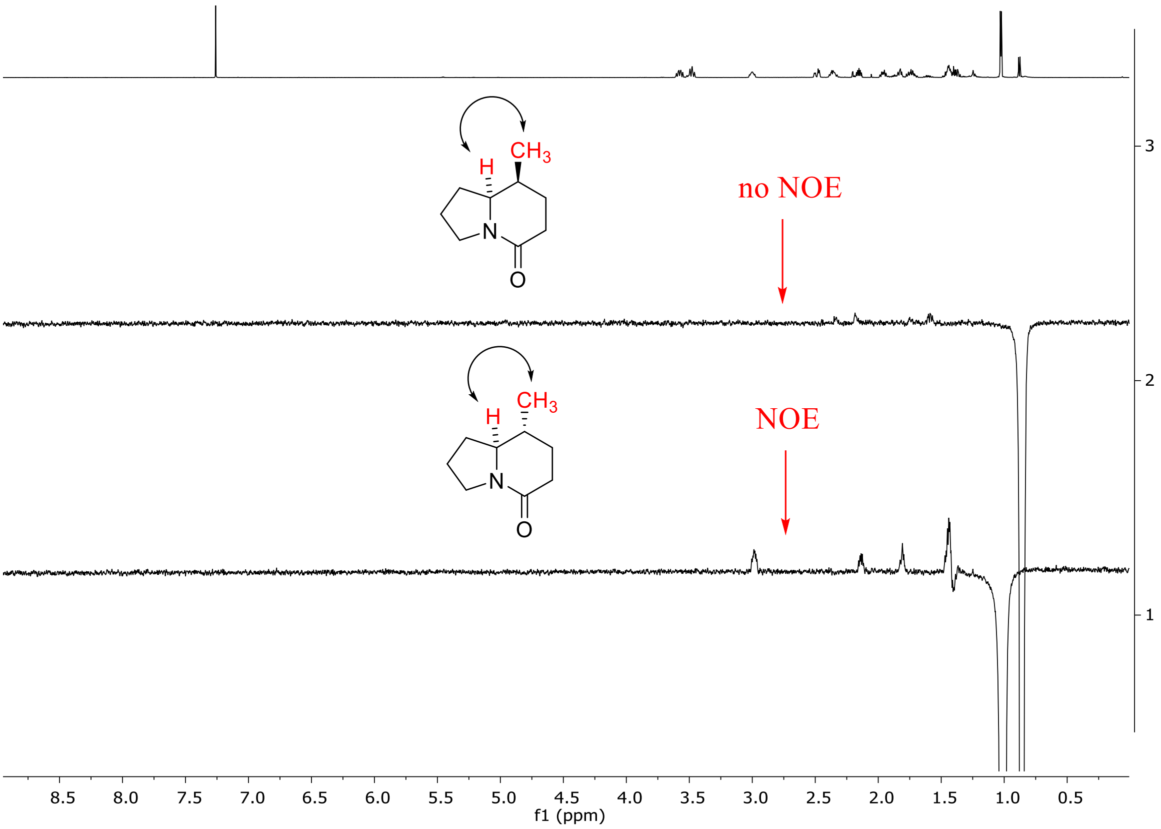

Supplement: Supplementary file 1 [file SC-010-C9SC02067E-s001.pdf]
